# Conserved brain-wide emergence of emotional response from sensory experience in humans and mice

**DOI:** 10.1126/science.adt3971

**Published:** 2025-05-29

**Authors:** Isaac Kauvar, Ethan B. Richman, Tony X. Liu, Chelsea Li, Sam Vesuna, Adelaida Chibukhchyan, Lisa Yamada, Adam Fogarty, Ethan Solomon, Eun Young Choi, Leili Mortazavi, Gustavo Chau Loo Kung, Pavithra Mukunda, Cephra Raja, Dariana Gil-Hernández, Kishandra Patron, Xue Zhang, Jacob Brawer, Shenandoah Wrobel, Zoe Lusk, Dian Lyu, Anish Mitra, Laura Hack, Liqun Luo, Logan Grosenick, Peter van Roessel, Leanne M. Williams, Boris D. Heifets, Jaimie M. Henderson, Jennifer A. McNab, Carolyn I. Rodríguez, Vivek Buch, Paul Nuyujukian, Karl Deisseroth

**Affiliations:** 1 Human Neural Circuitry program, Stanford University, Stanford, CA, USA.; 2 Department of Bioengineering, Stanford University, Stanford, CA, USA.; 3 Graduate School of Education, Stanford University, Stanford, CA, USA.; 4 Neurosciences Graduate Program, Stanford University, Stanford, CA, USA.; 5 Department of Psychiatry and Behavioral Sciences, Stanford University, Stanford, CA USA.; 6 Department of Neurosurgery, Stanford University, Stanford, CA, USA.; 7 Department of Neurology and Neurological Sciences, Stanford, CA, USA.; 8 Department of Psychology, Stanford University, Stanford, CA, USA.; 9 Department of Radiology, Stanford University, Stanford, CA, USA.; 10 Veterans Affairs Palo Alto Health Care System, Palo Alto, CA, USA.; 11 Department of Biology, Stanford University, Stanford, CA, USA.; 12 Howard Hughes Medical Institute, Stanford University, Stanford, CA, USA.; 13 Department of Psychiatry, Weill Cornell Medicine, New York, NY, USA.; 14 Department of Anesthesiology, Perioperative and Pain Medicine, Stanford University, Stanford, CA, USA.; 15 Department of Electrical Engineering, Stanford University, Stanford, CA, USA.

## Abstract

Emotional responses to sensory experience are central to the human condition in health and disease. We hypothesized that principles governing the emergence of emotion from sensation might be discoverable through their conservation across the mammalian lineage. We therefore designed a cross-species neural activity screen, applicable to humans and mice, combining precise affective behavioral measurements, clinical medication administration, and brain-wide intracranial electrophysiology. This screen revealed conserved biphasic dynamics in which emotionally salient sensory signals are swiftly broadcast throughout the brain and followed by a characteristic persistent activity pattern. Medication-based interventions that selectively blocked persistent dynamics while preserving fast broadcast selectively inhibited emotional responses in humans and mice. Mammalian emotion appears to emerge as a specifically distributed neural context, driven by persistent dynamics and shaped by a global intrinsic timescale.

Human beings experience sensory-evoked emotional states that modulate behavior both adaptively and maladaptively. These emotional responses are defined by exhibiting (i) behavioral, (ii) physiological, and (iii) subjective components (1, 2). While subjective experience can be assessed using human-specific social communication, affective behaviors that comprise the external displays of an emotional response can also be observed and characterized across diverse animal species, with shared properties including scalability, persistence, valence, and generalization (2–10). Emotional states that integrate rich information to shape behavior may be especially complex in the mammalian lineage, supported by the distinctive size and structure of the mammalian brain.

Large brains with distributed mapping of perception, cognition, and action are well-suited to organize and encode diverse information, but the same scale and complexity may pose difficulties for the rapid integration of available information to generate adaptive behavior. It is unclear from a brain-wide systems perspective how neural processes for establishing and maintaining a fully integrated and informed emotional state may operate on fast neurophysiological and cognitive timescales. Moreover, beyond this basic science perspective, clinical relevance of these processes is substantial since diverse psychiatric disorders involve altered emotional responses.

Recent advances using high-speed invasive and brain-wide neural recording in laboratory animals have revealed that internal states relevant to primary survival drives (such as hunger and thirst) can shape brain-wide neural activity and guide behavior by acting as a distributed neural context (11–17). However, these studies did not address the emergence of emotional states. Thus, we sought to explore when and where emotional states emerge using similarly high-speed, invasive, and global methods while recognizing that given the subjective quality of emotion, deep insight may also require obtaining verbal reports from human experimental participants. We therefore explicitly bridged mouse and human systems by defining temporally precise affective behavioral measures, clinically compatible pharmacological interventions, and deep brain-spanning intracranial electrophysiological readouts, which could all be similarly carried out in parallel in both human beings and mice, to investigate conserved principles underlying the emergence of lasting emotional states from brief sensory input. Patterns observed in these global neural activity screens, from species separated evolutionarily by many tens of millions of years, could have the potential to reveal conserved and ancestral solutions to the challenge of manifesting emotional states within the complex and distributed mammalian brain.

## Precisely timed affective behavior in mice and humans

**Fig. 1.**
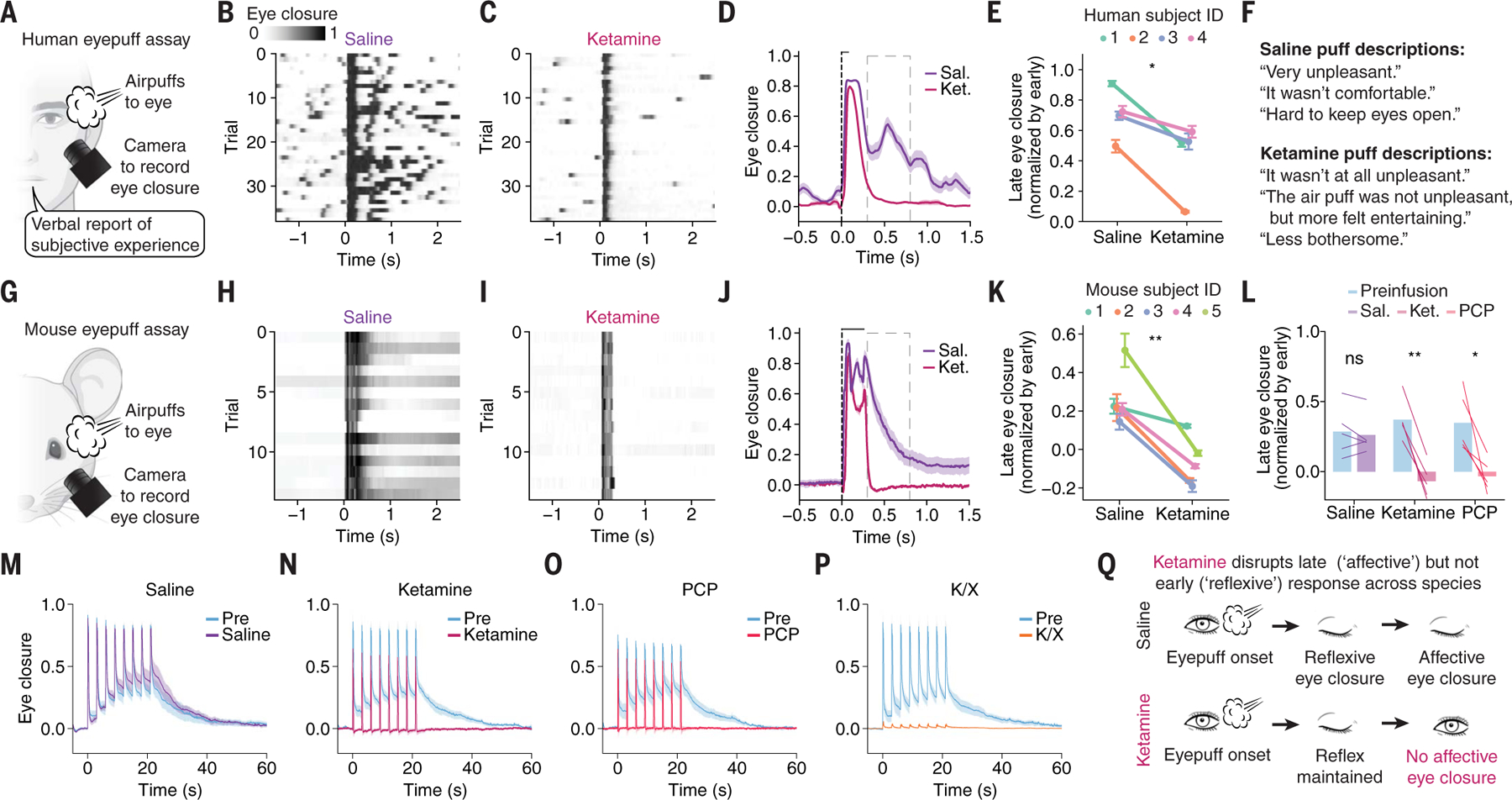
A cross-species measurement of rapidly evoked negative emotion. (**A**) Human eyepuff assay schematic. (**B**) Single-trial puff-aligned eye closure of example human participant during saline infusion or (**C**) ketamine infusion (0.5 mg/kg intravenous over 40 min) and (**D**) summarized across trials (mean ± SEM vertical dashed line, airpuff onset; horizontal bar, airpuff duration; dashed box, time window of interest corresponding to late eye closure, 0.3 to 0.8 s after airpuff onset). (**E**) Late eye closure, normalized by early eye closure (0.1 to 0.2 s after airpuff onset), decreases during ketamine (*n* = 4 human participants; each color represents a participant, mean ± SEM across trials). (**F**) Participants’ descriptions of experiences during eyepuff assay with saline or ketamine infusion. (**G**) Mouse eyepuff assay schematic. (**H**) Single-trial eye closure of example mouse during saline infusion and (**I**) ketamine infusion and (**J**) summarized across trials (mean ± SEM vertical dashed line, airpuff onset; horizontal bar, airpuff duration; dashed box, time window of interest corresponding to late eye closure, 0.3 to 0.8 s after airpuff onset). (**K**) Late eye closure, normalized by early eye closure (0.1 to 0.2 s afterpuff onset), decreases during ketamine (*n* = 5 mice; each color represents one participant, mean ± SEM across trials). (**L**) Effect of dissociative drugs ketamine (50 mg/kg) and PCP (20 mg/kg) on late, normalized by early, eye closure relative to preinfusion (*n* = 5 mice; bar height indicates mean). (**M**) Eye closure across sequence of eight closely spaced puffs, with administration of (M) saline, (**N**) ketamine, (**O**) PCP, and (**P**) general anesthetic dose of ketamine/xylazine cocktail (135 mg/kg ketamine with 15 mg/kg xylazine). (M) to (P) *n* = 5 mice, mean ± SEM (**Q**) Summary of ketamine’s cross-species impact on reflexive and affective components of the eyepuff eye closure response. ns (not significant), *P*-value ≥ 0.05, *P-value < 0.05. **P-value < 0.01. See table S3 for information on statistical analyses and sample sizes. [(A) and (G) partially created with BioRender.com.]

We first sought to define a precise and quantifiable behavioral assay that would capture the fast-timescale onset of an emotional state, and that would be comparably controllable across species from humans to mice. We identified a puff of air directed at the cornea (eyepuff) as a candidate stimulus with critical features including clinical safety, temporal precision, and repeatability. Eyepuffs are innately aversive stimuli across species (18–21) and evoke broad cortical neural responses in mice (11); moreover, these stimuli are temporally delimited, precisely targeted, compatible with head-fixed neural recording, and safe to administer to human beings (forming part of standard ophthalmological assessment). Finally, the mammalian behavioral response to an eyepuff (eye closure) is fast, scalable, repeatable, easily scored in an automated manner, and similar between mice and humans; moreover, the neural circuitry governing eyepuff-triggered reflexive eyeblinks is well-mapped (22–25). We hypothesized that eyepuff stimuli of sufficient intensity and duration would evoke a behavioral response with fast reflexive but also persistent affective features, and that these features could manifest in temporally separable neural activity patterns. To test these hypotheses, we developed a protocol for administering eyepuffs to human participants or mice (Fig. 1) under conditions that would allow measurements of behavioral responses and brain-wide screens for neural responses, as well as intervention with medications, in both cases (figs. S1A, S2A, and S3).

Under control conditions (during intravenous saline administration) in human participants (Fig. 1A), we observed a multiphasic response to the eyepuff (Fig. 1B), composed of a consistent and fast (“early”) reflexive blink followed by a more variable and extended (“late”) partial eye closure (fig. S1, B, C, and E). If the late eye closure indeed could be considered an affective behavior corresponding to an emotional response, we would predict that (i) human participants would describe a negative emotional state evoked by the sequence of eyepuffs and (ii) this described emotional state and the late eye closure would selectively be reduced by the dissociative medication ketamine, which causes emotional blunting (26), without blocking stimulus awareness.

We found that human participants described a response to the sequence of eyepuffs during the saline (control) session that was consistent with a negative emotional state, which includes properties of valence, arousal, persistence, and generalization (2, 27). The participants self-reported displeasure (“unpleasant”) and arousal (“it made me jump!”) (fig. S1F) and exhibited eye closure that extended beyond the duration of the stimulus, consistent with the self-reporting of a persistent state (Fig. 1, B, D, and F, and fig. S1, E and F). One participant (#1) exhibited substantial lacrimation in addition to persistent eye closure, revealing a generalized behavioral response with multiple physiological dimensions. Additionally, we indeed found that administration of ketamine diminished the eyepuff-triggered late eye closure while preserving the early reflexive blink (Fig. 1, C to E, and fig. S1, B and C), and subjective reports of the aversive quality of the stimulus were largely abolished by ketamine without blocking awareness of the eyepuffs (fig. S1F). Natural human response variability was of value here: participants with the strongest ketamine effect on the eye closure phenotype also reported greater impact of ketamine on the subjective experience of the eyepuff (fig. S1, E and F; leftmost columns versus rightmost). The response to the eyepuffs thus exhibited affective features of an emotional response, which were abolished by ketamine without blocking the reflexive stimulus response. Additionally, consistent with the hypothesized emotional blunting mode of action of the medication, acute ketamine induced a dissociative state in these same participants as rigorously quantified (alongside eyepuff administration) with the validated Clinician-Administered Dissociative States Scale (CADSS) rating scale for human dissociation (fig. S1D). Together, these observations align with our predictions, revealing that eyepuffs evoke a negative emotional state and that the late eye closure can be considered an affective behavior corresponding to this emotional response.

We anticipated that this approach, by design, should be extendable beyond human participants; we therefore developed a similar eyepuff protocol for mice (Fig. 1G and fig. S2A). We found that the multiphasic eye closure response observed in humans was also present in mouse behavior, and that dissociative drugs such as ketamine and phencyclidine [(PCP), uniquely enabled for mice] similarly reduced late but not early eye closure (Fig. 1, H to L, and fig. S2, B to D). Since mice cannot verbally convey internal emotional states, we relied instead on behavioral criteria to assess how single negative-valence eyepuffs relate to the induction of a longer-timescale emotional state. We measured behavioral changes during and after repeated sequences of eyepuffs in close succession, which was enabled by the increased number of trials that were possible with mice relative to human participants (because of clinical considerations). We found that late-phase eye closure in mice accumulated across a sequence of eyepuffs and decayed slowly after the final eyepuff in the sequence (Fig. 1M), demonstrating persistence (fig. S2E) and scalability (fig. S2, F and G). Using a separate reward-based assay, we found that eyepuff sequences elicited a persistent reduction in reward seeking (fig. S4), suggesting generalization of the induced state across behaviors. These features and the similarity between human and mouse eyepuff behavioral response suggested that the late phase mouse behavior can be considered an affective response corresponding to a negative valence internal emotional state. For brevity, we hereafter refer to the late phase of eye closure as affective and the early phase as reflexive, in both mice and humans. Consistent with this interpretation, we found that under either of the two dissociative agents, ketamine and PCP, reflexive eyeblinks remained present but affective eye closure responses were greatly reduced in magnitude, did not accumulate, and did not persist following the eyepuff series (Fig. 1, N and O). By contrast, general anesthesia, achieved through addition of xylazine to ketamine (a common combination treatment when general anesthesia is desired), abolished both reflexive and affective responses (Fig. 1P). Dose-response investigation showed that these robust dissociative effects begin around 35 mg/kg ketamine and 10 mg/kg PCP (fig. S2, H to J), consistent with prior work (28). Similarly consistent with prior dissociation assessments in mice (28), buprenorphine and diazepam did not show the same disconnection of reflexive and affective eye closure (fig. S5, A to H). Eyepuff results were repeatable across days (fig. S2, K and L) and generalized across experimental conditions, including with different air pressures (fig. S2, G and M) and in both male and female mice (fig. S2N). Mice do not proactively close their eyes under these conditions and therefore this assay was not modulated by anticipation of the precise timing of upcoming eyepuffs (fig. S2O).

A key barrier to studying neural dynamics of emotional states is that most such states are not precisely replicable in timing and quality and thus identification of neural correlates lacks the statistical power afforded by trial-averaging. The eyepuff assay, however, allows for repeatedly and reliably evoking a well-timed and quantifiable affective response corresponding to an emotional state. This affective response can be selectively modulated through pharmacological intervention while preserving a reflexive response that serves as a crucial counterpoint (Fig. 1Q). The eyepuff assay may thus allow for precise and robust mapping of relationships between neural activity and emotional states across species. We therefore sought to track neural dynamics during rapid-timescale emergence of emotional states by applying the assay during high-speed, brain-wide neural recording in both species.

## Brain-wide activity screen during emergence of affect in human beings

To seek neural activity patterns underlying the emergence of emotional brain states that might be shared across mouse and human participants, we designed and conducted high temporal resolution brain-wide activity screens in humans and mice. To guide the design and application of the exploratory screens, we first formally verified the importance of forebrain neural structures for the emergence of the affective response, since affect would be expected to not rely solely on peripheral nervous system or brainstem circuitry. We intercepted suprabulbar eyepuff-induced neural activity by inhibiting the mouse ventral posteromedial (VPM) thalamus, which transmits ocular-tactile input to the cortex (29), and found reduced affective eye closure relative to the reflexive response (fig. S6, A to J).

**Fig. 2.**
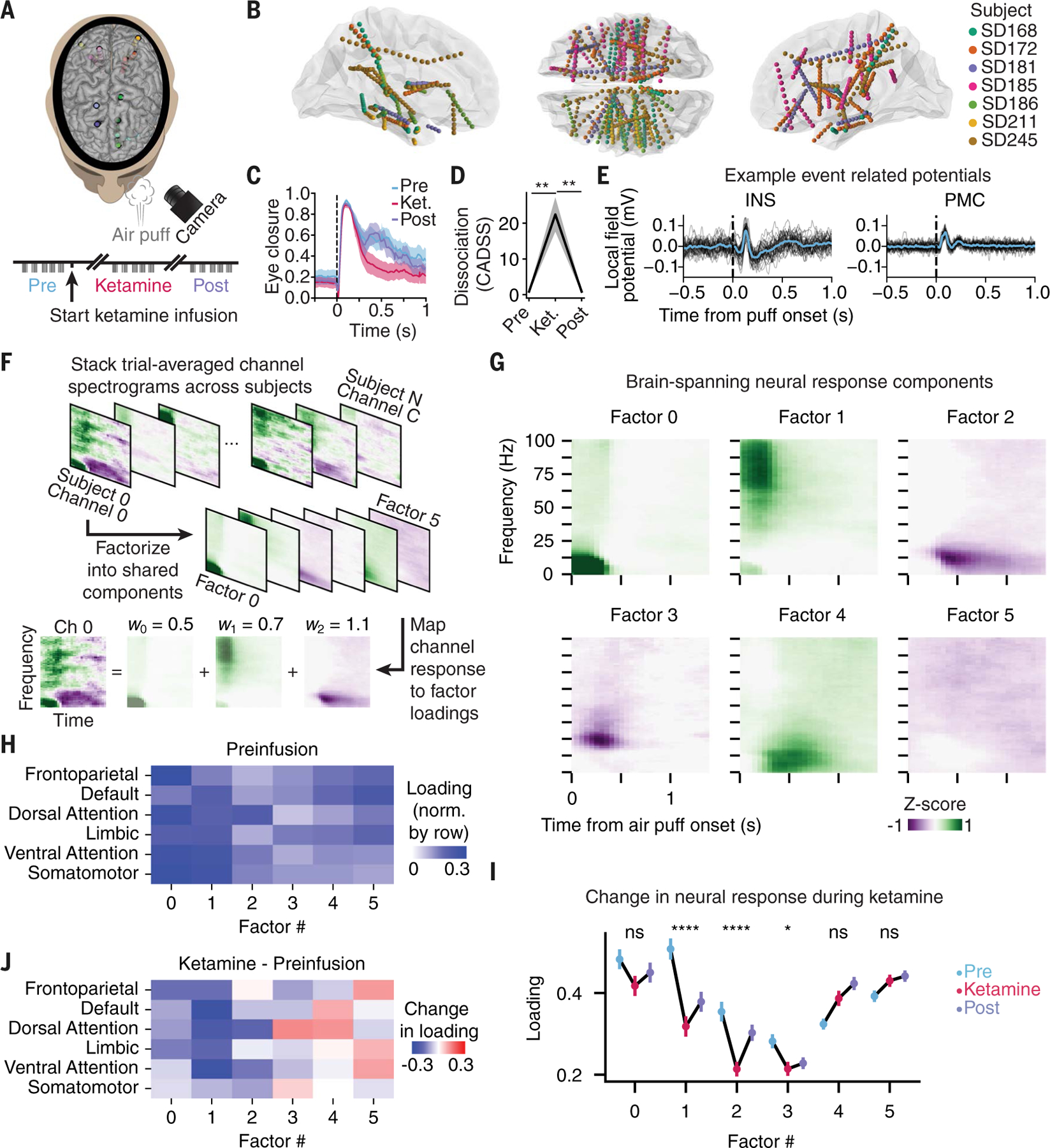
Human eyepuff-triggered intracranial electrical dynamics exhibit brain-spanning fast and slow components. (**A**) Recording simultaneous brain-spanning iEEG with eyepuff assay before, during, and after ketamine infusion. (**B**) Locations of recording sites. (**C**) Eye closure response to airpuffs before, during, and after ketamine infusion (*n* = 7 participants, mean ± SEM). Vertical dashed line indicates time of airpuff onset. (**D**) Clinician-Administered Dissociative States Scale (CADSS) before, during, and after infusion (*n* = 7 participants, mean ± SEM). (**E**) Example preinfusion single-channel event-related potentials from insula (INS) and posteromedial cortex (PMC), with mean shown in teal and single trials shown in gray. (**F**) Schematic of analysis approach for identifying shared spectrotemporal neural response components across participants and channels, using matrix factorization. Each channel’s response can be represented as a linear combination of factor activations (per-factor weights are “loadings,” denoted as *w*_factor_). (**G**) Spectrotemporal factors of neural eyepuff response, computed using pre- and post-infusion trials across participants (*n* = 7) and channels (*n* = 458), shown from onset of airpuff. (**H**) Loading of factors shown in G on Yeo7 cortical resting state networks (each row normalized by its sum). (**I**) Modulation of factor activity by ketamine. Factor loading shown before, during, and after infusion (mean ± SEM across channels), statistics compare infusion versus average of pre- and post-infusion. (**J**) Change in factor loading by ketamine for each Yeo7 resting state network. Blue indicates higher loading during preinfusion relative to during ketamine. ns, *P*-value ≥ 0.05. *P-value < 0.05. **P-value < 0.01. ***P-value < 0.001. ****P-value < 0.0001. See table S3 for information on statistical analyses and sample sizes.

Next, to map central nervous system neural dynamics corresponding to the stimulus-triggered affective response in human beings, we sought a recording method for measurement of electrical activity at high temporal resolution with brain-wide access (including in deep subcortical regions). We additionally required spatial resolution adequate for understanding local contributions to global patterns, as well as suitability for many hours of electrical recording across behavioral testing epochs, collection of verbal reports of subjective internal states, administration of psychiatric rating scales, and measurements prior to, during, and following administration of medications. The best available technique meeting all of these criteria was intracranial stereo-electroencephalography (iEEG) (30), for which we developed a suitable protocol (Fig. 2A and fig. S7A). We completed a full assessment (iEEG implantation followed by eyepuff administration alongside clinical ratings in the presence and absence of continuous intravascular infusion of the dissociative medication ketamine) in 14 human participants, of which 7 met inclusion criteria (fig. S8, A and B, and Methods). An additional cohort of 7 participants completed the study without the eyepuff administration but with ketamine infusion (fig. S7B). All participants received iEEG implantation with diverse electrode trajectories determined by individual clinical need (not study hypotheses), allowing an unbiased brain-spanning activity screen to be assembled across the population (Fig. 2B, fig. S7, C and D, and figs. S9, S10, and S11), with individual responses aligned in time (to temporally precise events such as the eyepuff) and aligned in space (to reference human anatomical maps).

As with the original non-iEEG-implanted outpatient participants (Fig. 1), behavior in the independent iEEG-implanted inpatient cohort displayed a multiphasic eyepuff response with a ketamine-elicited selective decrease in the affective phase of the response (Fig. 2C and fig. S8A); as expected, the iEEG-recorded participants also reported fully reversible induction of dissociation (measured with the CADSS instrument) during recordings (Fig. 2D). The simultaneous iEEG recordings revealed local field potential (LFP) signals exhibiting clear eyepuff-triggered event related potentials (ERPs) across the brain, exemplified by the broadband voltage traces from insula and posteromedial cortex (Fig. 2E). These ERPs could not be explained as neural signatures of eyelid closure nor of unexpectedness of the sensory stimulus: Despite similar magnitude of measured eyelid closure in spontaneous versus puff-triggered eyeblinks, the ERP pattern was not seen in spontaneous eyeblinks; further, the brain-wide eyepuff-triggered response pattern was absent in response to an unexpected auditory control, in which the eyepuff apparatus emitted the sound associated with the puff of air but was directed away from the participant (figs. S12, A to D, and S13D). Eyepuff-triggered LFPs across regions during preinfusion and ketamine infusion periods revealed that the eyepuff signal was rapidly and widely detectable across cortex (fig. S12E).

Ketamine was administered through a 40-min 0.5 mg/kg continuous infusion, eliciting dissociation as characterized in fig. S14, A and B, although we also explored either one- or two-step target-controlled infusions and either one or two step boluses as in fig. S14, C and D. We analyzed the brain-wide spectral effects of ketamine (fig. S15A) by region (fig. S15B), by individual channel (fig. S15C), and by Yeo7 resting-state network, a standard human cortical parcellation based on intrinsic functional connectivity (fig. S15D) (31). The time course of spectral changes that we observed matched the time course of the ketamine infusion pharmacokinetics (fig. S15, E and F), and these spectral changes were distributed across the brain (fig. S15, G to I).

We observed a complex pattern of puff-triggered spectrotemporal dynamics across recorded brain regions, with consistency observed across participants (fig. S13, A to D and fig. S16, A and B). This brain-wide response decreased in magnitude during ketamine infusion, recovered post infusion, and was not present during the auditory control (fig. S13). In general, shared features of spectrotemporal responses across regions—for example, similar <25 Hz patterns in motor cortex (MOT) and basal ganglia (BG) as shown in fig. S13A—suggested that spectrotemporal response dynamics across the brain may be low dimensional, with individual electrode channel responses drawn from a limited set of spectrotemporal response patterns. We therefore extracted a set of response patterns that spanned patients and regions, using a dimensionality reduction approach [nonnegative matrix factorization (NMF); see also Methods] applied across the preinfusion channel spectrograms (Fig. 2F). Factoring the responses into six components (Fig. 2G) was found to be optimal, with an explained variance of 0.47 on held-out trials and diminishing returns with more components (figs. S17, B and C, and S18A). These factors included bidirectional changes in spectral power, were not driven by single human participants (figs. S17C and S19A), and were globally distributed by region and Yeo7 resting-state network (Fig. 2G, figs. S19, B to D, and S20A) (31), albeit with distinctive and consistent patterns; for example, factors 0 to 2 loaded heavily onto attentional and somatomotor networks whereas factors 3 to 5 instead loaded heavily onto limbic, default-mode, and frontoparietal networks.

We next asked whether and how these factors were modulated when the affective behavior was suppressed with ketamine. Two of these factors (1 and 2) showed decreased representation during ketamine followed by post infusion recovery (Fig. 2I). We analyzed changes in the occurrence of these features within individual regions (fig. S19, B and C) and across Yeo7 resting-state networks (fig. S19, D and E). During drug infusion, occurrence of factor 1—a fast and partially sustained eyepuff-evoked elevation in high-frequency >50 Hz power—was suppressed substantially in limbic, dorsal, and ventral attention networks, whereas occurrence of factor 2—a delayed and sustained eyepuff-evoked reduction in mid to low frequency <20 Hz power—was suppressed in somatomotor, dorsal, and ventral attention networks (Fig. 2J). In summary, factor analysis revealed common spectrotemporal response patterns across brain regions to the eyepuff, suggesting brain-wide coordination; a subset of these patterns were reversibly disrupted by ketamine, suggesting that the disruption of affective responses by ketamine may also be distributed across multiple brain regions.

**Fig. 3.**
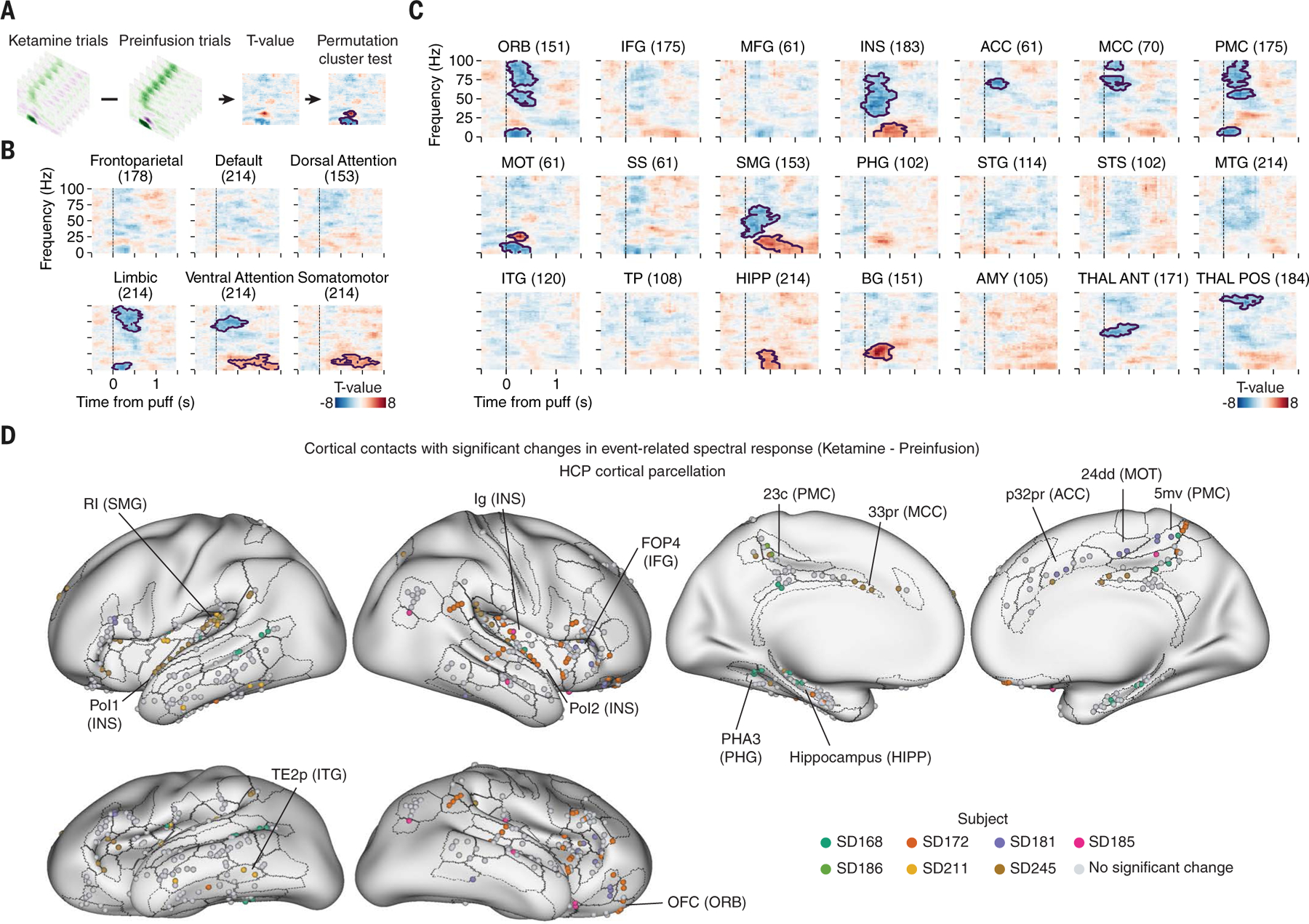
Selective disruption of human regional eyepuff-triggered dynamics by ketamine. (**A**) Schematic of permutation cluster test for identification of eyepuff-evoked spectrotemporal clusters that change significantly from preinfusion to ketamine. T-value (colormap) indicates size of change relative to variation across trials. Areas within black outlines indicate significantly changed spectrotemporal clusters. (**B**) Significantly changed clusters, by Yeo7 resting state network and (**C**) by brain region. See fig. S7C for full region names. Total number of trials across participants specified in parentheses; regions were sampled in different numbers of participants, yielding different numbers of trials per region. Blue signifies reduced power during ketamine. Vertical dashed lines indicate eyepuff onset. (**D**) Anatomical distribution of individual cortical contacts with significant changes, overlaid on Human Connectome Project (HCP) cortical parcellation. Representative fine-grained HCP areas with significantly changing contacts are indicated, with corresponding regions in parentheses. See table S3 for information on statistical analyses and sample sizes. ORB, Orbitofrontal Cortex; IFG, Inferior Frontal Gyrus; MFG, Middle Frontal Gyrus; INS, Insular Cortex; ACC, Anterior Cingulate Cortex; MCC, Mid-Cingulate Cortex; PMC, Posteromedial Cortex; MOT, Motor Cortex; SS, Somatosensory Cortex; SMG, Supramarginal Gyrus; PHG, Parahippocampal Gyrus; STG, Superior Temporal Gyrus; STS, Superior Temporal Sulcus; MTG, Middle Temporal Gyrus; ITG, Inferior Temporal Gyrus; TP, Temporal Pole; HIPP, Hippocampus; BG, Basal Ganglia; AMY, Amygdala; THAL ANT, Anterior Thalamus; THAL POS, Posterior Thalamus.

To more specifically identify and isolate regional and resting-state network spectrotemporal features that are consistently modulated by ketamine across trials and participants and whose change may therefore correspond to an alteration of the evoked emotional state, we performed an independent and complementary analysis: permutation cluster testing (Fig. 3A and Methods). Permutation cluster testing resolved ketamine-altered spectrotemporal clusters which reached statistical significance in the limbic, ventral attention, and somatomotor Yeo7 resting-state networks (contoured regions shown in Fig. 3B and fig. S21A), which were consistent with the earlier factor analysis and ketamine-elicited decreases in the magnitude of factors 0, 1, and 2 (Fig. 2J and figs. S21A and S20; note that factor 2 is negative-going so a decrease in its magnitude corresponds to an increase in power).

To more finely resolve the anatomical mapping of the changes in neural dynamics, we performed permutation clustering both by region (Fig. 3C and fig. S22A) and by the finer-grained 17-network resting-state atlas (31) (fig. S23, A and B). We also analyzed each channel, mapped anatomically onto the high-resolution Human Connectome Project (HCP) atlas (32) (Fig. 3D, table S1, fig. S24, A and B, and fig. S25A), and we further investigated possible structural connections through which brain-spanning activity may be coordinated (fig. S26). These analyses consistently identified distinctive ketamine-elicited spectrotemporal pattern changes in the insular cortex (INS), cingulate cortex (ACC, MCC), orbitofrontal cortex (ORB), posteromedial cortex (PMC), supramarginal gyrus (SMG), motor cortex (MOT), and subcortical structures including the hippocampus (HIPP), basal ganglia (BG, primarily caudate), and thalamus (THAL). Many of these are regions thought to be involved in pain perception (33), value encoding (34), self-related processing (35), and linking affect to sensation (36) (Supplemental Text 1). Of note, these regional changes in eyepuff neural response caused by ketamine were remarkably specific; spectrotemporal responses in many brain regions were not modulated by the ketamine administration when assessed with the measures used here, underscoring the fact that the eyepuff itself is well-represented across the brain whether or not ketamine is present.

This modulation by ketamine of spectrotemporal responses to the eyepuff occurred in participants in the context of ongoing dissociative experiences. We therefore sought deeper insight into the dissociative component of individual responses to ketamine. We found that diverse subjective experiences (fig. S14) were verbally reported by individuals on ketamine, although the study was not powered to investigate subscores within the CADSS dissociation scale (fig. S14, E and F). We observed that the overall CADSS dissociation score was correlated at the individual participant level with inhibition of the affective eyepuff response (fig. S14G); this finding is particularly relevant as unlinking of sensory awareness (which is maintained) from emotional responses (which are blunted) defines a core symptom of dissociation (28, 37). We further observed ketamine-induced low frequency oscillations in our human participants (fig. S27), consistent with previous reports (28, 38); here, we found that all participants with strong dissociation (CADSS score >10) exhibited low-frequency (<10 Hz) oscillations induced by ketamine (most notably in PMC and TP; we also observed that participants with high CADSS scores tended to exhibit a higher proportion of channels recording these low-frequency oscillations compared to participants with low CADSS scores: fig. S27).

In summary, precisely timed aversive eyepuffs consistently induced both reflexive and affective behavioral responses in human participants and were found to trigger complex global neural responses with considerable spectrotemporal and anatomical consistency. Shared features in specific regions and networks throughout the human brain (identified using either unbiased factor analysis or permutation cluster analysis, with similar outcomes using both methods) included fast-onset transient increases in separate bands of low- and high-frequency power, and delayed/persistent changes in an intervening band of mid- to low-frequency power. Suppression of the affective behavior in human participants with ketamine inhibited a specific subset of these neural responses, including the fast activation in limbic and ventral attention networks as well as the delayed changes in ventral attention and somatomotor networks, without altering representation of the eyepuffs in other major cortical and subcortical structures.

## Brain-wide activity screen during emergence of affect in mice

To more deeply explore how neural dynamics immediately following eyepuffs may accumulate into longer-timescale emotional state changes, we took advantage of the species-spanning nature of the assay system to provide key complementary information through mouse experiments. In particular, although our work in human participants offers verbal reports of subjective experience during emotional states as well as direct observation of human neural dynamics, neural recordings in mice would further offer single-neuron spiking activity across the brain, as well as greater numbers and dynamic range of experimental trials of aversive stimuli. In the mouse eyepuff assay, we showed that trials of eight puffs in close succession pushed the animal into a persistent negative emotional state in a scalable manner (Fig. 1, J to L). We therefore sought to characterize in mice the brain-wide, single-neuron spiking activity in response to single eyepuffs, across a series of eyepuffs, and during decay of the affective behavior following an eyepuff series.

**Fig. 4.**
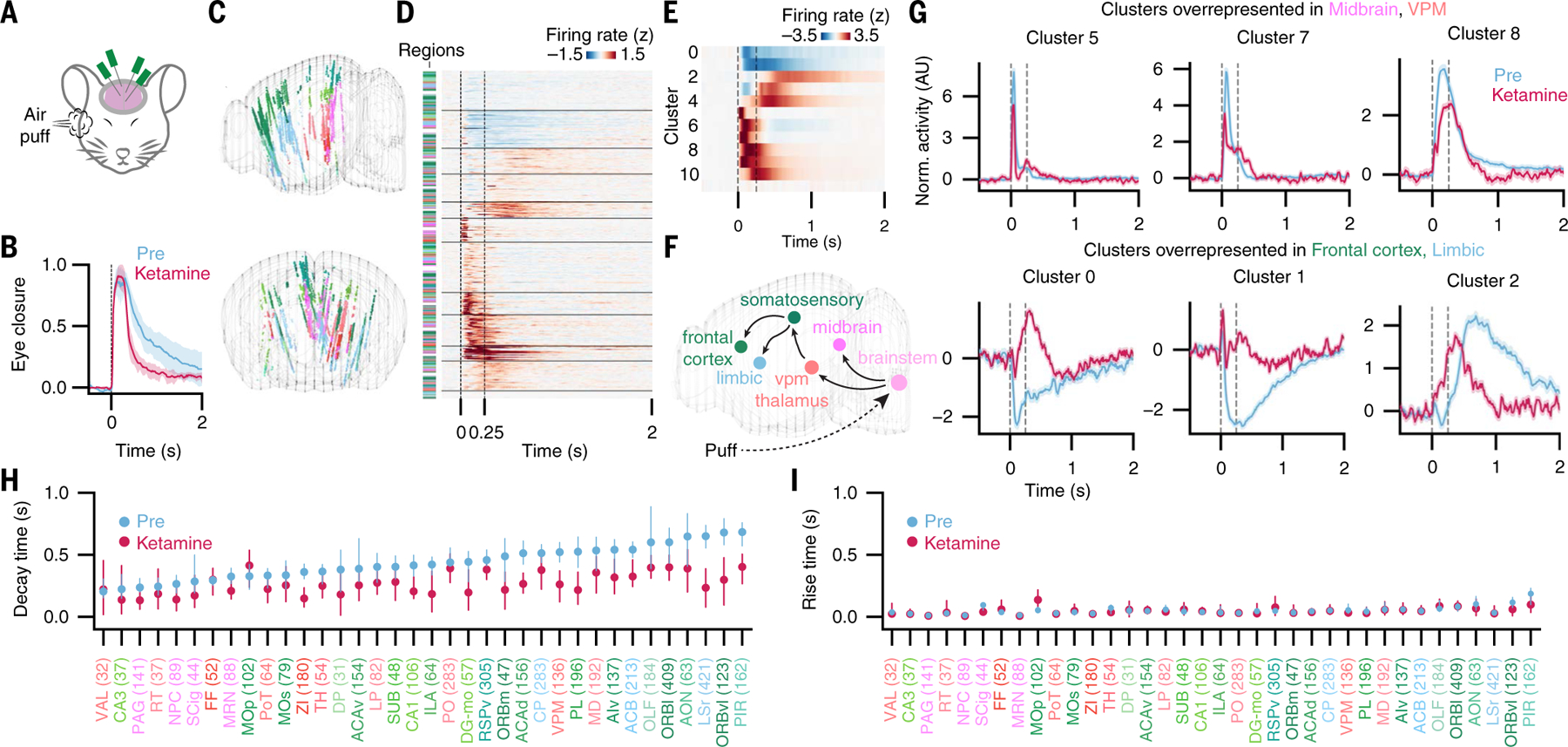
Brain-wide mouse eyepuff-triggered spiking dynamics have separable fast and slow components. (**A**) Recording high-density cellular electrophysiology with eyepuff assay before, during, and after ketamine infusion. (**B**) Eye closure in response to airpuffs in recorded mice (*n* = 13 sessions, 10 mice; mean ± 95% CI). (**C**) Locations of recorded units, colored by the Allen Brain Atlas colormap. (**D**) Airpuff-triggered cellular activity, z-scored and organized by unsupervised clustering, combining neurons from 13 sessions and 10 mice. Left vertical colorbar, region locations of individual units, colored by Allen Brain Atlas colormap. Vertical dashed lines, airpuff onset and offset. Horizontal black lines, division between unsupervised cluster identities. (**E**) Average cluster responses to airpuff. (**F**) Hypothesized information flow from airpuff onset, based on anatomy. (**G**) Average cluster responses before and during ketamine (*n* = 13 sessions, 10 mice; mean ± 95% CI). Clusters are numbered following (D) and (E) from top to bottom. (**H**) Decay time of puff-triggered neural activity by region, ordered according to pre-ketamine decays. (**I**) Rise time of puff-triggered neural activity. (H) and (I) median ± 95% CI. See table S3 for information on statistical analyses and sample sizes, and table S2 for region names.

We performed acute high density extracellular electrophysiology with three to four simultaneously inserted Neuropixels probes in mice undergoing the eyepuff assay (Fig. 4A), in the presence or absence of ketamine administration by remote injection (fig. S2A). In response to eyepuffs, mice showed affective eye closure that was reduced by ketamine (Fig. 4B and fig. S28A) (as had occurred in our human participants that were likewise undergoing intracranial recording). We obtained recordings from many thousands of single units throughout the brain, with simultaneous recording of dozens of regions (Fig. 4C, fig. S29, A and B, and table S2). We quantified ketamine-elicited brain-wide changes in spiking activity (fig. S29, C and D) and noted the prominent appearance of low frequency oscillations as we previously reported (28), with especially strong low frequency oscillations in PMC/retrosplenial cortex (RSP) and other correlated regions (fig. S29, C and D, and S30A). More broadly, ketamine altered single-unit correlation structure at rest by increasing within-region correlations such as within PMC and ORB (while also reducing correlations between regions; fig. S30A).

We next analyzed single-cell neural dynamics occurring in response to individual eyepuff events (Fig. 4D). Unsupervised clustering of perieyepuff timing histograms across all recording sessions revealed distributed groups of neurons with separable phases of eyepuff-triggered spiking (Fig. 4, D and E): Some clusters responded rapidly and briefly to the eyepuff whereas others responded persistently after either rapid (clusters 0 and 1) or delayed onset (cluster 2) (Fig. 4E). Each cluster was distributed across many brain regions (fig. S31A), with some regions overrepresented by a particular cluster (fig. S31B) and a shift in the representation when moving from midbrain to thalamus to frontal cortex. We hypothesized that progression of neural activity in response to the eyepuff might map onto hierarchies of anatomical circuitry, with fast responses in midbrain and thalamic regions and later responses in higher-order brain regions such as frontal cortex and limbic regions (Fig. 4F). Indeed, clusters overrepresented in midbrain (PAG) and thalamus (VPM) exhibited fast and sharp response dynamics, whereas clusters overrepresented in frontal cortex and limbic regions responded more slowly and persistently following the eyepuff (Fig. 4G and fig. S31A).

Guided by these results, we analyzed neural responses by region in two phases: time-to-rise after eyepuff onset, and time-to-decay following peak response (Fig. 4, H and I; see also Methods). We found that most regions exhibited fast rise times in response to the eyepuff (Fig. 4I), whereas decay times of regional neural activity were more variable and followed a general anatomical progression from subcortical to frontal cortical regions (Fig. 4H). Specific thalamic regions appeared to play a bridging role across timescales; though spiking activity in the ventral posteromedial (VPM) thalamus increased quickly in response to the eyepuff, decay from this peak was relatively late, consistent with its role in gating the affective response in mice (fig. S6). This pattern of persistence also held for the medial dorsal (MD) thalamus, which is strongly connected to ORB and INS (39) (both implicated in affective responses in our human participant work) (Figs. 2 and 3).

Administration of ketamine substantially altered the dynamics of certain neuron clusters but not others (Fig. 4G and fig. S31C): Clusters with more rapid preinfusion dynamics were minimally altered or unaltered by ketamine, whereas clusters with slower and more persistent preinfusion dynamics were substantially altered (Fig. 4G). On a regional basis as well, the rise times of average neural activity were not altered by ketamine (Fig. 4I and fig. S31, D to F), whereas regional times to decay from peak response were shortened (Fig. 4H and fig. S31G). These results revealed that information about the eyepuff rapidly spreads across the brain to most recorded regions in a multiphasic pattern, with transient responses primarily in subcortical regions and more persistent responses in frontal cortex and certain thalamic regions. These latter responses were shortened by ketamine.

**Fig. 5.**
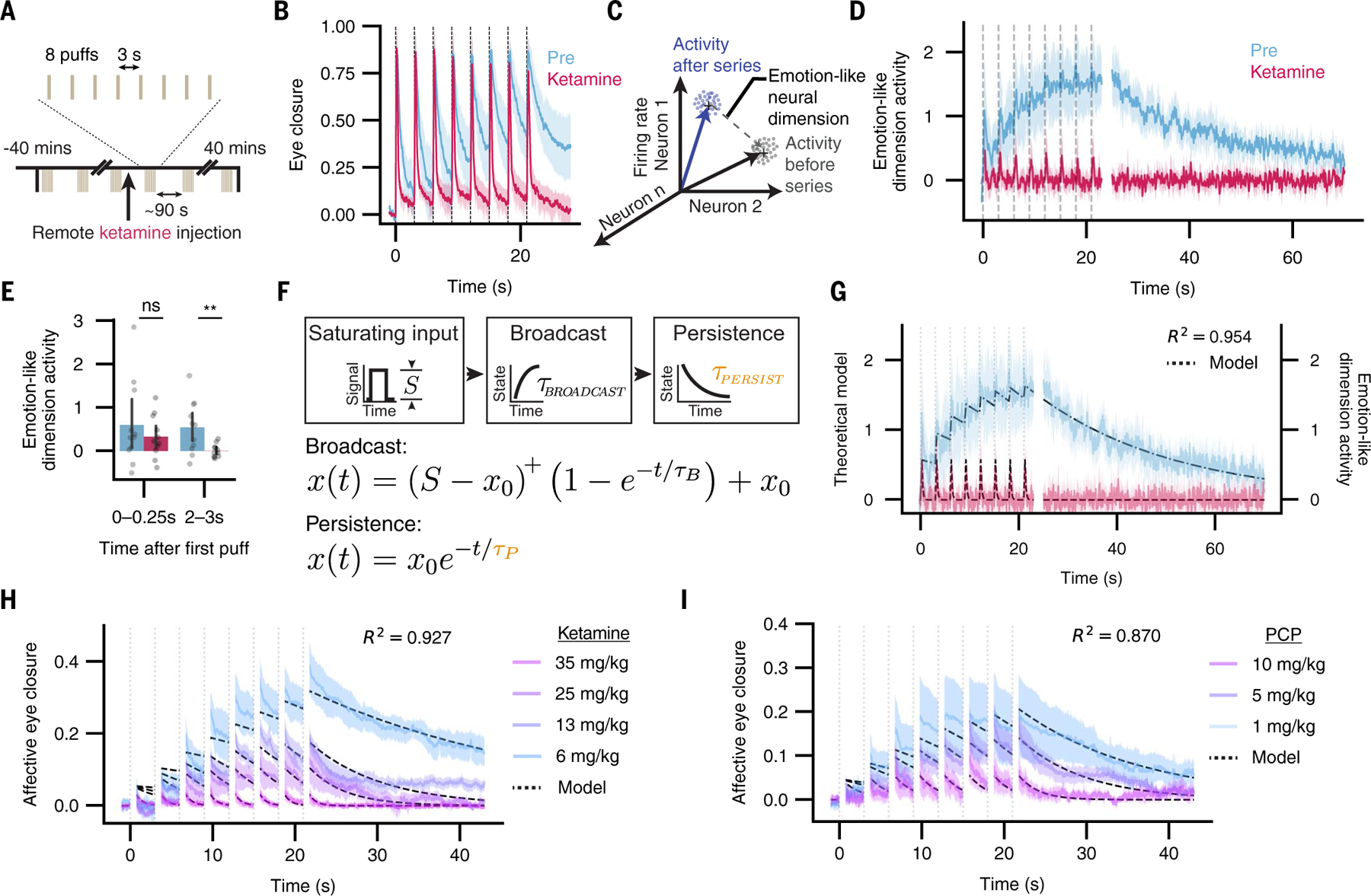
Mouse eyepuff-triggered neural activity accumulates to a persistent state with first order system dynamics. (**A**) Eyepuff protocol during neural recording. Each trial consists of a series of eight regularly spaced puffs, with twenty trials preceding and twenty trials following remote ketamine bolus infusion. (**B**) Eye closure across puff series in recorded mice (*n* = 11 sessions, 9 mice; mean ± 95% CI). (**C**) Schematic of coding dimension analysis to identify emotion-like neural population encoding. (**D**) Emotion-like population activity across puff series (*n* = 11 sessions, 9 mice; mean ± 95% CI). Dashed lines, airpuffs. The discontinuity at t = 23 s corresponds to the time window used to calculate the coding dimension, whose projection is definitionally high. (**E**) Comparison of emotion-like activity during the first puff presentation before and during ketamine, quantifying rapid (0 to 0.25 s after onset) and late emotion-like activity (2 to 3 s after first airpuff onset) epochs. Dots indicate sessions (*n* = 11 sessions, 9 mice). Bars indicate mean ± 95% CI. (**F**) Two-phase first order model of emotion-like neural state dynamics, as a piecewise time-dependent system with three key components: (i) a step input signal corresponding to each 250-ms eyepuff, which inputs to the system with a saturating positive magnitude *S*, which is the maximum attainable state value of the system; (ii) a broadcast phase, in which the state is driven upwards by the input at rate τ_BROADCAST_; and (iii) a persistence phase after input offset, in which the state decays at rate τ_PERSIST_. *x*_*0*_ is the state value at each phase transition (input onset or offset), and (*S* − *x*_*0*_)^+^ = max(0, *S* − *x*_*0*_), which enforces that the driving input is non-negative and that the state saturates at magnitude *S*. (**G**) Model fit to the emotion-like neural population activity (fitting performed jointly across all 11 sessions, 9 mice). The only parameter set to differ between preinfusion and ketamine conditions is τ_PERSIST_; other component values were fit jointly across preinfusion and ketamine. (**H**) and (**I**) Model fit to affective eye closure (masking out eye blinking in each 750-ms window post post puff-onset) for different doses of ketamine and PCP (*n* = 5 sessions and mice for each condition), with only τ_PERSIST_ as a free parameter. The other component values were fit jointly across doses of a given drug. ns, *P*-value ≥ 0.05. **P-value < 0.01. See table S3 for information on statistical analyses and sample sizes.

We hypothesized that the persistent neural responses following each eyepuff might give rise to a long-timescale emotional state, especially across sequences of eyepuffs (Fig. 5A). Consistent with our initial observations (Fig. 1, M and N, and fig. S2), recorded animals exhibited progressively accumulating eye closure behavior during each eyepuff series, which was abolished by ketamine (Fig. 5B). This behavior (scalable and persistent eye closure across the eyepuff series) might correspond to an emotion-like brain state comprised of population neural activity that outlasts the eyepuff series itself. To test this hypothesis, we extracted a linear dimension of neural activity from simultaneously recorded neurons that captured changes in brain state before versus after the eyepuff series (Fig. 5C; Methods). Measuring neural activity along this dimension revealed emotion-like properties of scalability through a saturating rise across the series of eyepuffs and of persistence through a decay lasting tens of seconds following each eyepuff series before returning to baseline (Fig. 5D). The neurons contributing to this dimension exhibited broad spatial distribution (fig. S32). This emotion-like neural dimension did not accumulate on ketamine (Fig. 5D), even though we observed transient increases along this dimension in response to each eyepuff (Fig. 5, D and E). These transient increases on ketamine were not different from those prior to ketamine, whereas late changes along the emotion-like dimension (seconds after each eyepuff) were reduced (Fig. 5E).

We observed that the emotion-like dimension of neural activity during and following the eyepuff series appeared consistent with a first-order (linear constant coefficient) system—that is, with dynamical behavior in response to perturbation dominated by an intrinsic time constant governing signal persistence. We conjectured that ketamine’s impact on emotional state dynamics might be the result of altering such an intrinsic time constant. To investigate this hypothesis, we constructed a two-phase first order dynamics model of the emotional state (Fig. 5F). We hypothesized that purely varying the model’s decay timescale, τ_PERSIST_, could capture the effects of ketamine on eyepuff-triggered emotional dynamics, and we therefore fit the parameters of the model to activity in the emotion-like neural dimension before and during ketamine infusion. Despite only varying the single parameter τ_PERSIST_ across preinfusion and ketamine conditions, we observed a close match between the model and the experimental data (Fig. 5G). Fitting an alternative single-phase model (in which τ_BROADCAST_ tracks equally with τ_PERSIST_) resulted in symmetric rise and decay dynamics and did not fit the neural data (fig. S33A); other alternative models in which ketamine modulated the input scale *S* or the broadcast rate τ_BROADCAST_ instead of τ_PERSIST_ also did not fit the data well, failing to capture the peri-puff emotion-like dimension activity during ketamine infusion (fig. S33, B to E). Finally, we assessed whether variation in decay timescale could quantitatively capture the partial disruptions to affective behavior induced by intermediate doses of ketamine. We found that varying τ_PERSIST_ alone across different doses of ketamine explained substantial variance across doses (Fig. 5H); indeed, the ability of this singular timescale parameter to explain internal state and behavior generalized to the structurally distinct dissociative agent PCP, for which varying τ_PERSIST_ was likewise able to capture behavioral variability across doses (Fig. 5I).

In summary, we have identified, measured, localized, and perturbed mouse neural population activity that accumulates in response to repeated aversive stimuli, scales with and persists with affective behavior corresponding to a negative emotional state, and closely corresponds in all these properties to a similar neurally and behaviorally defined state in human participants. We also found that a two-phase first order model robustly explains the neural dynamics of emotional state in this assay. We demonstrated that a single variable modulating timescale of state persistence substantially captures neural effects of intervention in this system. This model is consistent with the per-puff regional results of Fig. 4H, in which activity of most regions decays more rapidly during ketamine. Quantitatively longer per-puff decay timing in frontal and limbic regions suggests that these regions may play a special role in the establishment and maintenance of the persistent-population affective-state evoked activity across puffs, which is widely broadcast across most regions.

## Conserved emotion-related population dynamics across mammalian species

Our results thus far are consistent with emotional state emerging through a multiphasic process in which stimulus information is (i) rapidly and transiently broadcast throughout the brain and (ii) transformed into a more persistent and specific emotion-related activity trace in both humans and mice. We furthermore found that ketamine reduces this persistent activity trace in both species. Computational modeling of mouse neural dynamics and behavior revealed that the timescale of population neural activity is an important factor shaping this persistent activity in mice (Fig. 5, F to I).

We therefore hypothesized that specific disruption of the mammalian brain’s capacity for maintaining persistent activity might explain how agents such as ketamine disrupt eyepuff-elicited negative emotional states in both mice and humans. Moreover, this explanation might also cast light on general principles (independent of the eyepuff per se) governing the maintenance or termination of other emotional states. Indeed, intrinsic neural timescales, one way of measuring this capacity for persistent activity, have been shown to vary naturally depending on task demands, and to slow during task engagement, suggesting that variations in timescale might broadly impact neural processing (40–42). We sought to assess whether timescale plays a conserved role in emotional processing across mice and humans by explicitly comparing persistent population neural dynamics in both species. To do so, we leveraged the shared assay structure of single eyepuff responses and the simultaneity of activity recorded among single electrode contacts in human participants and among single neurons in mice.

**Fig. 6.**
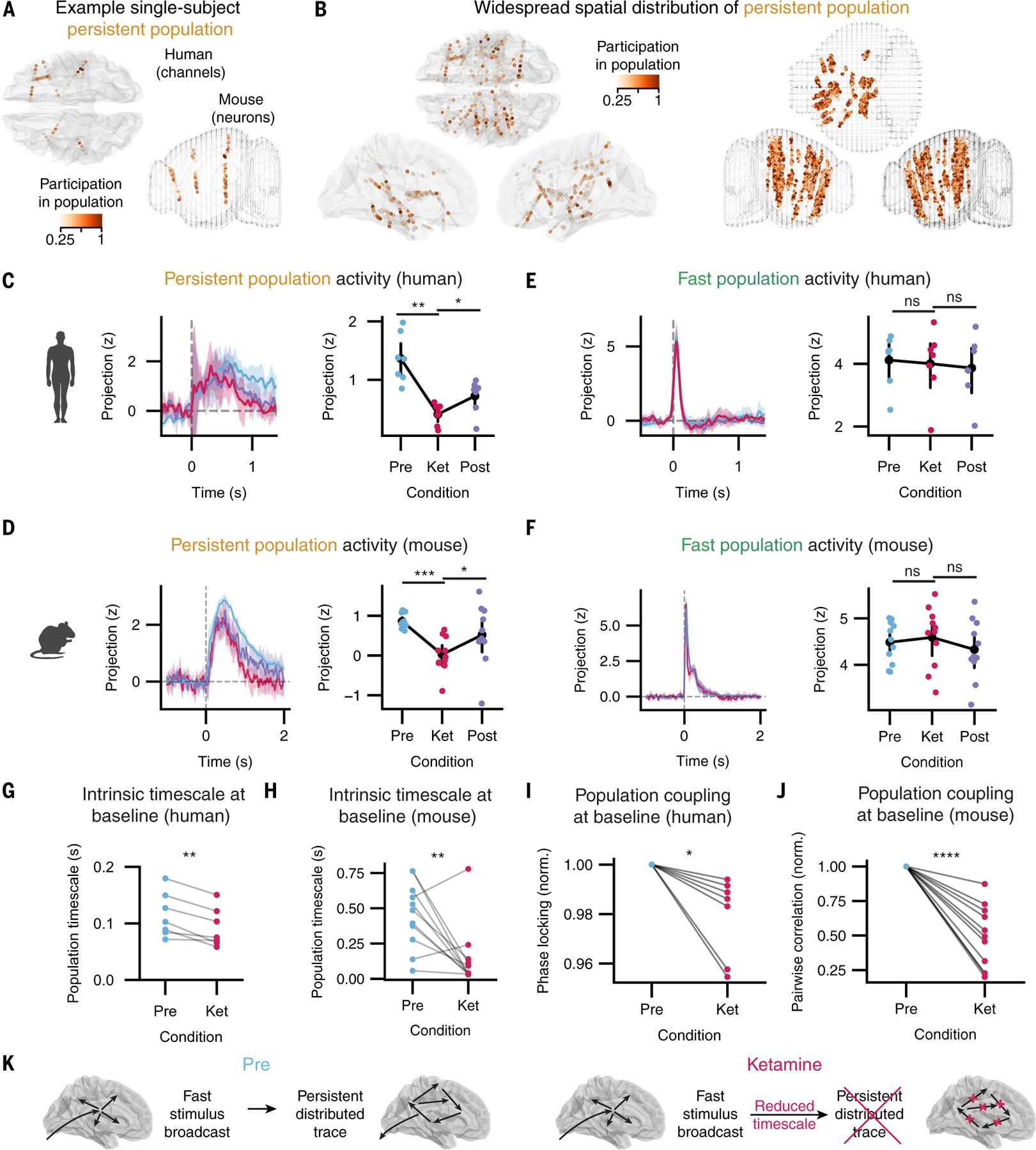
Persistent population dynamics disrupted by ketamine across species. (**A**) Puff-triggered persistent population is identified for each participant using a coding dimension analysis with simultaneously recorded sites (channels for humans, neurons for mice). Participation in the persistent population is measured as loading on the persistent dimension. (**B**) Anatomical distribution of the persistent coding dimension across participants (*n* = 7 human sessions from 7 participants, *n* = 11 sessions from 9 mice). (**C** and **D**) Persistent population activity is decreased by ketamine and then recovers for both humans and mice. Mean ± 95% CI. See Methods for details on time windows and quantification. Vertical dashed lines, airpuff onset. (**E** and **F**) Fast population activity is not significantly altered by ketamine in humans (coding dimension and mean activity computed during 50 to 100 ms after puff onset) nor mice (coding dimension and mean activity computed during 0 to 70 ms after puff onset). Mean ± 95% CI. (**G** and **H**) Intrinsic timescale at baseline (outside of eyepuff assay) of persistent population is reduced during ketamine relative to preinfusion in humans and mice. (**I** and **J**) Coupling at baseline (measured as mean phase locking between channels in humans and mean correlation between neurons in mice) within persistent population is reduced during ketamine relative to preinfusion for humans and mice. See Methods for detail on quantification. (**K**) Summary of proposed transformation of a brief stimulus into affective state and ketamine’s impact. We use a human brain to illustrate, but the same applies to the mouse brain. ns, *P*-value ≥ 0.05. *P-value < 0.05. **P-value < 0.01. ***P-value < 0.001. ****P-value < 0.0001. See table S3 for information on statistical analyses and sample sizes.

We began by identifying a neural activity dimension encoding persistent peri-eyepuff activity, across all simultaneously recorded individual electrode contacts (in human participants) and single neurons (in mice), which allowed for cross-species comparison of population dynamics (fig. S34A and Fig. 6A). This procedure reduced simultaneous population activity to a single affective-related value at each time point, in human participants and in mice, by assigning to each contact or neuron a higher weight if it exhibited greater activity during the affective response (relative to baseline). We found that the persistent dimension was not focally localized in either mice or humans; instead, a distribution spanning much of the brain was observed across both species (Fig. 6B, figs. S35, A and D, and S36A). Using this distributed persistent dimension, we analyzed the timecourse of brain-wide eyepuff-triggered responses and assessed the influence of ketamine (in both species) on these dynamics. Across species we observed a decrease, caused by ketamine, in eyepuff-triggered persistence that recovered post infusion (Fig. 6, C and D), whereas similar analysis of a dimension encoding fast eyepuff-triggered activity exhibited no decrease during infusion (Fig. 6, E and F, figs. S35, A to C, and S36, A and B). These results were observed in multiple frequency bands (fig. S37, A to C); significant and reversible ketamine-induced reduction of the projection along the persistent direction was observed in both delta and beta activity bands (fig. S37, A to C), and preinfusion dynamics of the latter exhibited greatest similarity between mouse and human (Fig. 6, C and D, and fig. S37, A to C).

We hypothesized that if ketamine modulates eyepuff-elicited emotion-related neural activity by accelerating intrinsic timescales, then the intrinsic timescale of spontaneous population activity at baseline should also be accelerated by ketamine. We therefore analyzed the intrinsic timescale of spontaneous activity in human participants and mice, operationalized as the decay rate of the autocorrelation of the previously identified persistent dimension (measured at baseline, entirely outside the eyepuff assay; for example, see fig. S34B). In both humans and mice, we observed that ketamine robustly accelerated the baseline intrinsic timescale of the population (Fig. 6, G and H).

Although there are many potential mechanisms through which ketamine could alter this timescale, intrinsic timescales have been linked to network coupling both within and across brain regions (41, 43–45). We therefore considered that one way in which ketamine might alter population timescale is through decreased network coupling within the persistent population. Accordingly, we analyzed functional coupling at baseline among the sites with the highest persistence; in both mice and humans, we discovered that ketamine decreased coupling among these sites (Fig. 6, I and J, consistent with fig. S30, and see additional control analyses in fig. S38). Together these behavioral and neurophysiological data suggest that emotionally salient brief stimuli give rise to an emotional state through a multiphasic process consisting of fast stimulus broadcast followed by a more persistent distributed trace that is shaped by a global intrinsic timescale. This global intrinsic timescale can be accelerated by agents such as ketamine, potentially through disruption of multiregional network interactions that are important for emotional states (Fig. 6K).

To explore the possible generality of this mechanism beyond negative-valence emotionally salient stimuli, we presented mice with sequences of an auditory tone instead of the eyepuff (mice do not increase eye closure during tone presentation; fig. S34C). Whereas fast tone-related neural activity was unchanged on ketamine (fig. S34D), persistent tone-related activity decreased on ketamine and then recovered (fig. S34E). We note that persistent population responses to the emotionally neutral tone were shorter than those to the emotionally salient eyepuff (fig. S34F), consistent with our model; we still could detect a ketamine-mediated acceleration in the intrinsic timescale of populations that (prior to ketamine) exhibited modestly persistent responses to the tone (fig. S34G), and under ketamine these populations exhibited reduced functional coupling even before tone presentation (fig. S34H). These results suggest that ketamine may generally accelerate population timescale through decreased network coupling in the mammalian brain, one consequence of which is disruption of persistent dynamics that are necessary for emergence of an emotional state. Indeed, a dynamical motif consisting of fast driving activity that transforms into recurrently mediated persistent activity may be a general feature underlying phenomena ranging from visual processing (46) to brain-spanning emotional processing.

## Discussion

We integrated subjective verbal reporting, deep brain-wide electro-physiological measurement, fast and repeatable behavioral readout, and medication-based intervention—together explicitly bridging human and mouse systems—to investigate, in the mammalian brain, processes underlying the emergence of a lasting emotional response from brief sensory input. We identified, in both species, a multiphasic neural response to aversive stimuli consisting of fast stimulus broadcast across the brain followed by a persistent and distributed neural activity trace with dynamics shaped by an intrinsic timescale. In mice, this persistent trace accumulated across sequences of stimuli into a scalable and persistent brain state governed by dynamics well-modeled as a first order system, with response to perturbation similarly dominated by an intrinsic time constant. We leveraged the emotion-blunting property of the dissociative agent ketamine to pinpoint which neural processes were associated with emergence of emotional response to the transient negative stimuli. We found in both humans and mice that ketamine preserved the fast stimulus broadcast but blocked the persistent trace. Moreover, we found in both humans and mice that ketamine accelerated the baseline intrinsic timescale of stimulus-related population neural activity. These results suggest that the effect of ketamine on emotional processing in mammals could stem from disruption of the brain’s capacity to maintain persistent activity. More generally, the acceleration of intrinsic timescale may dampen integration of information and thus blunt emotional responses.

The functionally distributed nature of the large mammalian brain poses a challenge to the unified organization of behavior. Sensory information must be made available to diverse neural circuits across the brain and these circuits must coordinate their activity such that they produce coherent action. The persistence and brain-wide distribution of emotion-like neural activity we observed here suggest that emotional brain states may function as a global context to inform local circuit function (13, 17), and such brain-wide representations of complex emotionally informed contexts may require the presence of certain global timescales in order to be maintained and employed (17). How these timescales are established remains an important open question; it is possible that population coupling changes can modulate global intrinsic timescale, and optogenetic approaches with the power to shift population coupling in specific ways, such as those used to induce dissociative-like states (26), may find utility in exploring this idea.

Our blockade of the affective response here was accompanied by reduced baseline activity coupling across the brain (Fig. 6, I and J, and figs. S30 and S38); these findings may thus link maintenance of emotional states to broader mechanisms of network-mediated signal persistence. Indeed, interactions between brain regions have been shown to underlie behaviorally relevant persistent activity, such as recurrent activity between anterior lateral motor cortex (ALM) hemispheres (47), or between the ALM and thalamus in the context of a delayed-response whisker-detection task in mice (48), or between the PFC and MD thalamus in a rule-based sensory selection task (49). Multiregional band-limited oscillations, which we observed to be altered by ketamine across species, have also been hypothesized to be important in interregional communication (50–53). Although we observed widespread emotion-related activity in our eyepuff assay, we found particularly conserved patterns of activity centered on the ORB, PMC, and INS, which could interact to shape the persistent network dynamics. We note that our observations do not exclude the additional participation of other regions not subject to dense recordings in our study population, including those such as the amygdala, that are known to be involved in processing aspects of emotional state quality or valence. Future work will build upon the system-level dynamics we present here to further investigate network mechanisms for coordination of emotional states across brain regions and indeed across the body (54).

Dependence of persistent network activity on NMDA receptors has been theorized in the context of working memory (55) and demonstrated in nonhuman primates (56), in a task that required maintenance of information across a 2.5-s delay period. Ketamine and PCP are both NMDA receptor antagonists and have both been used as models for certain aspects of schizophrenia symptomatology (26, 57–59). We previously reported the role that dissipative stochastic fluctuations may play in brain state transitions relevant to motivation (17), hypothesizing a link to psychiatric diseases such as schizophrenia. Our results here support the idea that loss of persistence may drive a general inability to stabilize brain states, which may be especially relevant in diseases such as schizophrenia, wherein symptomology often includes disorganized patterns of thinking (thought disorder) and reduced expression of emotion (flattened affect) (37).

We anticipate that increased understanding of stimulus-triggered persistent dynamics may additionally illuminate aspects of the etiology and treatment of psychiatric conditions which could instead be characterized by overstabilized brain states. Indeed, although longer intrinsic timescales may allow more coupling among participating regions for inclusion in brain-wide representations, these timescales could also contribute to maladaptive recruitment of circuitry in neuropsychiatric diseases (such as centralized pain, depression, obsessive-compulsive disorder, borderline personality disorder, and eating disorders, which all may involve excessively potent or intrusive interregional communication). Finally, distinct from determination of such regional recruitment patterns, maladaptively long timescales could cause impairment in circuitry important for temporally demanding tasks such as language and social-information processing. For example, autism spectrum disorder has been linked to hyperexcitable neurophysiology (extending to epilepsy in many cases), which in promoting excitatory coupling could contribute to longer intrinsic timescales. These longer timescales would be expected to give rise to excessive signal accumulation and difficulty in following rapidly changing input, for example, causing challenges in processing high bit-rate streams of social communication, whereas slow or static information processing tasks would reveal relatively reduced impairment and potentially even enhanced performance.

## Materials and Methods

### Eyepuff assay (human)

#### Eyepuff rig.

Ocularly-targeted air puffs were delivered using a clinical tonometer (Keeler Pulsair Desktop Non-Contact Tonometer for experiments with no neural recording, and Keeler Pulsair intelliPuff Tonometer for experiments with neural recording). The duration of each air puff was ~60 ms, and was not adjustable. Videos of each eye were recorded at 200 frames per second with two cameras (Basler ace acA1300-200uc Color USB 3.0 Camera), recorded simultaneously on a single computer using Bonsai-rx (60). Electronic control of the clinical device was not accessible, so we used an Arduino-controlled solenoid (Adafruit, driven by a BOJACK L298N Motor DC Dual H-Bridge Motor Driver) to manually push the Demo button to release each air puff. Sync LEDs (1 mm diameter, from Green Stuff World) were illuminated during each puff, visible to the recording cameras. Participants placed their head on a headrest such that their forehead was 15 mm away from the air puff nozzle, and the nozzle was aligned manually to target the left eyeball. Participants were told: “You will receive air puffs across a period of approximately five minutes. Please stare straight ahead.” Forty total puffs were delivered in blocks of eight puffs, with a 3 to 8 second interpuff interval, and 35 second interblock interval. The block structure was designed so as to not overwhelm the human participants, and the variable interpuff interval was selected so that the participant could not predict precisely when each puff would occur, with a minimum interpuff interval determined by the tonometer specifications.

Videos were analyzed using DeepLabCut (61) to extract eye closure. For each session, at least 20 video frames were manually labeled to specify the position of the top and bottom eyelids, and these labeled frames were used to train a Resnet50 tracking model, with which the position of the eyelids was identified for all frames. Additionally, the state of the sync LED was extracted from each video frame and used to identify the time of each eyepuff. Each trial was manually inspected to ensure that an air puff actually occurred. Trials with no air puff in any session were excluded from all sessions in that participant (to maintain consistent numbers of trials across sessions). Additionally, the first trial of each session was excluded from analyses.

#### Drug infusion.

For experiments without neural recording, as part of a larger, separate study (clinicaltrials.gov # NCT03475277) (62), we enrolled 4 nonclinical adult participants aged 22 to 51. We enrolled the participants in a date range from August 22, 2021 to September 8, 2021. The Stanford Institutional Review Board-approved study inclusion and exclusion criteria included minimal clinical symptoms and at least 2 previous ketamine uses, among others – all participants documented written informed consent and passed these screening criteria. As a cross-over, randomized study, participants received placebo saline and 0.5 mg/kg ketamine across two visits, with a random infusion order for each individual. The participants, licensed study clinicians (who monitored the infusions and performed safety assessments), research nurses (who placed the intravenous (IV) peripheral catheter), and research coordinators (who performed study assessments) were blinded to the infusion ordering. Because ketamine infusion is a fairly potent intervention, we expected that the effect size should be fairly large (with a Cohen’s d on the order of 1 to 1.5) with reasonably high correlation between participants, and taking into consideration the within-participant study design, we thus expected that 4 participants should be adequate to observe the hypothesized effect of the drug. As part of the larger study, participants also participated in a third visit, where 0.05 mg/kg ketamine was infused, but these sessions are not included in the current study. Each patient received both infusions across the duration of the trial. The choice of IV ketamine as the method of administration was informed by the dosage’s predictability and complete bioavailability. To mitigate effects of prolonged drug wash-out, 10-14 days separated each infusion visit. Prior to morning infusion visits, participants were asked to fast to reduce risks of emesis. Upon arrival to the Clinical Trial Research Unit at Stanford University, participants completed a pregnancy test (if applicable), completed a urine drug test, had baseline vitals measured, and received a peripheral IV catheter. After being accompanied to the Stanford Center for Cognitive and Neurobiological Imaging, participants met the study clinician and underwent the infusion. Over a period of 40 min, an elastomeric pump (Braun Easypump ST/LT) delivered either 0.9% normal saline (Mariner Advanced Parhamcy Corp, San Mateo, CA) or racemic IV ketamine (0.5 mg/kg). To ensure participant safety during the infusion, the team monitored and recorded participant pulse oximetry, pulse, and blood pressure at intervals of 10-15 min. The eyepuff assay was administered approximately 30 minutes after the start of infusion. The Clinician-Administered Dissociative States Scale (CADSS), a 23-item questionnaire, was assessed by a study clinician 40 minutes after the start of the infusion.

For experiments with neural recording, as part of a larger study (clinicaltrials.gov, # NCT04861051) we recruited from the community clinical adult epilepsy patients who were undergoing Phase 2 extended-stay monitoring in Stanford Hospital. All participants passed the study screening procedure and endorsed baseline symptoms permissible by the study inclusion and exclusion criteria. The Stanford Institutional Review Board approved the study protocol, and participants documented their written informed consent prior to entry into the study. Participants were clearly informed that they could discontinue participation in the study at any point during or between sessions. The study consists of five total visits. During a screening visit, after providing informed consent to participate, a physician completed the following assessments: review of psychiatric and medical history, Hamilton Depression Rating Scale (HAMD-17), Montgomery-Asberg Depression Rating Scale (MADRS), Clinician-Administered Dissociative States Scale (CADSS), Columbia Suicide Severity Rating Scale (C-SSRS), and a review of concomitant medications. If deemed eligible, after electrode implantation and participant recovery, a baseline visit occurred at the Stanford Medical Center Epilepsy Monitoring Unit, with the following assessments: eyepuff assay, HAMD-17, MADRS, CADSS, C-SSRS, Brief Pain Inventory (BPI), urine toxicology, vital signs measurement, review of adverse events, review of concomitant medications, Snaith Hamilton Pleasure Scale, the Revised Social Anhedonia Scale, and confirmation of eligibility for ketamine infusion according to inclusion/exclusion criteria. Video and audio of the participant was recorded throughout the baseline visit. The ketamine infusion visit was scheduled in consultation with neurosurgical and neurological clinical care teams. Participants were restricted from solid food within 6 hours and clear liquids within 2 hours preceding infusion. Ketamine was infused through indwelling intravascular access. For most participants, 0.5 mg/kg ketamine was infused over 40 minutes. For a subset of patients, target-controlled infusion (TCI) was instead used, a well-established method to achieve stable blood levels for a variety of commonly used drugs. TCI administration was medically directed and overseen by an anesthesiologist. TCI consisted of up to two sequential stages, with the first stage targeting a ketamine blood level of 0.13 mcg/ml, and the second targeting 0.26 mcg/ml. Two participants received bolus infusions of 0.35 mg/kg ketamine. Ketamine was administered adjunctively to current medications. Video and audio of the participant was recorded throughout the ketamine infusion visit. The eyepuff assay was administered preceding infusion, at approximately 30 minutes after the infusion start, and 110 minutes after infusion start. For a subset of participants, an audio control was administered at 115 minutes after infusion start, consisting of the complete eyepuff protocol, but with the apparatus moved to the side of the participant such that the air puff was not directed at the participant. The following assessments were performed: CADSS (pre-infusion, 10 min, 20 min, 40 min, 110 min after infusion start), HAMD-17 & MADRS (preinfusion, 110 min after infusion start), BPI (40 min and 110 min after infusion start), Vital signs (preinfusion, 10 min, 20 min, 40 min, 110 min after infusion), review of adverse events, Snaith Hamilton Pleasure Scale (20 and 110 min), Revised Social Anhedonia Scale (20 and 110 min). The post-ketamine infusion visit occurred the following day, with the following assessments: HAMD-17, MADRS, CADSS, C-SSRS, BPI, review of adverse events. The follow-up visit occurred post-discharge, approximately 1 week after infusion, with the following assessments: HAMD-17, MADRS, C-SSRS, BPI, review of adverse events. Identifiable clinical data was stored in a secure manner using Stanford Medical Box, and assessments were collected using REDCap.

27 participants were recruited for the overall study, 16 of whom were administered the eyepuff assay, and 7 of whom met behavioral inclusion criteria for neural analysis. These inclusion criteria for neural analysis were designed to mitigate the impact of variability unrelated to the phenomenon of study. We excluded from analysis in this work the 2 participants who received bolus infusions, because dosing and timing were not comparable to the other participants and we did not have data from sufficient participants to appropriately power separate analysis. Among the remaining 14 participants, inclusion criteria were met for participants exhibiting: 1) a significant change of late eye closure, normalized to early eye closure, between preinfusion and ketamine conditions, and 2) a significant change of late eye closure, normalized to early eye closure, between ketamine and post infusion conditions, in the direction corresponding to a recovery toward preinfusion. Based on our initial behavioral results (without neural recording), we expected to see a significant behavioral effect of ketamine, relative to preinfusion. Based on the kinetics of ketamine, we furthermore expected to see some degree of recovery at the post infusion timepoint, which is important to observe because it also indicates that any apparent reduction in response during ketamine is not simply due to habituation to the air puff stimulus. Lack of meeting the behavioral inclusion criteria appears to have arisen from a variety of potentially independent reasons, some of which were possibly due to the more complex clinical considerations of these participants and that were beyond our control. Apparent reasons for exclusion included: 1) Minimal effect of ketamine (e.g. SD163, SD178), potentially due to other onboard medications such as lamotrigine, a widely used antiepileptic drug known to reduce the effects of ketamine (63); 2) low preinfusion magnitude of late eye closure (e.g. SD170, SD184); 3) reduced reflexive response during ketamine (e.g. SD215); or 4) possible habituation of the participants to the eyepuffs across sessions, manifesting as a post infusion response less than the infusion response (e.g. SD207, SD242).

### iEEG recording and processing

#### Electrical recording.

Brain activity was recorded with intracranial electrodes as part of clinical routine practice, continuously (excluding occasional recording disruptions related to clinical care) throughout the entire multi-day hospital stay, including the Baseline and Ketamine Infusion sessions. Neural data was deidentified for subsequent analysis. Electrodes used were from AdTech Inc, had 12-16 platinum contacts, and were 0.86 mm in diameter, 2.29 mm in height, with 3-5 mm center-to-center inter-electrode distance. Data was recorded with the Nihon Kohden recording system, using the JE-120 amplifier for digitization and the LAN converter to write to the secondary data stream (64).

iEEG voltage traces were lowpass filtered for anti-aliasing (finite impulse response filter), then downsampled to 200 Hz. Voltage traces were notch filtered to remove 60 Hz electrical noise (second order infinite impulse response filter with quality factor of 20, applied forward and backward). Channels manually identified as bad, due to poor signal quality or artifacts identifiable in their spectrogram, or outside the brain were excluded. Channels were rereferenced with a bipolar montage.

#### Electrode localization.

We registered intracranial electrodes to specific anatomical regions using each subjects’ preimplantation T1-weighted MRI and postimplantation CT scans. Throughout this entire registration process, we leveraged the iELVis toolbox (65). From the MRI scan, we extracted both the subcortical segmentation and cortical surface (FreeSurfer v6.0.0 recon-all command (66). We subsequently aligned the preimplant MRI and postimplant CT scan (using bbregister from FreeSurfer (69) or flirt from Oxford Centre for Functional MRI of the Brain Software Library (67, 68) . Given that the electrodes were visible on the now T1-registered CT image, we marked these locations by hand using BioImage Suite (70). The anatomical labels assigned to each electrode were determined by a study-result blinded neuroanatomist and neurologist, Josef Parvizi, who based labelling upon individual-specific neural morphology and landmarks. Subsequently, the MATLAB script dykstraElecPjct (71) was used to map BioImage Suite-derived electrode coordinates into pial coordinates.

To generate the Yeo7 labels for each electrode we used inhouse MATLAB code that takes an input of FreeSurfer folder and pial electrode coordinates from BioImage Suite, and outputs the Yeo7 network labels for each coordinate in gray matter.

First, this code identifies the electrodes that are in gray matter. It uses the subfunction getPtdIndex_custom [modified from (72)]. Proximal Tissue Density is an index reflecting the density of neocortical gray and white matter surrounding a stereotactic electrode that has its centroid either in neocortical gray or white matter. Voxel labels are taken from the FreeSurfer aparc_aseg.mgz file. Then a 3x3 voxel cube is drawn around the centroid of the electrode, and the number of gray and white matter voxels in the cube are counted. If an electrode has 1 gray matter voxel in the cube, it is counted as a gray matter electrode.

Next, the Yeo7 (and Yeo17) network labels were generated using the subfunction elec2Parc_subf (modified from elec2parc, a part of the iELVis toolbox to skip gray matter depth electrodes which cannot be labeled in surface atlases). This code first loads the pial (.PIAL) coordinates of the electrodes. Then for each hemisphere, it reads the pial surface file (h.pial.T1). This file has the coordinates of each vertex of the surface. Next, it loads the cortical parcellation file that Freesurfer generates for Yeo7 (h_Yeo2011_7Networks_N1000.mat), this file has a Yeo7 label associated with each vertex from the pial surface file. Now for each gray matter surface electrode, it finds the closest vertex in the pial surface file and its associated Yeo7 label. Once it loops through all electrodes, all surface gray matter electrodes have a Yeo7 label. Software libraries used for plotting anatomy include Pysurfer and nilearn.

#### HCP cortical parcellation.

We performed a more specific anatomical localization of the cortical contacts by identifying the cortical areas that were implanted in using the Human Connectome Project (HCP) cortical parcellation (32). Since complete HCP-style imaging datasets were not available for these participants, we processed each participant’s T1w structural MRI image with the “LegacyStyleData” and “MSMSulc” options in the PreFreeSurferPipeline, FreeSurferPipeline, and Post FreeSurferPipeline steps of the HCP processing pipeline (www.humanconnectome.org/software/hcp-mr-pipelines). This registered each participant’s cortical surface to the HCP fsaverageLR32k average cortical surface template using MSMSulc, an algorithm warping the vertices of spherical cortical surface meshes based on features, in this case, cortical folding (sulcal depth) (73). Next, each participant’s cortical contacts were assigned to the nearest registered surface mesh vertices using wb_command in Connectome Workbench (www.humanconnectome.org/software/workbench-command). This resulted in a list of cortical contacts pooled across all participants with assignments to vertices in the HCP fsaverageLR32k cortical surface mesh. Contacts were visualized on the HCP cortical surface using spherical foci drawn at their vertices using wb_command in Connectome Workbench.

### iEEG analysis

#### ERP analysis.

A sync signal from the air puff device was used to identify the timing of each individual air puff, which also corresponded to illumination of a sync LED visible to the eyepuff cameras. Event related potentials were computed as the average local field potential (200 Hz downsampled, bipolar rereferenced, 60 Hz notch filtered).

#### Multitaper Spectrogram.

For time-frequency analysis of puff-related dynamics, a multitaper spectrogram was computed with time band-width of 2 (yielding use of 3 tapers), stride of 0.05 s, and window width of 0.4 s (74). The spectrogram for each condition (preinfusion, infusion, post infusion, etc.) was z-scored at each frequency, based on a 2 minute baseline period preceding the onset of the puff sequence. To further highlight puff-specific dynamics, spectrograms were further z-scored at each frequency for each puff, using a 1.5 s baseline preceding the onset of the puff.

#### Factorization.

Nonnegative matrix factorization (NMF) was used as a dimensionality reduction technique to identify shared spectrotemporal components across channels. The constraint of NMF that the factor coefficients are nonnegative often yields improved interpretability of the factors (as compared with other methods such as principal component analysis, in which the factors with positive and negative coefficients can cancel each other out and result in worse interpretability). NMF can also be viewed as a form of soft clustering. The puff-triggered z-scored spectrogram of each channel across all patients, from 0 to 1.4 s from puff onset, was averaged across a train-set consisting of half of the preinfusion and post infusion trials (every-other trial, the test-set consisted of the remaining trials), flattened, and then stacked into a [channels x time*frequency] array. Preinfusion and post infusion trials were used together during training in order to promote identification of components that were shared across these conditions which exhibited similar behavioral responses. Because the spectrograms were z-scored, they could have both positive and negative values. To account for this and convert the data into a format compatible with NMF, the spectrogram array was split into positive and negative components. In the positive array, all negative entries were set to zero. In the negative array, all positive entries were set to zero and the negative entries were represented by their absolute value. Each array (both are now nonnegative) was factored using non-negative matrix factorization into the product of two matrices W and H, minimizing the Frobenius norm. The number of factors was selected by plotting number of factors versus the reconstruction error on spectrograms computed from the test set of trials (the trials not in the train set). A clear “elbow” in the reconstruction error plot was identified, beyond which additional factors provide diminishing returns, at a total of six factors (three positive and three negative). The footprint (projection) of each factor was constructed from H. The loading of each channel onto a factor was extracted from W. Loadings represent the extent to which a factor is “active” in the neural response of that channel. For analysis of each factor, channels with an average loading on that factor of <0.1 (across the preinfusion and post infusion test trials) were excluded from subsequent analysis of that factor. To compare loadings across sessions (preinfusion, infusion, and post infusion), loadings were computed for each session based on test trials, using the factorization derived from pre- and post infusion training trials. Infusion loadings were compared with the average of preinfusion and post infusion loadings, using a mixed-effects linear model, grouped by patient, to identify significant changes in infusion loading. The anatomical distribution of each factor was computed by averaging the loadings of all channels within a region or Yeo7 resting state network. For visualization, loadings within a region were normalized across the six factors. Regional change in loading was similarly computed using the average change in loading (infusion minus preinfusion) for all channels in a region.

#### Permutation cluster test.

Within each patient, channels were aggregated by each labeled region, and the mean spectrogram across channels within a region was used as a regional spectrogram. Only channels where both inputs to the bipolar rereferencing were in the same region were used. For cross-patient analyses, the regional spectrograms were concatenated for each region, yielding a [trial x time x frequency] array with the number of trials equal to the sum of the number of trials across all patients in which that region was recorded, for each eyepuff session (preinfusion, infusion, post infusion). To identify changes in response during the infusion, a permutation clustered one-sample t-test [mne.stats.permutation_cluster_1samp_test from MNE-python (75)] was used on the paired difference between the infusion and preinfusion sessions, yielding a t-value for each time-frequency, and clusters identifying the presence of significant changes between sessions. Trials were paired across sessions by position in the puff sequence (the timing and sequence of puffs was identical across sessions), to account for potential consistent bias in response across the sequence of puffs. Relaxing this constraint yielded similar results. A cluster forming threshold of 0.01 was used. The lowest (most significant) *P*-value in each region was further corrected for multiple comparisons across regions, using Benjamini-Hochberg False Discovery Rate correction. Regions with a corrected lowest *P*-value < 0.01 were deemed to have a significant cluster. For regions with a significant cluster, all clusters with uncorrected p-value < 0.01 are displayed. To identify individual channels exhibiting significant changes, the same procedure was applied to individual channels. The robustness of these was assessed across different hyperparameters and settings, including cluster forming thresholds of 0.05, 0.01, and 0.001, a threshold free cluster enhancement with range [0, 0.1]. Results were additionally assessed when computing the t-value with either an independent t-test (as opposed to paired) or a mixed-effects linear model with trials grouped by participant.

#### Coding Dimension.

Bandpower over time for canonical frequency bands was computed from the local field potential by bandpass filtering and then computing the squared magnitude of the hilbert transform [scipy.signal.hilbert from SciPy (76)] of the bandpassed signal. A fifth-order butterworth filter was used (scipy.signal.butter, with scipy. signal.sosfiltfilt). Bandpower was z-scored within each session, across a 20-minute epoch beginning 10 minutes before the first eyepuff, and a window around each eyepuff was extracted. For each patient, using the preinfusion session, a coding dimension was computed based on the difference in the mean activity of each channel during the 0.5 s preceding each puff and either an early (0.05 s immediately after each puff) or late (0.5 to 1.5 s after each puff) time window, corresponding to the “fast” and “persistent” neural dimensions (fig. S34A). The coding dimension weight for a channel is mean(early or late) – mean (prepuff) / [sqrt(var(early or late) + var(prepuff)], where the mean and variance are across trials, and then normalized by the sum of the absolute value of weights across channels. The following frequency bands were investigated: delta (1 to 4 Hz), theta (4 to 8 Hz), alpha (8 to 12 Hz), beta (12 to 30 Hz), low gamma (30 to 50 Hz), high gamma (65 to 95 Hz), and broadband (unfiltered LFP). It was not obvious a priori which frequency band was most relevant to our cross-species comparison, so we computed the correlation of the mouse coding dimension trajectory with the preinfusion coding dimension trajectory for each human frequency band. We identified the beta bandpower as most similar and used that for cross-species analyses. Using the preinfusion coding dimension weights, the projection onto the coding dimension was computed at each timepoint of the ERP for each session. Within each session, the projection was z-scored within a window from 0.5 s before to 1.5 s after each puff onset, and then baseline subtracted, using a window from 0.5 to 0 s preceding puff onset. The z-scoring allows comparison of dynamics, while accounting for the possibility of a global shift in activity levels during different sessions.

#### Intrinsic timescale.

Using the preinfusion persistent neural dimension weights, the coding dimension time series was computed using beta bandpower (based on the reasoning in the *Coding Dimension* section), for an eight minute time period immediately preceding onset of the eyepuff assay in either the preinfusion or infusion conditions. The intrinsic timescale of this trace was extracted by first computing the autocorrelation of the entire signal (scipy.signal.correlate) and then fitting an exponential decay from the peak of the autocorrelation to 2.5 s to the right of the peak. An exponential of the form a*exp(-x/b) + c was fit using scipy.optimize.curve_fit, and an initialization of a=1, b=0.2, c=0. The fit value of b was the timescale.

#### Phase-locking Value.

To quantify the consistency in phase differences between channels, which might indicate the presence of inter-regional functional relationships, phase-locking value was computed between all pairs of recorded channels. The over-time version of phase-locking value (PLV) computed here, a variant of the cross-trial phase locking computed in (77), is referred to in some texts as “inter-site phase clustering over time” (78). For frequency bands of interest, the instantaneous angle was extracted from the cleaned voltage traces by band-pass filtering the signal (fifth-order butterworth filter, applied forward and backward with cascaded second-order sections, scipy.signal.butter, with scipy.signal.sosfiltfilt), then taking the angle of the filtered signal’s Hilbert transform (using `angle` and `Hilbert` functions from NumPy (79) and SciPy). Notably, applying the filter forward and backward prevents the distortion of phase. Subsequently, instantaneous angular difference between each pair of signals was computed. The phase-locking value, or consistency of phase difference, was then calculated for a particular time window by taking the magnitude of the circular mean of angular differences (80). To compute the changes in phase-locking value over time, we used sliding 5-second windows to compute phase locking value.

To understand changes in PLV during ketamine relative to preinfusion, each channel’s PLV values were z-scored to its respective preinfusion window. To compute network disruption by ketamine in specific subnetworks (such as between sites with high post-puff persistence), z-scored ketamine change in phase locking was averaged across all edges (electrode pairs) for which both sites were within the subnetwork (i.e. classified as high persistence when ranked by coding dimension magnitude). When classifying sites as having high or low persistence, the top and bottom tenth percentile of sites per patient were classified as high or low persistence, respectively, and white matter contacts were excluded on the basis of their lack of consistent post-puff activity. Preinfusion time windows of 25 minutes prior to the start of ketamine infusion were used, excluding periods of time in which the patient was undergoing the eyepuff assay or which exhibited strong epileptic or sleep-related activity. The peak ketamine time window was taken to be the final 25 minutes of the ketamine infusion, excluding periods of the eyepuff assay. For patients who received a single step of the targeted-controlled infusion dosage, the peak ketamine time window was taken to start at minute five, to account for the fact that the pharmacokinetics of the infusion paradigm (initial bolus, followed by continuous infusion) resulted in a peak blood level that occurred earlier than the peak blood level for patients receiving a continuous 40-minute infusion. Additional testing confirmed that network coupling results were not dependent on the exact time windows used. In cross-species analyses, based on the selection of the beta frequency band as detailed in *Coding Dimension*, human phase locking changes on ketamine were also computed using the beta frequency range (12 to 30 Hz).

To assess the impact of the specific technique used to compute phase-locking value, the results were also repeated using a Morlet wavelet-based approach to compute rolling phase-locking value, as implemented in the mne package by the `spectral_connectivity_time` in mode `cwt_morlet`. To assess the impact of the specific time window sizes used to compute rolling phase-locking values, the results were repeated using different window sizes, including 1 and 60 seconds, as well as adaptive window sizes which scaled window sizes across frequencies to ensure that 5 cycles were incorporated per window.

#### Band-limited oscillation detection.

Changes in spectral power can be a consequence of changes in aperiodic and periodic signal properties (81). Importantly, these different types of changes can have very different biophysical interpretations—for example, periodic, band-limited oscillations are indicative of synchronous activity in the recorded neural circuit (50), whereas aperiodic power changes may be indicative of general shifts in asynchronous, overall firing rates in the recorded circuit (82, 42). Given this important distinction, a computational pipeline was implemented to enable the detection of band-limited oscillations prior to, during, and after the ketamine infusion, with the additional function of identifying band-limited oscillations which were distinct (i.e. had a statistically significant increase magnitude or change in frequency localization) during ketamine. Power spectral densities during the three key time “windows” (preinfusion, ketamine infusion, post infusion) were computed from the multitaper spectral power outputs [time-half-bandwidth of 30, 59 tapers, window size of 30 sec, stride of 15 sec, (74)]. Window ranges were selected to avoid sleep and ictal activity, and the ketamine window was chosen to coincide with peak ketamine pharmacokinetic levels (which differed across patients based on infusion type, i.e. continuous, TCI, and bolus infusions). Each time window was subsequently split into multiple short (3 minute) time “segments.” Subsequently, the FOOOF (81) toolbox was used to subtract out the aperiodic component of the power spectra for each time segment, which fits the aperiodic component using an iterative approach to remove the locations of periodic phenomena prior to aperiodic estimation. The location and properties of peaks in the aperiodic-removed power spectrum were subsequently extracted for each time segment (scipy.signal.findpeaks). Even at baseline, identified peaks exhibit fluctuations due to electrical noise and natural variability (such as arousal). Thus, to determine band-limited oscillations which were consistently present across baseline (preinfusion and post infusion) and ketamine (during infusion) states, peaks identified in each short time segment were clustered across the time windows (based on 80% overlap of peak width in frequency space), where pre- and post-infusion windows were combined. Peak clusters which were observed in fewer than 40% of time segments were discarded. Subsequently, peak clusters present during ketamine were tested for whether they were distinct in frequency and/or magnitude from peaks present during baseline. If a ketamine peak cluster over-lapped in frequency (within 2 Hz) with a baseline peak cluster, a one-sided rank sum test (with alpha of 0.001) was performed on the respective oscillatory peak height distributions across time segments, to determine whether this was an oscillation whose amplitude was significantly amplified by ketamine. If a ketamine peak cluster was unique in frequency, this was determined to be an oscillation occurring at a new frequency during ketamine. In sum, this procedure led to the identification of the set of band-limited oscillations which were unique to the ketamine time window.

In order to test whether there was a trend toward more low frequency oscillations recorded in patients with stronger dissociation (CADSS score greater than or equal to 10), the observed fraction of total oscillating electrodes in high and low CADSS participants was first separately computed (i.e. numerator is number of electrodes with an oscillation detected, denominator is total number of electrodes recorded in high or low CADSS participants). Next, the difference in fraction of observed oscillating electrodes in high vs. low CADSS participants was computed—if this difference was greater than zero, it indicated a higher fraction of oscillating electrodes in high CADSS participants. Finally, this observed fractional difference was compared against a null distribution in which the per-participant high vs. low CADSS label was shuffled across participants without replacement, and the difference in fraction oscillating electrodes was recomputed each time.

#### Tractography structural connectivity.

Analyses were performed on diffusion MRI data from 200 participants of the Human Connectome Project (HCP) (83). The data were acquired with a 3T MRI scanner at an isotropic resolution of 1.25 mm. Three acquisitions with different gradient tables were obtained. Each table had approximately 90 diffusion directions at b=1000, 2000, and 3000 s/mm^2^ and 6 interspersed b=0 images. Data were obtained already preprocessed, including topup, eddy current, and gradient nonlinearity corrections and Bayesian Estimation of Diffusion Parameters Obtained using Sampling Techniques (BEDPOST X) (84, 85).

To obtain the seed masks for tractography, electrode locations were obtained from a CT scan co-registered to the MPRAGE (T1-weighted) MR image for patients SD168, SD172, SD181, SD185. Coordinates of the electrodes were obtained and used as the center to generate 2mm spheres. These electrode masks were transformed into MNI space. These and all other co-registration were performed using the Advanced Normalization Tools (ANTs) (86). The MNI space electrode masks were co-registered to each individual HCP participant. Additionally, thalamus masks from the Morel atlas (87) were co-registered to each individual HCP participant. Masks of the individual thalamic nuclei included in the atlas were also obtained. For exclusion purposes, a corpus callosum mask was obtained from the FreeSurfer (66) segmentation in native space of ten participants. The ten callosal masks were transformed to MNI space, averaged, resulting in a mask in MNI space, then inverse transformed to native space of all participants. All registrations were done using a concatenation of a rigid transformation followed by a non-linear deformation, first of the MNI space to the individual T1-weighted image included in the HCP dataset and then from the T1-weighted image to the b=0 image.

Probabilistic tractography was performed in FSL (88) using the probtrackx tool. 10000 streamlines were launched from each seed voxel (each electrode mask), with a 0.5 mm step size, a 75° curvature threshold and with distance correction and loop-check enabled. Tractography was performed from each electrode as seed, the whole thalamus as target, and the corpus callosum as an exclusion mask (to inhibit erroneous bilateral results). Tractography was performed in the native space of each participant, left and right hemispheres separately. All tracts corresponding to electrodes within the same non-thalamic region (e.g. ORB, PMC, etc.) were first averaged in each hemisphere of every participant. For each of these participant-level tracts, streamlines that intersected with each thalamic nucleus were counted. Streamline counts were normalized to the total number of streamlines intersecting the thalamus from a given non-thalamic seed region to obtain the proportion of streamlines in each thalamic nucleus for the given non-thalamic seed region. These proportions were then averaged across all the participants in the sample (n=200) and plotted with a heatmap representation on a matrix. For visualization purposes, the participant-level tracts were also transformed into MNI space and averaged.

### Eyepuff assay (mouse)

#### Eyepuff rig.

Following (15), head fixation posts and a plastic carrying tube were mounted on a magnetic base (Thorlabs KB3X3) to facilitate animal placement and ensure repeatable positioning. Two high speed cameras (Basler ace acA1300-200uc Monochrome USB 3.0) with 6 mm UC series fixed focal length machine vision lens (Edmund Optics #33-301) were mounted on each side of the mouse using 1/4-20 Mounting Adapter (Edmund Optics #88-517). Bonsai-rx was used for simultaneous acquisition from both cameras. Infrared lighting was positioned at multiple sides surrounding the mouse (Thorlabs LIU850A). Pressurized building air was regulated down to 30 PSI, controlled through an electronically controllable solenoid (NResearch 2-way NC isolation valve) and directed at the location of the mouse’s right eye through a pipette tip with the tip slightly cut off. Infrared sync LEDs were placed within the field of view of each camera. The solenoid and sync LEDs were controlled through an Arduino. Inter-trial timing and data acquisition were controlled with a python script.

Trials consisted of 8 puffs, each 250 ms long, with an interpuff interval of 3 s (including puff duration). A longer puff length was used here relative to the human assay because we observed a stronger behavioral effect with a longer air puff, important for maximizing clarity of the behavioral and neural investigations. Inter-trial intervals were of random duration over the range 45 to 90 s. For infusion experiments, 20 preinfusion trials were presented before infusion. After infusion, at least 20 more trials were administered.

For experiments without neural recording, eye closure was extracted as follows: 1) The right eye was tightly cropped out; 2) Frames in which the mouse had maximal eye closure were identified and averaged as template; 3) The frame at each timepoint was correlated with the template frame, yielding a measure of eye closure across time. Synchronization with the air puff was ensured using the sync LEDs. Each trial was baseline-subtracted based on the eye closure during 2 s preceding the trial. The eye closure was then scaled by 1/(1-baseline), where baseline corresponds to the pre-trial baseline eye closure averaged across the first two trials (when the eye should be fully open). For experiments with neural recording, in which the mice can be more stressed and there can be more variation in the eye closure across trials (including occasional Harderian secretion), DeepLabCut was used to precisely extract the eye size at each time point (using the same procedure as with the human data), with the same subsequent processing. The manual labeling and processing required for this procedure precluded it from application to the higher throughput behavioral experiments. We verified through manual inspection that both procedures accurately measured eye closure.

Software libraries used generally for analysis (of both mouse and human data) include Matplotlib (89), Pandas (90), Seaborn (91), and Scikit-learn (92).

#### Eyepuff assay with liquid reward available.

In an alternative behavioral assay, mice were trained to freely collect a liquid caloric reward (Vanilla Ensure) from a lick spout. A small amount of Ensure was dispensed onto the tip of a spout approximately every 4.75 s (min 4.5 s, average 4.75 s, max 5 s, with an exponential distribution of times between 4.5 s and 5 s). A small amount of Ensure was additionally delivered if mice licked the spout at any time, subject to a 0.5 s refractory period. Mice received two sequences of 8 eyepuffs (each 3 s apart) spaced by approximately 90 s. Licking was measured across the eyepuff sequence and lick probability per time bin was measured combining both trials within animal and averaging across animals (n = 6).

#### Drug infusion.

Mice were C57BL/6J (Jackson Laboratory #664), between 8 to 20 weeks old, and predominantly female. For behavioral experiments without neural recording, intraperitoneal (IP) infusion was used. After 20 preinfusion trials had completed, the mouse was removed from head restraint, manually infused IP with a 32G needle, and head restrained again, at which point the post infusion trials commenced. Only trials occurring at least 3 minutes after the time of infusion were considered post infusion. The head restraint device was designed such that the animal’s location was identical with each head restraint. In many cases, the same mice were used for experiments with different drugs, with at least one full day of separation between experiments. For the baseline saline, ketamine (50 mg/kg), and PCP (20 mg/kg) experiments of Fig. 1, the same five mice were used for all conditions, with at least 3 days of separation between any drug infusion, with the order for all mice as ketamine (50 mg/kg), then saline, then PCP (20 mg/kg).

For remote infusion during neural recording, a subcutaneous cannula was used. Ketamine (100 mg/kg) was infused after 20 preinfusion trials had completed. This higher dose was used to ensure robust drug effects, given the increased stress due to the implanted cannula and the prolonged head-fixation when preparing the neural recording. The cannula was implanted with a protocol designed to minimize discomfort, as follows: Mice were briefly anesthetized under 5% isoflurane. Topical lidocaine (4%) was applied to 2 mm^2^ of the dorsal surface, 1 cm posterior of the scruff. A flexible catheter (PTFE tubing, SUBL-160 0.016” OD x 0.010” ID, 40 cm long with an attached 27G blunted luer hub, product name MIC STD from Braintree Scientific) connected to 1 meter of 1.57 OD x 1.14 ID polyethylene tubing primed with ketamine solution (ketamine 5mg/mL in saline) was inserted subcutaneously along the back of the mouse, using a 20G needle and secured in place with cyanoacrylate glue. A small piece of extended-release lidocaine patch (4%) was placed atop the cannula insertion site. The mouse was then head restrained and the tubing was affixed out of reach from the mouse’s limbs. Ketamine could then be infused without touching the mouse, midway through neural recording sessions. This protocol was developed to minimize discomfort associated with the implanted cannula.

#### Muscimol inhibition.

Female C57BL/6 mice less than six months old were affixed with a metal head bar at least five days before the start of habituation and behavior. On the first day, the mice were first habituated on the air puff rig with ten trials of eight puffs of air each delivered. On the second day, baseline behavior was recorded, and then craniotomies were performed under isoflurane administration at −1.8 AP and ± 1.5 ML and subsequently covered with Kwik-Cast (World Precision Instruments). Mice were given 0.5 mg/kg Buprenorphine SR and allowed to recover for at least 3 days. For the saline injection control, mice were injected without anesthetic bilaterally with saline at ± 1.6 ML, −1.9 AP, −3.6 DV with 500 nl at 100 nl/min each side. After one hour, behavior was recorded on the air puff rig with twenty trials of eight air puffs each. Mice were then allowed two days to recover. For the muscimol inhibition behavior, a 1:3 mixture of 1 mg/mL fluorescently conjugated muscimol and 1 mg/mL muscimol was prepared. Mice were injected with this muscimol mixture without anesthetic bilaterally at ± 1.6 ML, −1.9 AP, −3.6 DV with 500 nl at 100 nl/min each side. After one hour, behavior was recorded on the air puff rig with twenty trials of eight air puffs each. The mice were then perfused for histological analysis.

### Neuropixels recording and processing

Electrophysiological recordings were conducted as described in (17), with 3 to 4 dye-coated Neuropixels 1.0 probes inserted acutely per recording session. TTL pulses for synchronizing behavioral trial events with neural data were recorded on a Nidaq (Texas Instruments). Electrophysiology data and Nidaq-recorded synchronization signals were acquired using SpikeGLX (B. Karsh), preprocessed with CatGT (B. Karsh), and spike sorted with Kilosort3 (93). Extracted spikes and behavioral events were aligned with T-Prime (B. Karsh). Preprocessing through T-Prime was orchestrated using a python script based on the Ecephys pipeline (https://github.com/jenniferColonell/ecephys_spike_sorting). Spike sorted units were quality curated using BombCell (https://doi.org/10.5281/zenodo.8172821), taking only well-isolated units assigned as “somatic.” Units were further required to have inter-spike interval violations of less than 0.1, signal-to-noise ratio of greater than 2, and greater than 500 spikes; units not meeting these criteria were excluded. Data was loaded and further preprocessed using the brainwide-npix python package (17, 94) (https://github.com/erichamc/brainwide-npix). Firing rates were binned at 10 ms resolution and smoothed with a causal moving average filter over 50 ms. For cross-puff and population analyses, we excluded one animal that exhibited notable stress response (Harderian gland secretion) during the pre-ketamine epoch.

#### Anatomical referencing.

Following recordings, mice were euthanized and brains were collected, chemically cleared, and imaged as previously described in Richman et al., 2023, with the exception that ethyl cinnamate (Sigma) was used during light sheet imaging, rather than dibenzyl ether. Anatomical registration and matching of unit location to template anatomy was performed as described in Richman et al., 2023. In brief, following imaging, brains were registered to the Allen Brain Institute Reference Atlas (CCFv3, https://allensdk.readthedocs.io/en/latest/); dye tracts from inserted probes were reconstructed using Napari (95) and reconstructed electrode tracts were mapped to anatomical locations using the brainwide-npix package. Regions are color coded following the CCFv3 regional colormap.

#### Comparison of rates between pre-infusion, ketamine infusion, and post infusion.

Experimental epochs were divided into pre-infusion, “Pre”; ketamine infusion, “Ketamine”; and post infusion, “Post “. These epochs were divided based on air puff trial number, with each trial consisting of a series of 8 air puffs. As ketamine was infused following the completion of the 20th trial, the Pre epoch was defined as the first 20 trials. To allow some time for ketamine onset and to capture the peak effects of ketamine following infusion, trials 22 to 32 were taken as the Ketamine epoch. For analyses of recovery following ketamine infusion, the Post epoch was defined as trials 34 to 40, which should be considered to capture the late influence of ketamine, given that neural effects of ketamine are still present, albeit reduced.

#### Example-session unsupervised clustering and spectral analysis.

Firing rates across example sessions (fig. S29) were downsampled by 5 times and reordered using Rastermap (96) with the following parameters: n_PCs, 128; n_clusters, 65; locality, 0.75; time_lag_window, 10; grid_upsample, 10. Spectral power density was computed over the given 1 minute windows using scipy.signal.welch using non-downsampled data and an nperseg parameter of 2048.

#### Regional correlation analysis.

Pairwise Pearson correlation coefficients were quantified between individual units during the entirety of the pre-infusion period and during a 10-minute period following the onset of ketamine infusion. For visualization (fig. S30), units were grouped by anatomical region assignment and further sorted by anatomical depth along the probe insertion trajectory.

#### Significant modulation.

Some analyses were restricted to neurons whose firing rates were significantly modulated by the air puff presentation. Significant modulation was defined by an uncorrected P-value of less than 0.01 in paired T-tests comparing average firing rates in any of the following windows: 1 s prepuff, 200 ms post puff; 1 s prepuff, 300 ms to 800 ms post puff; 1 s prepuff, 1 to 2 s post puff; 1 s before the first puff of a series, 1 s before the last puff of a series; 1 s following the first puff of a series, 1 s following the last puff of a series.

#### Unsupervised clustering of peri-puff firing rates.

Firing rates in a 3 s window (−1 s, 2 s) surrounding each air puff were tabulated from cells across all recordings. Cells with no significant modulation by the air puff or trial structure were excluded from analysis. Firing rates were averaged per-cell across all pre-infusion puff trials and individual puffs to yield a single peri-puff timeseries per cell. Peri-puff timeseries were baseline subtracted using the 1 s average pre-puff activity of each neuron. Clusters were obtained with agglomerative clustering using Scanpy (97): principle components were computed using solver = “arpack”; a neighborhood graph was computed using n_neighbors = 30 and n_pcs = 20; a uniform manifold approximation and projection (UMAP) embedding was computed (98); and clusters were assigned using leiden clustering on the UMAP embedding (99). Average firing rates for each cell were z-scored across the peri-puff time window before visualization (Fig. 4, D and E).

#### Cumulative fraction of cells in cluster by region.

For each cluster, the fraction of cells in that cluster in a given region was calculated. For visualization (fig. S31A), cluster fractions were stacked vertically, with clusters ordered and colored by the time for average (across all cells in all regions assigned to a given cluster) per-puff firing rate activity to decay to 25% of its peak. Regions were ordered by sorting according to the regional decay time, calculated as a weighted average of per-cluster decay times, with weights corresponding to the fraction of cells in a given cluster in the given region.

#### Regional fraction of cells per cluster.

For each cluster, the fraction of cells in that cluster out of the total significantly modulated cells (see above) in a given region was tabulated. P-values corresponding to the fraction of cells observed in a given cluster in a given region were obtained by comparison to a null hypothesis that assumed cluster labels of cells in each region were drawn from a categorical distribution with equally probable clusters. Resulting P-values were corrected for multiple comparisons across regions using Benjamini-Hochberg False Discovery Rate correction. Labels were shown for regions with a corrected P-value of less than 0.05.

#### Analysis of similarity between trial average responses across ketamine trials.

Peri-puff firing rates were averaged across all pre-ketamine trials for neurons in each brain region to create reference regional peri-puff firing rates. Cells with no significant modulation were excluded from the analysis. For every trial following administration of ketamine, cosine distance (scipy.spatial.distance.cosine) was calculated between the given trial and the reference (fig. S31, D and E).

#### Regional rise and decay time.

Neuron firing rates were pooled per region across recording sessions and filtered according to the selectivity criteria described above. Regions were filtered to those having greater than or equal to 30 significantly modulated neurons across all recordings. Firing rates were averaged within unsupervised cluster assignment (as described above) within a given region, for the first two puffs of each puff-series. The first two puffs per series were used (rather than all puffs) in order to maximize firing rate changes from baseline. Regional cluster puff-averaged firing rates were baseline subtracted by removing the average firing rate activity in the 1 s before puff onset. These baseline-subtracted regional cluster average peri-puff firing rates were converted to d’ time series by comparing the mean across puffs of each time bin post-puff to the mean across puffs of time bins at baseline, and this difference of means was normalized by the combined standard deviation of the baseline rates and post-puff rates in a given time bin. Time to rise was defined as the time it took a regional d’ trace to rise to 25% of its post-puff-onset peak. Time to decay was defined as the time it took to decay to 25% from its post -puff-onset peak. Regions with d’ peak values less than 1.3 were excluded from analysis to avoid miscalling peaks due to noise. Regions with peaks occurring greater than 750 ms following puff onset were removed from analysis to avoid spurious peaks. Cluster decay or rise values were combined per region using a weighted average, with weights determined for each cluster by the fraction of cells in a given region assigned to that cluster. Confidence intervals (CIs) were bootstrapped by repeatedly sampling with replacement from the set of puff replicates in a given region. Regional rise and decay times were visualized by sorting regions by their time to decay (Fig. 4, H and I).

#### Emotion-like neural dimension.

Simultaneously recorded neurons were analyzed for their firing rate activity along a neural dimension (“emotion-like neural dimension”) separating activity following the puff series from activity immediately before the puff series. This neural dimension was constructed by taking the mean difference between the 1 s window starting 5 s after the last puff onset of each preinfusion puff-series and a 750 ms window ending 250 ms before the onset of the first puff of each preinfusion puff-series and normalizing the per-neuron mean differences by the per-neuron variance in both time windows. The resulting vector of per-neuron weights was normalized to a unit vector. Only puff-modulated neurons were included in the analysis. To avoid contamination of the emotion-like neural dimension with neurons merely reflecting eye closure, a second dimension was constructed in a similar manner from the saturation phase of the puffseries (last 4 trials), comparing firing rate differences in the 200 ms following each puff onset from the 200 ms just prior to puff onset. The final emotion-like neural dimension was subsequently obtained by orthogonalization of the original affective neural dimension with this fast per-puff response dimension via QR decomposition. Neural activity along the emotion-like dimension was measured by linearly projecting simultaneously recorded activity in each time bin onto the emotion-like neural dimension. For visualization of neural activity along the emotion-like neural dimension during Pre or Ketamine puff series trials (Fig. 5D), activity for each recording session was projected onto that session’s emotion-like neural dimension and baseline subtracted per-trial by average activity in the 250 ms before the first puff of each series. Projections were smoothed for visualization by a 5th order butterworth filter (scipy.signal.butter, with scipy.signal.sosfiltfilt), with a filter width of 200 ms. Projections onto the training time windows used for constructing the emotion-like neural dimension were omitted from visualization (discontinuity in Fig. 5D). When analyzing regionally-restricted activity along an emotion-like dimension (fig. S32C), neurons were filtered by session to only those that were simultaneously recorded in a given region.

#### Cross-puff peri-stimulus timing histogram.

Neurons were sorted by their weights along the emotion-like dimension, from low to high. Session z-scored firing rates were averaged across trials of 8-puff sequences and further individually z-scored across the visualized epoch for ease of visualization.

#### Coding dimension, mouse peri-puff.

Simultaneously recorded neurons were analyzed for their firing rate activity along a neural dimension (“fast neural dimension”, fig. S34A and Fig. 6F; “persistent neural dimension”, fig. S34A and Fig. 6, D, H, and J) separating activity following each puff from activity immediately before the puff. This neural dimension was constructed by taking the mean difference between a target window (see below) and a reference window (average activity in the 1s before each puff) and normalizing the per-neuron mean differences by the per-neuron variance in both time windows. The resulting vector of per-neuron weights was normalized to a unit vector. Only puff-modulated neurons were included in the analysis.

For the fast neural dimension analyses, the target window used for the coding dimension was 0 to 70ms following puff onset. This window was selected to capture fast rise time dynamics. 70ms is the approximate (on average, across all regions) peak time following puff onset. For quantification of activity in this fast neural dimension (Fig. 6F), the same 0 to 70ms window was used.

For persistent neural dimension analyses (Fig. 6, D, H, J), the target used for the coding dimension was 0.5 to 1s following puff onset. This window was chosen to capture decaying puff-related signals across most regions while avoiding rise-related dynamics (see Fig. 4, H and I). Activity along this dimension was compared in the 1 to 2s following puff onset. This quantification window was chosen to be as late as possible, in order to maximally capture differences in persistent activity.

#### Coding dimension, mouse peri-tone.

Tone-related coding dimension analysis for simultaneously recorded neurons was performed as described above, except using windowed activity surrounding tone series presentation (16 kHz, 250ms on, 2750ms off, repeated 8 times). For fast neural dimension analyses (fig. S34C), 0 to 70ms following tone onset was used to construct and quantify activity in the coding dimension, matching what was used for the puff. For persistent neural dimension analyses (fig. S34, D to F), windows closer to the tone onset were used compared to those used for the puff, since tone-related neural activity decayed more rapidly than that induced by puffs (based on examination of average neural activity post tone onset, data not shown). We thus chose a window to construct the coding dimension that captured decaying activity while avoiding rise activity (150 to 350ms post tone onset) and quantified average neural activity in a subsequent 350 to 500ms window following tone onset (choosing this later window to end when activity along the persistent dimension during the ketamine condition returned to baseline).

#### Comparison of eyepuff and tone projections.

Projections along the persistent coding dimension for the eyepuff and the tone were transformed to detectability values (d’) as a form of normalization using the 1s pre-stimulus neural activity as baseline. This transformation yielded a per-session measurement of detectability for each signal.

#### Intrinsic timescale analysis, mouse.

Firing rates in the 30s before each stimulus (puff, Fig. 6H, or tone, fig. S34E) series were projected onto the persistent neural dimension to obtain activity time series along the persistent neural dimension at baseline. The intrinsic timescale of these time series were extracted by first computing the autocorrelation of the entire signal (scipy.signal.correlate) and then fitting an exponential decay from the peak of the autocorrelation to 2.5 s to the right of the peak. An exponential of the form a*exp(-x/b) + c was fit using scipy.optimize.curve_fit, and an initialization of a=1, b=0.2, c=0. The fit value of b was the timescale. The extracted baseline timescales were averaged across puff-series trials for each condition (pre-infusion, ketamine) within recording sessions and examined for paired changes across conditions.

#### Baseline population coupling, mouse.

Pairwise Pearson correlation coefficients were computed between all simultaneously recorded neurons in a session, using time windows 30s prior to the start of each stimulus (puff, Fig. 6J, tone, fig. S34F) series. Pairwise correlations were then filtered to only those of edges between neurons in the top 90th percentile of absolute value persistent dimension weight and averaged within recording session per condition (preinfusion, ketamine). Correlations values were visualized with normalization to preinfusion, thus indicating the change of the scale of coupling between these neurons across conditions.

### Theoretical model

#### Model description.

We modeled affective state dynamics and affective behavior using a biphasic first order system (Fig. 5F). System dynamics were determined by parameters *S*, τ_PERSIST_, and τ_BROADCAST_ as per the equation given in Fig. 5F. Puff sequences were simulated as a sequence of eight step input rises (for 250 ms) followed by decays (for 2750 ms), followed by a decay for 50 s. During periods of positive, non-zero input, the system was modeled using the broadcast dynamics x(t) = (S–x_0_)^+^(1-exp(-t/τ_BROADCAST_)) + x_0_. During periods with no input, the system was modeled with persistence dynamics x(t) = x_0_exp(-t/τ_PERSIST_). In these expressions, x_0_ is the value of x(t) immediately preceding the onset of each broadcast or persistence phase.

#### Model fitting, neural data.

The model was fit with least squares optimization (scipy.optimize.curve_fit) and the per-parameter boundary constraints [0,100]. The model was fit to the average across all recording sessions of the affective neural dimension activity during the puff series and subsequent decay, excluding the time periods used for constructing the affective state coding dimension (Fig. 5G). Pre-infusion and ketamine neural data was concatenated for use in parameter fitting. During fitting, this concatenated neural data was compared to concatenated model output for pre-infusion and ketamine conditions, in which parameters *S* and τ_BROADCAST_ were constant across conditions while the parameter τ_PERSIST_ was allowed to vary across conditions. For alternative models, *S* or τ_BROADCAST_ were allowed to vary across conditions while τ_PERSIST_ was held constant across conditions.

#### Model fitting, behavioral data.

The model was fit using the same procedure as with the neural data, but applied to behavioral eye closure data (Fig. 5, H and I). For each condition (different drug doses), eye closure data was averaged across participants and concatenated together, with 750 ms windows immediately following each air puff removed to avoid fitting the model to the reflexive component of the eye closure behavior. During fitting, model parameters *S* and τ_BROADCAST_ were kept constant across conditions while the parameter τ_PERSIST_ was allowed to vary across conditions.

#### Model variance explained.

Variance explained was quantified as the variance of the neural or behavioral data explained by the free parameter allowed to vary across conditions. This was computed as 1 - (MSEmodel / MSEnull). MSEmodel was computed as the mean squared difference between the experimental data and the model prediction. MSEnull was computed as the mean squared difference between the experimental data and the prediction of a null model in which no parameter was allowed to vary across conditions.

## Data and materials availability:

Study summary and participant data are deposited at (100). For human electrophysiology and behavior analyses and mouse behavior analyses, code can be found at (101) and data can be found at (102). For mouse electrophysiology analyses, code can be found at (103) and data can be found at (104).

## Supplementary Material

Supplementary Material

MDAR Reproducibility Checklist

science.org/doi/10.1126/science.adt3971

Supplemental Note 1; Figs. S1 to S38; Tables S1 to S3; References (60–104); MDAR Reproducibility Checklist
