# Supplementary material for: Conserved brain-wide emergence of emotional response from sensory experience in humans and mice
Source: Science. Author manuscript; Available in PMC 2025 Jul 23. (PMC12286656; doi:10.1126/science.adt3971)
Supplement: Supplementary Material [file NIHMS2090574-supplement-Supplementary_Material.pdf]

Submitted Manuscript: Confidential
Template revised November 2023
Science
AAAS

Supplementary Materials for

Conserved brain-wide emergence of emotional response from sensory experience in humans and mice

Isaac Kauvar[1,2,3]†, Ethan B. Richman[1,2]†, Tony X. Liu[1,2]†, Chelsea Li[4], Sam Vesuna[1,5], Adelaida Chibukhchyan[1,2], Lisa Yamada[1,2,6], Adam Fogarty[1,2,7], Ethan Solomon[1,5], Eun Young Choi[1,6], Leili Mortazavi[8], Gustavo Chau Loo Kung[2,9], Pavithra Mukunda[1,5], Cephra Raja[2], Dariana Gil-Hernández[1,5], Kishandra Patron[1,2], Xue Zhang[5], Jacob Brawer[5], Shenandoah Wrobel[2], Zoe Lusk[7], Dian Lyu[7], Anish Mitra[1,5], Laura Hack[5,10], Liqun Luo[11,12], Logan Grosenick[13], Peter van Roessel[1,5], Leanne M. Williams[5,10], Boris D. Heifets[1,14], Jaimie M. Henderson[6], Jennifer A. McNab[9], Carolyn I. Rodríguez [1,5,10]*, Vivek Buch[1,6]*, Paul Nuyujukian[1,2,6,15]*, and Karl Deisseroth[1,2,5,12]*

[1]Human Neural Circuitry (HNC) program, Stanford University, Stanford, CA, USA.
[2]Department of Bioengineering, Stanford University, Stanford, CA, USA.
[3]Graduate School of Education, Stanford University, Stanford, CA, USA.
[4]Neurosciences Graduate Program, Stanford University, Stanford, CA, USA.
[5]Department of Psychiatry and Behavioral Sciences, Stanford University, Stanford, CA USA.
[6]Department of Neurosurgery, Stanford University, Stanford, CA, USA.
[7]Department of Neurology and Neurological Sciences, Stanford, CA, USA.
[8]Department of Psychology, Stanford University, Stanford, CA, USA.
[9]Department of Radiology, Stanford University, Stanford, CA, USA.
[10]Veterans Affairs Palo Alto Health Care System, Palo Alto, CA, USA.
[11]Department of Biology, Stanford University, Stanford, CA, USA.
[12]Howard Hughes Medical Institute, Stanford University, Stanford, CA, USA.
[13]Department of Psychiatry, Weill Cornell Medicine, New York, NY, USA.
[14]Department of Anesthesiology, Perioperative and Pain Medicine, Stanford University, Stanford, CA, USA.
[15]Department of Electrical Engineering, Stanford University, Stanford, CA, USA.

*Corresponding authors. Email: deissero@stanford.edu (K.D.); vpbuch@stanford.edu (V.B.); cr2163@stanford.edu (C.I.R.); 23sciencemag@pn.stanford.edu (P.N.)
†These authors contributed equally to this work.

The PDF file includes:

Supplemental Note 1
Figs. S1 to S38
Tables S1 to S3
References (60-104)

Supplemental Note 1

In the following supplemental note, we discuss correspondences between human and mouse brain regions that are described in the study. Notably, the study's primary claims regarding the nature of neural dynamics underlying emotional responses do not rest upon direct anatomical mappings between species. For example, the computational technique of extracting different coding dimensions is agnostic to the exact anatomical distribution of relevant neural dynamics. This neural activity dimension approach allows for conclusions to be made about neural dynamics which are present at the population-level across recorded brain regions in both species, without necessarily relying on the presence of a particular recorded brain region.

Cross-species correspondence of eyepuff assay
The core features of the eyepuff task are conserved between mouse and human. However, we also note the many ways in which the experience of human participants in the task may be quite different from the experience of mice. For example, humans certainly have conscious awareness of task context, and subjective experience of the task itself, whereas these aspects are less clear in mice. These potential distinctions underscore the value of developing a cross-species translational approach to allow direct assessment of the human experience while also leveraging the powerful tools available with mice. We do not intend to claim that the mouse experience is identical to the human experience, but rather that it has many similar features, especially on the fast timescales surrounding individual puffs, which suggest that some (if not all) aspects are conserved. Using mice allows us to investigate additional details that we cannot access in humans, but the combination with measurements from humans is complementary and allows us to further investigate the neural basis of the human experience.

Cross-species correspondence of specific brain regions
We focus below on aspects of cross-species conservation among specific brain regions, to elaborate upon certain of the regions where eyepuff-driven emotional responses may emerge (see regions with significant changes in Fig. 3C). We note that there are of course substantial differences between mouse and human brain anatomy and function, including the lissencephalic nature of the mouse brain, the differing scale of brain sizes, and the absence of circuits dedicated to language use. Especially in non-sensorimotor cortical regions (including but not limited to orbital frontal cortex, cingulate cortex), the mappings between rodent and human brain regions are unlikely to be one-to-one.

- **Insula**: This region has been shown to be involved in interoception across humans and mice, and in the assignment of valence to internal and external, current and future stimuli (Gogolla 2017, Craig et al. 2011). Across species, insular cortex is comprised cytoarchitecturally of granular, dysgranular and agranular subdivisions, and receives functional input from sensory, interoceptive, and limbic regions (Gehrlach et al. 2020). Relevant to our study's focus on the emergence of affect from sensation, humans and rodents with insula lesions have been shown to sometimes have "pain asymbolia," in which participants are aware of pain, but do not seem to attribute negative emotional valence to the experience (Berthier et al. 1988). Denoted "INS" in human Fig 3; "AIv" in mouse Fig. 4.
  References:
  N. Gogolla, The insular cortex. Curr. Biol. 27, R580–R586 (2017).
  A. D. B. Craig, Significance of the insula for the evolution of human awareness of feelings from the body. Ann. N. Y. Acad. Sci. 1225, 72–82 (2011).
  D. A. Gehrlach, C. Weiand, T. N. Gaitanos, E. Cho, A. S. Klein, A. A. Hennrich, K.-K. Conzelmann, N. Gogolla, A whole-brain connectivity map of mouse insular cortex. Elife 9 (2020).
  M. Berthier, S. Starkstein, R. Leiguarda, Asymbolia for pain: a sensory-limbic disconnection syndrome. Ann. Neurol. 24, 41–49 (1988).

- **Orbitofrontal cortex**: Studies have found the relevance of this region to reward valuation, emotional processing, and decision making in both humans and mice (Wallis 2011, Rudebeck et al. 2022). For example, causal experiments in mice and humans have shown the importance of orbital frontal cortex to reward devaluation tasks, in which participants must make choices based on the perceived value of choice outcomes (Lichtenberg et al. 2021, Howard et al. 2020). In humans, alterations in orbitofrontal cortex activation and connectivity have been linked to altered emotional processing, depression and other psychiatric conditions (Rolls et al. 2020). Notably, orbitofrontal cortex is among the supramodal (non-sensorimotor) regions likely present in early mammalian

ancestors of both mice and humans (Kaas 2011, Beauchamp et al. 2022). Denoted “ORB” in human Fig 3; “ORBm”, “ORBl”, “ORBvl” in mouse Fig 4.
References:
J. D. Wallis, Cross-species studies of orbitofrontal cortex and value-based decision-making. Nat. Neurosci. 15, 13–19 (2011).
P. H. Rudebeck, A. Izquierdo, Foraging with the frontal cortex: A cross-species evaluation of reward-guided behavior. Neuropsychopharmacology 47, 134–146 (2022).
N. T. Lichtenberg, L. Sepe-Forrest, Z. T. Pennington, A. C. Lamparelli, V. Y. Greenfield, K. M. Wassum, The medial orbitofrontal cortex-basolateral amygdala circuit regulates the influence of reward cues on adaptive behavior and choice. J. Neurosci. 41, 7267–7277 (2021).
J. D. Howard, R. Reynolds, D. E. Smith, J. L. Voss, G. Schoenbaum, T. Kahnt, Targeted stimulation of human orbitofrontal networks disrupts outcome-guided behavior. Curr. Biol. 30, 490-498.e4 (2020).
E. T. Rolls, W. Cheng, J. Feng, The orbitofrontal cortex: reward, emotion and depression. Brain Commun. 2, fcaa196 (2020).
J. H. Kaas, Neocortex in early mammals and its subsequent variations. Ann. N. Y. Acad. Sci. 1225, 28–36 (2011).
A. Beauchamp, Y. Yee, B. C. Darwin, A. Raznahan, R. B. Mars, J. P. Lerch, Whole-brain comparison of rodent and human brains using spatial transcriptomics. Elife 11 (2022).

- **Cingulate cortex**: In the latest consensus definitions, human cingulate cortex can be roughly subdivided into anterior cingulate cortex (also in literature as ventral ACC), mid-cingulate cortex (also in literature as dorsal ACC), and posterior cingulate cortex (van Heukelum 2020). Across mice and humans, anterior cingulate cortex seems to be chiefly involved in processing affective and autonomic signals, exhibiting stronger structural connectivity to autonomic brainstem regions, amygdala, and frontal cortex (Etkin 2011, van Hout 2024). In contrast, the more caudal mid-cingulate cortex seems to be involved in aspects of cognitive control, and shows stronger structural connectivity to regions like the parietal and motor cortex (van Heukelum 2020). Posterior cingulate cortex and retrosplenial cortex have been shown to be involved in self-referential thought (Alexander 2023). Here, we adopt the updated ACC and MCC rostral-caudal sub-division nomenclature in both mouse and human, which has been shown to allow for more effective functional and anatomical homologies compared to the mouse subdivisions of Cg1, Cg2, infralimbic, and prelimbic cortices. Notably, cingulate cortex is among the supramodal (non-sensorimotor) likely present in early mammalian ancestors of both mice and humans (Kaas 2011, Beauchamp 2022). Anterior cingulate**:** “ACC” in human Fig. 3; anterior subdivisions of “ACAd”, “ACAv” [area A24], “ILA” (infralimbic), “PL” (prelimbic) in mouse Fig 4. Midcingulate**:** “MCC” in human Fig. 3; posterior subdivisions of “ACAd”, “ACAv” [area A24’]. Posterior cingulate**:** “PMC” in human Fig. 3; “RSPv” in mouse Fig. 4.
References:
S. van Heukelum, R. B. Mars, M. Guthrie, J. K. Buitelaar, C. F. Beckmann, P. H. E. Tiesinga, B. A. Vogt, J. C. Glennon, M. N. Havenith, Where is Cingulate Cortex? A Cross-Species View. Trends Neurosci. 43, 285–299 (2020).
A. Etkin, T. Egner, R. Kalisch, Emotional processing in anterior cingulate and medial prefrontal cortex. Trends Cogn. Sci. 15, 85–93 (2011).
A. T. B. van Hout, S. van Heukelum, M. F. S. Rushworth, J. Grandjean, R. B. Mars, Comparing mouse and human cingulate cortex organization using functional connectivity. Brain Struct. Funct. 229, 1913–1925 (2024).
A. S. Alexander, R. Place, M. J. Starrett, E. R. Chrastil, D. A. Nitz, Rethinking retrosplenial cortex: Perspectives and predictions. Neuron 111, 150–175 (2023).
J. H. Kaas, Neocortex in early mammals and its subsequent variations. Ann. N. Y. Acad. Sci. 1225, 28–36 (2011).
A. Beauchamp, Y. Yee, B. C. Darwin, A. Raznahan, R. B. Mars, J. P. Lerch, Whole-brain comparison of rodent and human brains using spatial transcriptomics. Elife 11 (2022).

- **Supramarginal gyrus**: In humans, this sub-region of the posterior parietal cortex has been shown to be involved in functions as diverse as complex tool use, verbal working memory, and language processing (Oberhuber et al. 2016, Deschamps et al. 2014, Wandelt et al. 2022). In mice, the posterior parietal cortex has been shown to be involved in navigation, decision-making, and sensory processing (Lyamzin et al. 2019). Denoted “SMG” in human Fig. 3; not recorded from in mouse.
References:

M. Oberhuber, T. M. H. Hope, M. L. Seghier, O. Parker Jones, S. Prejawa, D. W. Green, C. J. Price, Four functionally distinct regions in the left supramarginal gyrus support word processing. Cereb. Cortex 26, 4212–4226 (2016).
I. Deschamps, S. R. Baum, V. L. Gracco, On the role of the supramarginal gyrus in phonological processing and verbal working memory: evidence from rTMS studies. Neuropsychologia 53, 39–46 (2014).
S. K. Wandelt, S. Kellis, D. A. Bjånes, K. Pejsa, B. Lee, C. Liu, R. A. Andersen, Decoding grasp and speech signals from the cortical grasp circuit in a tetraplegic human. Neuron 110, 1777-1787.e3 (2022).
D. Lyamzin, A. Benucci, The mouse posterior parietal cortex: Anatomy and functions. Neurosci. Res. 140, 14–22 (2019).

- **Motor cortex:** Primary motor cortex demonstrates functional conservation across species, and is essential for the precise control of movement – including for the orbicularis oculi muscles essential for motor control of the eyelid in humans and mice (Paradiso et al. 2005, Pronichev et al. 1998). Notably, mice have large primary motor cortices, dominating much of their frontal cortex, whereas the human motor cortex occupies a smaller fraction of relative area, located anterior to the central sulcus (Ebbesen et al. 2017). Relative to all other mouse and human cortical regions, sensorimotor cortex exhibits the highest overall correlation in the expression profile of homologous genes between species (Beauchamp et al. 2022). This is reflected in the high levels of conservation of primary motor cortex cell type hierarchies across humans and mice – with notable differences, including the fact that the ratio of glutamatergic to GABAergic neurons in humans and mice are 2:1 and 5:1, respectively (Bakken et al. 2021). Denoted "MOT" in human Fig. 3; "MOp" and "MOs" in mouse Fig. 4.
References:
A. Beauchamp, Y. Yee, B. C. Darwin, A. Raznahan, R. B. Mars, J. P. Lerch, Whole-brain comparison of rodent and human brains using spatial transcriptomics. Elife 11 (2022).
G. O. Paradiso, D. I. Cunic, C. A. Gunraj, R. Chen, Representation of facial muscles in human motor cortex. J. Physiol. 567, 323–336 (2005).
I. V. Pronichev, D. N. Lenkov, Functional mapping of the motor cortex of the white mouse by a microstimulation method. Neurosci. Behav. Physiol. 28, 80–85 (1998).
C. L. Ebbesen, M. Brecht, Motor cortex - to act or not to act? Nat. Rev. Neurosci. 18, 694–705 (2017).
T. E. Bakken, N. L. Jorstad, Q. Hu, B. B. Lake, W. Tian, B. E. Kalmbach, M. Crow, R. D. Hodge, F. M. Krienen, S. A. Sorensen, J. Eggermont, Z. Yao, B. D. Aevermann, A. I. Aldridge, A. Bartlett, D. Bertagnolli, T. Casper, R. G. Castanon, K. Crichton, T. L. Daigle, R. Dalley, N. Dee, N. Dembrow, D. Diep, S.-L. Ding, W. Dong, R. Fang, S. Fischer, M. Goldman, J. Goldy, L. T. Graybuck, B. R. Herb, X. Hou, J. Kancherla, M. Kroll, K. Lathia, B. van Lew, Y. E. Li, C. S. Liu, H. Liu, J. D. Lucero, A. Mahurkar, D. McMillen, J. A. Miller, M. Moussa, J. R. Nery, P. R. Nicovich, S.-Y. Niu, J. Orvis, J. K. Osteen, S. Owen, C. R. Palmer, T. Pham, N. Plongthongkum, O. Poirion, N. M. Reed, C. Rimorin, A. Rivkin, W. J. Romanow, A. E. Sedeño-Cortés, K. Siletti, S. Somasundaram, J. Sulc, M. Tieu, A. Torkelson, H. Tung, X. Wang, F. Xie, A. M. Yanny, R. Zhang, S. A. Ament, M. M. Behrens, H. C. Bravo, J. Chun, A. Dobin, J. Gillis, R. Hertzano, P. R. Hof, T. Höllt, G. D. Horwitz, C. D. Keene, P. V. Kharchenko, A. L. Ko, B. P. Lelieveldt, C. Luo, E. A. Mukamel, A. Pinto-Duarte, S. Preissl, A. Regev, B. Ren, R. H. Scheuermann, K. Smith, W. J. Spain, O. R. White, C. Koch, M. Hawrylycz, B. Tasic, E. Z. Macosko, S. A. McCarroll, J. T. Ting, H. Zeng, K. Zhang, G. Feng, J. R. Ecker, S. Linnarsson, E. S. Lein, Comparative cellular analysis of motor cortex in human, marmoset and mouse. Nature 598, 111–119 (2021).

- **Hippocampus**: This region plays a conserved role in memory function across species, exhibiting structural and functional homologies (Manns et al. 2006). Pertaining to alterations in hippocampal eyepuff responses, hippocampal activity has been shown to be modulated by the emotional valence of stimuli in mice and humans (Meyer et al. 2019, Qasim et al. 2023). Concordant with the expansion of higher order association cortices in primates, the human hippocampus appears more functionally coupled (via fMRI correlations) to association cortices, whereas the rodent hippocampus appears more directly coupled to sensory cortices (Bergmann et al. 2016). Denoted "HIPP" in human Fig. 3; "DG-mo", "CA", "CA3" in mouse Fig. 4.
References:
J. R. Manns, H. Eichenbaum, Evolution of declarative memory. Hippocampus 16, 795–808 (2006).
H. C. Meyer, P. Odriozola, E. M. Cohodes, J. D. Mandell, A. Li, R. Yang, B. S. Hall, J. T. Haberman, S. J. Zacharek, C. Liston, F. S. Lee, D. G. Gee, Ventral hippocampus interacts with prelimbic cortex during inhibition

of threat response via learned safety in both mice and humans. Proc. Natl. Acad. Sci. U. S. A. 116, 26970–26979 (2019).
S. E. Qasim, U. R. Mohan, J. M. Stein, J. Jacobs, Neuronal activity in the human amygdala and hippocampus enhances emotional memory encoding. Nat. Hum. Behav. 7, 754–764 (2023).
E. Bergmann, G. Zur, G. Bershadsky, I. Kahn, The organization of mouse and human Cortico-hippocampal networks estimated by intrinsic functional connectivity. Cereb. Cortex 26, 4497–4512 (2016).

- **Basal ganglia**: These are a cluster of subcortical nuclei involved in motor control and reward learning, conserved in many respects across humans and mice (Cox et al. 2019, Hardman et al. 2002). Based on transcriptomic homologies, mouse and human striatal regions show strong conservation, with mappings between mouse caudoputamen and human caudate and putamen (Beauchamp et al. 2022). Based on connectomic homologies from functional MRI, mice seem to lack certain subdivisions of caudate most strongly connected to frontal cortex (Balsters et al. 2020). Denoted "BG" in human Fig 3; "CP" (caudoputamen), "ACB" (nucleus accumbens), "LSr" (lateral septal nucleus) in mouse Fig 4.
  References:
  J. Cox, I. B. Witten, Striatal circuits for reward learning and decision-making. Nat. Rev. Neurosci. 20, 482–494 (2019).
  C. D. Hardman, J. M. Henderson, D. I. Finkelstein, M. K. Horne, G. Paxinos, G. M. Halliday, Comparison of the basal ganglia in rats, marmosets, macaques, baboons, and humans: volume and neuronal number for the output, internal relay, and striatal modulating nuclei. J. Comp. Neurol. 445, 238–255 (2002).
  A. Beauchamp, Y. Yee, B. C. Darwin, A. Raznahan, R. B. Mars, J. P. Lerch, Whole-brain comparison of rodent and human brains using spatial transcriptomics. Elife 11 (2022).
  J. H. Balsters, V. Zerbi, J. Sallet, N. Wenderoth, R. B. Mars, Primate homologs of mouse cortico-striatal circuits. Elife 9 (2020).
- **Thalamus**: Both humans and mice exhibit multi-nucleated thalamic structures, functioning in part to relay information from sensory to cortex, cortex to cortex, and from subcortex to cortex (Schmitt et al. 2017). Studies have demonstrated homologies between thalamo-cortical connectivity in mice and humans of primary sensory nuclei, including medial geniculate nucleus (Keifer et al. 2015). Notably, the ventral posteromedial nucleus is among the principal sensory nuclei in humans and mice (Morel et al. 1997). Given the size of the human intracranial electrodes and volume of tissue recorded relative to the size of thalamic nuclei, it is challenging to assign particular recordings to different human thalamic nuclei. Denoted "THAL ANT" and "THAL POS" in human Fig. 3; "MD" (mediodorsal nucleus), "VPM" (ventral posteromedial nucleus), "PO" (posterior complex), "LP" (lateral posterior), "TH" (other thalamic nuclei), "PoT" (posterior triangular nucleus) in mouse Fig. 4.
  References:
  L. I. Schmitt, M. M. Halassa, Interrogating the mouse thalamus to correct human neurodevelopmental disorders. Mol. Psychiatry 22, 183–191 (2017).
  O. P. Keifer Jr, D. A. Gutman, E. E. Hecht, S. D. Keilholz, K. J. Ressler, A comparative analysis of mouse and human medial geniculate nucleus connectivity: a DTI and anterograde tracing study. Neuroimage 105, 53–66 (2015).
  A. Morel, M. Magnin, D. Jeanmonod, Multiarchitectonic and stereotactic atlas of the human thalamus. J. Comp. Neurol. 387, 588–630 (1997).
- Notably, there are several regions we were able to record from in one species but not the other. In mice, we were uniquely able to record from brainstem and midbrain regions. In human participants, this category includes supramarginal gyrus and amygdala.

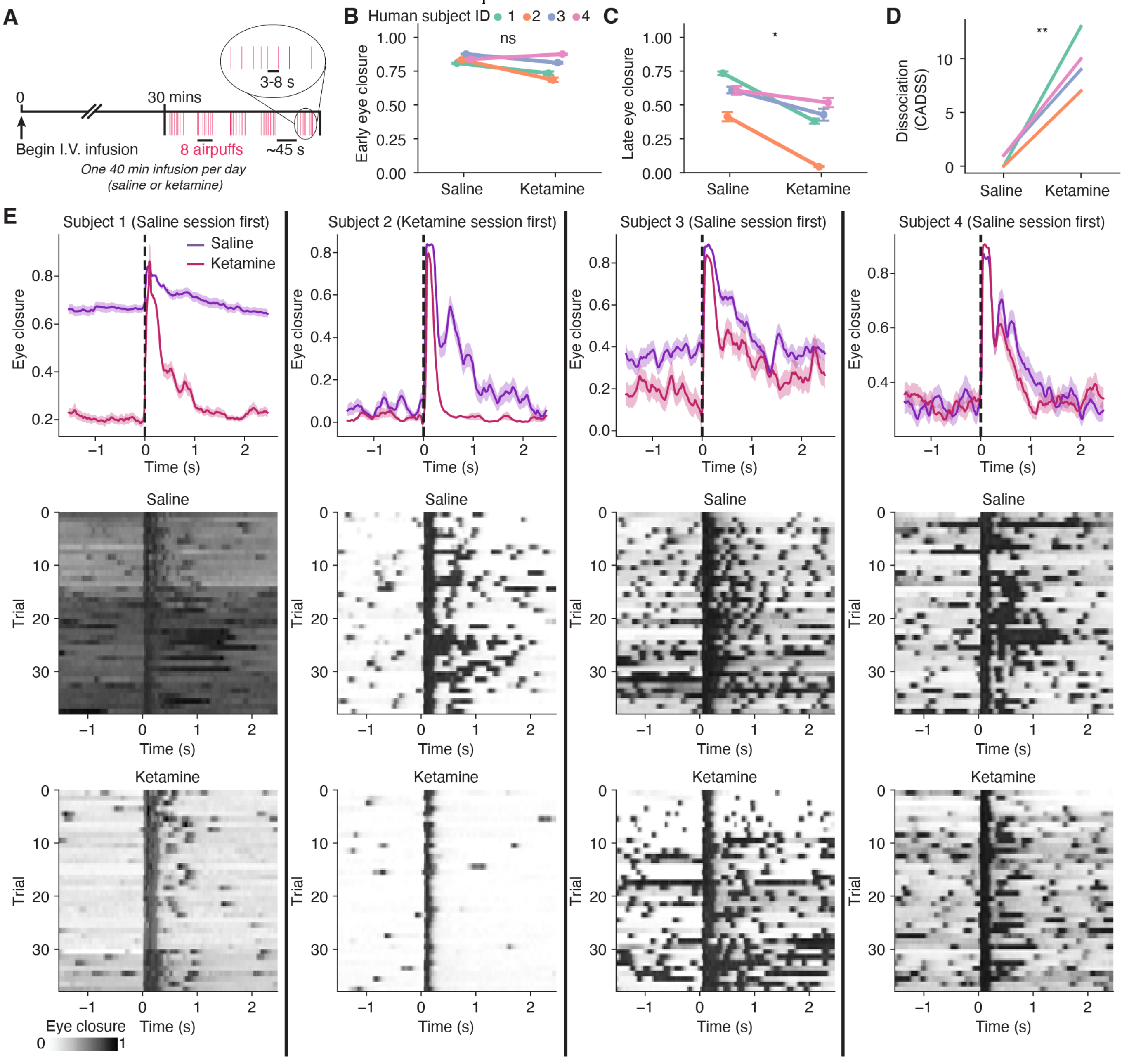

F

Description of eyepuffs during saline session			
"Hard to keep eyes open."	"It made me jump!"<br>"Wow, that was a lot less NOT unpleasant than [the ketamine session]!"	"It wasn't comfortable."<br>"It was annoying."	"Very unpleasant."

Description of eyepuffs during ketamine session			
"The air puff was not unpleasant, but more felt entertaining."<br>"I just did not give a f*** at all."	"It was this thing that was happening to me, but I wasn't really there paying attention to it."<br>"It felt like little whispers on my eyeballs"<br>"It wasn't at all unpleasant."	"Less bothersome."	"That was horrible."<br>"This is so strange."

Fig. S1. Additional details about eyepuff assay in humans. (A) Eyepuff protocol during infusion of ketamine or saline. During each session, a total of 40 air puffs are directed at the subject's left cornea, with an inter-puff interval that varies

between 3 to 8 s to prevent subjects from predicting the timing of each air puff. To reduce stress for the subject, there is a 35 s interval after every eighth puff. To allow for direct comparison, the same inter-puff interval timing sequence was used in saline and infusion sessions and across subjects. For analysis, we do not include the first two trials in order to exclude an initial response that can differ from subsequent trials. (B) Early eye closure (during 0.1 to 0.2 s after puff onset) does not change between saline and ketamine sessions (n=38 trials per subject, median ± 95% confidence interval). (C) Late eye closure (during 0.3 to 0.8 s after puff onset) decreases with ketamine relative to saline (n=38 trials per subject, median ± 95% confidence interval). (D) Dissociation increases during ketamine, as measured using the Clinician-Administered Dissociative States Scale (CADSS). (E) Puff-triggered eye closure for each subject. top: summary, mean ± s.e.m across trials. middle: saline individual puffs, bottom: ketamine individual puffs. (F) Self-reported descriptions of subjective experience of eyepuffs during saline or ketamine. ns, P-value ≥ 0.05. *, P-value < 0.05. **, P-value < 0.01. ***, P-value < 0.001. ****, P-value < 0.0001. See Supplementary table 3 for information on statistical analyses and sample sizes.

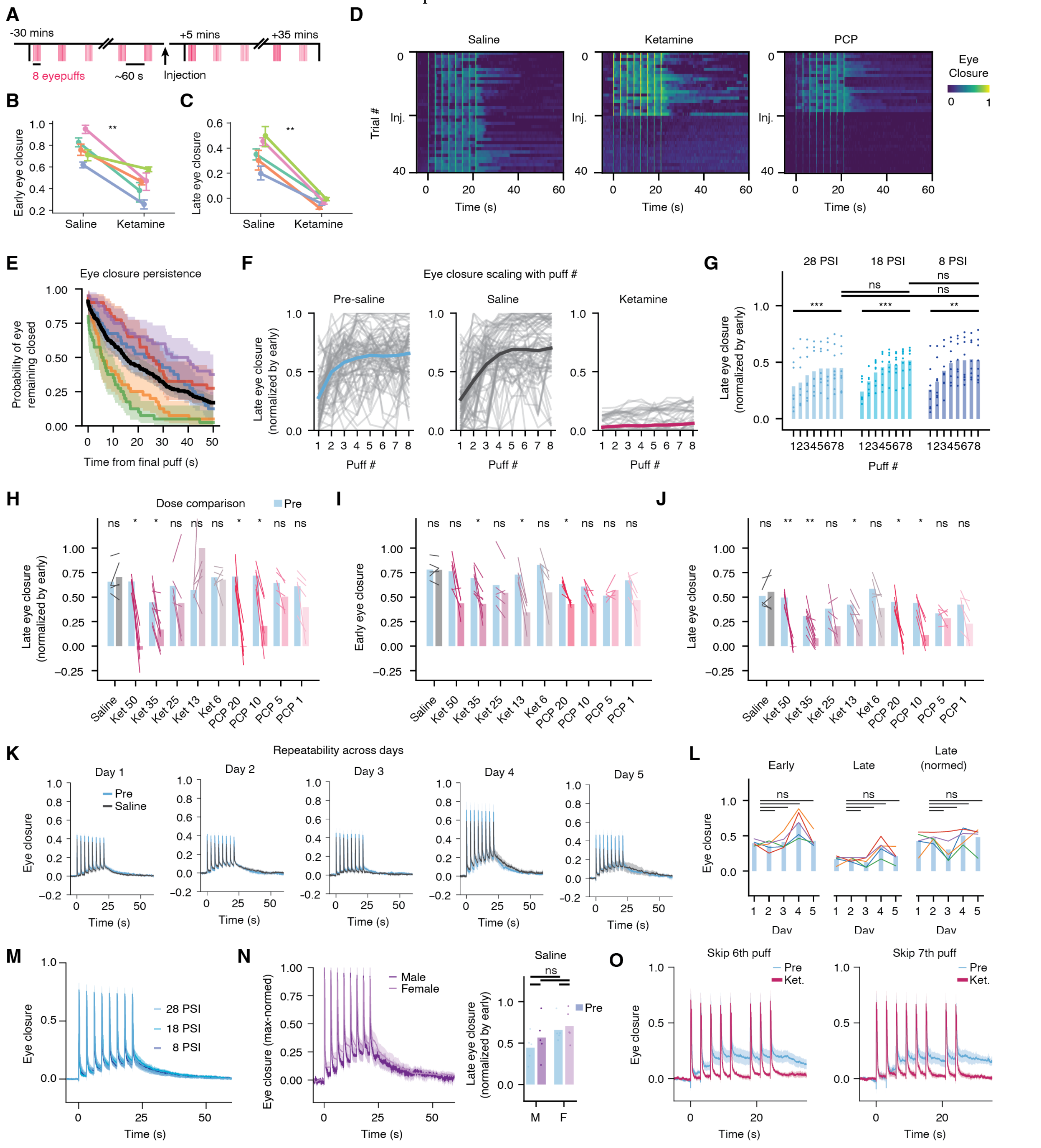

Fig. S2. Additional characterization of eyepuff assay in mice. (A) Eyepuff protocol with bolus drug infusion. Each trial consists of 8 air puffs with an inter-puff interval of 3 s. The inter-trial interval ranges from 50 to 70 s. Twenty trials are presented before infusion, and twenty trials after infusion. (B) Early eye closure (during 0.1 to 0.2 s after puff onset) decreases with ketamine but remains present (n=18 trials per subject, median ± 95% confidence interval). (C) Late eye closure (during 0.3 to 0.8 s after puff onset) is abolished by ketamine (n=18 trials per subject, median ± 95% confidence interval). (D) Eye closure on individual trials (each consisting of eight puffs) with saline, ketamine, or PCP infusion at trial

20. Saline elicits no change in eye closure, which consists of a fast eye blink in response to each puff and an increasing extended eye closure across puffs in a trial. In contrast, ketamine and PCP maintain the presence of the fast eye blink, but the extended eye closure is abolished. (E) Persistence of eye closure after final puff of each trial (Kaplan Meier survival time =15.16 s, n=100 trials from N=5 mice, each colored line is an individual mouse, median ± 95% confidence interval). (F) Scaling of late eye closure with number of puffs in a sequence, colored lines are mean across sequences, gray lines are individual sequences. The increase in late closure across the sequence of puffs has some variability on single trials, and on average scales upwards to a saturating level of closure. (G) Late (normed by early) eye closure scales with puff number but not puff intensity, comparing air puffs of different pressure (PSI, pounds per square inch). (H) Dose comparison of ketamine and PCP, specified in mg/kg for late normalized by early, (I) early, and (J) late eye closure. (K) Eye closure preceding and after saline infusion for the same mice across five days. While there can be some variability in the overall magnitude of eye closure, the early and late eye closures scale together (n=5 mice, mean ± s.e.m.). (L) Early, late, and late normalized by early eye closure on each day does not significantly differ from on the first day. (M) Eye closure during puff sequence with different puff intensities (n=8 mice, mean ± s.e.m.). (N) Male and female mice exhibit similar eye closure, preceding and after saline infusion (n=5 mice, mean ± s.e.m.). (O) Mice do not predict occurrence of a puff, and do not reflexively close their eye if a puff is skipped (n=5 mice, mean ± s.e.m.), while late eye closure remains persistently elevated during skipped puff. ns, P-value ≥ 0.05. *, P-value < 0.05. **, P-value < 0.01. ***, P-value < 0.001. ****, P-value < 0.0001. See Supplementary table 3 for information on statistical analyses and sample sizes.

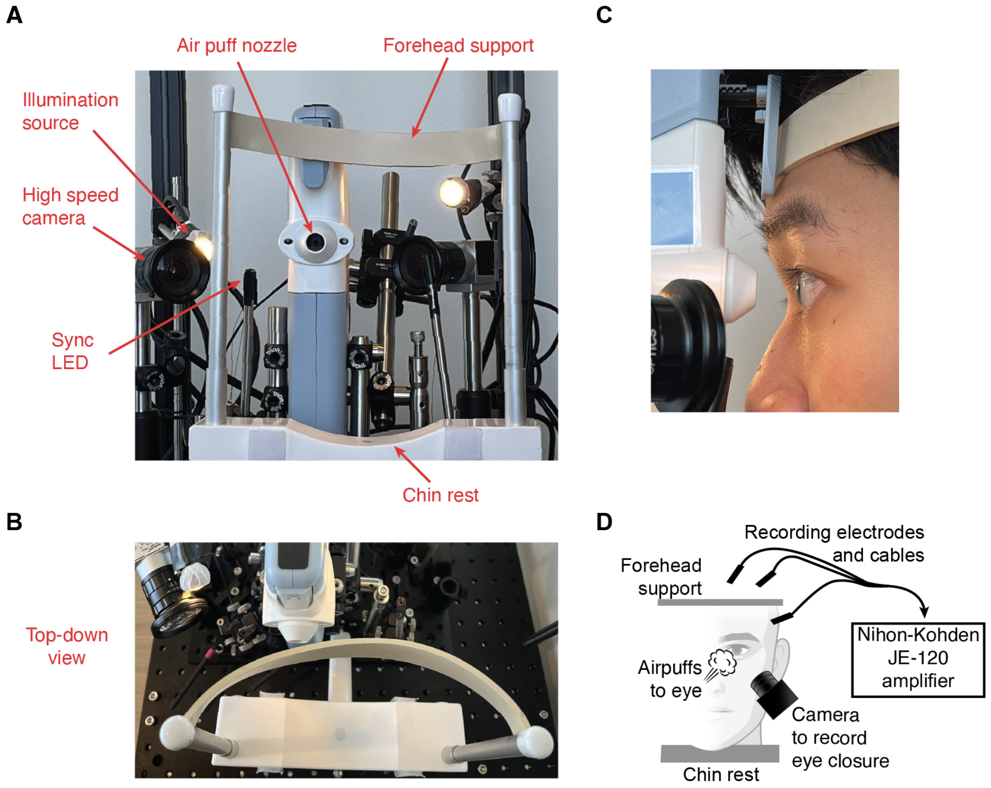

Fig. S3. Photos of the human eyepuff system. (A) Photo of eyepuff device, from the perspective of the subject before placement of head against the chin rest and forehead support. (B) Top-down view of the eyepuff device. (C) Example alignment of air puff nozzle towards subject's left eye, with the camera visible in the foreground of the image. (D) Schematic of iEEG recording during eyepuff assay.

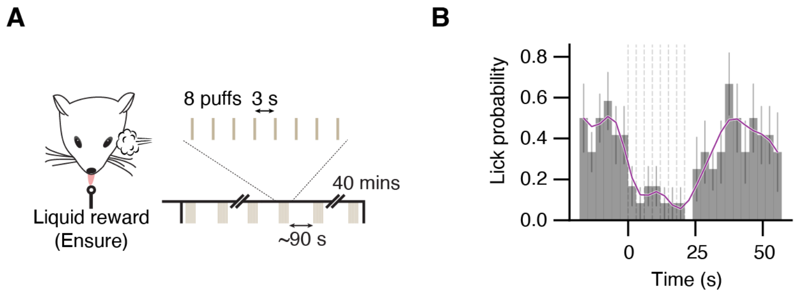

Fig. S4. Generalization of eyepuff-evoked aversive behavioral state to reduced consumption of liquid reward. (A) Schematic of protocol for measuring reward consumption during the eyepuff assay. Throughout the entire session, mice were free to collect a small amount of liquid reward (Ensure, a sweet caloric drink) from a spout within reach of their tongue, while the standard eyepuff assay was administered. A small amount of liquid was dispensed automatically every ~4.75 s (with exponentially distributed random time of [4.5, 5], mean 4.75). An additional small amount of water was dispensed if the spout was licked, with refractory period of 0.5 s. (B) Binned (3s) lick probability during eyepuff sequence. Purple line, smoothed probability estimate for ease of visualization. Lick probability decreased persistently during the air puff sequences and slowly recovered after the final air puff of the sequence, demonstrating that persistent behavioral responses to eyepuffs generalizes beyond eye closure, and providing additional evidence that eyepuffs induce a negative affective state.

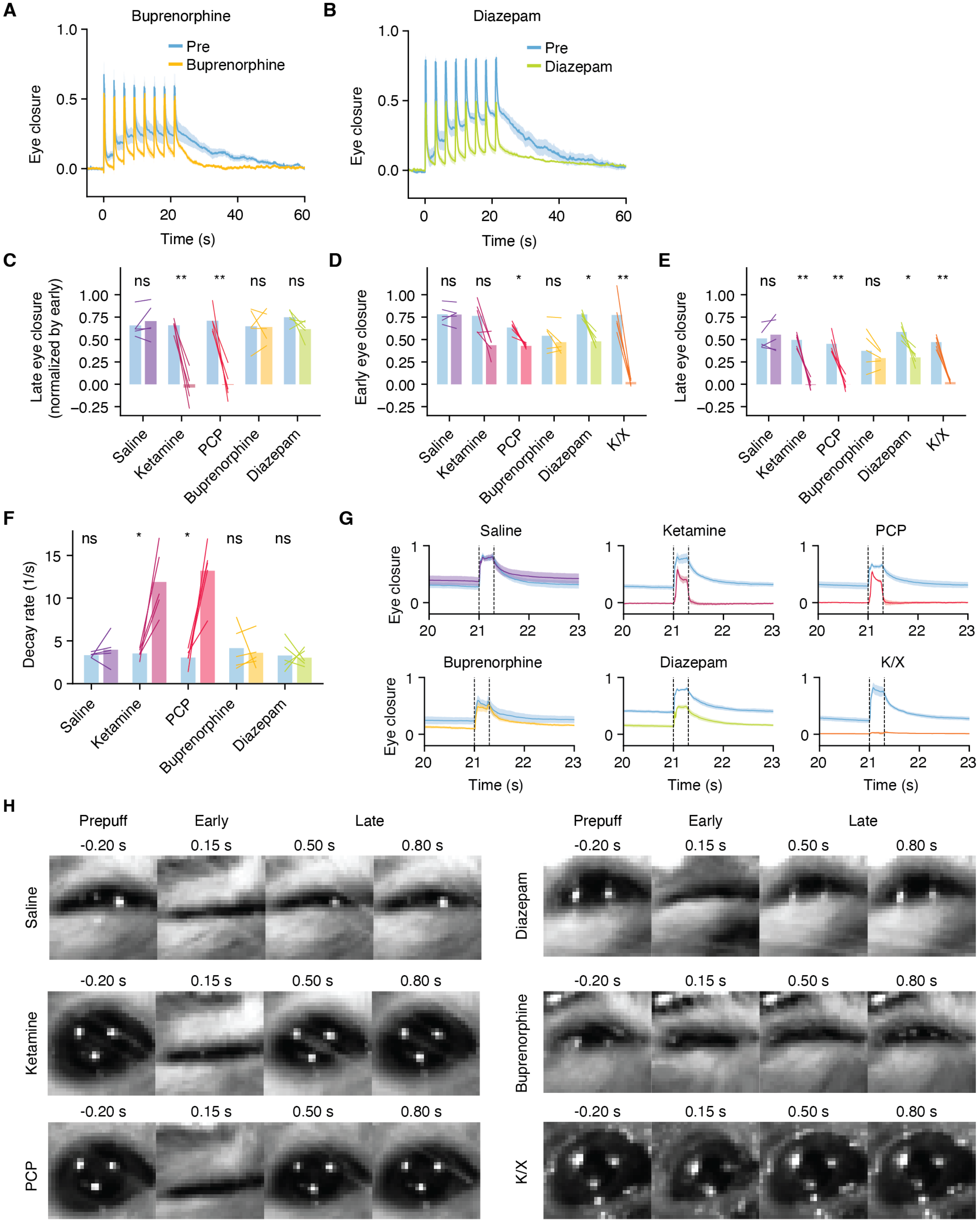

Fig. S5. Comparison of different drugs on eyepuff assay in mice. (A) Eye closure before and after injection of buprenorphine (an opioid analgesic, 2 mg/kg), and (B) diazepam (a benzodiazapene, 2 mg/kg). (A-B, n=5 mice, mean ± s.e.m). (C) Only ketamine (50 mg/kg) and PCP (20 mg/kg) decrease late normalized by early eye closure. (D) Change in

early eye closure and (E) late eye closure. Diazepam and K/X both yield significant decreases in early and late eye closure, as a consequence of overall eye closure decreasing but without late eye closure specifically exhibiting a greater reduction. (F) Decay rate of an exponential fit to eye closure during 0.2 to 2 s after the onset of the final (eighth) puff in each trial. (G) Eye closure during the final puff in each trial (which occurs 21 s after the onset of the first puff in a trial). Unlike with Saline, Buprenorphine, and Diazepam, with Ketamine and PCP there is no accumulation of extended eye closure across the sequence of puffs. (n=5 mice, mean ± s.e.m). (H) Example images of eye preceding, during, and after the final puff of a trial, after infusion of indicated drug. With all drugs except for K/X, there is an early eye closure, but with Ketamine and PCP the eye quickly opens wide. ns, P-value ≥ 0.05. *, P-value < 0.05. **, P-value < 0.01. ***, P-value < 0.001. ****, P-value < 0.0001. See Supplementary table 3 for information on statistical analyses and sample sizes.

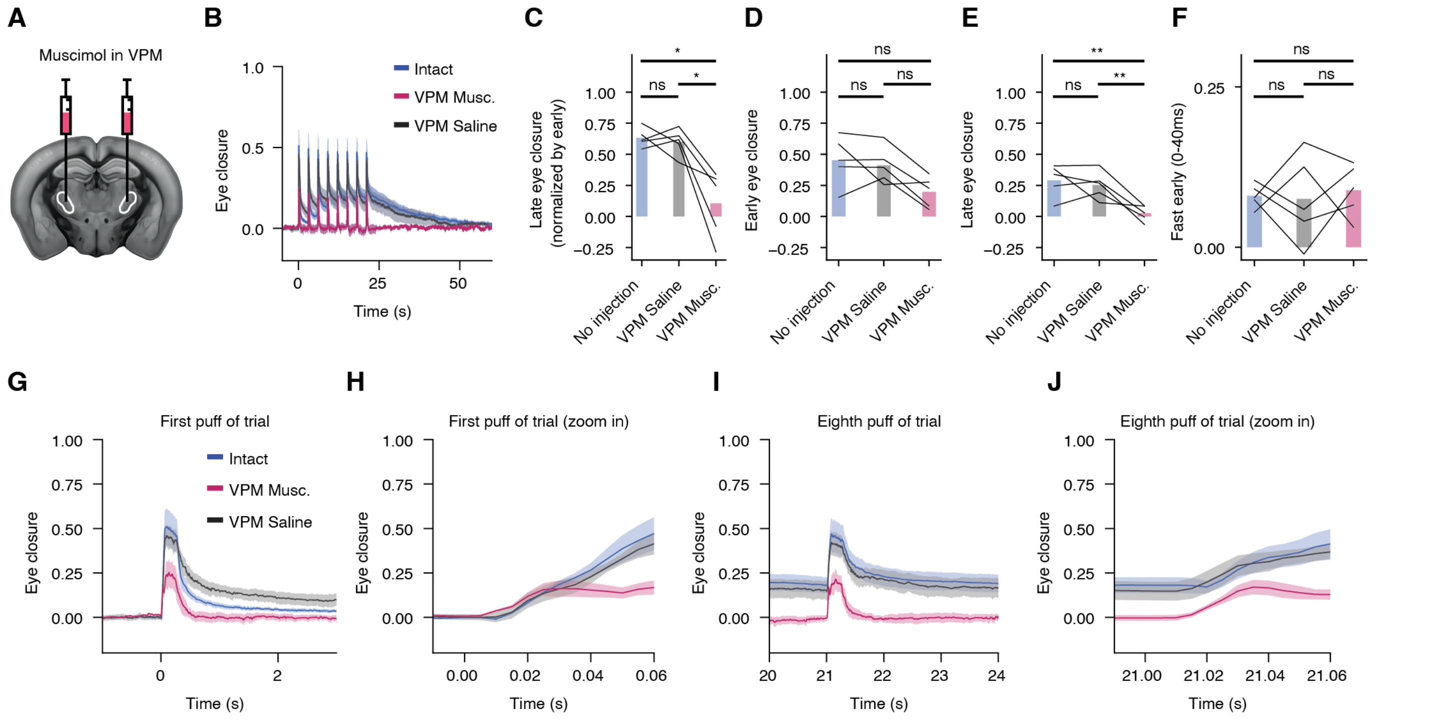

Fig. S6. Inhibition of mouse VPM thalamus with muscimol reduces affective eyepuff response. (A) Location of bilateral muscimol injection (coordinates: ± 1.6 ML, -1.9 AP, -3.6 DV). (B) Eye closure during puff sequence in each condition. With muscimol, there is early eye closure after each puff, but no accumulating late eye closure. (C) Late (normalized by early) eye closure. (D) Early eye closure. (E) Late eye closure. (F) Fast early eye closure (within first 40 ms after the first puff of each trial). (G) Eye closure during first puff and (H) zoomed in. While the early eye closure does not reach the overall same magnitude, the fast component of the early eye closure is nearly identical to the intact and saline settings. (I) Eye closure during final puff (which occurs at 21 s after the onset of the first puff), and (J) zoomed in. All traces are mean ± s.e.m., n=5 mice. ns, P-value ≥ 0.05. *, P-value < 0.05. **, P-value < 0.01. ***, P-value < 0.001. ****, P-value < 0.0001. See Supplementary table 3 for information on statistical analyses and sample sizes.

A

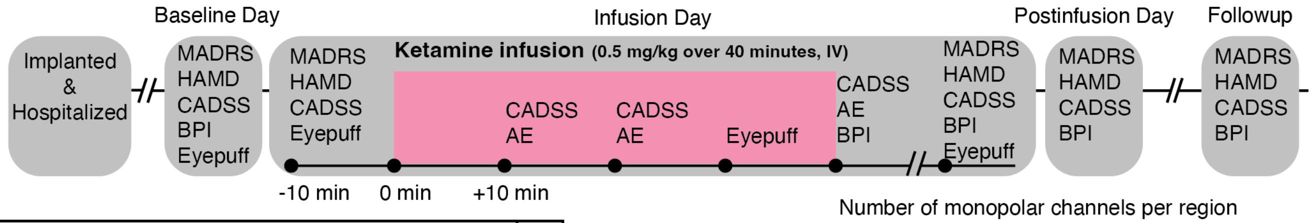

B

Number of participants contacted	42
Number of participants signed the consent form	27
Number of participants completed the study	21
Number of participants withdrew/withdrawn from study	6
Number of participants administered eyepuff assay	16
Number of participants meeting eyepuff inclusion criteria	7

C

ACC	Anterior Cingulate Cortex
AG	Angular Gyrus
AMY	Amygdala
BG	Basal Ganglia
CLA	Claustrum
CS	Collateral Sulcus
CUN	Cuneus
FG	Fusiform Gyrus
FO	Frontal Operculum
HIPP	Hippocampus
IC	Internal Capsule
IFG	Inferior Frontal Gyrus
INS	Insular Cortex
IPS	Intraparietal Sulcus
ITG	Inferior Temporal Gyrus
LG	Lingual Gyrus
MCC	Mid-cingulate Cortex
MFC	Medial Frontal Cortex
MFG	Middle Frontal Gyrus
MOG	Middle Occipital Gyrus
MOT	Motor Cortex
MSFG	Medial Superior Frontal Gyrus
MTG	Middle Temporal Gyrus
OP	Operculum
ORB	Orbitofrontal Cortex
OTS	Occipitotemporal Sulcus
PHG	Parahippocampal Gyrus
PMC	Posteromedial Cortex
POSTCG	Post Central Gyrus
PRECG	Pre Central Gyrus
SFG	Superior Frontal Gyrus
SMC	Somatosensory + Motor Cortex
SMG	Supramarginal Gyrus
SPL	Superior Parietal Lobule
SS	Somatosensory Cortex
STG	Superior Temporal Gyrus
STS	Superior Temporal Sulcus
THAL	Thalamus
THAL ANT	Anterior Thalamus
THAL CM	Centromedian Thalamus
THAL MD	Mediodorsal Thalamus
THAL MID	Middle Thalamus
THAL POS	Posterior Thalamus
THAL VLA	Ventral Lateral Anterior Thalamus
THAL VLD	Ventral Lateral Dorsal Thalamus
TP	Temporal Pole
VMPFC	Ventromedial Prefrontal Cortex

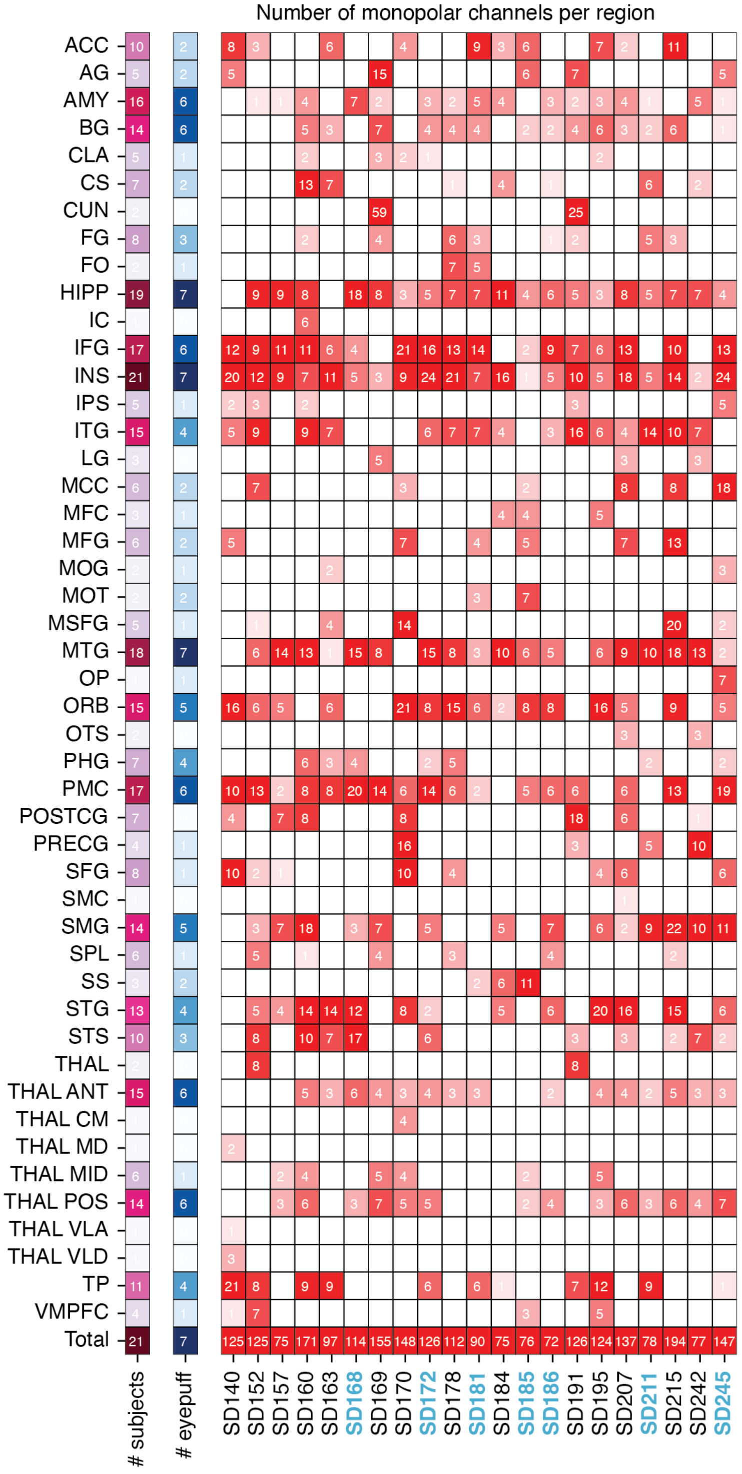

Fig. S7. Human iEEG study details. (A) Full study protocol, including psychiatric questionnaires, ketamine infusion, and eyepuff assay. (B) Participant study completion. See Methods for explanation of inclusion criteria. Note that we excluded from eyepuff analysis the 2 participants who received bolus infusions, because dosing and timing were not comparable to the other participants, and we thus considered 14 participants for the eyepuff assay in the main text. (C) Region acronym

legend. (D) Anatomical distribution for included subjects. From left: number of subjects with at least one channel in a given region; number of subjects meeting inclusion criteria for eyepuff analysis that have at least one channel in a given region; and number of monopolar channels per region for each subject. Eyepuff subjects meeting inclusion criteria are specified in blue.

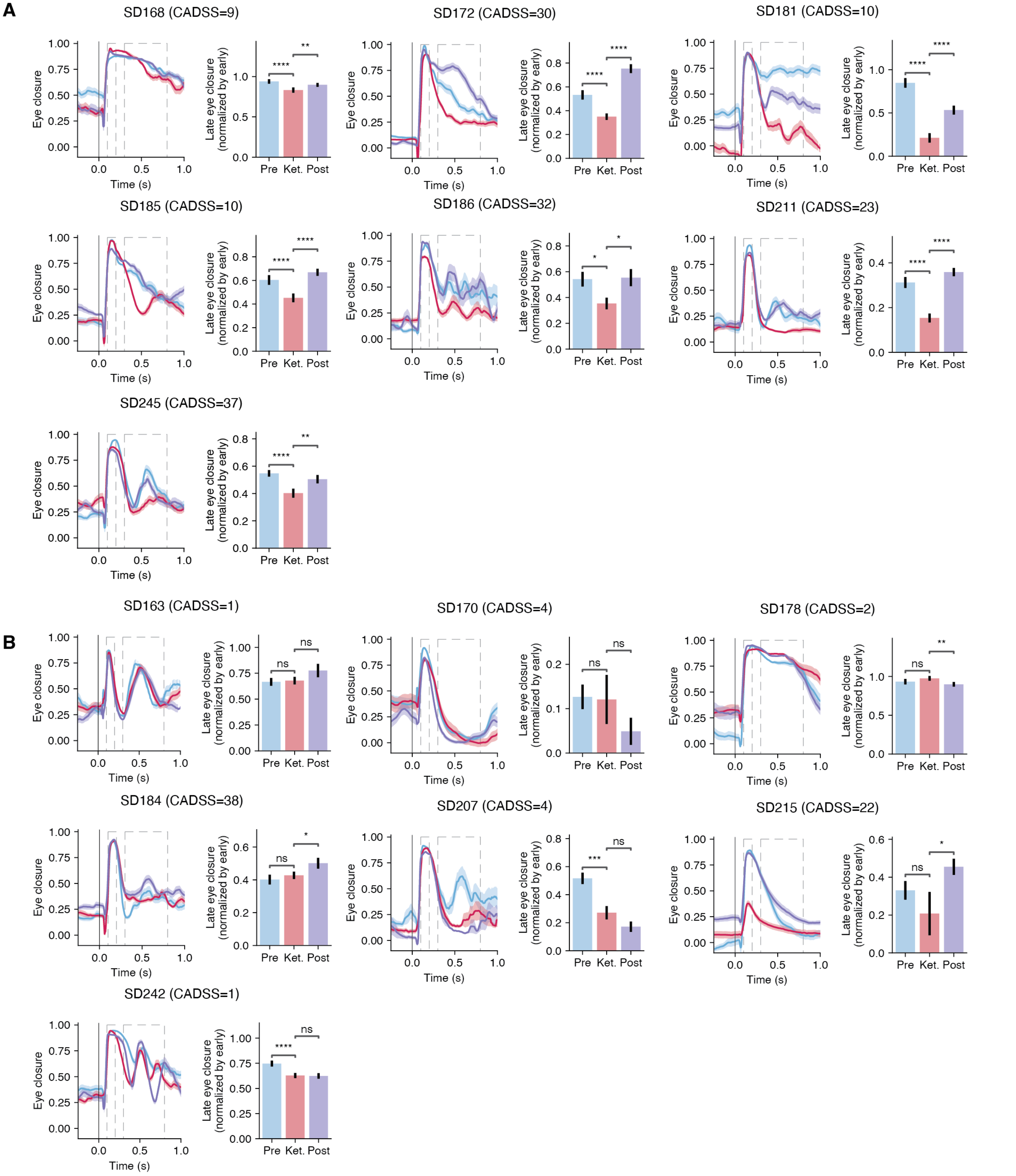

Fig. S8. Additional details for human eyepuff behavior. (A) Eye closure for each condition for subjects that meet the behavioral inclusion criteria for neural analysis of eyepuff-triggered dynamics. Left: eye closure timecourse (dashed boxes: left, window for quantifying early eye closure, right, window for quantifying late eye closure). Right: summary of late eye closure normalized by early eye closure. Dissociation symptoms during infusion (CADSS score) indicated for each subject.

(B) Eye closure of subjects not meeting the inclusion criteria. Inclusion criteria was defined as a subject exhibiting significant change in late eye closure normalized by early eye closure: preinfusion vs. ketamine, and ketamine vs. postinfusion (Methods).Timecourse plots, mean ± s.e.m., n=12 to 39 trials depending on subject. Vertical dashed lines represent, starting from left: onset of early window, offset of early window, onset of late window, offset of late window. ns, P-value ≥ 0.05. *, P-value < 0.05. **, P-value < 0.01. ***, P-value < 0.001. ****, P-value < 0.0001. See Supplementary table 3 for information on statistical analyses and sample sizes, and Methods for additional description of inclusion criteria.

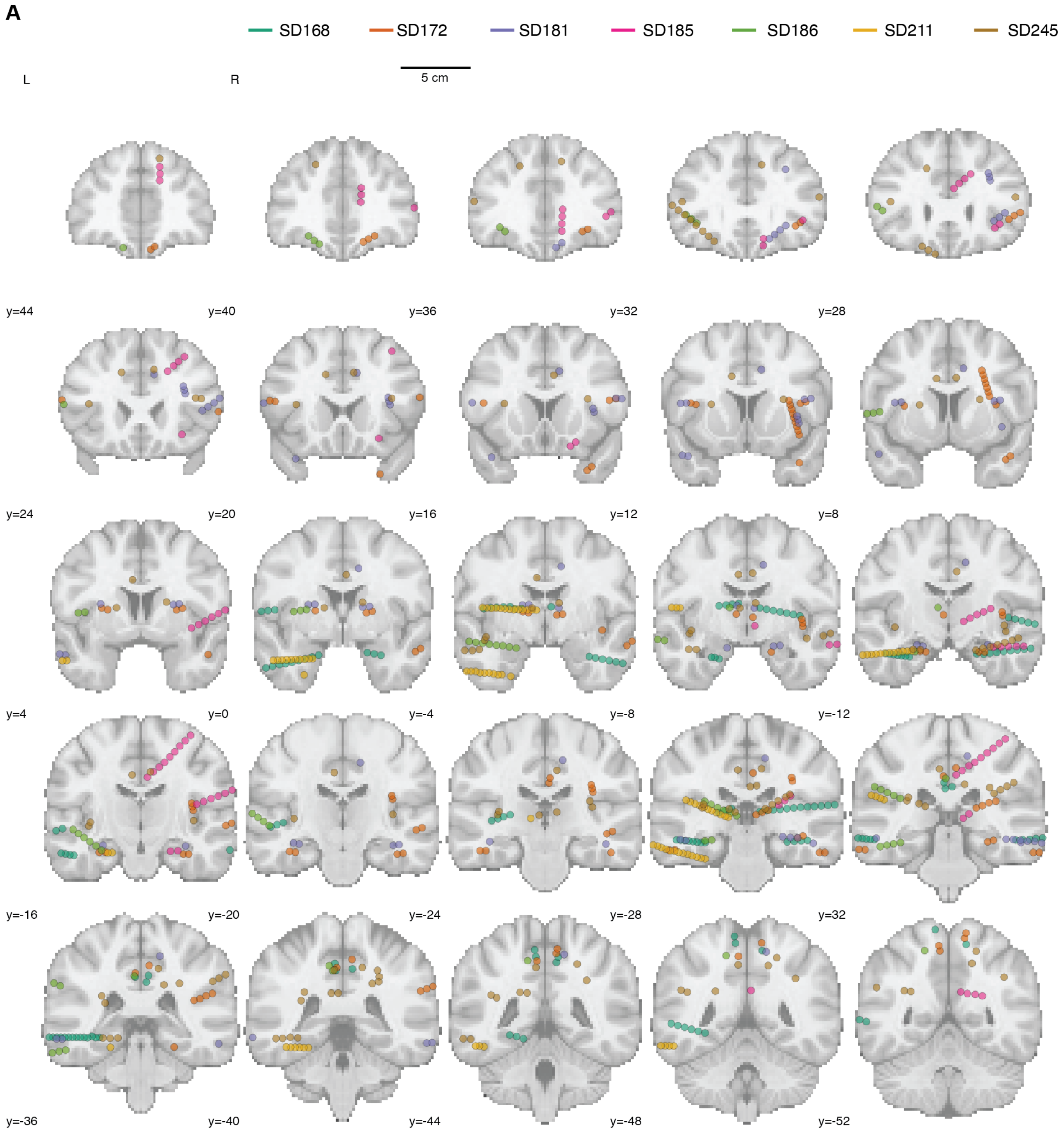

Fig. S9. Human recording sites for subjects that meet eyepuff assay behavioral inclusion criteria. (A) Recording sites transformed into MNI coordinates and plotted on coronal slices of MNI152 template.

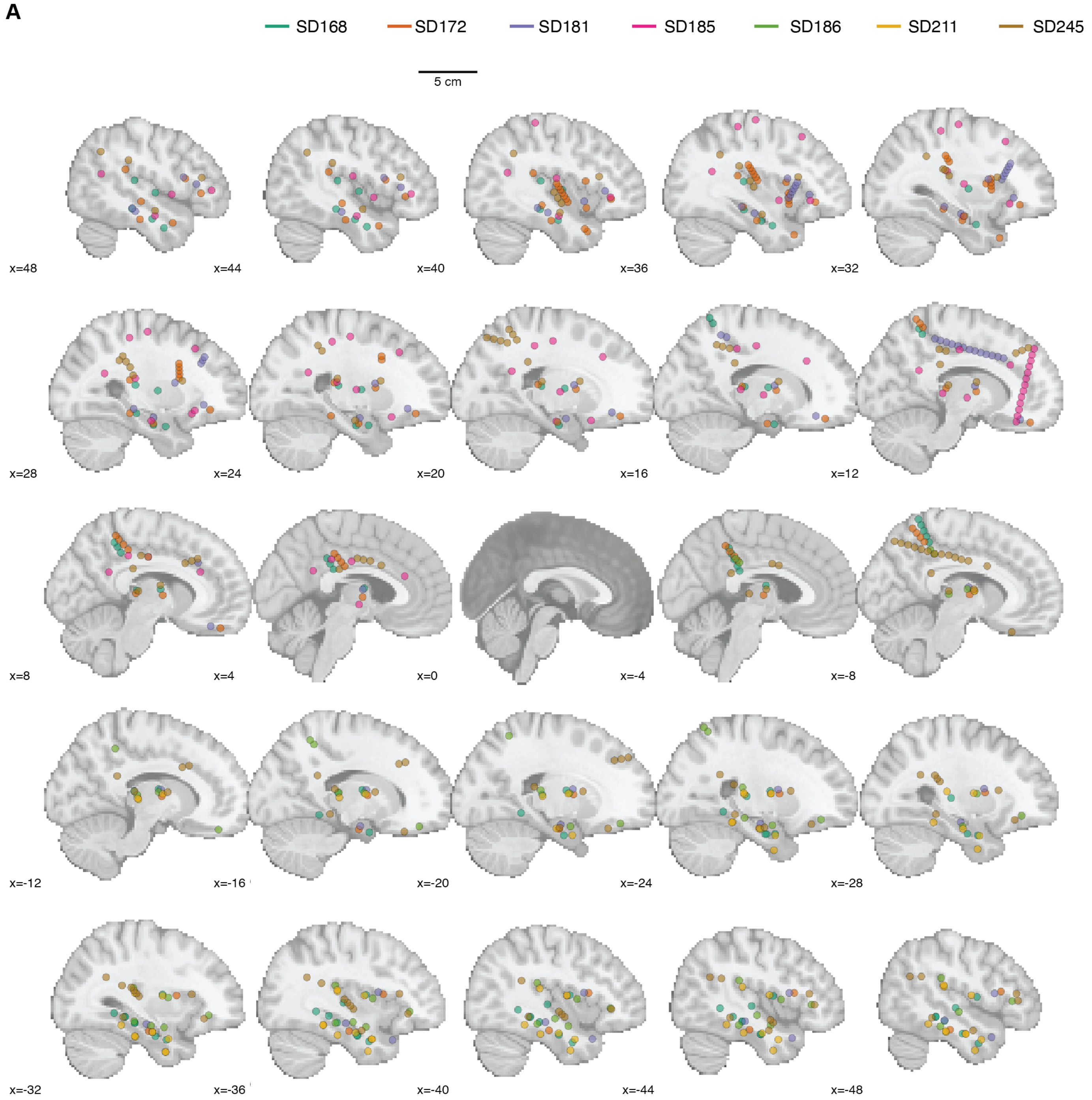

Fig. S10. Human recording sites for subjects that meet eyepuff assay behavioral inclusion criteria. (A) Recording sites transformed into MNI coordinates and plotted on sagittal slices of MNI152 template.

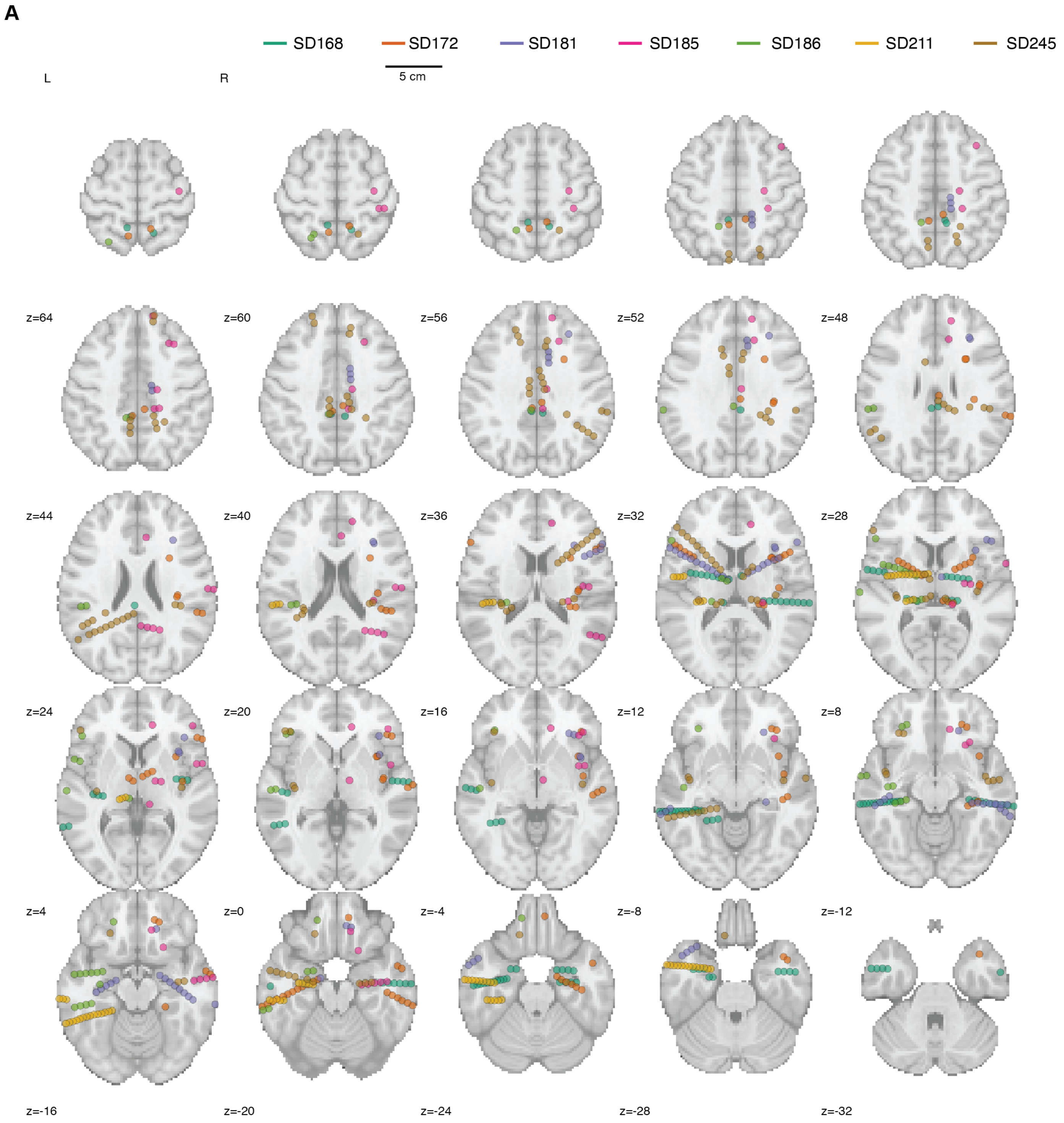

Fig. S11. Human recording sites for subjects that meet eyepuff assay behavioral inclusion criteria. (A) Recording sites transformed into MNI coordinates and plotted on horizontal slices of MNI152 template.

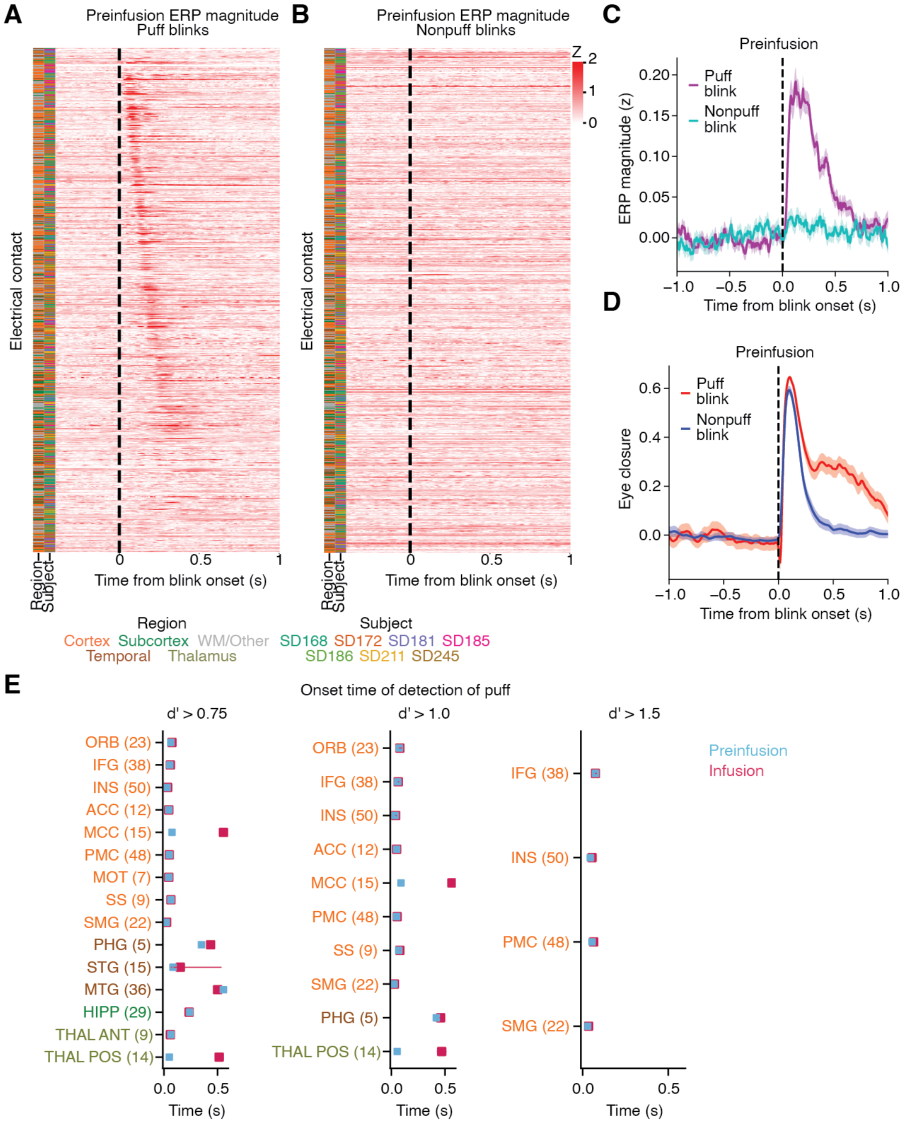

Fig. S12. Human puff-triggered electrical activity is different than activity during non-puff-triggered eyeblinks. (A) Preinfusion puff-triggered event related potential (ERP) for all channels (n=773 channels from N=7 subjects), sorted using peak time of a separate validation set of trials. (B) Same ordering, ERP of non-puff-triggered eyeblinks. (C) Magnitude of ERP is much larger during puff-triggered eyeblinks (n=773 total channels from 7 subjects, mean ± s.e.m., ERP for each channel computed from 6 to 9 blink events). (D) Puff-triggered and non-puff-triggered eyeblinks reach the same magnitude of eye closure (n=52 trials from 7 subjects, mean ± s.e.m.). (E) Onset time of detection (d' across trials) of puff by the average puff-triggered LFP trace across all channels in a region, for different d' thresholds. Regions across the brain, and especially cortex, exhibit fast detectability of the puff during both preinfusion and infusion. Error bars are bootstrap 95% confidence intervals. See Supplementary table 3 for information on statistical analyses and sample sizes.

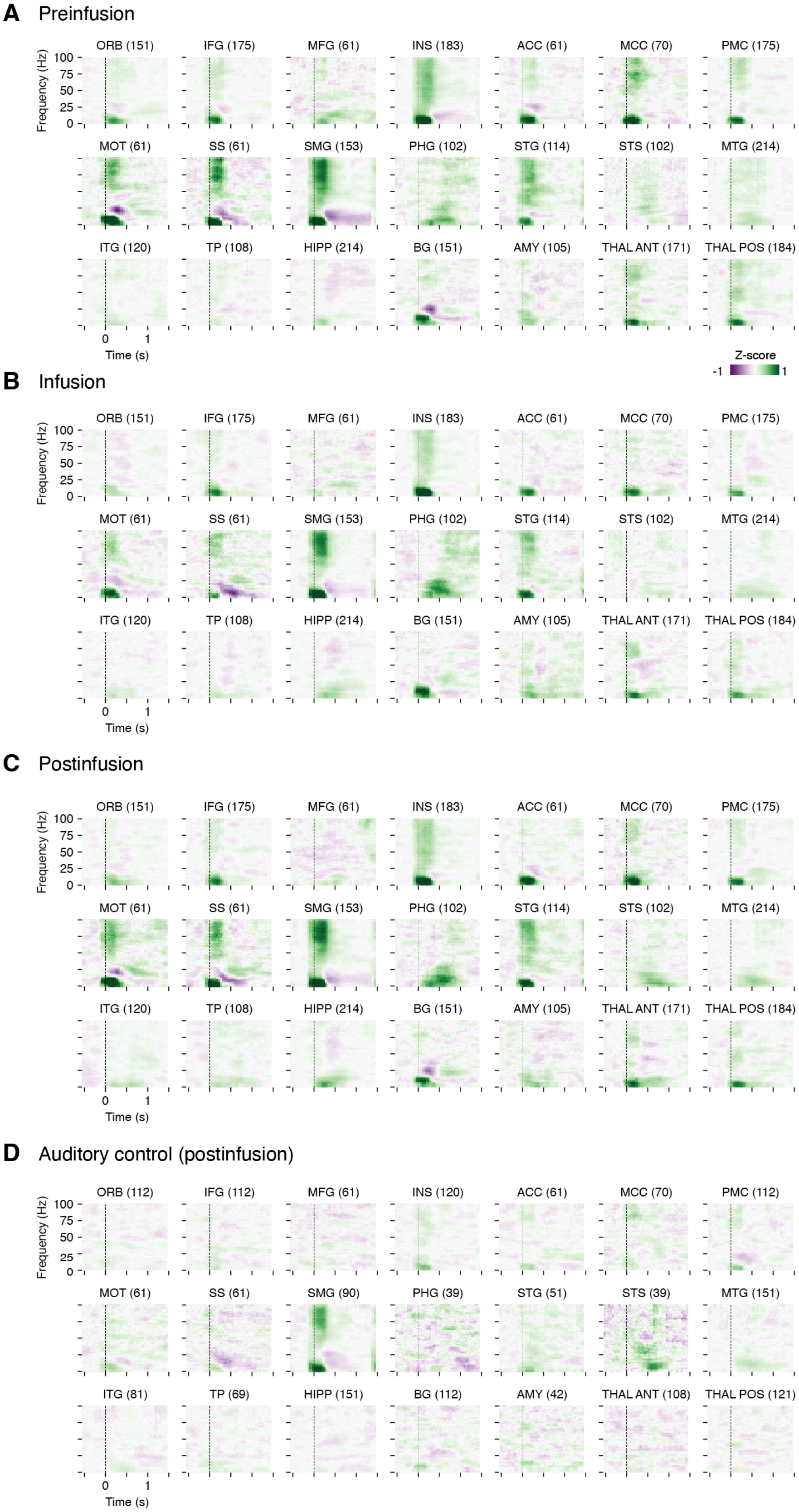

Fig. S13. Human puff-triggered spectrograms by brain region. (A) Preinfusion, (B) Infusion, (C) Postinfusion, (D) Auditory control (following Postinfusion assay, the eyepuff assay protocol is run but with the air puff nozzle pointed away from the subject). For each subject, a regional trace was computed as the average across channels in that region; all trials for a region were then stacked across subjects. Total number of trials indicated next to each region; different regions were

sampled in different subsets of subjects, so the number of trials differed across regions. Similar patterns of puff-triggered dynamics are exhibited by regions across the brain, suggesting there may be a low dimensional representation of the puff-triggered response. Dashed vertical line, air puff onset.

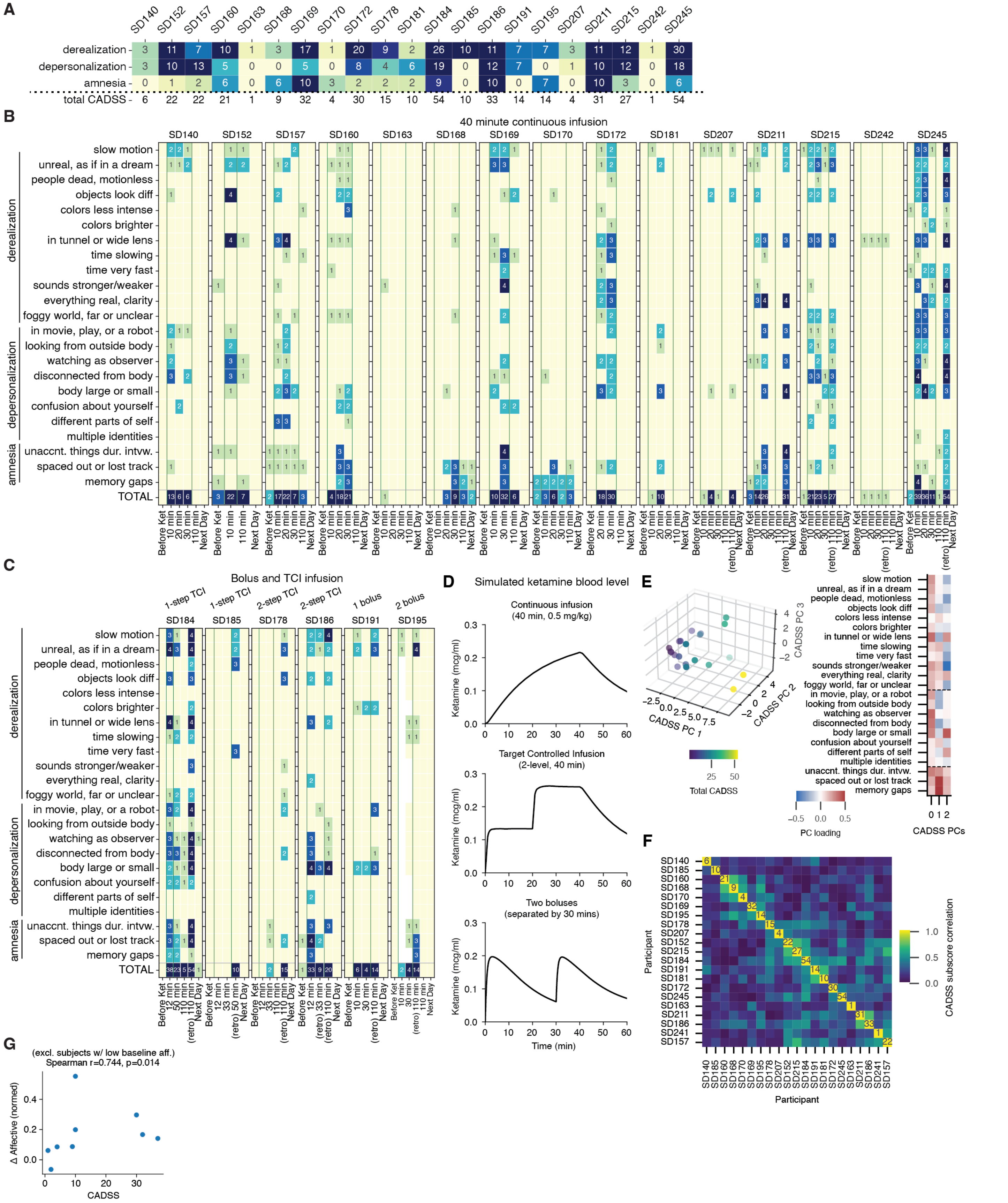

Fig. S14. Diverse Clinician-Administered Dissociative States (CADSS) symptom profiles across human participants receiving ketamine. (A) Summary of peak CADSS scores across study subjects. (B) CADSS scores across time points for

subjects receiving continuous 40-minute infusions (0.5 mg/kg). Retro denotes retrospective CADSS, in which a patient recounted after the infusion their peak experience during the infusion. (C) CADSS scores across time points for subjects receiving bolus or target-controlled infusions (TCI). (D) Simulated ketamine blood levels for different infusion types in (B) and (C), using StanpumpR PK/PD simulation program. (E) Left: Distribution of patients in dimensionality-reduced CADSS space (each point is a patient). Right: Loading of CADSS subquestions across first three PC axes. (F) Correlation of each patients' CADSS subscores. Patients are sorted using hierarchical clustering. Patient total CADSS scores are annotated along diagonal. There appear to be soft clusters of CADSS response-types, however this study was not powered to investigate those differences. (G) Spearman correlation between ketamine change in affective normalized by reflexive eye closure (Δ defined here as: infusion – mean(preinfusion, postinfusion)) vs. CADSS score. Only subjects with preinfusion normalized affective eye closure > 0.5 were included in this analysis, to ensure that there was an adequate baseline level from which to detect changes. See Supplementary table 3 for information on statistical analyses and sample sizes.

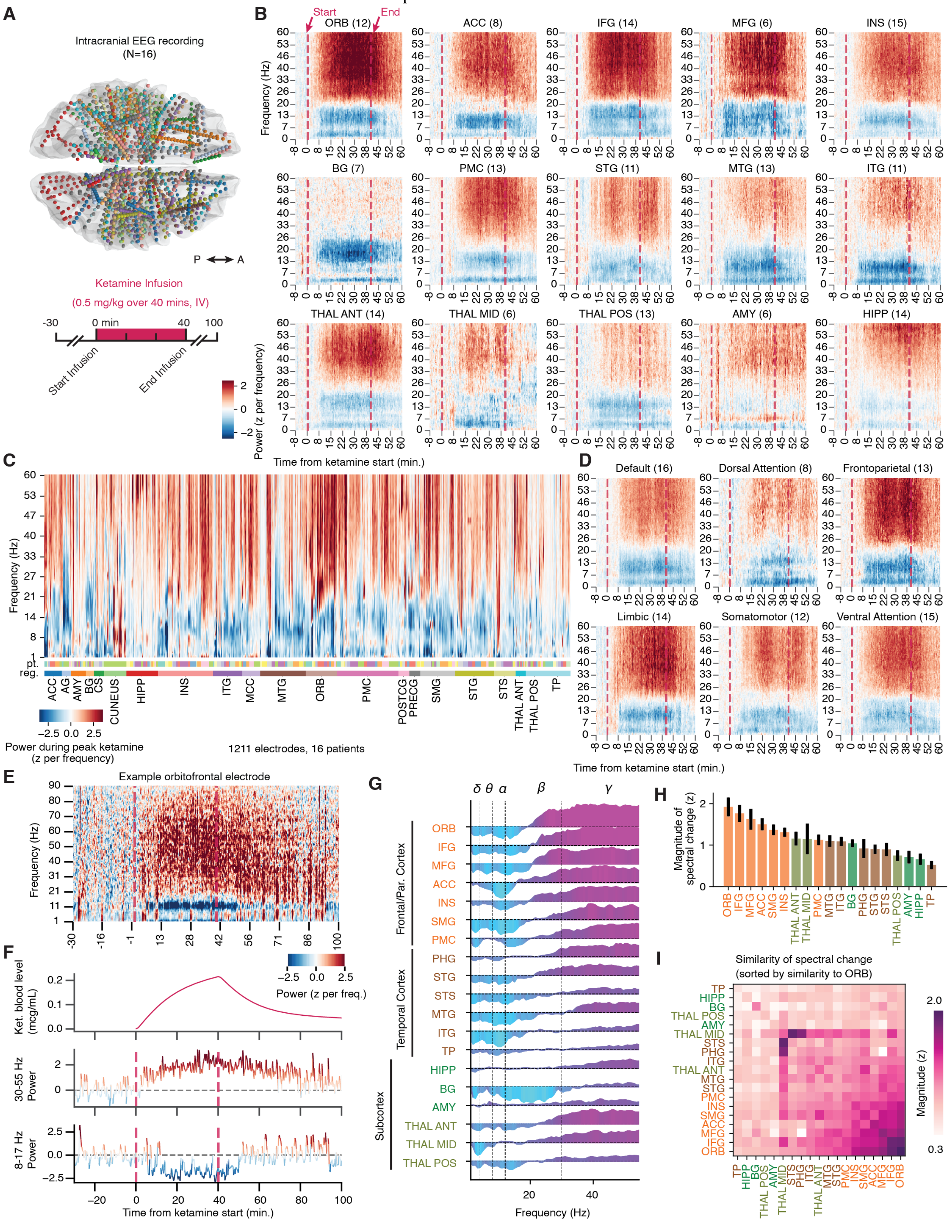
A
Intracranial EEG recording
(N=16)
P A
Ketamine Infusion
(0.5 mg/kg over 40 mins, IV)
-30
0 min
40
100
Start Infusion
End Infusion
B
Start
End
ORB (12)
ACC (8)
IFG (14)
MFG (6)
INS (15)
BG (7)
PMC (13)
STG (11)
MTG (13)
ITG (11)
THAL ANT (14)
THAL MID (6)
THAL POS (13)
AMY (6)
HIPP (14)
Frequency (Hz)
Power (z per frequency)
Time from ketamine start (min.)
C
Frequency (Hz)
pt.
reg.
ACC
AG
AMY
BG
CS
CUNEUS
HIPP
INS
ITG
MCC
MTG
ORB
PMC
POSTCG
PRECG
SMG
STG
STS
THAL ANT
THAL POS
TP
Power during peak ketamine
(z per frequency)
1211 electrodes, 16 patients
D
Default (16)
Dorsal Attention (8)
Frontoparietal (13)
Limbic (14)
Somatomotor (12)
Ventral Attention (15)
Time from ketamine start (min.)
E
Example orbitofrontal electrode
Frequency (Hz)
Power (z per freq.)
F
Ket. blood level
(mcg/mL)
30-55 Hz
Power
8-17 Hz
Power
Time from ketamine start (min.)
G
δ θ α β γ
Frontal/Par. Cortex
Temporal Cortex
Subcortex
ORB
IFG
MFG
ACC
INS
SMG
PMC
PHG
STG
STS
MTG
ITG
TP
HIPP
BG
AMY
THAL ANT
THAL MID
THAL POS
Frequency (Hz)
H
Magnitude of
spectral change (z)
ORB
IFG
MFG
ACC
SMG
INS
THAL ANT
THAL MID
PMC
MTG
ITG
BG
PHG
STG
STS
THAL POS
AMY
HIPP
TP
I
Similarity of spectral change
(sorted by similarity to ORB)
Magnitude (z)
2.0
0.3

Fig. S15. Human intracranial spectral power changes during ketamine infusion. (A) Schematic of intracranial neural measurements conducted during 40-minute ketamine infusions. (B) Average spectrograms across regions of ketamine-induced power changes. Values are z-scored at each frequency to a 25-minute pre-ketamine baseline window. Numbers in parentheses denote patient count. (C) Spectral power changes, averaged during 30-40 minute peak ketamine window. Each column is an individual channel. (D) Average spectral power changes for Yeo7-defined functional networks. Numbers in parentheses denote patient count. (E) Example spectrogram from a single orbitofrontal bipolar channel. (F) Ketamine pharmacokinetics (top), compared to spectral power change over time shown in (E) for high (middle) and low (bottom) power bands. (G) Change in power (ketamine – preinfusion), z-scored at each frequency, median across subjects. Sorted anatomically, dark green: subcortical, lime green: thalamus, orange: frontal/parietal cortical, brown: temporal cortical. (H) Magnitude of spectral change, by region (mean ± s.e.m.). Frontal cortex exhibited the largest magnitudes of change, led by orbitofrontal cortex. (I) Similarity of spectral changes, sorted by similarity to orbitofrontal cortex. Cortical regions exhibited similar changes, especially frontal cortex, that were also reflected by anterior thalamus and to a lesser extent mid-thalamus. Other subcortical regions, such as basal ganglia, exhibited distinct changes.

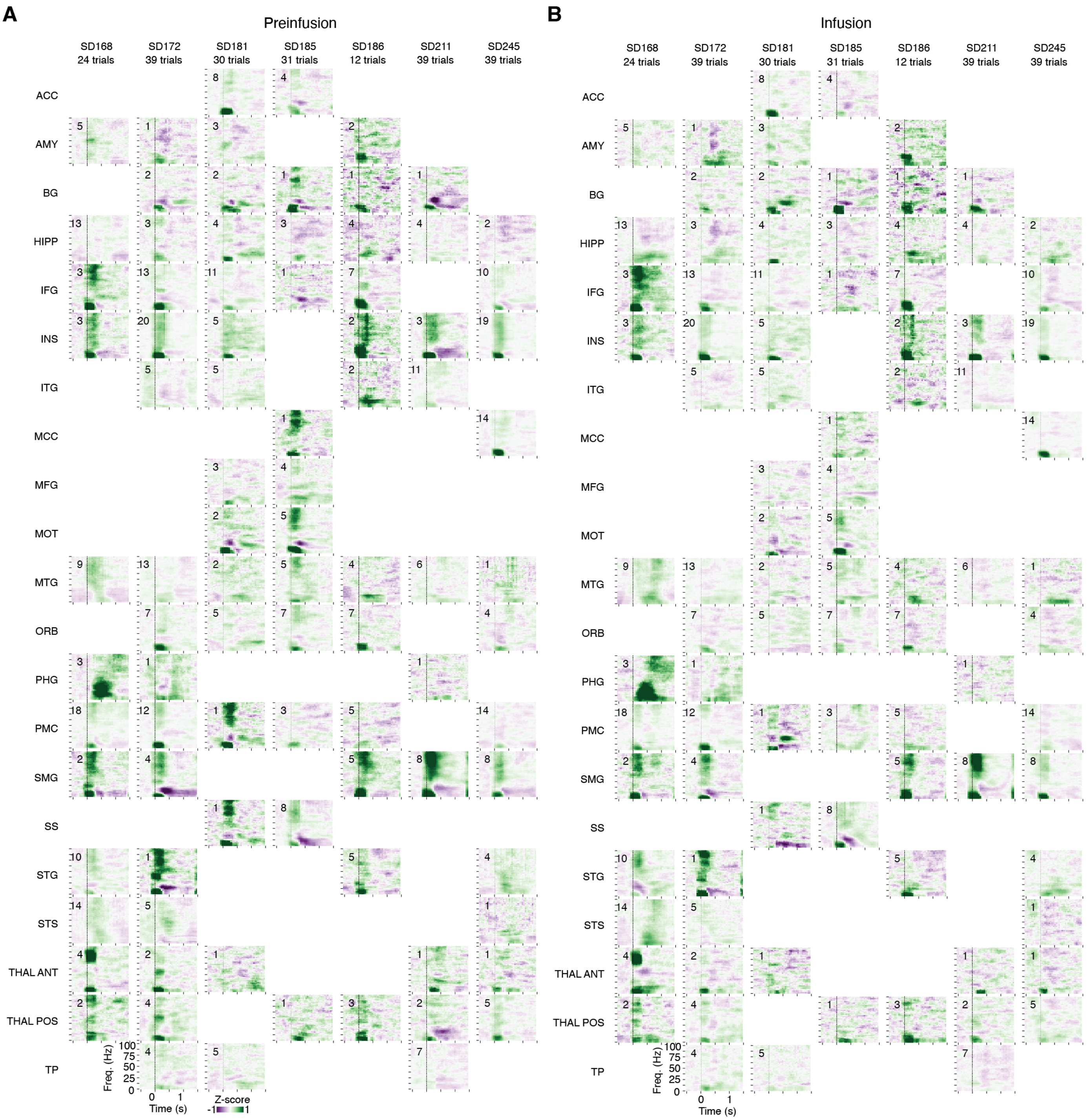

Fig. S16. Puff-triggered spectrogram, by human subject and region, averaged across trials. (A) Preinfusion and (B) Infusion. Number of channels indicated in top left corner of each spectrogram.

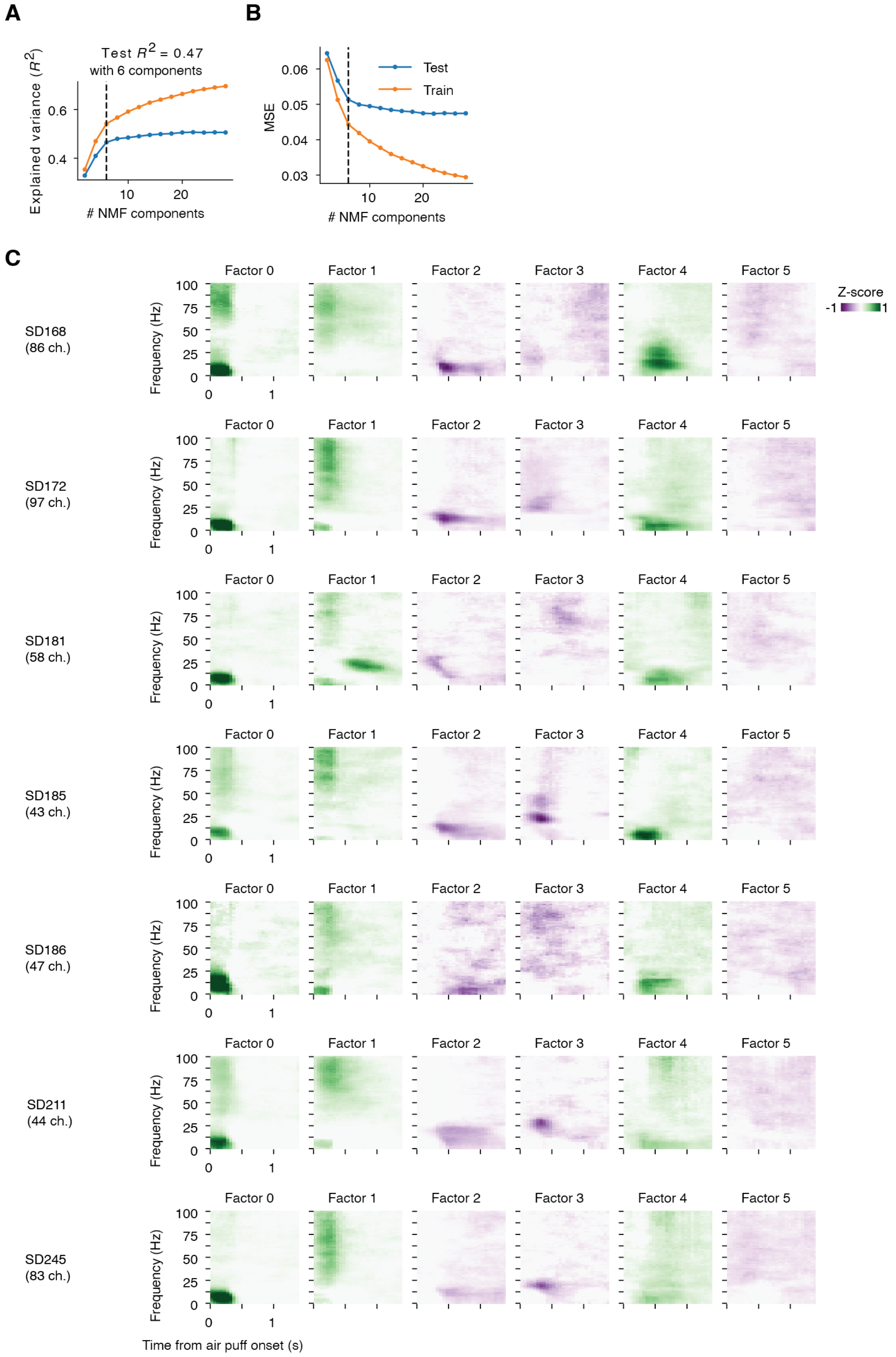

Fig. S17. Additional detail on human puff-triggered spectrogram factorization. (A) Explained variance on a train and test set (with the same channels, but using different sets of trials to compute the trial-averaged spectrogram, split ratio = 0.5) as a function of the number of NMF components. As indicated by performance on the test set, the optimal number of components is determined by the location of the ‘elbow’ to be six, past which there are diminishing returns for increasing the number of components. (B) Mean squared error (MSE) on a test set of trials as a function of number of NMF components. (C) Factors computed on each subject individually, showing that most factors are present in some form in all subjects. See Supplementary table 3 for information on statistical analyses and sample sizes.

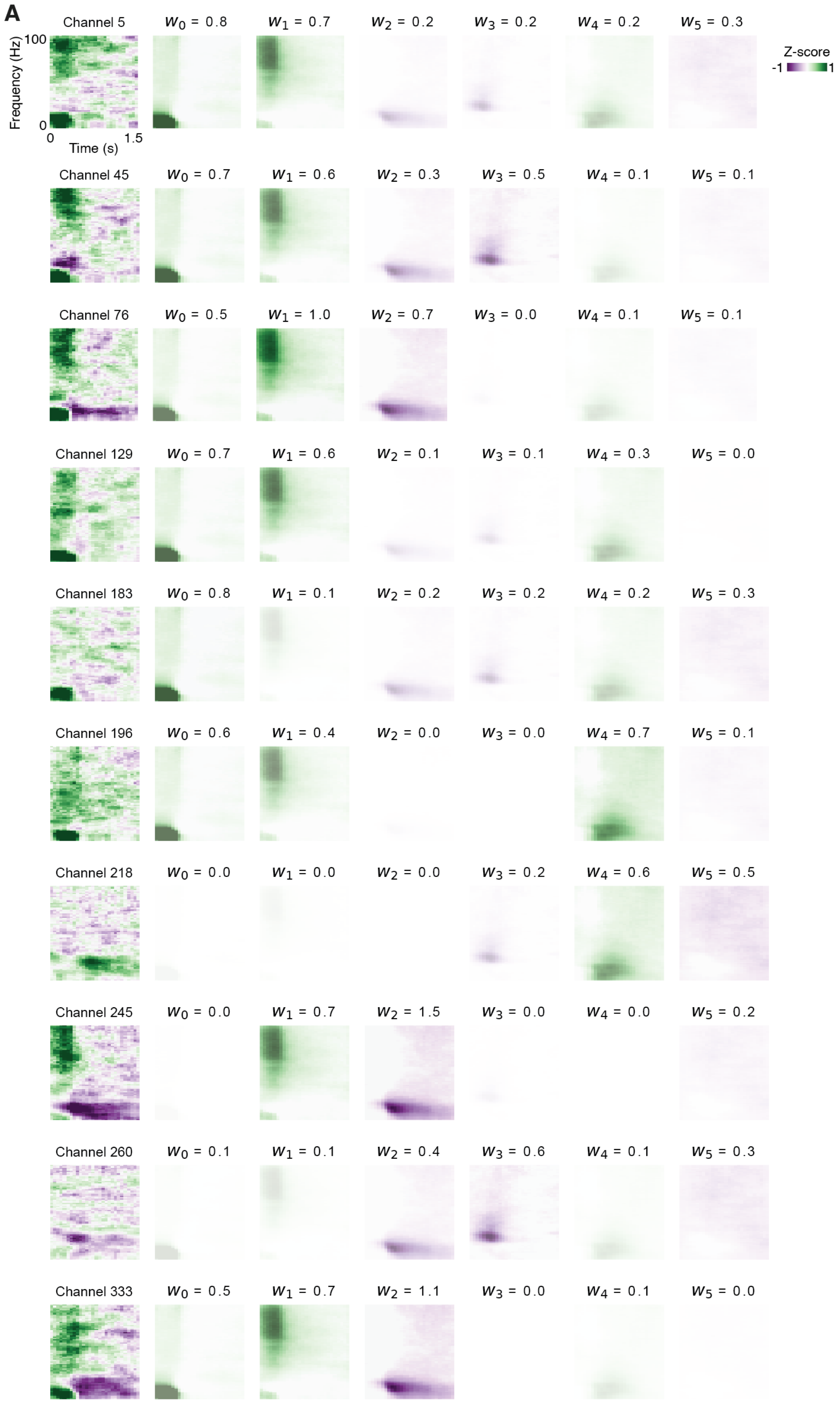

Fig. S18. Additional detail on human puff-triggered spectrogram factorization by channel. (A) Visualization of factor loadings for a set of example channels. Left column, the spectrogram of the channel (averaged across trials). Right columns, the factors and their loadings for that channel (denoted w_{factor}), with alpha transparency of the factor set according to loading.

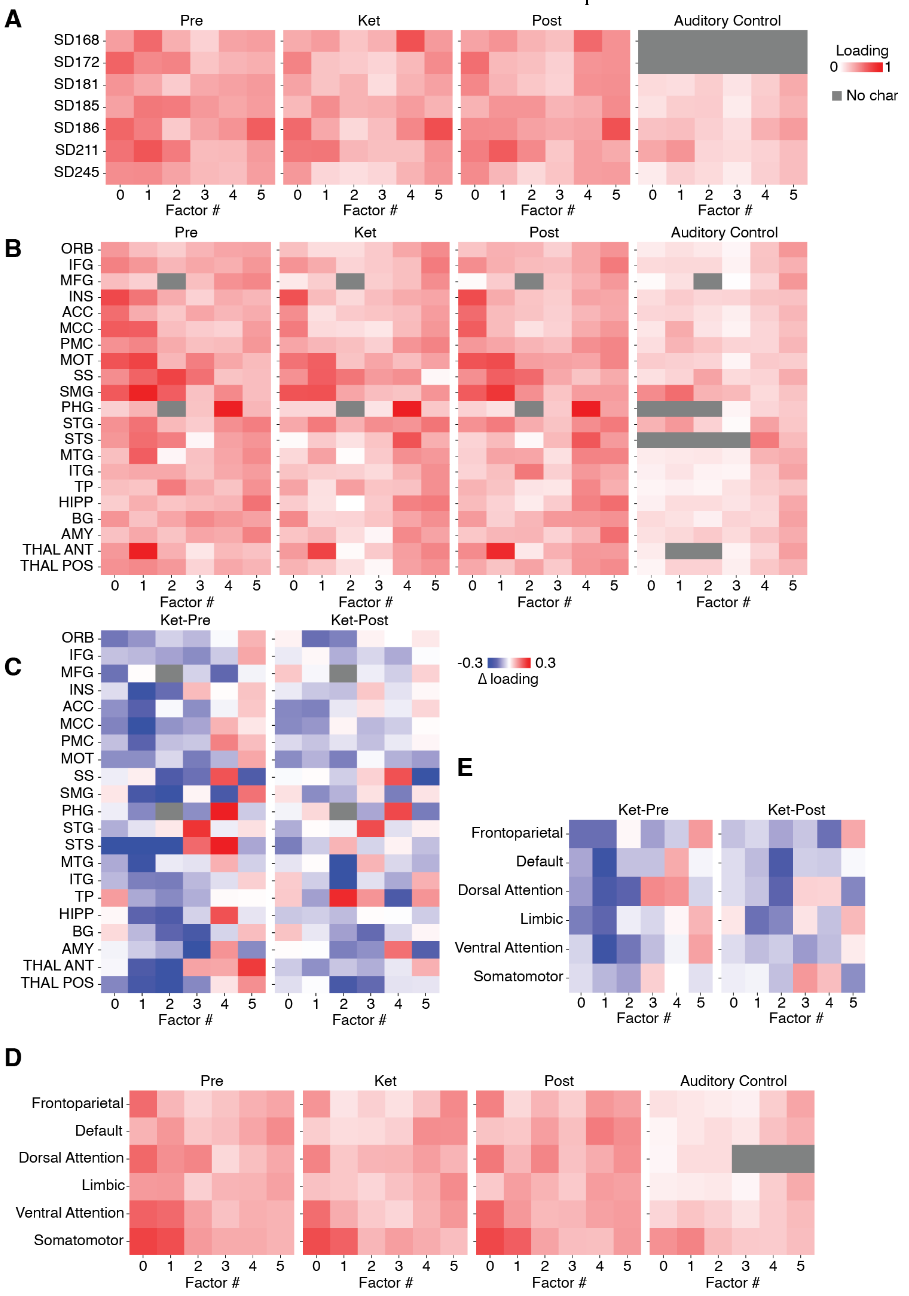

Fig. S19. Anatomical loading of each factor by condition. (A) Loading of each factor, averaged across channels within each subject, during preinfusion and infusion, indicating that most factors have substantial loading on multiple subjects. (B) Loading of each factor, averaged across channels within each region, during preinfusion, ketamine infusion, postinfusion, and auditory control conditions. The auditory control exhibits substantially lower loading overall. (C) Change in loading by region during infusion, compared with preinfusion and postinfusion. (D) Loading of each factor, averaged across channels within each Yeo7 resting state networks during each condition. (E) Change in loading by Yeo7 network during infusion relative to preinfusion and postinfusion.

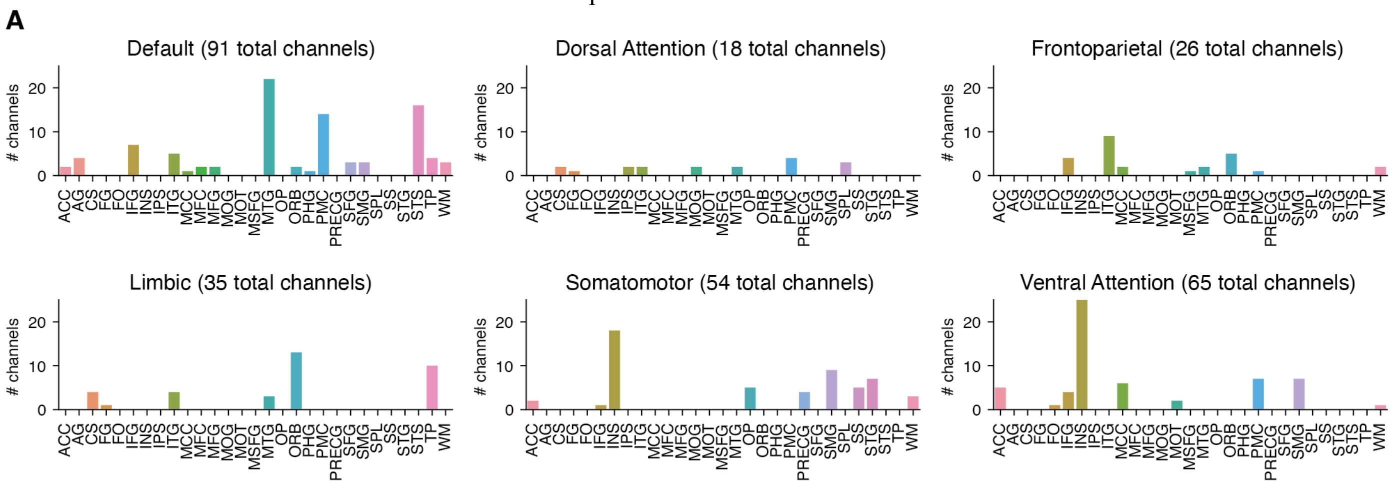

Fig. S20. Yeo7 human anatomical mapping. (A) Number of channels by region assigned to each Yeo7 network. Note that contribution to the somatomotor network is primarily composed of channels from insula.

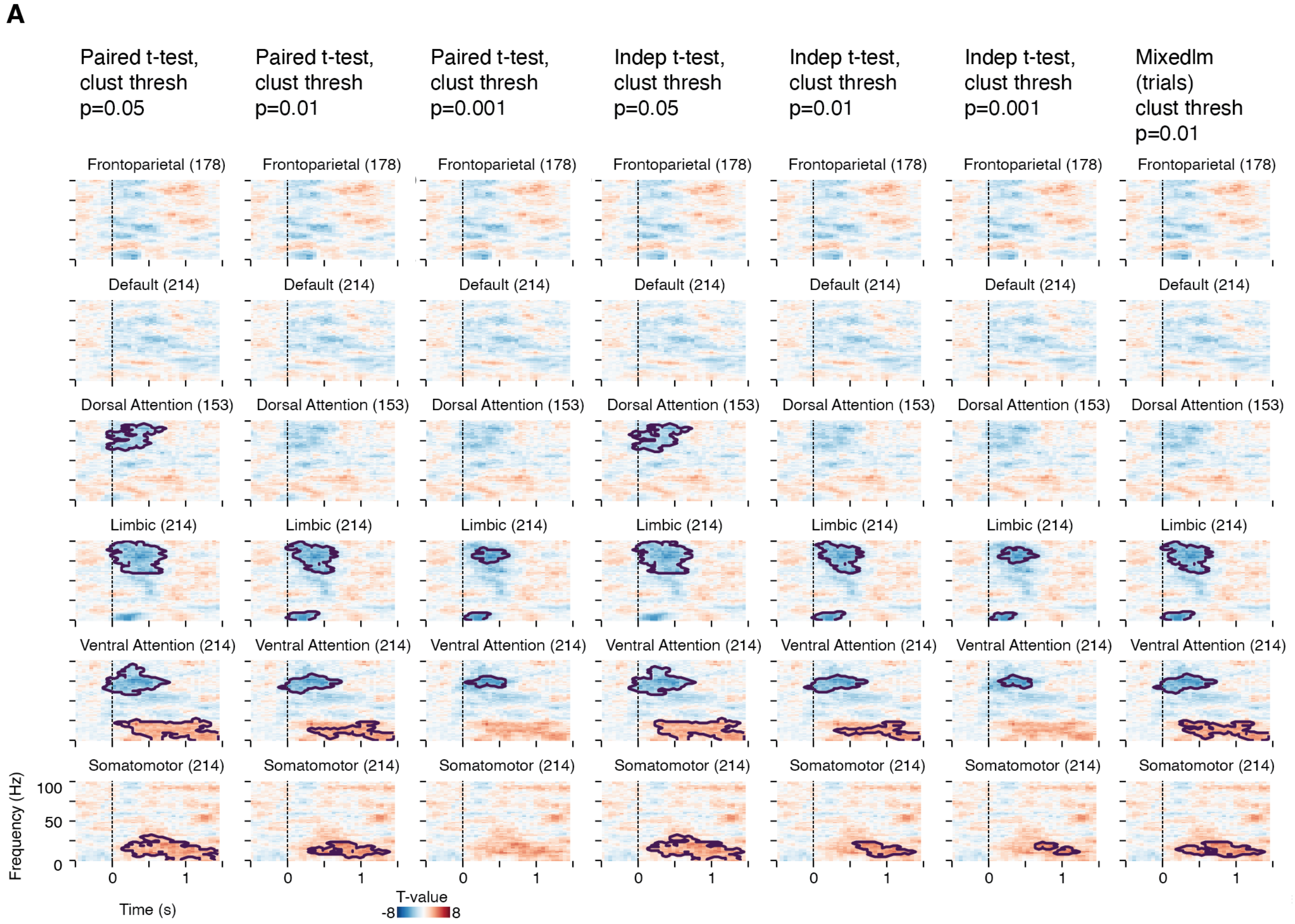

Fig. S21. More detail on permutation cluster test by human Yeo7 networks. (A) Significant changes (infusion – preinfusion) by Yeo7 resting state network, comparing different permutation cluster test parameters: cluster threshold p-value, and statistical test for comparing conditions. These results indicate that the significant clusters are robust to different permutation cluster hyperparameters. Vertical line, air puff onset.

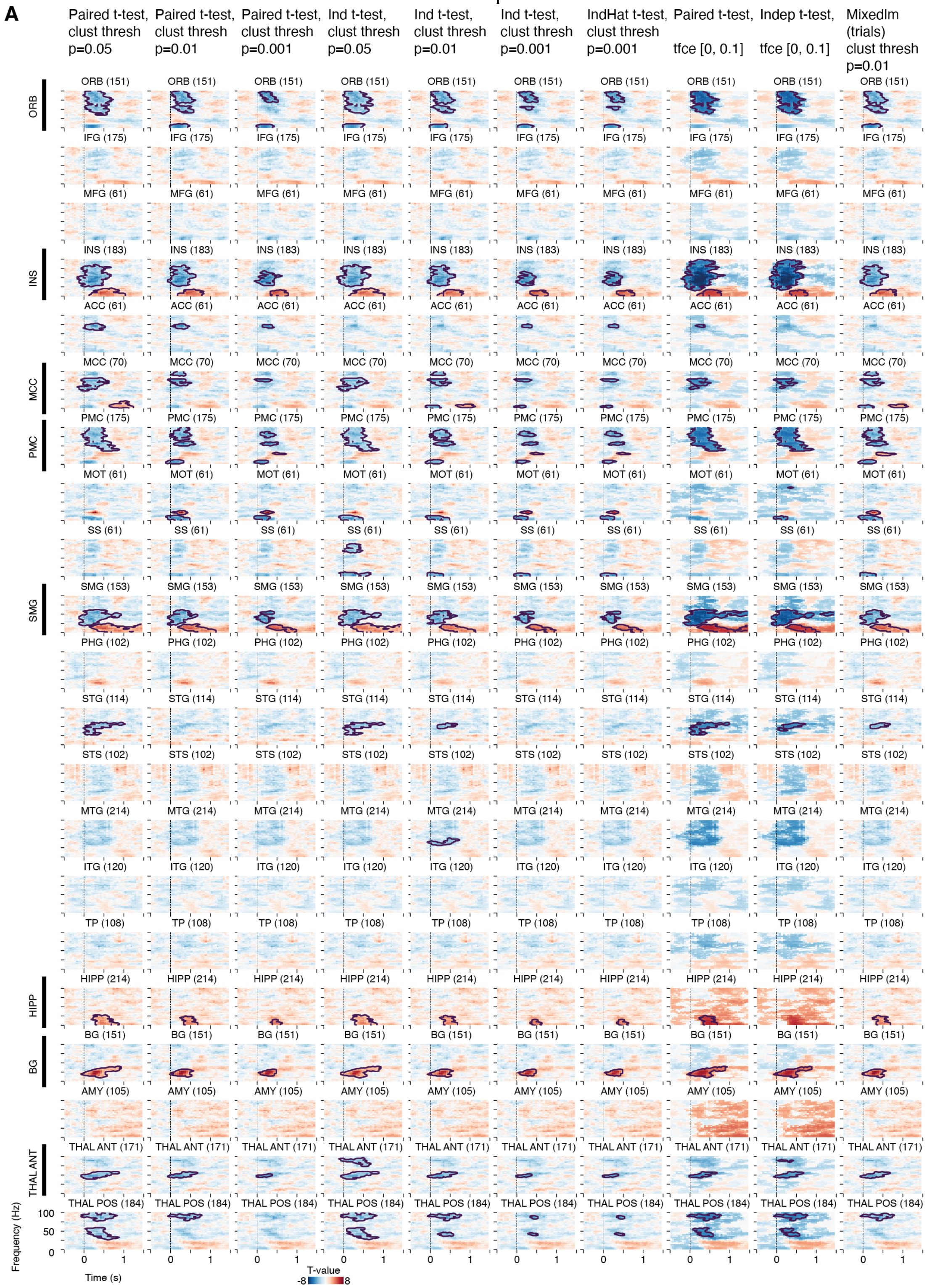

Fig. S22. More detail on permutation cluster test by human brain region. (A) Significant changes (infusion – preinfusion) by region, comparing different permutation cluster test parameters: cluster threshold p-value (0.05, 0.01, 0.001, and threshold-free cluster enhancement), and statistical test for comparing conditions (paired t-test, independent t-test, independent t-test with hat variance correction, and mixed-effects linear model across trials grouped by subject with paired t-test). Regions that exhibit significant changes robust to all hyperparameter settings are indicated at left. Vertical line, air puff onset.

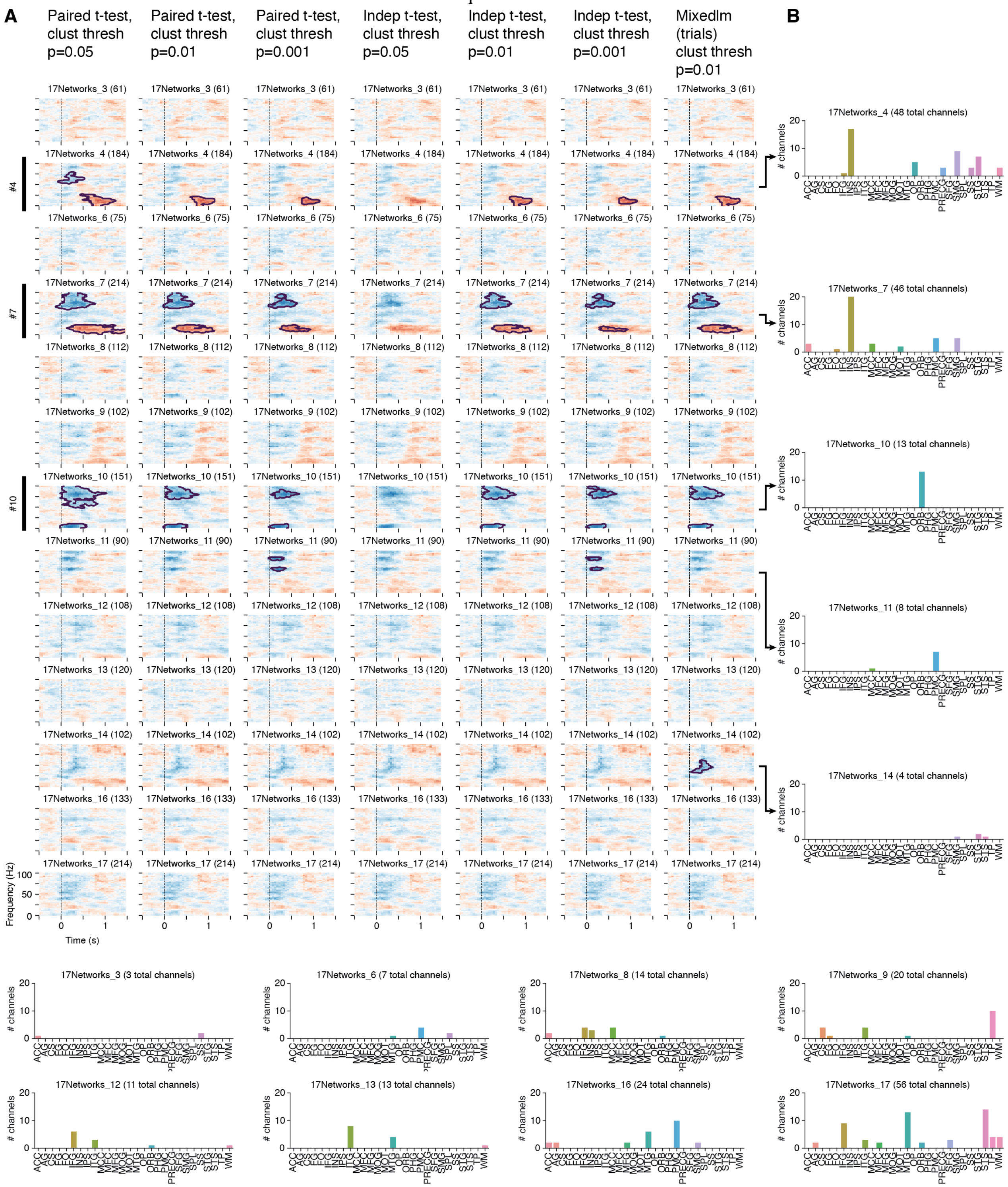

Fig. S23. Permutation cluster test by human Yeo17 network. A finer-grained 17-network resting-state atlas. (A) Significant changes (infusion – preinfusion) for each Yeo17 resting state network, comparing different permutation cluster test parameters: cluster threshold p-value, and statistical test for comparing conditions. Vertical lines, air puff onset. (B) Number of channels by region assigned to each Yeo17 network. Networks 4 & 7 (primarily insular), and 10 (primarily orbitofrontal) exhibited significant changes that were robust to hyperparameter settings. This analysis yielded similar results as the coarser-grained 7-network resting-state atlas, and further highlighted significant changes in orbitofrontal cortex (ORB) and insular cortex (INS), as well as supramarginal gyrus (SMG).

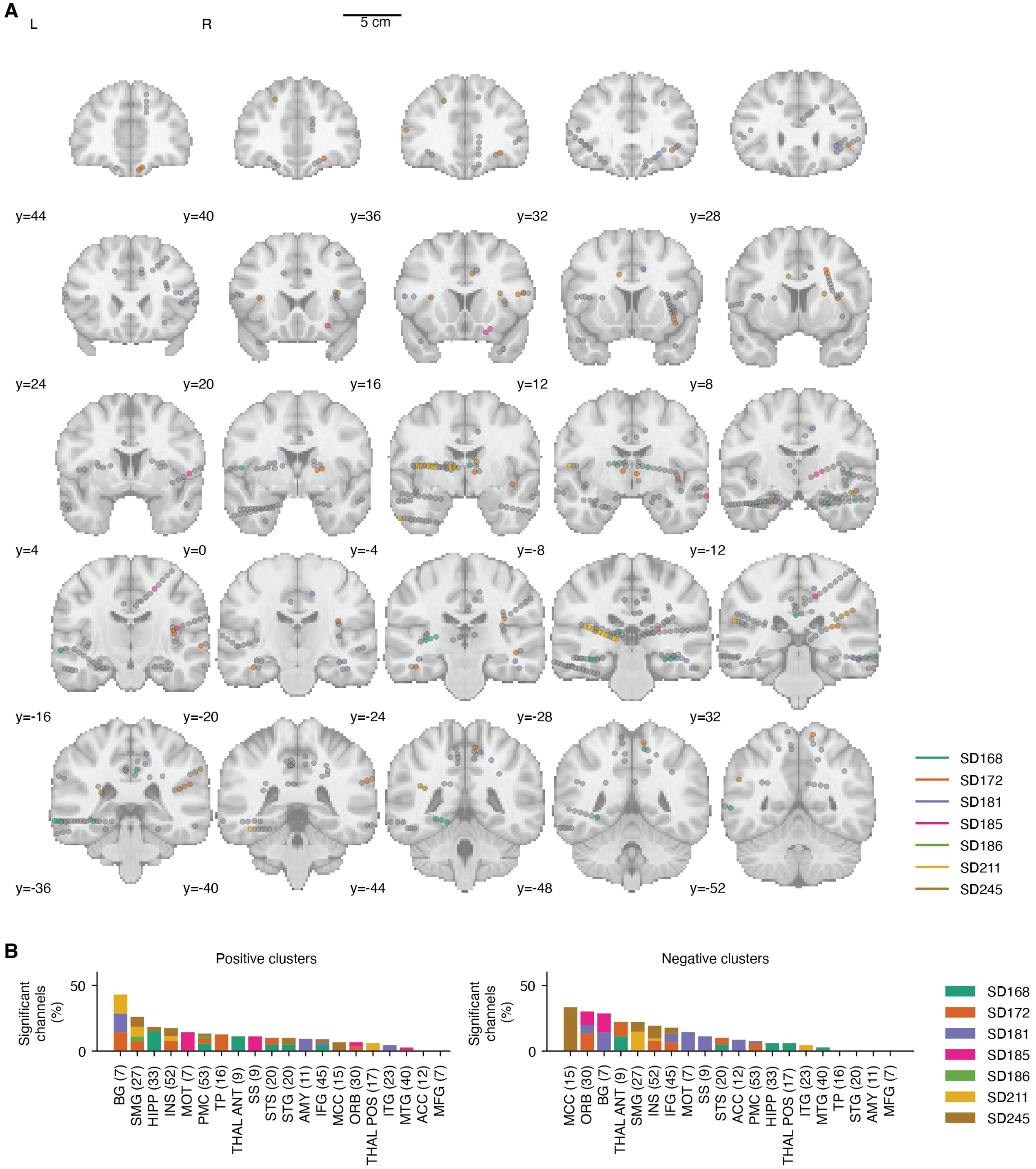

Fig. S24. Permutation cluster test, by individual human channel. (A) Location of channels with a significantly changing spectrotemporal cluster (infusion – preinfusion). (B) Percent of channels, by region and subject, that have a cluster of significantly increasing (positive) activation or significantly decreasing (negative) activation. Total number of channels per region in parentheses. Regions with a large fraction of significant channels are generally those that exhibited robust region-level significant spectrotemporal changes as shown in Fig. 3C.

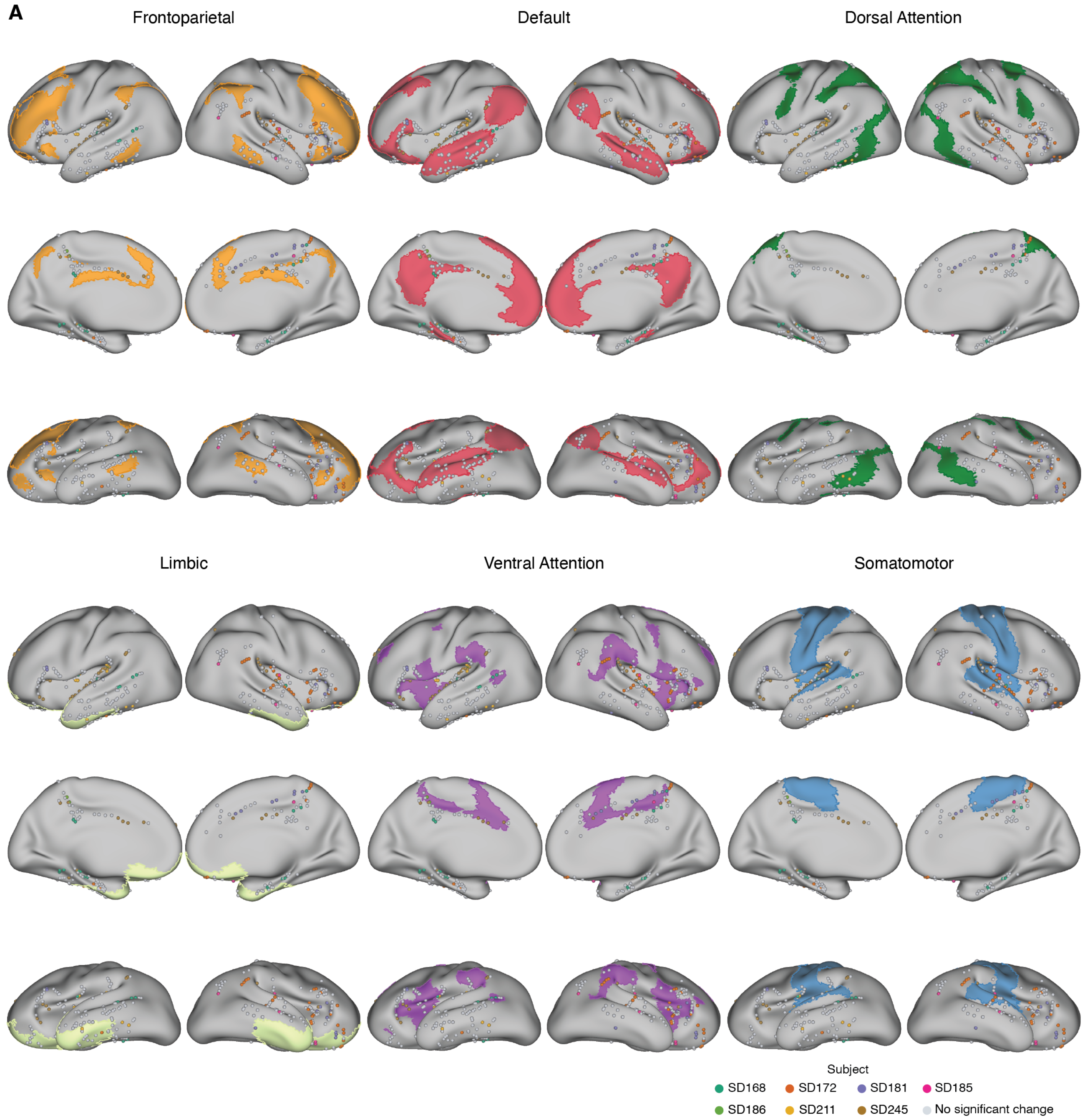

Fig. S25. Permutation cluster test by individual human channel, on Yeo7 parcellation. (A) Location of channels with a significantly changing spectrotemporal cluster (infusion – preinfusion) overlaid on Yeo7 resting-state network anatomical parcellation.

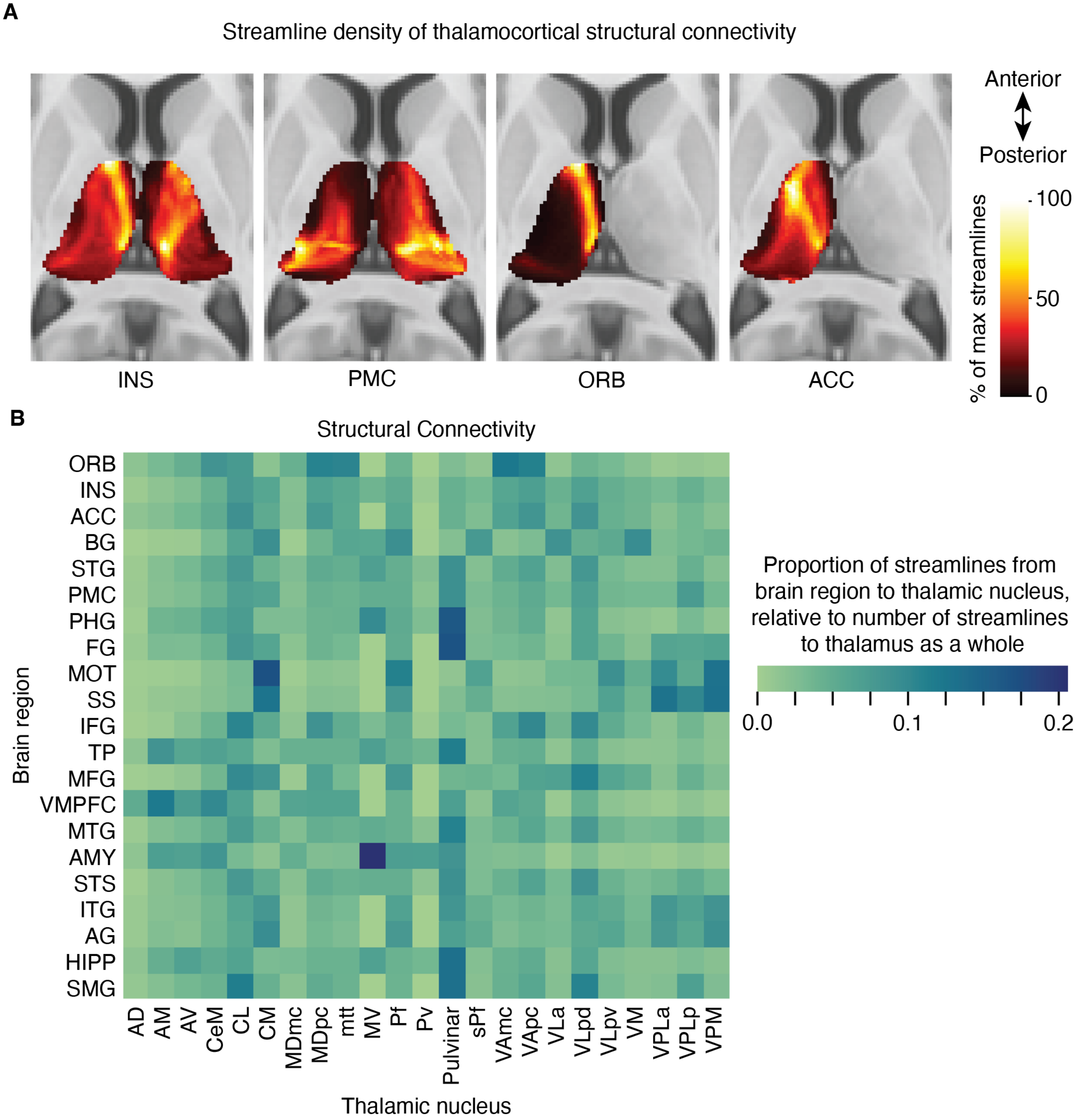

Fig. S26. Tractography-based structural connectivity to thalamus. The presence of shared stimulus-evoked spectrotemporal neural activity patterns suggests the possibility that brain regions with a ketamine-mediated change in eyepuff response directly coordinate their activity through anatomical connections. For example, certain specific regions, including several that are widely-separated across the brain, showed strikingly similar patterns of modulation–such as PMC and ORB, which exhibited same-direction modulation in distinct high and low frequency bands (Fig. 3C), resembling the ketamine-modulation response of the limbic network (Fig. 3B) associated with factor 2 (Fig. 2F, J). Unlike PMC and ORB, insula (INS) instead revealed the opposite-direction modulation in distinct high and low frequency bands (Fig. 3C) closely resembling ketamine-induced changes seen in the ventral attention network (Fig. 3B) associated with factors 2 and 3 (Fig. 2F, J). One possible mechanism for coordination of cortical region activity could be through shared thalamic connectivity. To investigate this, we used diffusion tractography to assess structural connectivity between thalamus and seed locations corresponding to patient contact locations. (A) Density of streamlines connecting thalamus to seeds placed on patient contact

locations in insular, posteromedial, orbitofrontal, and anterior cingulate cortices (Max Intensity Projection). INS and ORB exhibit stronger connectivity to anterior thalamus, whereas PMC exhibits stronger connectivity to posterior thalamus. ORB also exhibits some connectivity to posterior thalamus. This suggests that the strikingly similar effect of ketamine on PMC (exhibiting more robust connectivity to posterolateral thalamic structures) and ORB (with distinct anterior thalamic connectivity preference), both in terms of resting spectrotemporal profile (fig. S15 G-I) and eyepuff-evoked dynamics (Fig. 3C), may arise through mechanisms beyond shared thalamic connectivity. Alternative possibilities for coordination among these regions include direct ORB-PMC connectivity, intra-thalamic connectivity, finer-scale connectivity with thalamus not resolvable by diffusion tractography, and indirect coordination via other interconnected regions. (B) Summary of structural connectivity from non-thalamic brain regions to each thalamic nucleus.

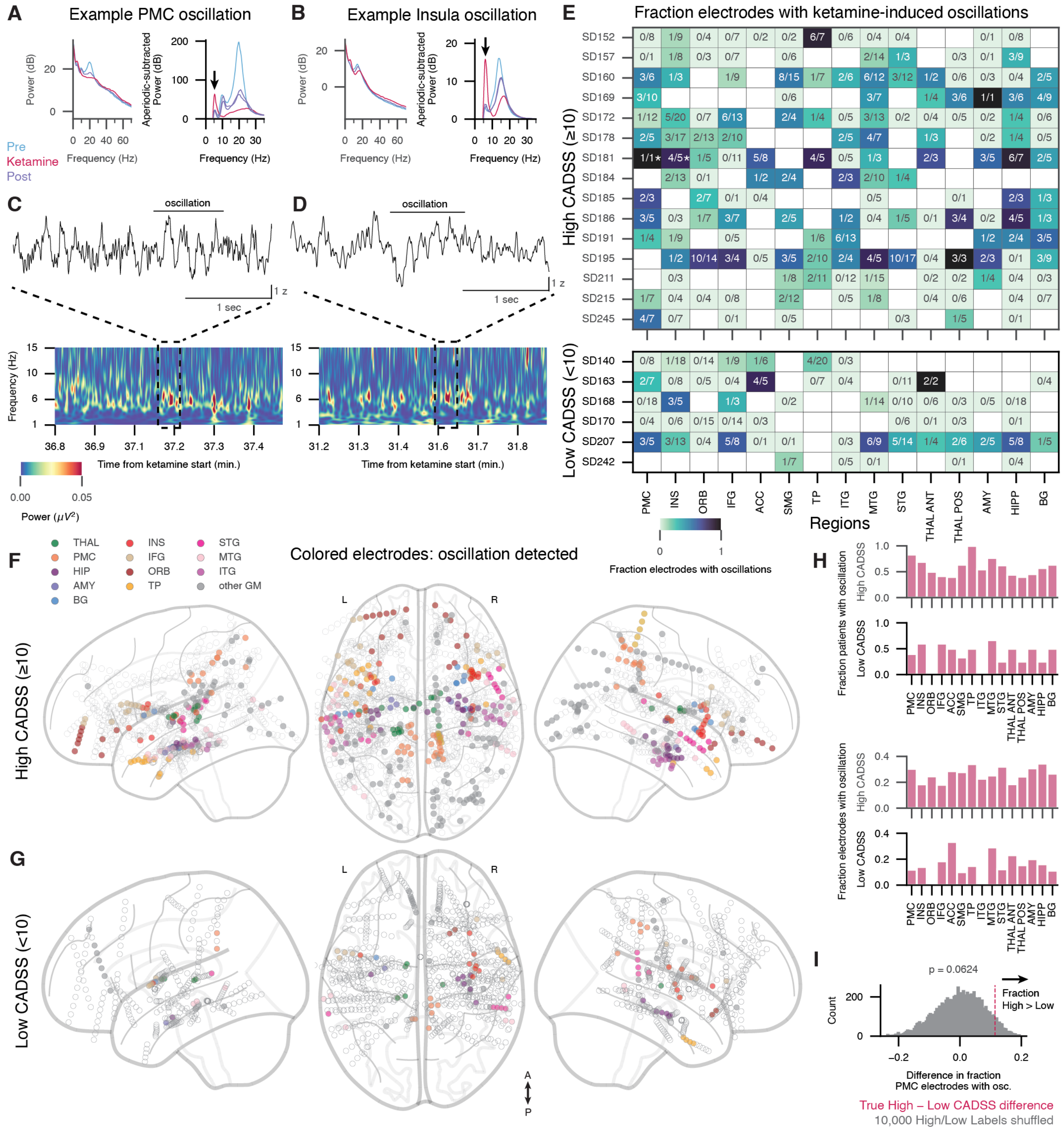

Fig. S27. Ketamine induces slow oscillations broadly across human brain regions. We previously reported that ketamine induces slow oscillations in mice and that seizure aura-associated dissociation is associated with slow oscillations in human PMC (*28*); subsequent iEEG investigation in humans also found a low frequency spectral power increase in PMC during rapidly-administered ketamine (0.5 mg/kg over 14 minutes, a ~3x faster infusion rate than our continuous infusion) (*38*). Here we investigate ketamine-induced band-limited, low-frequency oscillations (*81*) in our human participants. All oscillations in this figure refer to sub-10 Hz slow oscillations. (A) Left: Power spectral density (PSD) demonstrating a ketamine-induced slow oscillation for an example posteromedial cortex (PMC) bipolar contact, from SD181. Right: PSD with 1/f aperiodic spectrum subtracted, highlighting band-limited oscillatory peaks. Black arrow indicates a sub-10 Hz slow oscillation. (B) Same as (A), but for an example insula bipolar contact, from SD181. (C) Top: Bipolar voltage trace of PMC

contact in (A), showing example slow oscillation (black line). Bottom: spectrogram of PMC contact. (D) Same as (C), but for example insula bipolar contact in (B). (E) Distribution of ketamine-induced slow oscillations across regions and patients, subdivided by high and low CADSS score. Ketamine-induced oscillations are identified when their amplitude and/or frequency are different compared to pre- and post-infusion. (F) Spatial arrangement of ketamine-induced slow oscillations for high CADSS subjects (CADSS >= 10). Uncolored channels did not have detected oscillations. (G) Same as (F), for low CADSS subjects. (H) Comparison across high vs. low CADSS subjects of channel fraction with slow oscillations per region (top), and of patient fraction with > 0 slow oscillations (bottom). (I) Comparison of measured difference between high and low CADSS subject's total channel fraction with slow oscillations versus a null distribution in which high and low subject labels are randomly permuted 10,000 times. Despite heterogeneity across subjects (as expected from diversity in patient-specific responses to ketamine, as well as anatomical sampling differences dictated by clinically-guided sEEG electrode placement) (fig. S27 E, F), all subjects with strong dissociation (CADSS score >10) exhibited low-frequency (<10 Hz) oscillations induced by ketamine (most notably in PMC and TP; we also observed that subjects with high CADSS scores tended to exhibit a higher proportion of channels recording these low-frequency oscillations compared to subjects with low CADSS scores: fig. S27 E–I).

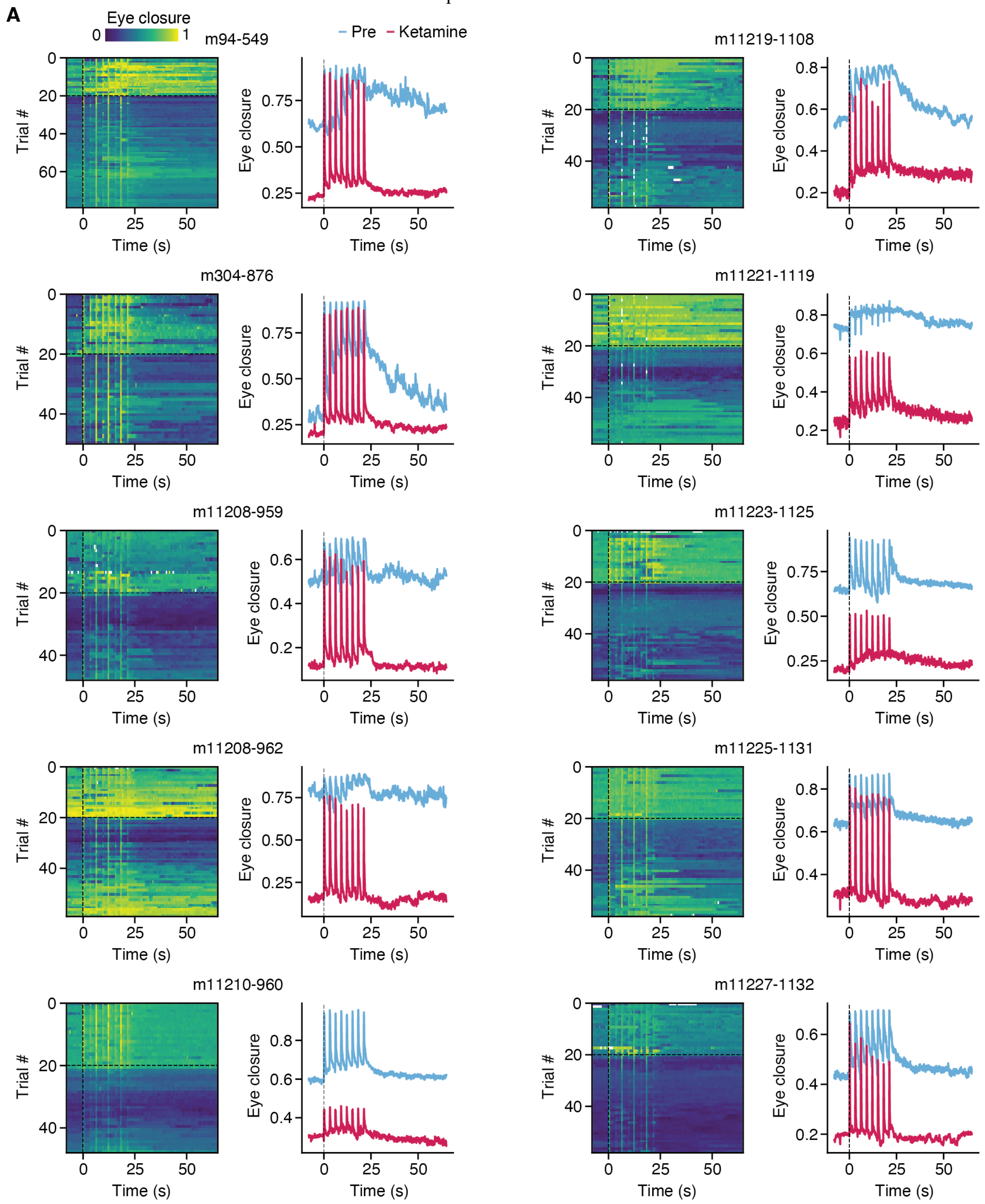

Fig. S28. Mouse eye closure during Neuropixels recording sessions. (A) Left, eye closure on individual trials (each consisting of eight puffs); Right, mean across trials during preinfusion and ketamine conditions. Bolus ketamine infusion occurs at trial 20.

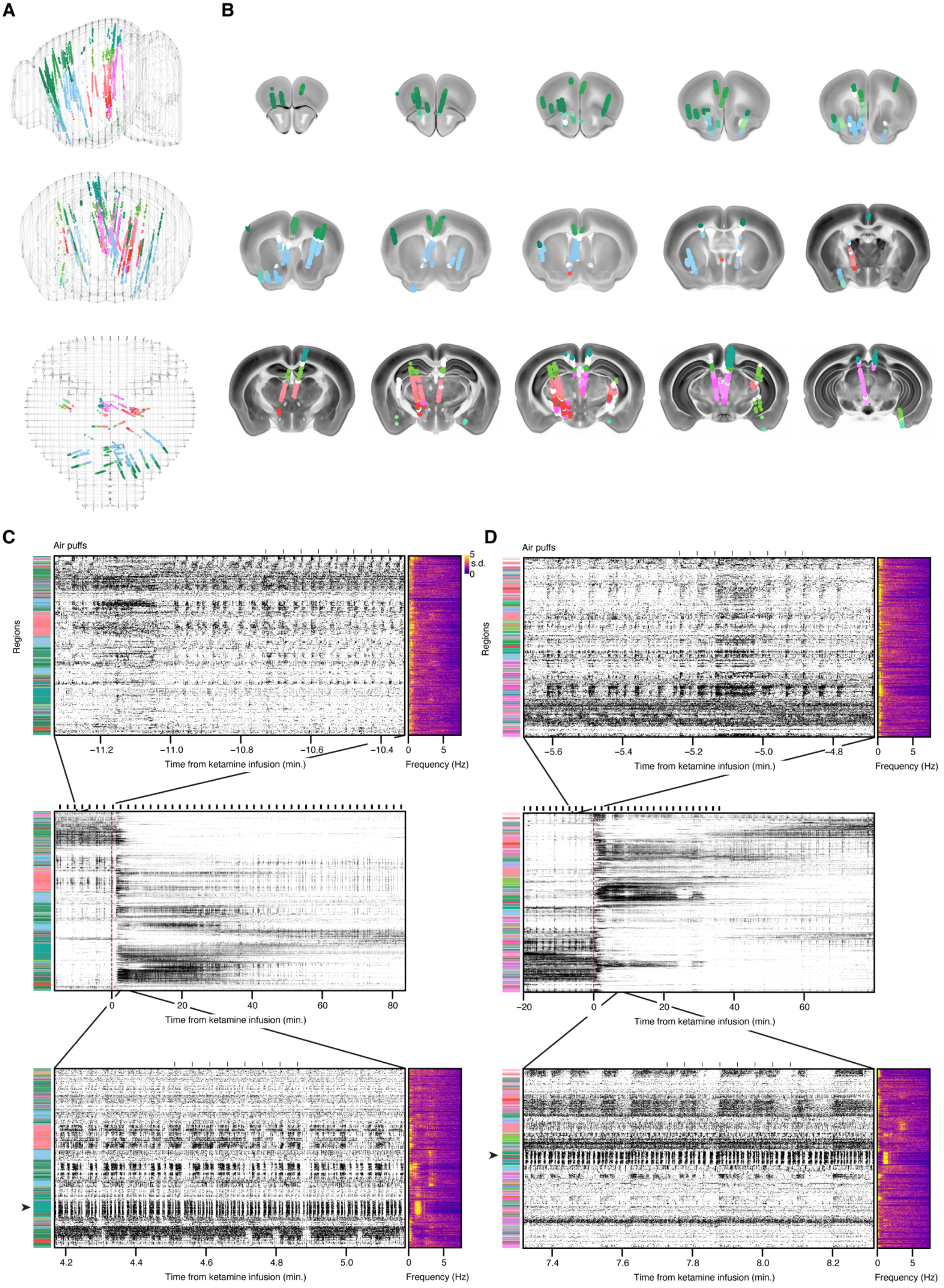
A
B
C
D
Air puffs
Regions
5
s.d.
0
−11.2
−11.0
−10.8
−10.6
−10.4
Time from ketamine infusion (min.)
Frequency (Hz)
−5.6
−5.4
−5.2
−5.0
−4.8
0
20
40
60
80
−20
4.2
4.4
4.6
4.8
5.0
7.4
7.6
7.8
8.0
8.2

Fig. S29. Additional details on mouse Neuropixels recording during ketamine infusion. (A, B) Anatomical location of all recorded units. (C, D) Example recording. Red dashed line indicates time of bolus ketamine infusion. Units ordered by rastermap clustering algorithm. Top, preinfusion. Bottom, after infusion. Left, region labels, colored as in (B). Black arrow indicates retrosplenial cortex. Right, power spectral density of each channel. Retrosplenial cortex exhibits particularly strong rhythmic activity.

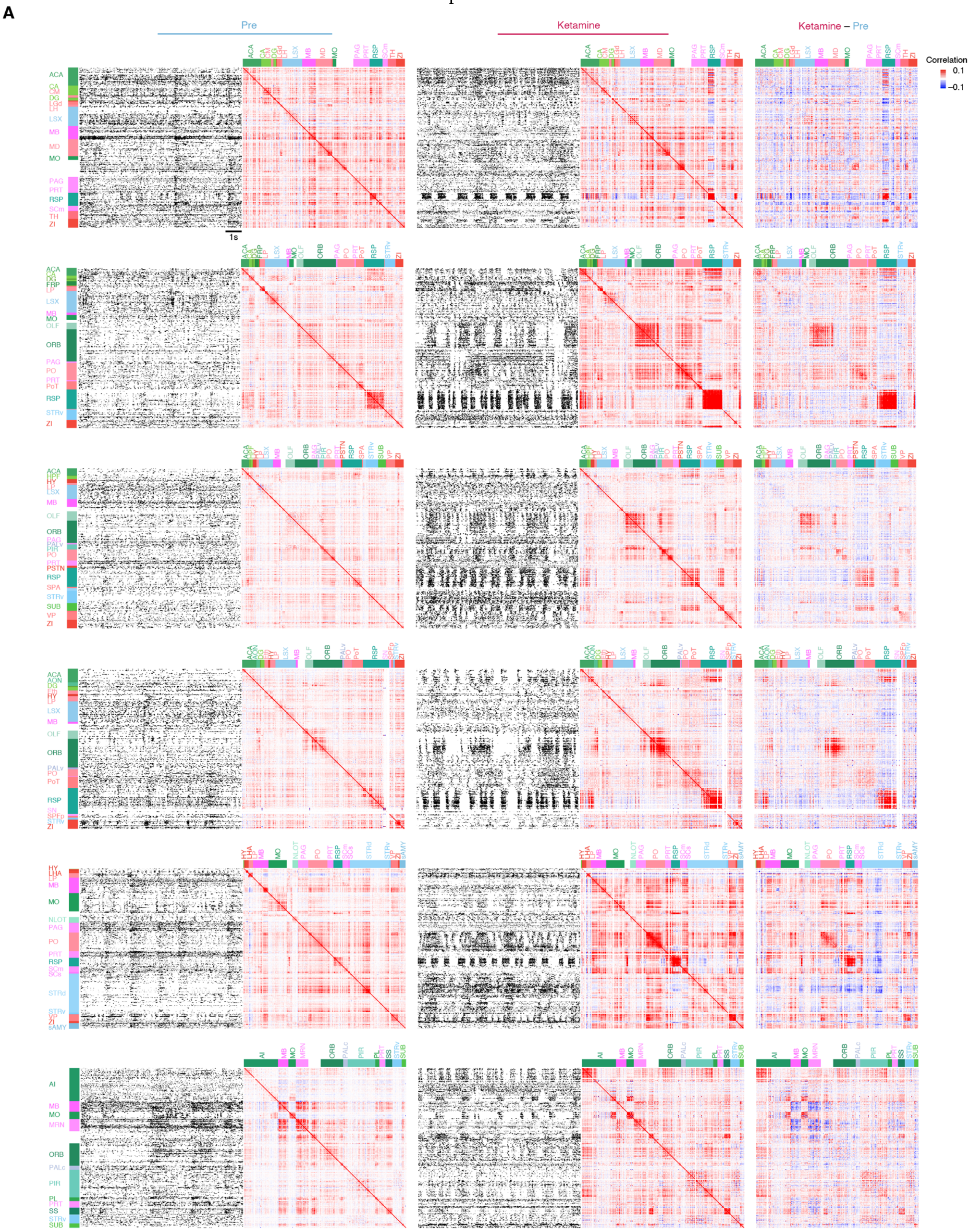

Fig. S30. Change of mouse neural activity correlation structure with ketamine. (A) Left: preinfusion spiking activity of simultaneously recorded units during example ten second window, and single unit correlation matrix across 10 min window. Middle: same, but following bolus ketamine infusion. Right: Change of correlation matrix (ketamine – preinfusion).

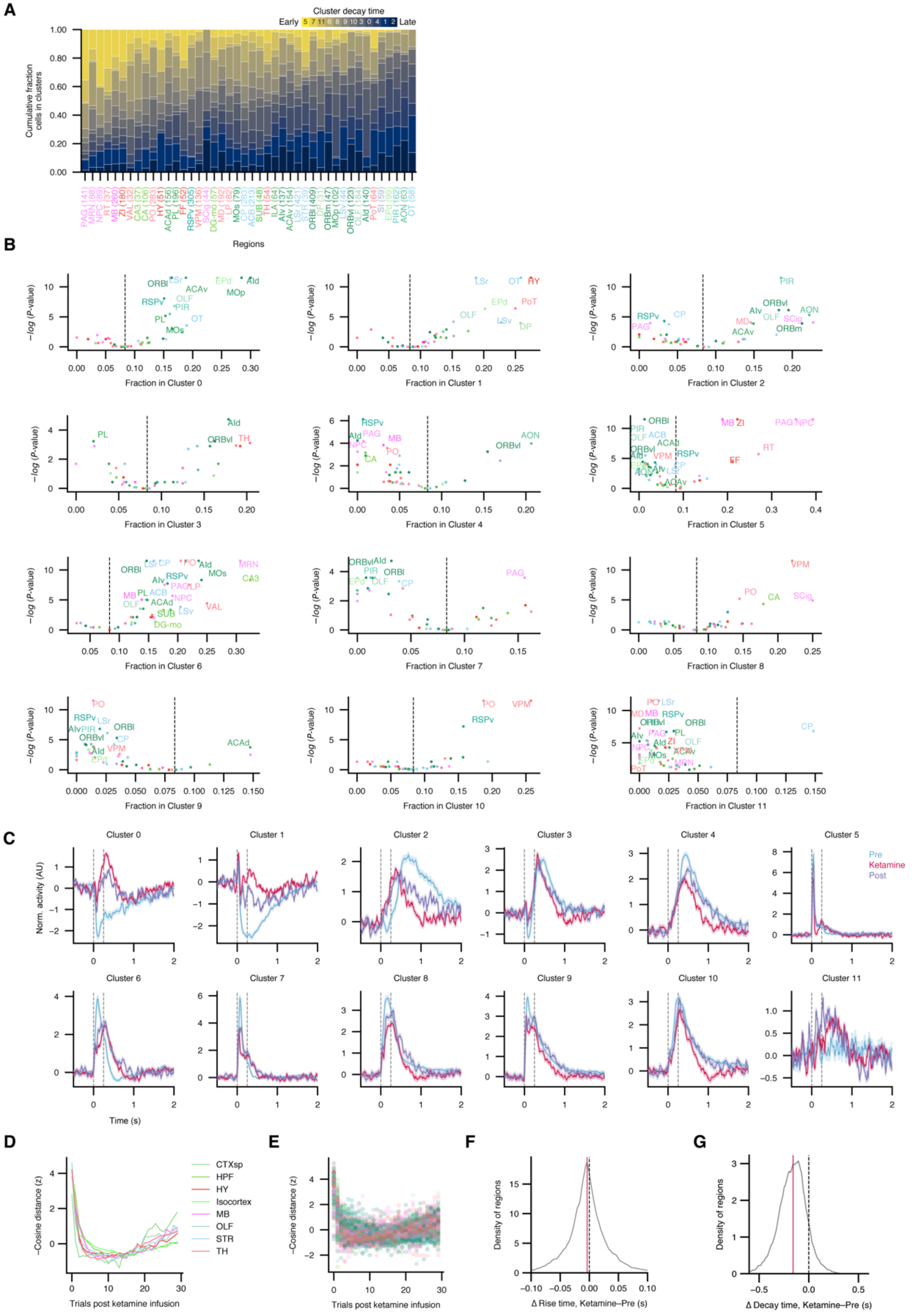
A
Cluster decay time
Early
Late
Cumulative fraction cells in clusters
Regions
B
−log (P-value)
Fraction in Cluster 0
Fraction in Cluster 1
Fraction in Cluster 2
Fraction in Cluster 3
Fraction in Cluster 4
Fraction in Cluster 5
Fraction in Cluster 6
Fraction in Cluster 7
Fraction in Cluster 8
Fraction in Cluster 9
Fraction in Cluster 10
Fraction in Cluster 11
C
Cluster 0
Cluster 1
Cluster 2
Cluster 3
Cluster 4
Cluster 5
Cluster 6
Cluster 7
Cluster 8
Cluster 9
Cluster 10
Cluster 11
Pre
Ketamine
Post
Norm. activity (AU)
Time (s)
D
E
F
G
CTXsp
HPF
HY
Isocortex
MB
OLF
STR
TH
−Cosine distance (z)
Trials post ketamine infusion
Density of regions
Δ Rise time, Ketamine−Pre (s)
Δ Decay time, Ketamine−Pre (s)

Fig. S31. Additional detail on mouse single unit activity and peri-puff changes on ketamine. (A) Cumulative fraction of cells in clusters, by region. Clusters are colored by the rank order time for average activity across all recorded cells in a cluster to decay to 0.25x the peak value post-puff. Clusters are numbered according to their order from top to bottom in Fig. 4D. Regions sorted in ascending order by a weighted average of cluster decay times, with weights equal to the fraction of cells in a cluster in a given region. Region acronyms colored by the Allen Brain Atlas colormap. (B) Representation of regions in each cluster. (C) Mean cluster activity (normalized within each condition) for all clusters. Mean ± 95% CI. (D, E) Similarity (negative z-scored cosine distance) between preinfusion cluster activity and activity on each trial following bolus ketamine infusion. Similarity averaged across mice within coarse anatomical grouping (D) and finer anatomical grouping (E, each region shown as individual dot for each time bin). Bootstrapped distribution of changes in peri-puff rise times (F) and decay times (G) across regions. Red line, median. See Supplementary table 3 for information on statistical analyses and sample sizes.

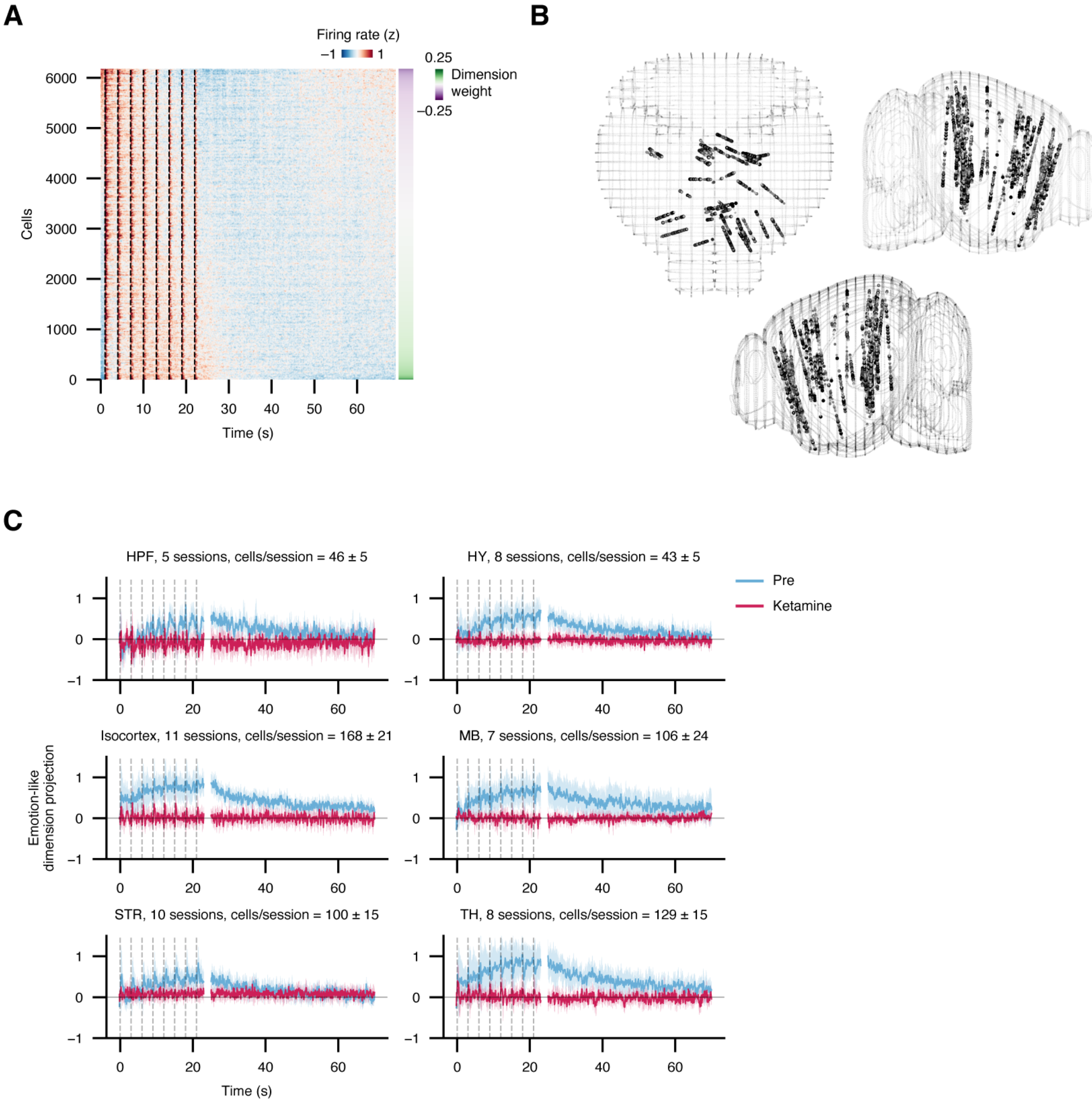

Fig. S32. Additional detail on emotion-like neural dimension. (A) Trial-averaged single cell responses, where a trial consists of a sequence of eight puffs. Cells sorted based on their weight in the emotion-like neural dimension, indicated on the right. (B) Spatial distribution of emotion-like neural dimension weights, showing broad anatomical distribution. (C) Emotion-like neural dimension projection computed by high-level brain area. Mean ± 95% CI. All high-level brain areas (where there are sufficient numbers of simultaneously recorded neurons to perform population analyses) exhibit some degree of accumulating persistent dynamics during preinfusion that is abolished by ketamine.

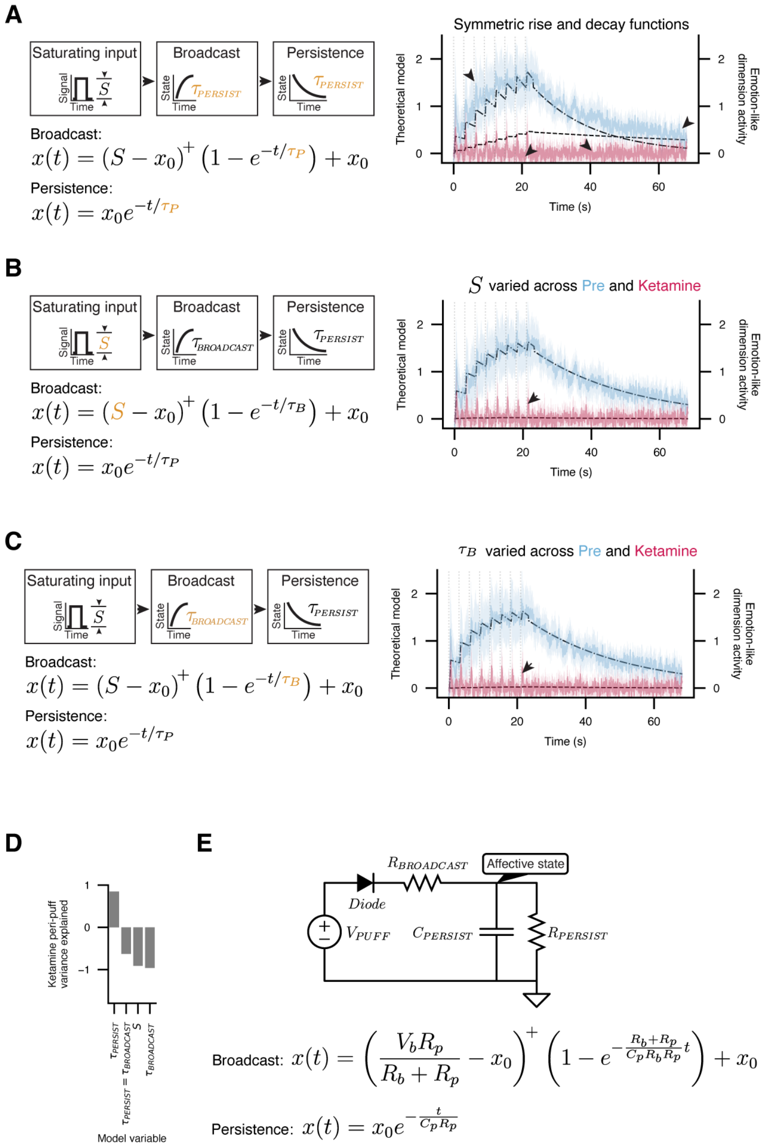

Fig. S33. Additional detail on first order system model of emotion-like state. (A) Alternative symmetric model in which there is only a single timescale across the broadcast and persistence phases. Forcing the rise and decay rates to be symmetric leads to an inability to accurately fit the preinfusion or infusion states. The leftmost and rightmost arrows indicates a poor preinfusion fit, and the middle two arrows indicate a poor infusion fit. (B) Alternative model in which the magnitude of the input signal S is varied across conditions instead of $t_{PERSIST}$. This leads to an inability to fit the transient peri-puff increases in emotion-like state during ketamine infusion, as indicated by the arrow. (C) A similar inability occurs in an alternative model where $t_{BROADCAST}$ is the only free parameter across conditions, as indicated by the arrow. (D) Variance explained (R^2) of the model evaluated specifically on the puff series during ketamine epoch. This suggests that a model where ketamine modulates $t_{PERSIST}$, as opposed to $t_{BROADCAST}$ or S, is a better fit to the experimental data. (E) An example of a physical system that exhibits the same first order dynamics as our model: an analog electrical circuit with passive components. The affective state is represented as the voltage at the node preceding the capacitor. The diode enforces asymmetry between a broadcast phase when V_{PUFF} is high, and a persistence phase when V_{PUFF} is low. The capacitor serves to store the input signal and causes the input signal to saturate. The persistence timescale is determined by $t_{PERSIST} = C_{PERSIST}R_{PERSIST}$, and the broadcast timescale is further controlled by $R_{BROADCAST}$. The dynamics were solved using Kirchoff's Current Law.

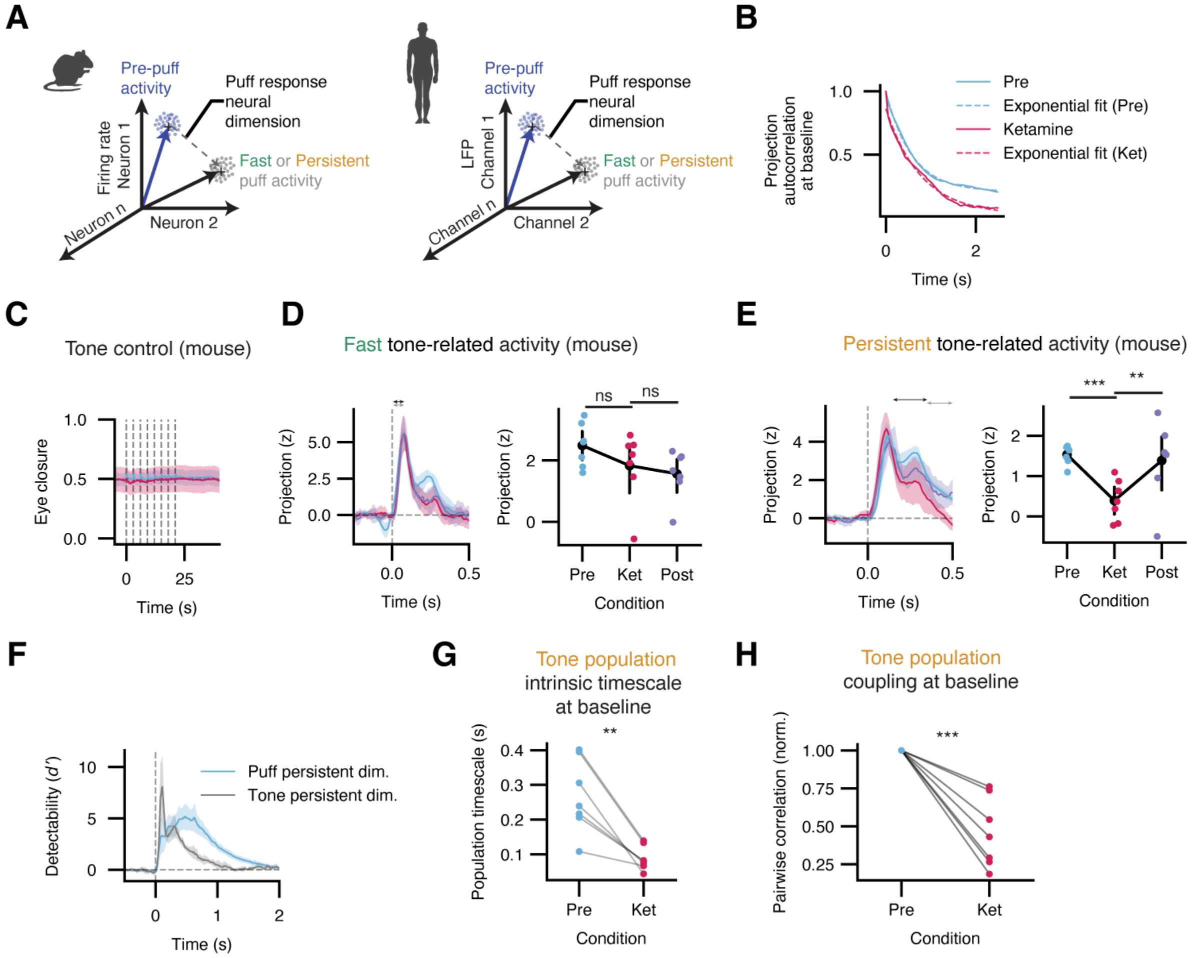

Fig. S34. Additional details on coding dimension analysis. (A) Schematic of analysis to identify neural dimensions encoding either fast or persistent puff-triggered population activity, relative to pre-puff activity. In mice, the dimension is in the space of neuron firing rates, whereas in humans it is in the space of channel LFPs. (B) Example autocorrelation of coding dimension projection during preinfusion or ketamine (solid lines), and exponential fits (dashed lines) used to compute the intrinsic timescale. Autocorrelation functions were fit to the exponential function $f(x) = a*\exp(-x/b)+c$. The ketamine autocorrelation decays more quickly than the preinfusion autocorrelation, corresponding to a reduced intrinsic timescale (parameter b, above) with ketamine. (C) Mice do not exhibit substantial eye closure in response to a sequence of auditory tones during preinfusion or ketamine (dashed lines represent onset of each 250 ms tone). (D) Fast tone-related population activity is not significantly altered by ketamine (coding dimension and mean activity computed during 0 to 70 ms following tone onset). (E) Persistent tone-related population activity is reduced by ketamine relative to preinfusion and postinfusion (coding dimension computed during 150 to 350 ms following tone onset, and mean activity quantified 350 to 500 ms following tone onset). Black arrow, coding dimension window; gray arrow, mean activity window. See Methods for detail on choice of time windows. (F) Detectability (d') of the projection of the persistent neural dimension for either eyepuff or tone. The affectively salient eyepuff elicits substantially longer persistent dynamics than the neutral tone. C–F, mean ± 95% CI. (G) Intrinsic timescale at baseline of persistent tone population is reduced during ketamine relative to preinfusion. (H) Coupling at baseline within persistent tone population is reduced during ketamine relative to preinfusion. ns, P-value ≥ 0.05. *, P-value < 0.05. **, P-value < 0.01. ***, P-value < 0.001. ****, P-value < 0.0001. See Supplementary table 3 for information on statistical analyses and sample sizes.

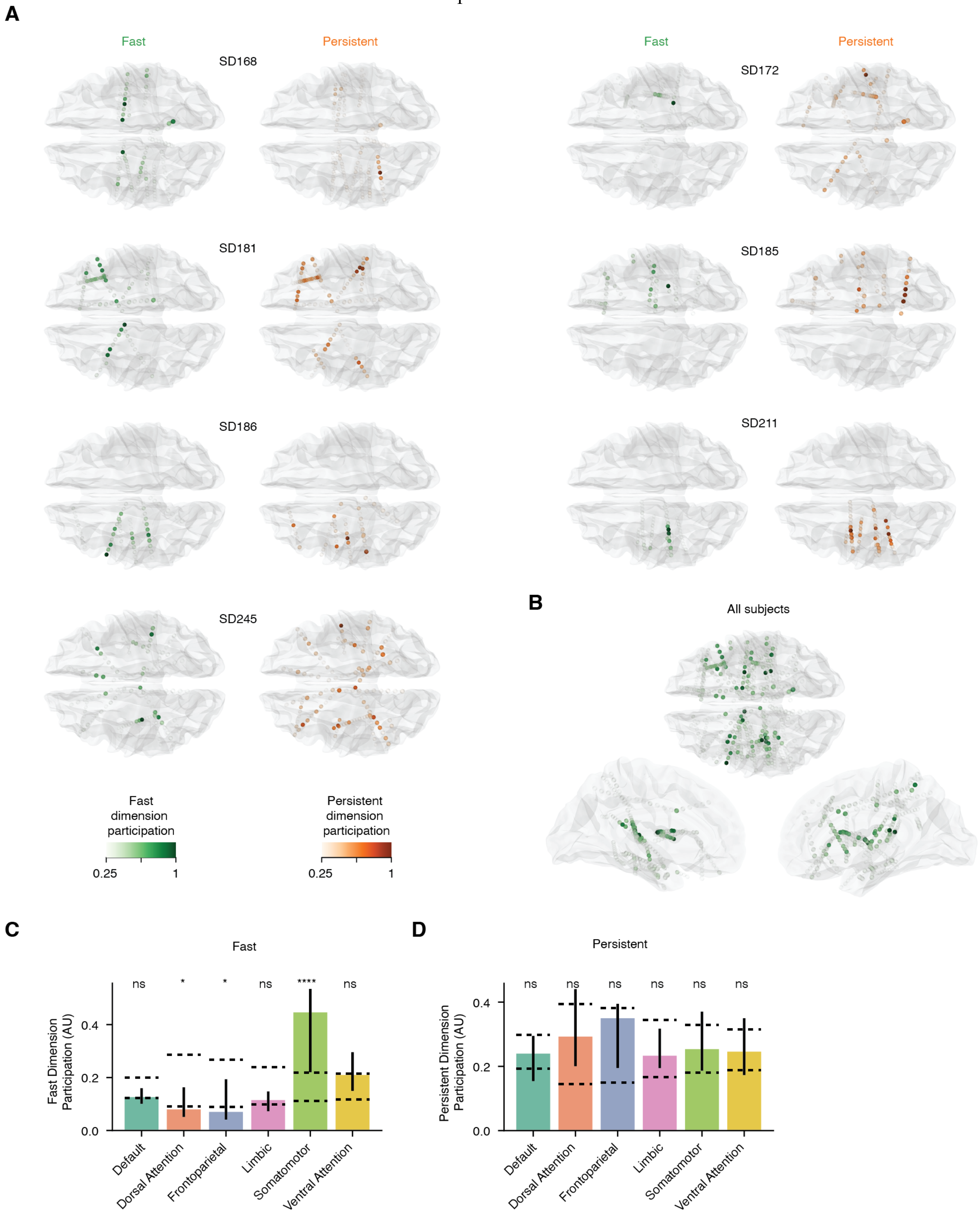

Fig. S35. Distributed human anatomical participation of puff-related coding dimensions. (A) Participation (loading) of each site for each subject for Fast and Persistent coding dimensions. (B) Sites from all subjects overlaid, for Fast coding dimension loadings. (C) Aggregate participation in Fast dimension for Yeo7 resting state networks. Participation is not uniformly distributed across networks, with somatomotor exhibiting over-participation. Dashed lines represent upper and lower thresholds signifying over- or under-participation in the Fast dimension relative to other networks (median ± 95% confidence interval, thresholds determined with bootstrap shuffles of network label, 1000 shuffles). (D) Aggregate participation in Persistent dimension for Yeo7 resting state networks. Participation is global, and uniformly distributed

across networks. ns, P-value ≥ 0.05. *, P-value < 0.05. **, P-value < 0.01. ***, P-value < 0.001. ****, P-value < 0.0001. See Supplementary table 3 for information on statistical analyses and sample sizes.

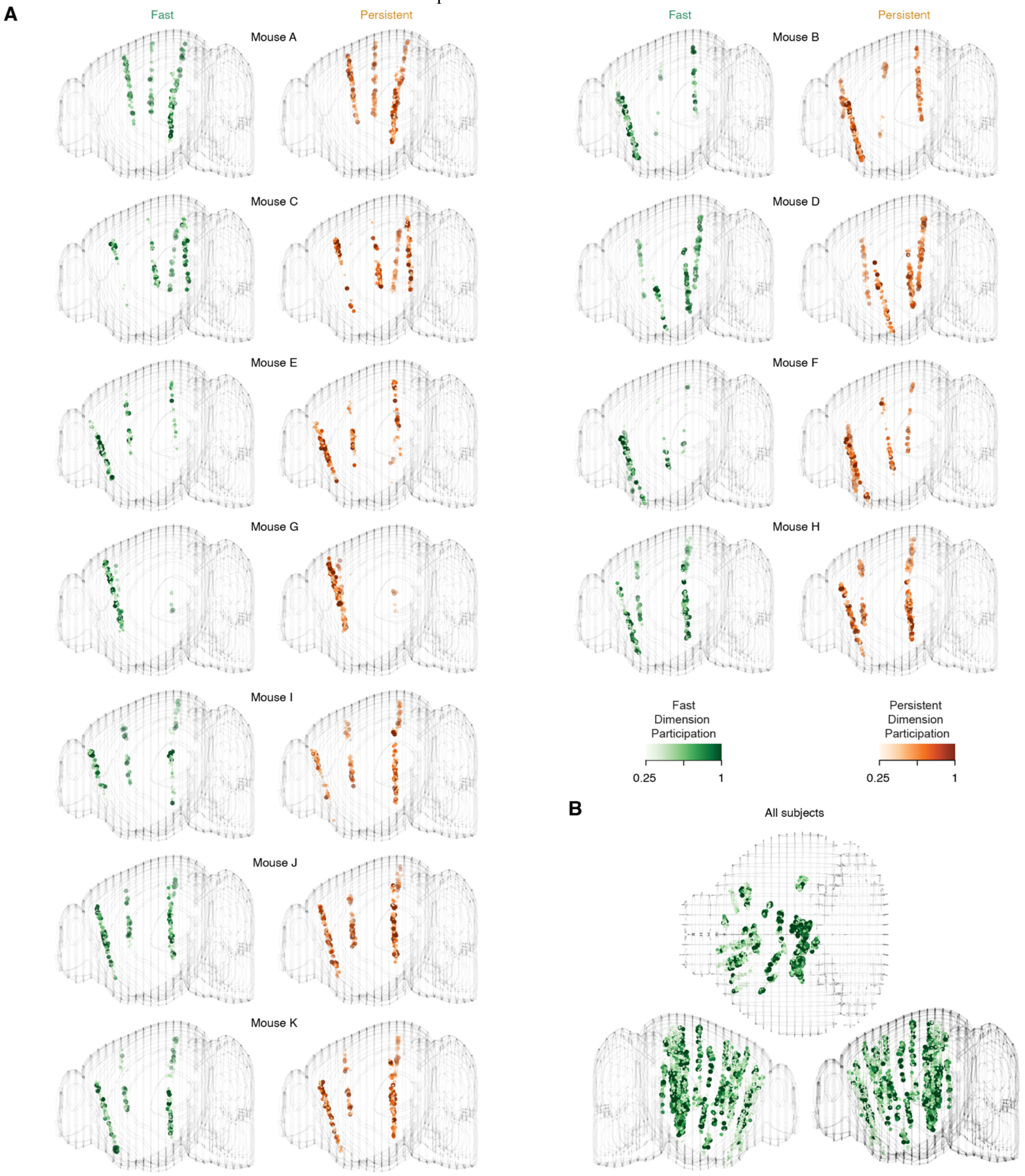

Fig. S36. Distributed mouse anatomical loading of puff-related coding dimensions. (A) Loading of each neuron for each mouse for Fast and Persistent coding dimensions. (B) Loading for all neurons from all mice for the Fast coding dimension.

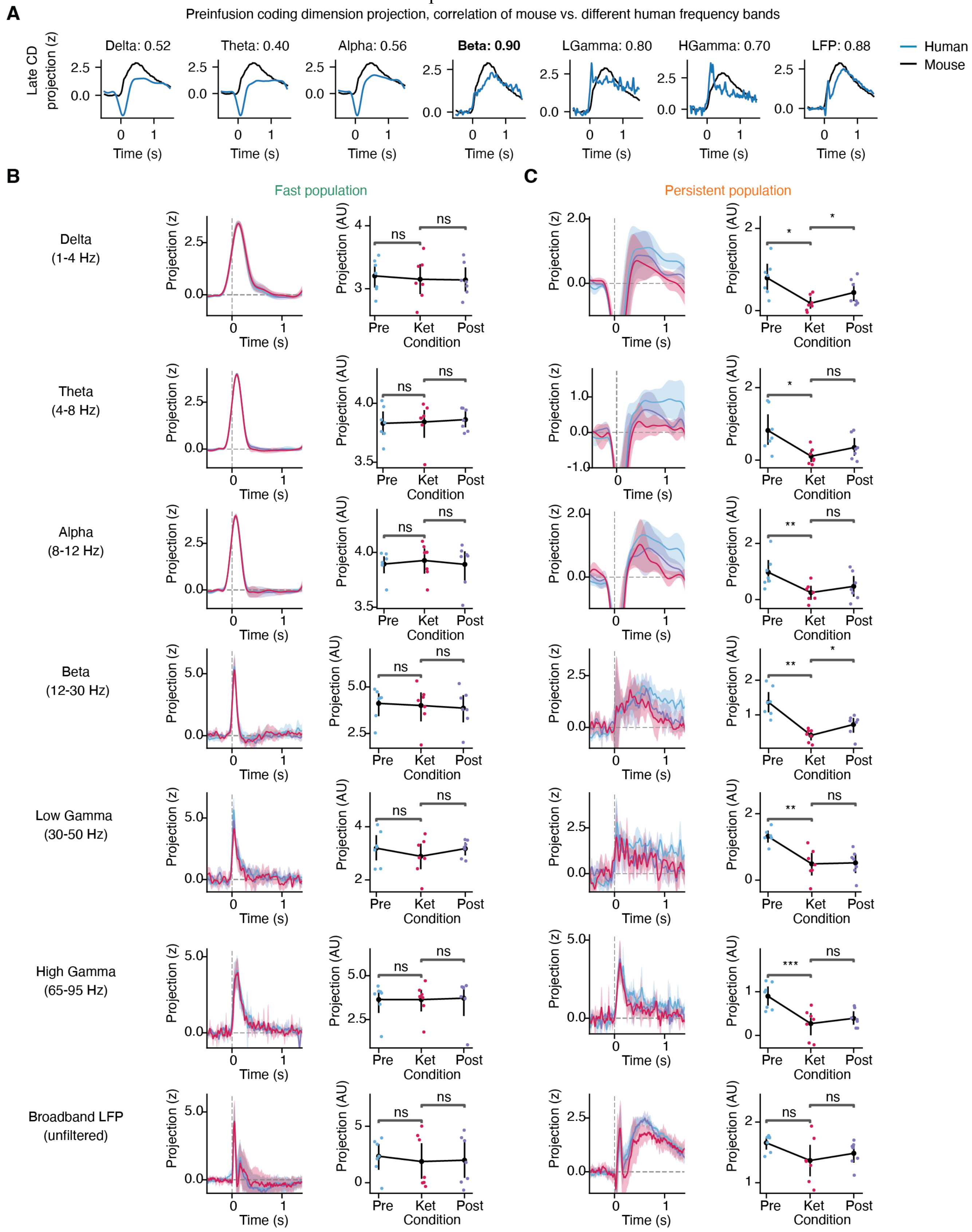

Fig. S37. Additional details on human coding dimension. (A) Preinfusion projection of late coding dimension (CD) computed using different spectral components of the electrical recording, and correlation with mouse firing rate-based CD. Beta power yields a CD projection that is most similar to that of the mouse. (B) Left: Early CD projections in humans for each spectral band during preinfusion, infusion, and postinfusion conditions (n=7 subjects, mean ± s.e.m.). Right: No

significant differences across conditions of mean projection during early window. (C) Left: Late CD projections in humans for each spectral band (n=7 subjects, mean ± s.e.m.). Right: All power bands (except for broadband LFP) exhibit significant decrease of persistent population projection from preinfusion to ketamine. Beta and delta further exhibit significant recovery during postinfusion. ns, P-value ≥ 0.05. *, P-value < 0.05. **, P-value < 0.01. ***, P-value < 0.001. ****, P-value < 0.0001. See Supplementary table 3 for information on statistical analyses and sample sizes.

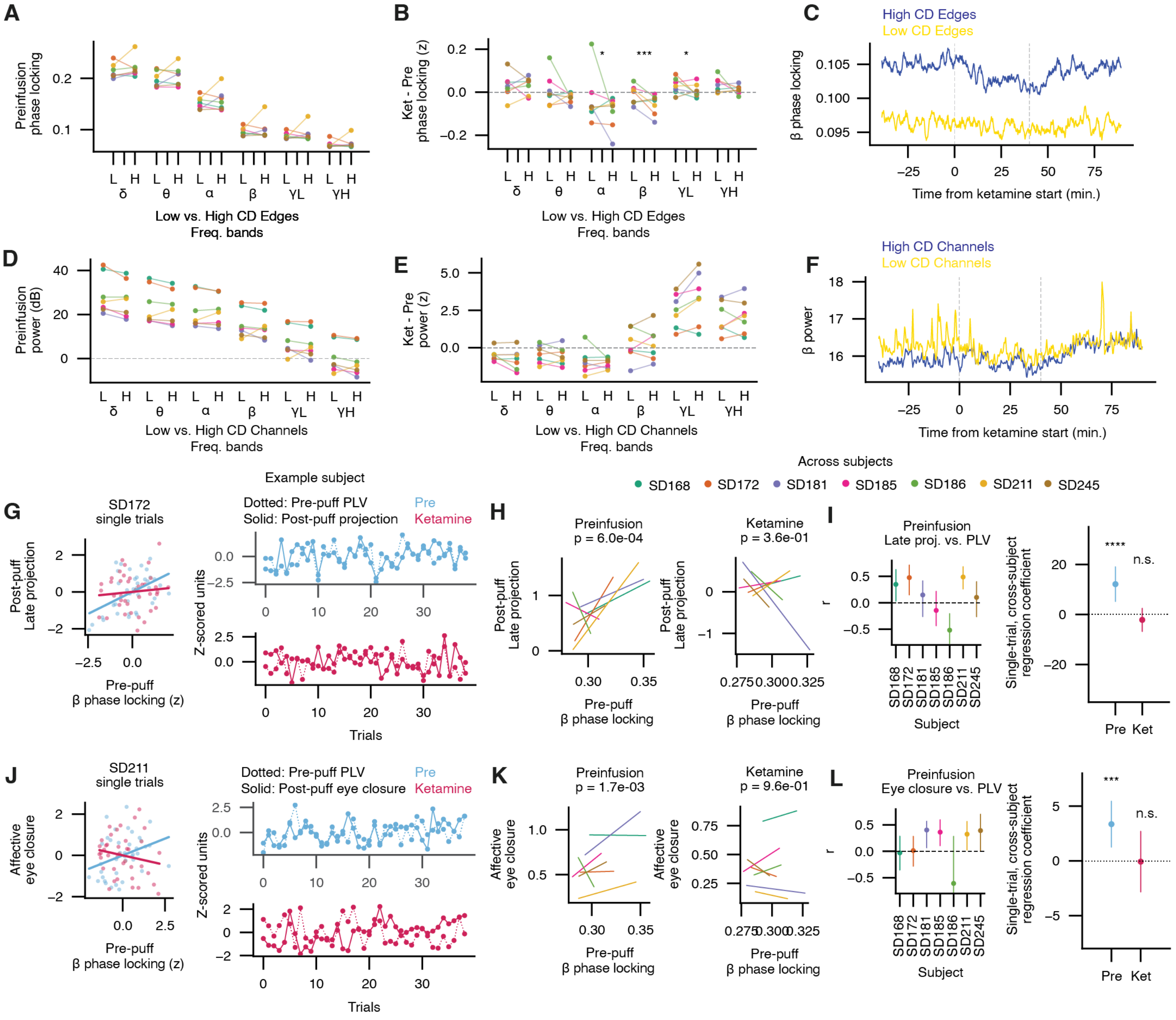

Fig. S38. Additional detail on human coupling changes across frequency bands and alterations by ketamine. (A) Preinfusion, out-of-task phase locking is not significantly different among edges between low puff-triggered persistence sites (low CD, denoted as L) vs. among edges between high persistence sites (high CD, denoted as H). (B) However, ketamine-induced decreases in coupling are greater in magnitude between high persistence sites, compared to between low persistence sites. Plot shows change (ketamine – preinfusion) in phase locking among low or high CD weight sites. Bipolar sites are classified as H or L by their weight assigned within the persistent peri-eyepuff neural activity dimension, or coding dimension, abbreviated CD. Low refers to sites in the bottom 10 percentile of CD weight and high refers to sites in the top 10 percentile of CD weight, for CDs computed using power in each spectral band. (C) Beta phase locking changes among high persistence sites show kinetics (mean over edges and patients) that resemble the pharmacokinetics of ketamine, and differ from the kinetics of low CD edges. This suggests a link between the dissociative state and coupling changes among the persistent sites. (D) Preinfusion power of low persistence sites is not significantly different from high persistence sites. (E) Ketamine alteration of power is also not significantly different between low and high persistence sites, and thus differences in ketamine-induced coupling changes between high and low persistence subnetworks (shown in (B)) are not necessarily due to differences in spectral power changes. Plot shows change (ketamine – preinfusion) in spectral power among low or high CD weight sites. (F) Temporal progression of beta spectral power around 40-minute ketamine infusion. Kinetics of spectral power do not markedly differ between high and low CD sites, in contrast to kinetics of phase locking (as shown in (C)). (G) Single-trial correlation of pre-puff network coupling with post-puff persistent activity for an example subject, SD168. Network coupling is measured as the beta phase locking value averaged across all recorded edges between

non-noise channels, averaged in the 0.5 seconds before a puff. Persistent activity is measured as the projection of population activity along the affective window beta coding dimension, i.e. the linear combination of all channel beta-filtered powers weighted by coding dimension weights. Left: single-trial scatter and best linear fit, comparing preinfusion and ketamine trials in different colors. Right: the progression of these metrics over trials during preinfusion (top) and ketamine (bottom) air puff sessions. Pre-puff network coupling is represented by a dotted line, and post-puff persistent activity is represented by a solid line. (H) Network coupling and persistent activity correlation as in (G) but represented across all patients. The two plots represent the best linear fits for all patients during preinfusion and ketamine, each patient represented in a different color. P-value is computed as the significance of the cross-patient correlation coefficient, from the hierarchical mixed-effects linear model with random intercept, grouping trials across patients. (I) Statistical significance of each patients' per-trial coupling to persistent activity correlation. Left: per-patient correlation coefficient (*r*) with bootstrapped confidence intervals. Right: cross-patient regression coefficient is significantly positive for preinfusion air puff sessions, but not significant for ketamine sessions. Cross-patient coefficients are computed using a hierarchical mixed linear model with random intercept grouped across patients. Error bars denote the 95% confidence interval of coefficient estimates. (J) Single trial correlation of pre-puff network coupling with post-puff affective eye closure for an example subject, SD211. Left and right are analogous to (G). Pre-puff network coupling is represented by a dotted line, and post-puff affective behavior is represented by a solid line. (K) Network coupling and affective behavior correlation as in (J) but represented across all patients. The two plots represent best linear fits for all patients during preinfusion and ketamine, each patient represented in a different color. (L) Statistical significance of each patients' per-trial coupling to eye closure correlation. Left: per-patient correlation coefficient (*r*) with bootstrapped confidence intervals. Right: cross-patient regression coefficient is significantly positive for preinfusion air puff sessions, but not significant for ketamine sessions. Cross-patient coefficients computed as above. ns, P-value ≥ 0.05. *, P-value < 0.05. **, P-value < 0.01. ***, P-value < 0.001. ****, P-value < 0.0001. See Supplementary table 3 for information on statistical analyses and sample sizes.

		Number of Significant Contacts							Total Number of Contacts							Total	Total	Fraction
HCP Cortical Area	Region	SD168	SD172	SD181	SD185	SD186	SD211	SD245	SD168	SD172	SD181	SD185	SD186	SD211	SD245	Significant	Contacts	Significant
R_RSC_ROI	PMC	0	0	0	0	0	0	1	2	0	0	0	0	0	3	1	5	0.2
R_PSL_ROI	SMG	0	2	0	0	0	0	0	0	2	0	0	0	0	1	2	3	0.7
R_PCV_ROI	PMC	0	1	0	0	0	0	0	0	2	0	0	0	0	0	1	2	0.5
R_STV_ROI	SMG	0	2	0	0	0	0	0	0	2	0	0	0	0	0	2	2	1.0
R_23d_ROI	PMC	0	0	0	0	0	0	2	0	1	0	1	0	0	3	2	5	0.4
R_5mv_ROI	PMC	1	0	3	0	0	0	0	2	0	3	0	0	0	0	4	5	0.8
R_23c_ROI	PMC	0	0	0	1	0	0	0	0	2	0	2	0	0	0	1	4	0.3
R_24dd_ROI	SMC	0	0	2	0	0	0	0	0	0	2	0	0	0	0	2	2	1.0
R_7Am_ROI	PMC	0	3	0	0	0	0	0	0	3	0	0	0	0	0	3	3	1.0
R_MIP_ROI	IPS	0	0	0	0	0	0	1	0	0	0	0	0	0	1	1	1	1.0
R_p32pr_ROI	ACC	0	0	2	0	0	0	1	0	0	3	0	0	0	1	3	4	0.8
R_47m_ROI	ORB	0	1	0	0	0	0	0	0	1	0	0	0	0	0	1	1	1.0
R_44_ROI	IFG	0	3	0	0	0	0	0	0	4	3	0	0	0	1	3	8	0.4
R_45_ROI	IFG	0	1	0	0	0	0	0	0	1	2	3	0	0	1	1	7	0.1
R_11l_ROI	ORB	0	2	0	0	0	0	0	0	3	0	0	0	0	0	2	3	0.7
R_13l_ROI	ORB	0	0	3	0	0	0	0	0	0	3	0	0	0	0	3	3	1.0
R_OFC_ROI	ORB	0	3	0	0	0	0	0	0	3	2	1	0	0	0	3	6	0.5
R_OP1_ROI	SMC	0	0	0	1	0	0	0	0	0	0	3	0	0	0	1	3	0.3
R_OP2-3_ROI	INS	0	2	0	0	0	0	0	0	3	0	0	0	0	0	2	3	0.7
R_RI_ROI	IFG	0	0	0	0	0	0	4	0	0	0	0	0	0	5	4	5	0.8
R_PFcm_ROI	SMG	0	0	0	0	0	0	1	0	0	0	0	0	0	1	1	1	1.0
R_PoI2_ROI	INS	1	4	0	0	0	0	0	2	4	0	0	0	0	0	5	6	0.8
R_FOP4_ROI	IFG	0	1	2	0	0	0	1	0	1	3	0	0	0	1	4	5	0.8
R_MI_ROI	INS	0	3	0	0	0	0	0	0	5	3	0	0	0	0	3	8	0.4
R_AVI_ROI	IFG	0	0	1	0	0	0	0	0	0	1	2	0	0	0	1	3	0.3
R_FOP3_ROI	INS	0	1	0	0	0	0	0	0	5	0	0	0	0	0	1	5	0.2
R_H_ROI	HIPP	4	0	0	0	0	0	0	5	2	5	3	0	0	2	4	17	0.2
R_A5_ROI	STS	0	2	0	0	0	0	0	1	3	0	0	0	0	1	2	5	0.4
R_STSdp_ROI	STS	0	1	0	0	0	0	0	0	2	0	0	0	0	0	1	2	0.5
R_TE1a_ROI	MTG	0	0	0	1	0	0	0	3	1	0	2	0	0	0	1	6	0.2
R_TE2p_ROI	FG	0	0	1	0	0	0	0	0	0	1	0	0	0	0	1	1	1.0
R_TPOJ3_ROI	AG	0	0	0	1	0	0	0	0	0	0	1	0	0	0	1	1	1.0
R_31a_ROI	PMC	2	0	0	0	0	0	0	2	0	0	0	0	0	1	2	3	0.7
R_pOFC_ROI	ORB	0	0	0	2	0	0	0	0	0	0	2	0	0	0	2	2	1.0
R_PoI1_ROI	INS	0	1	0	0	0	0	5	0	3	0	0	0	0	6	6	9	0.7
R_Ig_ROI	INS	0	2	0	1	0	0	0	0	2	0	1	0	0	0	3	3	1.0
R_FOP5_ROI	IFG	0	1	2	0	0	0	0	0	1	3	0	0	0	0	3	4	0.8
R_MBelt_ROI	STG	1	0	0	0	0	0	0	1	0	0	0	0	0	0	1	1	1.0
R_PI_ROI	STG	0	0	0	0	0	0	2	0	0	0	0	0	0	2	2	2	1.0
L_RSC_ROI	PMC	1	0	0	0	0	0	0	1	0	0	0	0	0	0	1	1	1.0
L_PSL_ROI	SMG	0	0	0	0	0	0	2	0	0	0	0	0	0	2	2	2	1.0
L_PCV_ROI	PMC	0	0	0	0	0	0	1	0	0	0	0	0	0	2	1	2	0.5
L_d23ab_ROI	PMC	1	0	0	0	0	0	0	1	2	0	0	1	0	0	1	4	0.3
L_5mv_ROI	PMC	0	0	0	0	1	0	0	1	0	0	0	1	0	0	1	2	0.5
L_23c_ROI	PMC	0	0	0	0	1	0	1	1	1	0	0	2	0	2	2	6	0.3
L_33pr_ROI	MCC	0	0	0	0	0	0	3	0	0	0	0	0	0	3	3	3	1.0
L_9p_ROI	SFG	0	0	0	0	0	0	1	0	0	0	0	0	0	1	1	1	1.0
L_44_ROI	IFG	0	0	1	0	0	0	0	0	2	2	0	0	0	0	1	4	0.3
L_47l_ROI	IFG	0	0	0	0	0	0	1	0	0	0	0	1	0	1	1	2	0.5
L_IFSp_ROI	IFG	0	0	1	0	0	0	0	0	2	1	0	0	0	0	1	3	0.3
L_9a_ROI	SFG	0	0	0	0	0	0	2	0	0	0	0	0	0	2	2	2	1.0
L_OP4_ROI	PRECG	0	0	0	0	0	1	0	1	0	0	0	0	4	0	1	5	0.2
L_OP1_ROI	SMG	0	0	0	0	0	1	0	0	0	0	0	0	1	0	1	1	1.0
L_52_ROI	STG	1	0	0	0	0	0	0	1	0	0	0	0	0	1	1	2	0.5
L_RI_ROI	INS	0	0	0	0	2	3	6	0	0	0	0	3	3	6	11	12	0.9
L_PoI2_ROI	INS	0	0	0	0	0	2	0	1	0	0	0	0	2	0	2	3	0.7
L_FOP4_ROI	INS	0	0	0	0	0	0	2	0	1	0	0	0	0	2	2	3	0.7
L_AVI_ROI	IFG	0	0	0	0	0	0	1	0	0	0	0	0	0	1	1	1	1.0
L_FOP2_ROI	INS	1	0	0	0	0	1	0	2	0	0	0	0	1	0	2	3	0.7
L_H_ROI	HIPP	4	2	1	0	0	0	1	12	5	4	0	5	5	2	8	33	0.2
L_PHA1_ROI	PHG	1	0	0	0	0	0	0	1	0	0	0	0	1	0	1	2	0.5
L_PHA3_ROI	HIPP	1	0	0	0	0	0	1	1	0	0	0	0	2	1	2	4	0.5
L_STSvp_ROI	STS	2	0	0	0	0	0	0	5	0	0	0	0	0	1	2	6	0.3
L_TE2a_ROI	ITG	0	0	0	0	0	2	0	2	0	0	0	0	7	0	2	9	0.2
L_TF_ROI	TP	0	1	0	0	0	0	0	0	1	0	0	0	9	1	1	11	0.1
L_TE2p_ROI	ITG	0	0	0	0	0	2	0	0	0	0	0	1	3	0	2	4	0.5
L_TPOJ1_ROI	MTG	1	0	0	0	0	0	0	1	0	0	0	0	0	0	1	1	1.0
L_TPOJ2_ROI	MTG	1	0	0	0	0	0	0	3	0	0	0	0	0	0	1	3	0.3
L_VMV1_ROI	PHG	1	0	0	0	0	0	0	1	0	0	0	0	0	0	1	1	1.0
L_VMV2_ROI	PHG	1	0	0	0	0	0	0	1	0	0	0	0	0	0	1	1	1.0
L_PoI1_ROI	INS	0	0	0	0	0	0	7	0	0	0	0	0	0	9	7	9	0.8
L_FOP5_ROI	IFG	0	0	0	0	0	0	1	0	0	0	0	0	0	1	1	1	1.0
L_MBelt_ROI	STG	1	0	0	0	0	0	0	1	0	0	0	0	0	0	1	1	1.0
L_a32pr_ROI	MCC	0	0	0	0	0	0	1	0	0	0	0	0	0	2	1	2	0.5
All areas with significant contacts		26	39	19	7	4	12	49	55	70	41	21	14	38	70	156	309	0.5

		Number of Significant Contacts							Total Number of Contacts							Total	Total	Fraction
HCP Cortical Area	Region	SD168	SD172	SD181	SD185	SD186	SD211	SD245	SD168	SD172	SD181	SD185	SD186	SD211	SD245	Significant	Contacts	Significant
R_4_ROI	MOT	0	0	0	0	0	0	0	0	0	0	1	0	0	0	0	1	0.0
R_v23ab_ROI	PMC	0	0	0	0	0	0	0	0	0	0	1	0	0	0	0	1	0.0
R_d23ab_ROI	PMC	0	0	0	0	0	0	0	0	1	0	1	0	0	1	0	3	0.0
R_24dv_ROI	ACC	0	0	0	0	0	0	0	0	0	3	0	0	0	0	0	3	0.0
R_1_ROI	SS	0	0	0	0	0	0	0	0	0	0	3	0	0	0	0	3	0.0
R_33pr_ROI	MCC	0	0	0	0	0	0	0	0	0	0	0	0	0	3	0	3	0.0
R_d32_ROI	ACC	0	0	0	0	0	0	0	0	0	0	1	0	0	0	0	1	0.0
R_8BM_ROI	MSFG	0	0	0	0	0	0	0	0	0	0	0	0	0	2	0	2	0.0
R_8Av_ROI	MFG	0	0	0	0	0	0	0	0	0	0	2	0	0	0	0	2	0.0
R_8Ad_ROI	MFG	0	0	0	0	0	0	0	0	0	0	2	0	0	1	0	3	0.0
R_8BL_ROI	MFC	0	0	0	0	0	0	0	0	0	0	1	0	0	0	0	1	0.0
R_9p_ROI	MFC	0	0	0	0	0	0	0	0	0	0	1	0	0	1	0	2	0.0
R_8C_ROI	MFG	0	0	0	0	0	0	0	0	0	1	0	0	0	0	0	1	0.0
R_6r_ROI	IFG	0	0	0	0	0	0	0	0	1	0	1	0	0	0	0	2	0.0
R_IFSp_ROI	IFG	0	0	0	0	0	0	0	0	1	0	0	0	0	0	0	1	0.0
R_IFSa_ROI	IFG	0	0	0	0	0	0	0	0	1	0	0	0	0	0	0	1	0.0
R_AAIC_ROI	INS	0	0	0	0	0	0	0	0	0	1	0	0	0	0	0	1	0.0
R_EC_ROI	HIPP	0	0	0	0	0	0	0	1	0	0	0	0	0	0	0	1	0.0
R_PBelt_ROI	STG	0	0	0	0	0	0	0	1	0	0	0	0	0	0	0	1	0.0
R_PHA1_ROI	PHG	0	0	0	0	0	0	0	0	1	0	0	0	0	0	0	1	0.0
R_STSda_ROI	MTG	0	0	0	0	0	0	0	0	2	0	0	0	0	1	0	3	0.0
R_STSvp_ROI	MTG	0	0	0	0	0	0	0	0	1	0	0	0	0	0	0	1	0.0
R_TGd_ROI	TP	0	0	0	0	0	0	0	0	3	0	0	0	0	0	0	3	0.0
R_TE1p_ROI	ITG	0	0	0	0	0	0	0	0	2	4	0	0	0	0	0	6	0.0
R_TE2a_ROI	ITG	0	0	0	0	0	0	0	0	1	0	0	0	0	0	0	1	0.0
R_IP0_ROI	MOG	0	0	0	0	0	0	0	0	0	0	0	0	0	1	0	1	0.0
R_PFop_ROI	SS	0	0	0	0	0	0	0	0	0	0	1	0	0	0	0	1	0.0
R_PF_ROI	SMG	0	0	0	0	0	0	0	0	0	0	0	0	0	1	0	1	0.0
R_PGi_ROI	AG	0	0	0	0	0	0	0	0	0	0	4	0	0	4	0	8	0.0
R_A4_ROI	STG	0	0	0	0	0	0	0	1	0	0	0	0	0	0	0	1	0.0
R_STSva_ROI	MTG	0	0	0	0	0	0	0	0	3	0	2	0	0	0	0	5	0.0
R_TE1m_ROI	ITG	0	0	0	0	0	0	0	0	0	1	0	0	0	0	0	1	0.0
R_a32pr_ROI	ACC	0	0	0	0	0	0	0	0	0	1	1	0	0	0	0	2	0.0
R_p24_ROI	ACC	0	0	0	0	0	0	0	0	0	0	1	0	0	0	0	1	0.0
L_FFC_ROI	CS	0	0	0	0	0	0	0	0	0	0	0	0	1	0	0	1	0.0
L_23d_ROI	MCC	0	0	0	0	0	0	0	0	0	0	0	0	0	3	0	3	0.0
L_7AL_ROI	SPL	0	0	0	0	0	0	0	1	0	0	0	2	0	0	0	3	0.0
L_7PC_ROI	SPL	0	0	0	0	0	0	0	0	0	0	0	1	0	0	0	1	0.0
L_p24pr_ROI	MCC	0	0	0	0	0	0	0	0	0	0	0	0	0	1	0	1	0.0
L_47m_ROI	ORB	0	0	0	0	0	0	0	0	0	0	0	2	0	0	0	2	0.0
L_45_ROI	IFG	0	0	0	0	0	0	0	0	0	0	0	4	0	5	0	9	0.0
L_6r_ROI	IFG	0	0	0	0	0	0	0	0	1	0	0	2	0	0	0	3	0.0
L_13l_ROI	ORB	0	0	0	0	0	0	0	0	0	0	0	2	0	1	0	3	0.0
L_OFC_ROI	OFC	0	0	0	0	0	0	0	0	0	0	0	2	0	2	0	4	0.0
L_PFcm_ROI	SMG	0	0	0	0	0	0	0	0	0	0	0	1	1	0	0	2	0.0
L_MI_ROI	INS	0	0	0	0	0	0	0	0	0	0	0	1	0	0	0	1	0.0
L_FOP1_ROI	IFG	0	0	0	0	0	0	0	0	0	0	0	1	0	0	0	1	0.0
L_FOP3_ROI	INS	0	0	0	0	0	0	0	0	1	1	0	0	0	0	0	2	0.0
L_PeEc_ROI	TP	0	0	0	0	0	0	0	0	0	0	0	0	2	0	0	2	0.0
L_A5_ROI	STG	0	0	0	0	0	0	0	1	0	0	0	0	0	0	0	1	0.0
L_STSda_ROI	MTG	0	0	0	0	0	0	0	0	0	0	0	1	0	0	0	1	0.0
L_TGd_ROI	TP	0	0	0	0	0	0	0	0	0	3	0	0	1	0	0	4	0.0
L_TE1a_ROI	MTG	0	0	0	0	0	0	0	2	0	1	0	0	2	0	0	5	0.0
L_TE1p_ROI	MTG	0	0	0	0	0	0	0	0	1	2	0	2	2	2	0	9	0.0
L_PHT_ROI	ITG	0	0	0	0	0	0	0	0	0	0	0	0	1	0	0	1	0.0
L_PH_ROI	ITG	0	0	0	0	0	0	0	0	0	0	0	0	2	0	0	2	0.0
L_PF_ROI	SMG	0	0	0	0	0	0	0	0	0	0	0	0	2	0	0	2	0.0
L_PFm_ROI	SMG	0	0	0	0	0	0	0	0	0	0	0	0	0	1	0	1	0.0
L_31a_ROI	PMC	0	0	0	0	0	0	0	1	1	0	0	1	0	1	0	4	0.0
L_VVC_ROI	FG	0	0	0	0	0	0	0	0	0	0	0	0	3	0	0	3	0.0
L_Ig_ROI	INS	0	0	0	0	0	0	0	0	0	0	0	0	0	1	0	1	0.0
L_LBelt_ROI	STG	0	0	0	0	0	0	0	1	0	0	0	0	0	0	0	1	0.0
L_A4_ROI	STG	0	0	0	0	0	0	0	0	0	0	0	1	0	0	0	1	0.0
L_STSva_ROI	MTG	0	0	0	0	0	0	0	0	0	0	0	3	0	1	0	4	0.0
L_TE1m_ROI	MTG	0	0	0	0	0	0	0	0	2	0	0	0	3	0	0	5	0.0
L_PI_ROI	INS	0	0	0	0	0	0	0	0	0	0	0	2	0	1	0	3	0.0
All areas with no significant contacts		0	0	0	0	0	0	0	9	23	18	23	28	20	34	0	155	0.0

Table S1. Anatomical detail about human iEEG changes on ketamine, by contact, and parcellated by Human Connectome Project (HCP) area. For each area, the number of total recorded contacts are specified for each subject and overall, and the number of recorded contacts that exhibit a significant ketamine-elicited spectrotemporal change in the puff-triggered spectrogram, as determined by permutation cluster test across trials paired between preinfusion and infusion (from 12 to 39 trials depending on which subject a contact came from). Top table specifies regions with at least one recorded contact that exhibits significant changes, and the bottom table specifies regions with no significantly changing contacts.

Acronym	Full name	Unit count	Session count
AAA	Anterior amygdalar area	23	1
ACAd1	Anterior cingulate area, dorsal part, layer 1	9	1
ACAd2/3	Anterior cingulate area, dorsal part, layer 2/3	39	4
ACAd5	Anterior cingulate area, dorsal part, layer 5	153	6
ACAd6b	Anterior cingulate area, dorsal part, layer 6b	3	1
ACAv1	Anterior cingulate area, ventral part, layer 1	43	4
ACAv2/3	Anterior cingulate area, ventral part, layer 2/3	77	4
ACAv5	Anterior cingulate area, ventral part, layer 5	77	3
ACAv6a	Anterior cingulate area, ventral part, 6a	4	1
ACB	Nucleus accumbens	317	8
AId5	Agranular insular area, dorsal part, layer 5	153	2
AId6a	Agranular insular area, dorsal part, layer 6a	70	2
AIv2/3	Agranular insular area, ventral part, layer 2/3	36	2
AIv5	Agranular insular area, ventral part, layer 5	171	2
AON	Anterior olfactory nucleus	89	2
BLAa	Basolateral amygdalar nucleus, anterior part	1	1
BLAv	Basolateral amygdalar nucleus, ventral part	1	1
BMAp	Basomedial amygdalar nucleus, posterior part	1	1
BST	Bed nuclei of the stria terminalis	35	3
CA1	Field CA1	146	7
CA2	Field CA2	5	2
CA3	Field CA3	57	6
CEAm	Central amygdalar nucleus, medial part	13	1
CLI	Central linear nucleus raphe	9	1
CM	Central medial nucleus of the thalamus	20	2
COApl	Cortical amygdalar area, posterior part, lateral zone	1	1
COApm	Cortical amygdalar area, posterior part, medial zone	26	2
CP	Caudoputamen	516	7
DG-mo	Dentate gyrus, molecular layer	87	6
DG-po	Dentate gyrus, polymorph layer	10	4
DG-sg	Dentate gyrus, granule cell layer	29	7
DP	Dorsal peduncular area	37	3
EPd	Endopiriform nucleus, dorsal part	135	1
Eth	Ethmoid nucleus of the thalamus	16	1
FF	Fields of Forel	59	4
FRP6a	Frontal pole, layer 6a	22	1
FS	Fundus of striatum	10	1
HPF	Hippocampal formation	9	3
HY	Hypothalamus	59	7
IF	Interfascicular nucleus raphe	4	1
IG	Induseum griseum	13	1
IGL	Intergeniculate leaflet of the lateral geniculate complex	15	2
ILA1	Infralimbic area, layer 1	46	1
ILA2/3	Infralimbic area, layer 2/3	11	1
ILA5	Infralimbic area, layer 5	26	1
IMD	Intermediodorsal nucleus of the thalamus	1	1
LGd-co	Dorsal part of the lateral geniculate complex, core	12	3
LGd-ip	Dorsal part of the lateral geniculate complex, ipsilateral zone	12	1
LGd-sh	Dorsal part of the lateral geniculate complex, shell	13	1
LGv	Ventral part of the lateral geniculate complex	28	3
LH	Lateral habenula	18	3
LHA	Lateral hypothalamic area	25	2
LP	Lateral posterior nucleus of the thalamus	102	5
LSc	Lateral septal nucleus, caudal (caudodorsal) part	8	1
LSr	Lateral septal nucleus, rostral (rostroventral) part	521	7
LSv	Lateral septal nucleus, ventral part	64	5
MB	Midbrain	310	8
MD	Mediodorsal nucleus of thalamus	240	4
MEPO	Median preoptic nucleus	1	1
MGd	Medial geniculate complex, dorsal part	1	1
MGm	Medial geniculate complex, medial part	3	1
MGv	Medial geniculate complex, ventral part	1	1
MOp2/3	Primary motor area, Layer 2/3	21	3
MOp5	Primary motor area, Layer 5	79	3
MOp6a	Primary motor area, Layer 6a	113	4
MOp6b	Primary motor area, Layer 6b	10	3
MOs5	Secondary motor area, layer 5	39	5
MOs6a	Secondary motor area, layer 6a	91	4
MRN	Midbrain reticular nucleus	100	2
ND	Nucleus of Darkschewitsch	7	1
NLOT1	Nucleus of the lateral olfactory tract, molecular layer	2	1
NLOT2	Nucleus of the lateral olfactory tract, pyramidal layer	32	1
NLOT3	Nucleus of the lateral olfactory tract, layer 3	2	1
NOT	Nucleus of the optic tract	11	2

Acronym	Full name	Unit count	Session count
NPC	Nucleus of the posterior commissure	106	6
OLF	Olfactory areas	277	9
OP	Olivary pretectal nucleus	20	2
ORBl1	Orbital area, lateral part, layer 1	22	1
ORBl2/3	Orbital area, lateral part, layer 2/3	118	4
ORBl5	Orbital area, lateral part, layer 5	360	5
ORBl6a	Orbital area, lateral part, layer 6a	100	3
ORBm5	Orbital area, medial part, layer 5	50	1
ORBm6a	Orbital area, medial part, layer 6a	18	1
ORBvl2/3	Orbital area, ventrolateral part, layer 2/3	111	2
ORBvl5	Orbital area, ventrolateral part, layer 5	57	2
OT	Olfactory tubercle	91	2
PA	Posterior amygdalar nucleus	15	1
PAG	Periaqueductal gray	159	5
PCN	Paracentral nucleus	23	1
PF	Parafascicular nucleus	2	1
PIR	Piriform area	212	3
PL1	Prelimbic area, layer 1	49	1
PL2/3	Prelimbic area, layer 2/3	85	1
PL5	Prelimbic area, layer 5	128	3
PL6a	Prelimbic area, layer 6a	11	1
PO	Posterior complex of the thalamus	345	5
PPT	Posterior pretectal nucleus	7	1
PRC	Precommissural nucleus	13	2
PS	Parastrial nucleus	3	1
PSTN	Parasubthalamic nucleus	25	2
PVT	Paraventricular nucleus of the thalamus	1	1
PoT	Posterior triangular thalamic nucleus	95	2
RPF	Retroparafascicular nucleus	11	1
RSPagl2/3	Retrosplenial area, lateral agranular part, layer 2/3	8	1
RSPagl5	Retrosplenial area, lateral agranular part, layer 5	20	1
RSPagl6a	Retrosplenial area, lateral agranular part, layer 6a	7	1
RSPagl6b	Retrosplenial area, lateral agranular part, layer 6b	2	1
RSPd2/3	Retrosplenial area, dorsal part, layer 2/3	3	1
RSPd5	Retrosplenial area, dorsal part, layer 5	11	3
RSPd6a	Retrosplenial area, dorsal part, layer 6a	25	2
RSPv1	Retrosplenial area, ventral part, layer 1	35	5
RSPv2/3	Retrosplenial area, ventral part, layer 2/3	51	5
RSPv5	Retrosplenial area, ventral part, layer 5	243	6
RSPv6a	Retrosplenial area, ventral part, layer 6a	89	3
RSPv6b	Retrosplenial area, ventral part, layer 6b	12	3
RT	Reticular nucleus of the thalamus	46	1
SCdg	Superior colliculus, motor related, deep gray layer	10	1
SCdw	Superior colliculus, motor related, deep white layer	8	1
SCig	Superior colliculus, motor related, intermediate gray layer	50	2
SCiw	Superior colliculus, motor related, intermediate white layer	25	2
SCop	Superior colliculus, optic layer	8	2
SCsg	Superior colliculus, superficial gray layer	15	2
SCzo	Superior colliculus, zonal layer	9	3
SF	Septofimbrial nucleus	40	1
SI	Substantia innominata	79	4
SNc	Substantia nigra, compact part	6	1
SNr	Substantia nigra, reticular part	3	1
SPA	Subparafascicular area	8	2
SPFp	Subparafascicular nucleus, parvicellular part	8	1
SSp-ll6a	Primary somatosensory area, lower limb, layer 6a	35	1
SSp-ll6b	Primary somatosensory area, lower limb, layer 6b	3	1
SSp-m1	Primary somatosensory area, mouth, layer 1	4	1
SSp-m2/3	Primary somatosensory area, mouth, layer 2/3	7	1
SSp-m4	Primary somatosensory area, mouth, layer 4	16	1
SSp-m5	Primary somatosensory area, mouth, layer 5	17	1
SSp-m6a	Primary somatosensory area, mouth, layer 6a	40	1
SSp-ul6a	Primary somatosensory area, upper limb, layer 6a	8	1
SSp-ul6b	Primary somatosensory area, upper limb, layer 6b	1	1
SSs6b	Supplemental somatosensory area, layer 6b	1	1
STN	Subthalamic nucleus	19	1
STR	Striatum	59	9
SUB	Subiculum	67	3
TH	Thalamus	62	6
TTd	Taenia tecta, dorsal part	14	3
VAL	Ventral anterior-lateral complex of the thalamus	40	2
VPLpc	Ventral posterolateral nucleus of the thalamus, parvicellular part	52	2
VPM	Ventral posteromedial nucleus of the thalamus	107	1
VPMpc	Ventral posteromedial nucleus of the thalamus, parvicellular part	49	2
ZI	Zona incerta	201	7

Table S2. Anatomical details about mouse Neuropixels recordings. Areas parcellated according to Allen Brain Institute Reference Atlas (CCFv3).

Table S3.

Summary of statistical analyses

Fig. 1D
Analysis: Puff-triggered eye closure of an example human subject, averaged across trials, comparing saline and ketamine (0.5 mg/kg) conditions.

Condition	# trials	Plot
Saline	38	Mean ± s.e.m.
Ketamine	38	Mean ± s.e.m.

Fig. 1E
Analysis: Normalized late eye closure (late divided by early) in humans, saline vs. ketamine. Point estimates for each subject are mean ± s.e.m. across trials, excluding the first two trials.

Null hypothesis: No difference between ketamine and saline conditions.

Condition	# subjects	# trials per subject	Mean ± s.e.m.	Statistical test	Test statistic	p-Value
Saline	4	38	0.71 ± 0.08	Paired t-test	3.703	0.0342
Ketamine	4	38	0.43 ± 0.12			

Fig. 1J
Analysis: Puff-triggered eye closure of an example mouse, averaged across the first puff of each trial (trials consist of 8 closely-spaced puffs), comparing saline and ketamine (50 mg/kg) conditions.

Condition	# trials	Plot
Saline	15	Mean ± s.e.m.
Ketamine	15	Mean ± s.e.m.

Fig. 1K
Analysis: Normalized late eye closure (late divided by early) in mice for first puff of each trial, saline vs. ketamine.

Null hypothesis: No difference between ketamine and saline conditions.

Condition	# mice	# trials per mouse	Mean ± s.e.m.	Statistical test	Test statistic	p-Value
Saline	5	15	0.26 ± 0.06	Paired t-test	4.737	0.009
Ketamine	5	15	-0.07 ± 0.06			

Fig. 1L
Analysis: Normalized late eye closure (late divided by early) in mice for first puff of each trial, preinfusion vs. infusion for saline, ketamine (50 mg/kg), and PCP (20 mg/kg).

Null hypothesis: No difference between preinfusion and infusion.

Condition	# mice	# trials per mouse	Mean ± s.e.m.	Statistical test	Comparison	Test statistic	Corrected p-Value
Pre-saline	5	15	0.29 ± 0.08	Paired t-test with fdr-bh correction	Pre-saline vs. saline	0.73	0.504
Saline	5	15	0.26 ± 0.06				
Pre-ketamine	5	15	0.37 ± 0.06		Pre-ketamine vs. ketamine	9.53	0.002
Ketamine	5	15	-0.07 ± 0.06				

Pre-PCP	5	15	0.35 ± 0.10		Pre-PCP vs. PCP	3.41	0.041
PCP	5	15	-0.03 ± 0.05				

Fig. 1M
Analysis: Average eye closure across trials (a trial consists of 8 puffs each separated by 3 s), averaged across mice, comparing preinfusion and saline infusion conditions.

Condition	# mice	# trials per mouse	Plot
Preinfusion	5	15	mean ± s.e.m.
Saline	5	15	mean ± s.e.m.

Fig. 1N
Analysis: Average eye closure across trials (a trial consists of 8 puffs each separated by 3 s), averaged across mice, comparing preinfusion and ketamine (50 mg/kg) infusion conditions.

Condition	# mice	# trials per mouse	Plot
Preinfusion	5	15	mean ± s.e.m.
Ketamine	5	15	mean ± s.e.m.

Fig. 1O
Analysis: Average eye closure across trials (a trial consists of 8 puffs each separated by 3 s), averaged across mice, comparing preinfusion and PCP (20 mg/kg) infusion conditions.

Condition	# mice	# trials per mouse	Plot
Preinfusion	5	15	mean ± s.e.m.
PCP	5	15	mean ± s.e.m.

Fig. 1P
Analysis: Average eye closure across trials (a trial consists of 8 puffs each separated by 3 s), averaged across mice, comparing preinfusion and anesthetic dose of K/X (135 mg/kg ketamine with 15 mg/kg xylazine).

Condition	# mice	# trials per mouse	Plot
Preinfusion	5	15	mean ± s.e.m.
K/X	5	15	mean ± s.e.m.

Fig. 2C
Analysis: Average puff-triggered eye closure comparing preinfusion, infusion, and postinfusion conditions.

Condition	# human participants	# trials per subject	Plot
Preinfusion	7	mean: 30.6, min: 12, max: 39	mean ± s.e.m.
Infusion	7	mean: 30.6, min: 12, max: 39	mean ± s.e.m.
Postinfusion	7	mean: 30.6, min: 12, max: 39	mean ± s.e.m.

Fig. 2D
Analysis: Clinician administered dissociative states score (CADSS) before, during, and after ketamine infusion across the subjects that met the eyepuff behavioral inclusion criteria.

Null hypothesis: No difference between infusion and preinfusion or postinfusion.

Condition	# human participants	Mean ± s.e.m.	Statistical test	Comparison	Test statistic	Corrected p-Value
Preinfusion	7	0.86 ± 0.46	Paired t-test with fdr-bh correction			
Ketamine	7	22.43 ± 4.75		Preinfusion vs. ketamine	4.807	0.004

Postinfusion	7	0.86 ± 0.46		Postinfusion vs. ketamine	4.512	0.004

Fig. 2E
Analysis: Preinfusion event related potential for example channels from insula (INS) and posteromedial cortex (PMC), both from subject SD172. Mean across trials shown in teal, individual trials in gray. All trials, excluding the first trial, are shown (39 trials).

Fig. 2G
Analysis: Factor projections, corresponding to matrix H in a 6-component non-negative matrix factorization D = WH. The procedure is schematized in 2F. The matrix D is of shape [channels x frequency x time], formed by stacking the mean spectrogram across a training set of half of the preinfusion and postinfusion trials for all channels from all subjects (458 total channels). W is [channels x 6], and H is [6 x frequency x time]. NMF is applied by first splitting D into positive and negative parts, and computing NMF with k=3 on each part. Only channels localized to the 21 regions that were sampled in at least two subjects were included (ORB, IFG, MFG, INS, ACC, MCC, PMC, MOT, SS, SMG, PHG, STG, STS, MTG, ITG, TP, HIPP, BG, AMY, THAL ANT, THAL POS). The total number of channels was 458, with a breakdown by subject as follows: SD168=86, SD172=97, SD181=58, SD185=43, SD186=47, SD211=44, SD245=83. The number of components was selected by using cross-validation on a test set of trials, and identifying an elbow in the test set explained variance, which occurred at K=6.

For subsequent analyses, factor loadings for each channel, corresponding to rows in the matrix W are computed for each condition (e.g. preinfusion or infusion) by projecting H on to the D corresponding to that condition. Here, D is formed by averaging the spectrogram across a test set of half of the trials of that condition (and that are distinct from the training set of trials used to compute the factorization). Loadings represent the extent to which a factor is 'active' in the neural response of that channel. For each factor, channels with a loading of less than 0.1 on the average across preinfusion and postinfusion test set trials were excluded from subsequent analysis for that factor.

Fig. 2H
Analysis: Factor loadings, corresponding to W in NMF factorization D = WH, where W is [channels x 6], averaged across channels grouped by Yeo7 network. Loadings are then normalized within each row (divided by the sum of the row).

Fig. 2I
Analysis: Factor loading before, during, and after infusion, mean ± s.e.m. across channels. Mixed effect linear model, channels grouped by subject, [pre + post]/2 vs. ketamine (for channels with factor loading across the average of preinfusion and postinfusion test trials of at least 0.1).

Null hypothesis: No difference between infusion and average of preinfusion and postinfusion.

Condition	# human participants	# channels	Mean ± s.e.m.	Statistical test	Comparison	Coefficient	Corrected p-Value
Factor 0 Pre.	7	312	0.48 ± 0.02	Mixed-effect linear model, grouped by subject, with fdr-bh correction across factors	(Pre + Post)/2 vs. Infusion	-0.049	0.121
Factor 0 Inf.			0.42 ± 0.02				
Factor 0 Post.			0.45 ± 0.02				
Factor 1 Pre.	7	260	0.51 ± 0.02		(Pre + Post)/2 vs. Infusion	-0.125	2.56e-05
Factor 1 Inf.			0.32 ± 0.02				
Factor 1 Post.			0.38 ± 0.02				
Factor 2 Pre.	7	185	0.35 ± 0.02			-0.115	5.51e-08

Factor 2 Inf.			0.21 ± 0.01		(Pre + Post)/2 vs. Infusion		
Factor 2 Post.			0.30 ± 0.02				
Factor 3 Pre.	5	158	0.28 ± 0.01		(Pre + Post)/2 vs. Infusion	-0.041	0.016†
Factor 3 Inf.			0.21 ± 0.01				
Factor 3 Post.			0.23 ± 0.01				
Factor 4 Pre.	6	370	0.32 ± 0.01		(Pre + Post)/2 vs. Infusion	0.013	0.406
Factor 4 Inf.			0.39 ± 0.01				
Factor 4 Post.			0.42 ± 0.01				
Factor 5 Pre.	7	378	0.39 ± 0.01		(Pre + Post)/2 vs. Infusion	0.013	0.341
Factor 5 Inf.			0.43 ± 0.01				
Factor 5 Post.			0.44 ± 0.01				

†Mixed-effect model, grouped by subject, did not converge for Factor 3. Linear model without grouping by subject does converge and yields a corrected p-value of 0.023.

As a post-hoc test, we further assessed the comparison between infusion and preinfusion and separately between infusion and postinfusion.

Null hypothesis: No difference between infusion and either preinfusion or postinfusion.

Condition	# human participants	# channels	Mean ± s.e.m.	Statistical test	Comparison	Coefficient	Corrected p-Value
Factor 0 Pre.	7	312	0.48 ± 0.02	Mixed-effect linear model, grouped by subject, with fdr-bh correction across factors	Pre vs. Infusion	0.065	0.036
Factor 0 Inf.			0.42 ± 0.02		Post vs. Infusion	0.033	0.304
Factor 0 Post.			0.45 ± 0.02				
Factor 1 Pre.	7	260	0.51 ± 0.02		Pre vs. Infusion	0.190	1.80e-09
Factor 1 Inf.			0.32 ± 0.02		Post vs. Infusion	0.061	0.046
Factor 1 Post.			0.38 ± 0.02				
Factor 2 Pre.	7	185	0.35 ± 0.02		Pre vs. Infusion	0.140	1.25e-08
Factor 2 Inf.			0.21 ± 0.01		Post vs. Infusion	0.089	5.16e-05
Factor 2 Post.			0.30 ± 0.02				
Factor 3 Pre.	5	158	0.28 ± 0.01		Pre vs. Infusion	0.068	5.16e-04

Factor 3 Inf.			0.21 ± 0.01		Post vs. Infusion	0.014	0.394
Factor 3 Post.			0.23 ± 0.01				
Factor 4 Pre.	6	370	0.32 ± 0.01		Pre vs. Infusion	0.063	2.99e-04
Factor 4 Inf.			0.39 ± 0.01		Post vs. Infusion	0.037	0.046
Factor 4 Post.			0.42 ± 0.01				
Factor 5 Pre.	7	378	0.39 ± 0.01		Pre vs. Infusion	0.038	0.010
Factor 5 Inf.			0.43 ± 0.01		Post vs. Infusion	0.011	0.394
Factor 5 Post.			0.44 ± 0.01				

Fig. 2J
Analysis: Change in factor loadings, ketamine – preinfusion, corresponding to W in NMF factorization D = WH, where W is [channels x 6], averaged over the first dimension across channels grouped by Yeo7 network. Factorization H is computed using the average across preinfusion and postinfusion trials as described above, and used to compute W during ketamine and preinfusion.

Fig. 3B
Analysis: Permutation cluster test (mne.stats.permutation_cluster_test) comparing preinfusion and infusion puff-triggered spectrograms on each trial for each Yeo7 network (except for the Visual network, which was not present in our dataset). For each subject, all channels in a network were averaged into a single trace for each trial. All trials were stacked across subjects, yielding the total number of trials (which are indicated parenthetically for each network). The test statistic was a 1-sample t-test of the difference between each preinfusion and infusion trial (infusion – preinfusion). As the same eyepuff protocol was used during preinfusion and infusion (with the same ordering of interpuff interval), trials were paired to account for any within-puff-sequence trends. A pixel threshold of $p < 0.01$ was used to identify clusters. At the cluster level, a threshold of $p < 0.01$ was used to assign significance to a cluster. Fdr-bh multiple comparisons was used across the most significant (lowest p-value) cluster of each network. For results with additional test statistics and thresholds, see supplement.

Fig. 3C
Analysis: Same as previous, but with regions instead of Yeo7 networks. Regions were included if there were at least two subjects with representation of the region. Number of trials specified in parentheses.

Fig. 4B
Analysis: Average eye closure behavior on the first puff of each puff series, for pre and ketamine conditions. Traces are baseline subtracted with a 1s window before puffs. Mean and 95% confidence interval of the mean value is given (1.96 * s.e.m.), with trials averaged within session and variability taken across sessions.

Condition	# sessions	# trials	# subjects
Preinfusion	13	20	10
Ketamine	13	10	10

Fig. 4E

Analysis: Average cluster per-puff firing rate. Averages are baseline subtracted by average cluster activity 1s prior to airpuff onset. Cluster labels obtained in Fig. 3D. Mean cluster activity is z-scored across the peri-puff time window within cluster. Mean across all cells assigned to cluster, pooled across all datasets.

Condition	# cells	# airpuffs	# sessions	# subjects
Cluster 0	1070	160	13	10
Cluster 1	832	160	13	10
Cluster 2	803	160	13	10
Cluster 3	663	160	13	10
Cluster 4	639	160	13	10
Cluster 5	597	160	13	10
Cluster 6	547	160	13	10
Cluster 7	508	160	13	10
Cluster 8	473	160	13	10
Cluster 9	344	160	13	10
Cluster 10	317	160	13	10
Cluster 11	176	160	13	10

Fig 4G, fig. S31 A

Analysis: Average cluster per-puff firing rate compared between pre-infusion and ketamine infusion conditions. fig. S31 additionally shows post-infusion condition. Averages are baseline subtracted by average cluster activity 1s prior to airpuff onset. Cluster labels obtained in Fig. 4D. Mean cluster activity is z-scored across the peri-puff time window within cluster and condition. Mean across all cells assigned to cluster, pooled across all datasets. Mean ± 95% confidence interval (1.96 * s.e.m.), with variability measured across all cells within cluster.

Condition	# cells	# airpuffs	# sessions	# subjects
Cluster 0 - Pre	1070	160	13	10
Cluster 0 - Ketamine		64		
Cluster 0 - Post		64		
Cluster 1 - Pre	832	160	13	10
Cluster 1 - Ketamine		64		
Cluster 1 - Post		64		
Cluster 2 - Pre	803	160	13	10
Cluster 2 - Ketamine		64		
Cluster 2 - Post		64		
Cluster 3 - Pre	663	160	13	10
Cluster 3 - Ketamine		64		
Cluster 3 - Post		64		
Cluster 4 - Pre	639	160	13	10
Cluster 4 - Ketamine		64		
Cluster 4 - Post		64		
Cluster 5 - Pre	597	160	13	10
Cluster 5 - Ketamine		64		
Cluster 5 - Post		64		
Cluster 6 - Pre	547	160	13	10
Cluster 6 - Ketamine		64		
Cluster 6 - Post		64		

Cluster 7 - Pre	508	160	13	10
Cluster 7 - Ketamine		64		
Cluster 7 - Post		64		
Cluster 8 - Pre	473	160	13	10
Cluster 8 - Ketamine		64		
Cluster 8 - Post		64		
Cluster 9 - Pre	344	160	13	10
Cluster 9 - Ketamine		64		
Cluster 9 - Post		64		
Cluster 10 - Pre	317	160	13	10
Cluster 10 - Ketamine		64		
Cluster 10 - Post		64		
Cluster 11 - Pre	176	160	13	10
Cluster 11 - Ketamine		64		
Cluster 11 - Post		64		

Fig. 4H, I
Analysis: Rise and decay times of average regional firing rates. Peri-puff regional firing rates are averaged together within cluster identity and transformed to d' measurements on a per-time-bin basis. Time to rise is defined as the time post puff to reach 25% of the maximum d' value post puff. Time to decay is defined as the time to decay to 25% of the maximum d' value, following the peak. Regions with maximum d' less than 2 during either pre-infusion or ketamine-infusion conditions were excluded from the analysis. Regions with peak d' occurring 750ms following the puff were excluded from the analysis. After per-region per-cluster decay or rise times are obtained, they are combined per region into a single value using a weighted average, with weights determined by the fraction of cells in a given region assigned to each cluster. Point estimates given are median values across replicates of puffs. Bars indicate 95% confidence intervals of the median rise and decay values, obtained by bootstrap sampling of rise and decay times across puff replicates for each region.

Fig. 5B
Analysis: Average mouse eye closure behavior across puff series, for pre and ketamine conditions. Traces are baseline subtracted with the mean eye closure in a 1s window before each puff series. Mean and 95% confidence interval of the mean value is given (1.96 * s.e.m.), with trials averaged within session and variability taken across sessions.

Condition	# sessions	# trials	# subjects
Preinfusion	11	20	9
Ketamine	11	10	9

Fig. 5D
Analysis: Simultaneously recorded population neural activity along affective neural dimension, shown across puff series and subsequent inter-trial interval. Mean and 95% confidence interval of the mean value is given (1.96 * s.e.m.), with trials (each puff series) averaged within session and variability taken across sessions.

Condition	# sessions	# trials	# subjects
Preinfusion	11	20	9
Ketamine	11	10	9

Fig. 5E
Analysis: Mean activity along the affective neural dimension during the first puff of each puff-series, comparing

mean activity between pre-infusion and ketamine during a 0–0.25s window post-puff and during a 2–3s window post-puff. Point estimates of mean activity are obtained by averaging across trials within session. Statistics are computed on values across sessions. Error bars are given as 95% confidence intervals of the means.

Null hypothesis: No difference between each condition.

Condition	# mice	# sessions	# trials	Mean ± s.e.m.	Statistical test	Test statistic	Comparison	Corrected p-Value
0–0.25s	9	11	20 Pre, 10 Ket	Pre: 0.601 ± 0.28; Ket: 0.332 ± 0.138	Paired t-test with fdr-bh correction	1.209	Pre vs. Ketamine	0.254
2–3s	9	11	20 Pre, 10 Ket	Pre: 0.546 ± 0.174; Ket: 0.005 ± 0.043		2.874	Pre vs. Ketamine	0.033

Fig. 5G

Analysis: Comparison of simultaneously recorded population neural activity along affective neural dimension and mathematical model. Model is fit to the average within condition across sessions. Variance explained is calculated as 1–MSE_model / MSE_null, where MSE_model is the mean squared error of the fitted model prediction–allowing only the tau_persistence parameter to vary across condition–and MSE_null is the mean squared error of the fitted model with no parameter allowed to vary across condition.

Condition	# sessions	# trials	# subjects
Preinfusion	11	20	9
Ketamine	11	10	9

Fig. 5H, I

Analysis: Comparison of model fit to behavioral data across drug doses, with fits performed separately for each drug. Variance explained is calculated as described above. Only the tau_persistence parameter of the model is allowed to vary across drug dose conditions.

Condition	# sessions	# trials	# subjects
Ketamine 35 mg/kg	5	10	5
Ketamine 25 mg/kg	5	10	5
Ketamine 13 mg/kg	5	10	5
Ketamine 6 mg/kg	5	10	5
PCP 10 mg/kg	5	10	5
PCP 5 mg/kg	5	10	5
PCP 1 mg/kg	5	10	5

Fig. 6C

Analysis: Left, peri-puff population activity along persistent dimension. Mean ± 95% confidence interval (1.96 x s.e.m.). Right, average persistent population activity during 0.5 to 1.5 s after puff onset, compared between preinfusion, infusion, and postinfusion. Variation quantified across subjects, trials averaged per subject per condition.

Null hypothesis: No difference between infusion and preinfusion or postinfusion.

Condition	# human participants	Mean ± s.e.m.	Statistical test	Comparison	Test statistic	Corrected p-Value
Preinfusion	7	1.36 ± 0.16	Paired t-test with fdr-bh correction	Pre. vs. Ket.	5.887	0.002
Ketamine	7	0.41 ± 0.07				
Postinfusion	7	0.72 ± 0.11		Post vs. Ket.	3.171	0.019

Fig. 6D

Analysis: Left, peri-puff population activity along persistent dimension. Mean ± 95% confidence interval (1.96 x s.e.m.). Right, average persistent population activity during 1-2 s after puff onset, comparing preinfusion, infusion, and postinfusion. Variation quantified across sessions, trials averaged per session per condition.

Null hypothesis: No difference between infusion and preinfusion or postinfusion.

Condition	# subjects	# sessions	# trials	Mean ± s.e.m.	Statistical test	Comparison	Test statistic	Corrected p-Value
Preinfusion	9	11	20	0.86 ± 0.05	Paired t-test with fdr-bh correction	Pre. vs. Ket.	5.662	0.0004
Ketamine	9	11	10	0.02 ± 0.13				
Postinfusion	9	11	6	0.52 ± 0.21		Post vs. Ket.	2.657	0.024

Fig. 6E

Analysis: Left, peri-puff population activity along fast dimension. Mean ± 95% confidence interval (1.96 x s.e.m.). Right, average fast population activity during 0.05 to 0.10 s after puff onset, compared between preinfusion, infusion, and postinfusion, using beta band power. Variation quantified across subjects, trials averaged per subject per condition (12 to 40 trials per subject per condition).

Null hypothesis: No difference between infusion and preinfusion or postinfusion.

Condition	# human participants	Mean ± s.e.m.	Statistical test	Comparison	Test statistic	Corrected p-Value
Preinfusion	7	4.12 ± 0.31	Paired t-test with fdr-bh correction	Pre. vs. Ket.	0.432	0.681
Ketamine	7	4.00 ± 0.41				
Postinfusion	7	3.86 ± 0.38		Post vs. Ket.	0.576	0.585

Fig. 6F

Analysis: Left, peri-puff population activity along fast dimension. Mean ± 95% confidence interval (1.96 x s.e.m.). Right, average fast population activity during 0 to 70 ms after puff onset, compared between preinfusion, infusion, and postinfusion. 70 ms is chosen based on the median time to peak signal per region. Variation quantified across sessions, trials averaged per session per condition.

Null hypothesis: No difference between infusion and preinfusion or postinfusion.

Condition	# subjects	# sessions	# trials	Mean ± s.e.m.	Statistical test	Test statistic	Comparison	Corrected p-Value
Preinfusion	9	11	20	4.48 ± 0.129	Paired t-test with fdr-bh correction	–0.548	Pre. vs. Ket.	0.595
Ketamine	9	11	10	4.59 ± 0.185				
Postinfusion	9	11	6	4.32 ± 0.184		2.468	Post vs. Ket.	0.066

Fig. 6G

Analysis: Intrinsic timescale of persistent population activity during the 10 minutes preceding eyepuff assay during preinfusion and infusion conditions.

Null hypothesis: No difference between preinfusion and infusion conditions.

Condition	# human participants	Mean ± s.e.m.	Statistical test	Comparison	Test statistic	p-Value
Preinfusion	7	0.11 ± 0.01	Paired t-test	Preinfusion vs. ketamine	4.74	0.0032
Ketamine	7	0.09 ± 0.01				

Fig. 6H

Analysis: Intrinsic timescale of persistent population activity during 30s preceding each puff series, during preinfusion and infusion conditions. Statistics across session replicates. Values averaged within session across trials for each condition.

Null hypothesis: No difference between preinfusion and infusion conditions.

Condition	# subjects	# sessions	# trials	Mean ± s.e.m.	Statistical test	Comparison	Test statistic	p-Value
Preinfusion	9	11	10	0.45 ± 0.07	Paired t-test	Preinfusion vs. ketamine	3.357	0.0073
Ketamine	9	11	10	0.14 ± 0.06				

Fig. 6I

Analysis: Phase locking of persistent channels (defined as channels with greater than 90th percentile weight in the persistent population dimension), during preinfusion and infusion conditions, in the 10 minutes preceding eyepuff assay. Values are divisively normalized to preinfusion.

Null hypothesis: No difference between preinfusion and infusion conditions.

Condition	# human participants	Mean ± s.e.m.	Statistical test	Comparison	Test statistic	p-Value
Preinfusion	7	1 ± 0.00	Paired t-test	Preinfusion vs. ketamine	3.344	0.0155
Ketamine	7	0.979 ± 0.0061				

Fig. 6J

Analysis: Pairwise correlation between persistent neurons (selected as neurons with greater than 90th percentile weight in persistent population dimension), during 30s preceding each puff series, for preinfusion and infusion conditions. Statistics across session replicates. Values averaged within session across trials for each condition. Values are divisively normalized to preinfusion.

Null hypothesis: No difference between preinfusion and infusion conditions.

Condition	# subjects	# sessions	# trials	Mean ± s.e.m.	Statistical test	Comparison	Test statistic	p-Value
Preinfusion	9	11	10	1.0 ± 0.0	Paired t-test	Preinfusion vs. ketamine	7.463	2.2×10^{-5}
Ketamine	9	11	10	0.49 ± 0.07				

fig. S1 B

Analysis: Reflexive eye closure (sum of eye closure during 0.1 to 0.2 s after puff initiation), ketamine vs. saline. Point estimates for each subject are mean ± s.e.m. across trials, excluding the first two trials.

Null hypothesis: No difference between ketamine and saline conditions.

Condition	# human participants	# trials per subject	Mean ± s.e.m.	Statistical test	Test statistic	p-Value
Saline	4	38	0.84 ± 0.01	Paired t-test	1.622	0.2032
Ketamine	4	38	0.78 ± 0.04			

fig. S1 C

Analysis: Affective eye closure (sum of eye closure during 0.3 to 0.8 s after puff initiation), ketamine vs. saline. Point estimates for each subject are mean ± s.e.m. across trials, excluding the first two trials.

Null hypothesis: No difference between ketamine and saline conditions.

Condition	# human participants	# trials per subject	Mean ± s.e.m.	Statistical test	Test statistic	p-Value
Saline	4	38	0.59 ± 0.07	Paired t-test	3.653	0.0354
Ketamine	4	38	0.34 ± 0.10			

fig. S1 D

Analysis: Clinician administered dissociative states scale (CADSS), saline vs. ketamine.

Null hypothesis: No difference between ketamine and saline conditions.

Condition	# human participants	Mean ± s.e.m.	Statistical test	Test statistic	p-Value
Saline	4	0.50 ± 0.29	Paired t-test	7.034	0.006
Ketamine	4	9.75 ± 1.25			

fig. S1 E

Analysis: Puff-triggered eye closure of each human subject, averaged across trials excluding the first two trials, comparing saline and ketamine conditions.

Human subject	Condition	# trials	Plot
1	Saline	38	Mean ± s.e.m.
1	Ketamine	38	Mean ± s.e.m.
2	Saline	38	Mean ± s.e.m.
2	Ketamine	38	Mean ± s.e.m.
3	Saline	38	Mean ± s.e.m.
3	Ketamine	38	Mean ± s.e.m.
4	Saline	38	Mean ± s.e.m.
4	Ketamine	38	Mean ± s.e.m.

fig. S2 B

Analysis: Reflexive eye closure in mice during for first puff of each trial, saline vs. ketamine.

Null hypothesis: No difference between ketamine and saline conditions.

Condition	# mice	# trials per mouse	Mean ± s.e.m.	Statistical test	Test statistic	p-Value
Saline	5	15	0.78 ± 0.05	Paired t-test	6.854	0.002
Ketamine	5	15	0.45 ± 0.06			

fig. S2 C

Analysis: Affective eye closure in mice during for first puff of each trial, saline vs. ketamine.

Null hypothesis: No difference between ketamine and saline conditions.

Condition	# mice	# trials per mouse	Mean ± s.e.m.	Statistical test	Test statistic	p-Value
Saline	5	15	0.34 ± 0.07	Paired t-test	4.951	0.008
Ketamine	5	15	-0.02 ± 0.02			

fig. S2 E

Analysis: eye closure persistence after final puff of each trial, assessed as Kaplan Meier survival time.

Condition	# mice	Number of trials	Statistical test	Test statistic
Preinfusion	5	20 trials per mouse, 100 trials total	Kaplan Meier median survival time	15.16 seconds

fig. S2 G

Analysis: Scaling of normalized affective eye closure scales with puff number or puff intensity, after the eighth puff of each trial.

Null hypothesis: No difference between first and eighth puff at fixed puff intensity or between the eighth puff of two different puff intensities.

Condition	# mice	# trials per mouse	mean ± s.e.m.	Statistical test	Comparison	Test statistic	Corrected p-Value
Puff 1, 8 PSI	8	15	0.256 ± 0.067	Paired t-test with fdr-bh correction	8 PSI: Puff 1 vs. Puff 8	4.834	0.0038
Puff 8, 8 PSI	8	15	0.517 ± 0.058		18 PSI: Puff 1 vs. Puff 8	9.243	0.0001
Puff 1, 18 PSI	8	15	0.239 ± 0.032		28 PSI: Puff 1 vs. Puff 8	11.633	0.0001
Puff 8, 18 PSI	8	15	0.515 ± 0.037		Puff 8: 8 PSI vs. 18 PSI	0.024	0.982
Puff 1, 28 PSI	8	15	0.286 ± 0.068		Puff 8: 8 PSI vs. 28 PSI	1.106	0.458
Puff 8, 28 PSI	8	15	0.447 ± 0.061		Puff 8: 18 PSI vs. 28 PSI	0.718	0.596

fig. S2 H

Analysis: Effect of different doses of ketamine or PCP on normalized affective eye closure, for the eighth puff of each trial.

Null hypothesis: No difference between preinfusion and infusion at a given dose.

Condition	# mice	# trials per mouse	Mean ± s.e.m.	Statistical test	Comparison	Test statistic	Corrected p-Value
Pre-saline	5	15	0.66 ± 0.07	Paired t-test with fdr-bh correction	Pre vs. Saline	0.980	0.425
Saline	5	15	0.70 ± 0.09				
Pre-ketamine 50 mg/kg	5	15	0.66 ± 0.03		Pre vs. Ketamine 50 mg/kg	8.488	0.011
Ketamine 50 mg/kg	5	15	-0.04 ± 0.08				
Pre-ketamine 35 mg/kg	8	15	0.45 ± 0.06		Pre vs. Ketamine 35 mg/kg	3.843	0.0159
Ketamine 35 mg/kg	8	15	0.17 ± 0.07				
Pre-ketamine 25 mg/kg	5	15	0.61 ± 0.07		Pre vs. Ketamine 25 mg/kg	1.232	0.357
Ketamine 25 mg/kg	5	15	0.44 ± 0.19				
Pre-ketamine 13 mg/kg	5	15	0.57 ± 0.03		Pre vs. Ketamine 13 mg/kg	1.270	0.357

Ketamine 13 mg/kg	5	15	1.00 ± 0.34				
Pre-ketamine 6 mg/kg	5	15	0.70 ± 0.04		Pre vs. Ketamine 6 mg/kg	0.329	0.759
Ketamine 6 mg/kg	5	15	0.68 ± 0.07				
Pre-PCP 20 mg/kg	5	15	0.71 ± 0.06		Pre vs. PCP 20 mg/kg	6.080	0.016
PCP 20 mg/kg	5	15	-0.01 ± 0.07				
Pre-PCP 10mg/kg	5	15	0.72 ± 0.05		Pre vs. PCP 10 mg/kg	5.404	0.016
PCP 10 mg/kg	5	15	0.20 ± 0.13				
Pre-PCP 5 mg/kg	5	15	0.64 ± 0.06		Pre vs. PCP 5 mg/kg	2.682	0.110
PCP 5 mg/kg	5	15	0.51 ± 0.07				
Pre-PCP 1 mg/kg	5	15	0.61 ± 0.08		Pre vs. PCP 1 mg/kg	2.064	0.180
PCP 1 mg/kg	5	15	0.40 ± 0.13				

fig. S2 I

Analysis: Effect of different doses of ketamine or PCP on reflexive eye closure, for the eighth puff of each trial.

Null hypothesis: No difference between preinfusion and infusion at a given dose.

Condition	# mice	# trials per mouse	Mean ± s.e.m.	Statistical test	Comparison	Test statistic	Corrected p-Value
Pre-saline	5	15	0.78 ± 0.05	Paired t-test with fdr-bh correction	Pre vs. Saline	0.103	0.923
Saline	5	15	0.78 ± 0.05				
Pre-ketamine 50 mg/kg	5	15	0.76 ± 0.08		Pre vs. Ketamine 50 mg/kg	3.042	0.064
Ketamine 50 mg/kg	5	15	0.44 ± 0.06				
Pre-ketamine 35 mg/kg	8	15	0.69 ± 0.05		Pre vs. Ketamine 35 mg/kg	3.525	0.032
Ketamine 35 mg/kg	8	15	0.43 ± 0.06				
Pre-ketamine 25 mg/kg	5	15	0.62 ± 0.11		Pre vs. Ketamine 25 mg/kg	2.299	0.119
Ketamine 25 mg/kg	5	15	0.54 ± 0.10				
Pre-ketamine 13 mg/kg	5	15	0.73 ± 0.05		Pre vs. Ketamine 13 mg/kg	5.989	0.032
Ketamine 13 mg/kg	5	15	0.34 ± 0.07				
Pre-ketamine 6 mg/kg	5	15	0.83 ± 0.05		Pre vs. Ketamine 6 mg/kg	3.585	0.058
Ketamine 6 mg/kg	5	15	0.55 ± 0.11				
Pre-PCP 20 mg/kg	5	15	0.63 ± 0.03		Pre vs. PCP 20 mg/kg	4.842	0.032
PCP 20 mg/kg	5	15	0.43 ± 0.01				
Pre-PCP 10mg/kg	5	15	0.61 ± 0.03		Pre vs. PCP 10 mg/kg	3.220	0.064
PCP 10 mg/kg	5	15	0.44 ± 0.05				
Pre-PCP 5 mg/kg	5	15	0.51 ± 0.02		Pre vs. PCP 5 mg/kg	1.511	0.228
PCP 5 mg/kg	5	15	0.57 ± 0.04				
Pre-PCP 1 mg/kg	5	15	0.67 ± 0.05		Pre vs. PCP 1 mg/kg	2.082	0.132
PCP 1 mg/kg	5	15	0.47 ± 0.09				

fig. S2 J

Analysis: Effect of different doses of ketamine or PCP on affective eye closure, for the eighth puff of each trial.

Null hypothesis: No difference between preinfusion and infusion at a given dose.

Condition	# mice	# trials per mouse	Mean ± s.e.m.	Statistical test	Comparison	Test statistic	Corrected p-Value
Pre-saline	5	15	0.51 ± 0.05	Paired t-test with fdr-bh correction	Pre vs. Saline	0.898	0.4199
Saline	5	15	0.55 ± 0.08				
Pre-ketamine 50 mg/kg	5	15	0.49 ± 0.04		Pre vs. Ketamine 50 mg/kg	7.947	0.0068
Ketamine 50 mg/kg	5	15	-0.01 ± 0.03				
Pre-ketamine 35 mg/kg	8	15	0.31 ± 0.04		Pre vs. Ketamine 35 mg/kg	5.397	0.0068
Ketamine 35 mg/kg	8	15	0.08 ± 0.03				
Pre-ketamine 25 mg/kg	5	15	0.38 ± 0.07		Pre vs. Ketamine 25 mg/kg	1.716	0.2017
Ketamine 25 mg/kg	5	15	0.20 ± 0.08				
Pre-ketamine 13 mg/kg	5	15	0.42 ± 0.05		Pre vs. Ketamine 13 mg/kg	3.789	0.0386
Ketamine 13 mg/kg	5	15	0.27 ± 0.05				
Pre-ketamine 6 mg/kg	5	15	0.58 ± 0.06		Pre vs. Ketamine 6 mg/kg	2.252	0.1250
Ketamine 6 mg/kg	5	15	0.39 ± 0.09				
Pre-PCP 20 mg/kg	5	15	0.45 ± 0.05		Pre vs. PCP 20 mg/kg	5.750	0.0113
PCP 20 mg/kg	5	15	0.00 ± 0.03				
Pre-PCP 10mg/kg	5	15	0.44 ± 0.05		Pre vs. PCP 10 mg/kg	6.194	0.0113
PCP 10 mg/kg	5	15	0.11 ± 0.07				
Pre-PCP 5 mg/kg	5	15	0.33 ± 0.04		Pre vs. PCP 5 mg/kg	1.171	0.3406
PCP 5 mg/kg	5	15	0.29 ± 0.04				
Pre-PCP 1 mg/kg	5	15	0.42 ± 0.07		Pre vs. PCP 1 mg/kg	2.491	0.1123
PCP 1 mg/kg	5	15	0.23 ± 0.09				

fig. S2 K

Analysis: Average eye closure across trials (trials consist of 8 closely-spaced puffs), averaged across mice, comparing preinfusion and saline infusion conditions for five different sessions per mouse (one session per day).

Condition	# mice	# trials per mouse	Plot
Preinfusion	5	15	mean ± s.e.m.
Saline	5	15	mean ± s.e.m.

fig. S2 L

Analysis: Reflexive, affective, and normalized affective preinfusion eye closure across five sessions (one per day) with the same subject, for the eighth puff of each trial.

Null hypothesis: No difference between the first session and each subsequent session.

Normalized affective

Condition	# mice	# trials per mouse	Mean ± s.e.m.	Statistical test	Comparison	Test statistic	Corrected p-Value
Day 1	5	15	0.42 ± 0.06	Paired t-test with fdr-bh correction			
Day 2	5	15	0.47 ± 0.03		Day 1 vs. Day 2	0.74	0.6569
Day 3	5	15	0.31 ± 0.08		Day 1 vs. Day 3	1.38	0.5638
Day 4	5	15	0.50 ± 0.05		Day 1 vs. Day 4	1.24	0.5638
Day 5	5	15	0.48 ± 0.07		Day 1 vs. Day 5	0.48	0.6569

Reflexive

Condition	# mice	# trials per mouse	Mean ± s.e.m.	Statistical test	Comparison	Test statistic	Corrected p-Value
Day 1	5	15	0.39 ± 0.02	Paired t-test with fdr-bh correction			
Day 2	5	15	0.36 ± 0.03		Day 1 vs. Day 2	0.91	0.7425
Day 3	5	15	0.41 ± 0.05		Day 1 vs. Day 3	0.35	0.7425
Day 4	5	15	0.68 ± 0.08		Day 1 vs. Day 4	3.31	0.119
Day 5	5	15	0.43 ± 0.04		Day 1 vs. Day 5	0.63	0.7425

Affective

Condition	# mice	# trials per mouse	Mean ± s.e.m.	Statistical test	Comparison	Test statistic	Corrected p-Value
Day 1	5	15	0.17 ± 0.03	Paired t-test with fdr-bh correction			
Day 2	5	15	0.17 ± 0.01		Day 1 vs. Day 2	0.02	0.9879
Day 3	5	15	0.12 ± 0.03		Day 1 vs. Day 3	1.13	0.6418
Day 4	5	15	0.34 ± 0.05		Day 1 vs. Day 4	3.19	0.1324
Day 5	5	15	0.21 ± 0.04		Day 1 vs. Day 5	0.63	0.7475

fig. S2 M

Analysis: Average eye closure across trials (trials consist of 8 closely-spaced puffs), averaged across mice, comparing preinfusion conditions for three different airpuff pressures.

Condition	# mice	# trials per mouse	Plot
8 PSI	8	15	mean ± s.e.m.
18 PSI	8	15	mean ± s.e.m.
28 PSI	8	15	mean ± s.e.m.

fig. S2 N

Analysis: Left, average eye closure across trials (trials consist of 8 closely-spaced puffs), averaged across mice, comparing preinfusion condition for male and female mice.

Condition	# mice	# trials per mouse	Plot
Female	5	15	mean ± s.e.m.
Male	5	15	mean ± s.e.m.

Analysis: Right, normalized affective eye closure for male and female mice for the eighth puff of each trial. Paired t-test for within-subject comparisons, and independent t-test for across subject.

Null hypothesis: No difference between male and female mice.

Condition	# mice	# trials per mouse	Mean ± s.e.m.	Statistical test	Comparison	Test statistic	Corrected p-Value
Male Pre-saline	5	15	0.44 ± 0.08	T-test with fdr-bh correction	Male Pre vs. Saline	1.080	0.3827
Male saline	5	15	0.57 ± 0.11		Female Pre vs. Saline	0.980	0.3827
Female Pre-saline	5	15	0.66 ± 0.07		Pre Male vs. Female	2.079	0.2851
Female saline	5	15	0.70 ± 0.09		Saline Male vs. Female	0.983	0.3827

fig. S2 O

Analysis: Average eye closure across trials (trials consist of 8 closely-spaced puffs), averaged across mice, on trials in which either the 6th or 7th puff was skipped.

Condition	# mice	# trials per mouse	Plot
Preinfusion skip 6th puff	5	10	mean ± s.e.m.
Infusion skip 6th puff	5	10	mean ± s.e.m.
Preinfusion skip 7th puff	5	10	mean ± s.e.m.
Infusion skip 7th puff	5	10	mean ± s.e.m.

fig. S5 A

Analysis: Average eye closure across trials (trials consist of 8 closely-spaced puffs), averaged across mice, comparing preinfusion and drug infusion conditions.

Condition	# mice	# trials per mouse	Plot
Preinfusion	5	15	mean ± s.e.m.
Buprenorphine	5	15	mean ± s.e.m.

fig. S5 B

Analysis: Average eye closure across trials (trials consist of 8 closely-spaced puffs), averaged across mice, comparing preinfusion and drug infusion conditions.

Condition	# mice	# trials per mouse	Plot
Preinfusion	5	15	mean ± s.e.m.
Diazepam	5	15	mean ± s.e.m.

fig. S5 C

Analysis: Normalized affective eye closure (affective divided by reflexive) in mice for eighth puff of each trial, preinfusion vs. infusion for saline, ketamine (50 mg/kg), PCP (20 mg/kg), buprenorphine (2 mg/kg), diazepam (2 mg/kg). K/X is not shown here because loss of reflexive response makes normalized affective measure not applicable.

Null hypothesis: No difference between preinfusion and infusion conditions.

Condition	# mice	# trials per mouse	Mean ± s.e.m.	Statistical test	Comparison	Test statistic	Corrected p-Value
Pre-saline	5	15	0.66 ± 0.03		Pre-saline vs. saline	0.9797	0.4784

Saline	5	15	0.70 ± 0.09	Paired t-test with fdr-bh correction			
Pre-ketamine	5	15	0.66 ± 0.03		Pre-ketamine vs. ketamine	8.4882	0.0053
Ketamine	5	15	-0.04 ± 0.08				
Pre-PCP	5	15	0.71 ± 0.06		Pre-PCP vs. PCP	6.0804	0.0092
PCP	5	15	-0.01 ± 0.07				
Pre-bupr.	5	15	0.65 ± 0.09		Pre-bupr. vs. bupr.	0.0878	0.9342
Bupr.	5	15	0.64 ± 0.08				
Pre-diaz.	5	15	0.75 ± 0.04		Pre-diaz. vs. diaz.	1.6120	0.3038
Diaz.	5	15	0.62 ± 0.05				

fig. S5 D

Analysis: Reflexive eye closure in mice for eighth puff of each trial, preinfusion vs. infusion for saline, ketamine (50 mg/kg), PCP (20 mg/kg), buprenorphine (2 mg/kg), diazepam (2 mg/kg), K/X (135 mg/kg ketamine with 15 mg/kg xylazine).

Null hypothesis: No difference between preinfusion and infusion conditions.

Condition	# mice	# trials per mouse	Mean ± s.e.m.	Statistical test	Comparison	Test statistic	Corrected p-Value
Pre-saline	5	15	0.78 ± 0.05	Paired t-test with fdr-bh correction	Pre-saline vs. saline	0.1026	0.9232
Saline	5	15	0.78 ± 0.05				
Pre-ketamine	5	15	0.76 ± 0.08		Pre-ketamine vs. ketamine	3.0416	0.0575
Ketamine	5	15	0.44 ± 0.06				
Pre-PCP	5	15	0.63 ± 0.03		Pre-PCP vs. PCP	4.8422	0.0168
PCP	5	15	0.43 ± 0.01				
Pre-bupr.	5	15	0.54 ± 0.07		Pre-bupr. vs. bupr.	1.2745	0.3258
Bupr.	5	15	0.47 ± 0.07				
Pre-diaz.	5	15	0.78 ± 0.02		Pre-diaz. vs. diaz.	6.2356	0.0101
Diaz.	5	15	0.48 ± 0.04				
Pre-K/X	5	15	0.77 ± 0.09		Pre-K/X vs. K/X	8.6017	0.0060
K/X	5	15	0.03 ± 0.02				

fig. S5 E

Analysis: Affective eye closure in mice for eighth puff of each trial, preinfusion vs. infusion for saline, ketamine (50 mg/kg), PCP (20 mg/kg), buprenorphine (2 mg/kg), diazepam (2 mg/kg), K/X (135 mg/kg ketamine with 15 mg/kg xylazine).

Null hypothesis: No difference between preinfusion and infusion conditions.

Condition	# mice	# trials per mouse	Mean ± s.e.m.	Statistical test	Comparison	Test statistic	Corrected p-Value
Pre-saline	5	15	0.51 ± 0.05	Paired t-test with fdr-bh correction	Pre-saline vs. saline	0.8982	0.4199
Saline	5	15	0.55 ± 0.08				
Pre-ketamine	5	15	0.49 ± 0.04		Pre-ketamine vs. ketamine	7.9465	0.0041
Ketamine	5	15	-0.01 ± 0.03				
Pre-PCP	5	15	0.45 ± 0.05		Pre-PCP vs. PCP	5.7504	0.0091
PCP	5	15	0.00 ± 0.03				
Pre-bupr.	5	15	0.37 ± 0.09		Pre-bupr. vs. bupr.	1.5276	0.2416
Bupr.	5	15	0.29 ± 0.04				
Pre-diaz.	5	15	0.58 ± 0.04		Pre-diaz. vs. diaz.	3.9535	0.0252
Diaz.	5	15	0.30 ± 0.04				

Pre-K/X	5	15	0.47 ± 0.03		Pre-K/X vs. K/X	12.0602	0.0016
K/X	5	15	0.02 ± 0.01				

fig. S5 F
Analysis: Exponential decay rate of eye closure after the last puff of each trial in mice, preinfusion vs. infusion for saline, ketamine (50 mg/kg), PCP (20 mg/kg), buprenorphine (2 mg/kg), diazepam (2 mg/kg).

Null hypothesis: No difference between preinfusion and infusion conditions.

Condition	# mice	# trials per mouse	Mean ± s.e.m.	Statistical test	Comparison	Test statistic	Corrected p-Value
Pre-saline	5	15	3.33 ± 0.13	Paired t-test with fdr-bh correction	Pre-saline vs. saline	0.8204	0.7634
Saline	5	15	3.96 ± 0.77				
Pre-ketamine	5	15	3.53 ± 0.28		Pre-ketamine vs. ketamine	4.5198	0.0266
Ketamine	5	15	11.89 ± 1.74				
Pre-PCP	5	15	3.04 ± 0.46		Pre-PCP vs. PCP	5.3534	0.0266
PCP	5	15	13.20 ± 1.59				
Pre-bupr.	5	15	4.15 ± 1.15		Pre-bupr. vs. bupr.	0.4065	0.8243
Bupr.	5	15	3.60 ± 0.84				
Pre-diaz.	5	15	3.29 ± 0.78		Pre-diaz. vs. diaz.	0.2370	0.8243
Diaz.	5	15	3.03 ± 0.43				

fig. S5 G
Analysis: Average eye closure across trials (trials consist of 8 closely-spaced puffs), averaged across mice, zoomed in on the time surrounding the eighth puff of a trial, comparing preinfusion and drug infusion conditions.

Condition	# mice	# trials per mouse	Plot
Saline	5	15	mean ± s.e.m.
Ketamine	5	15	mean ± s.e.m.
PCP	5	15	mean ± s.e.m.
Buprenorphine	5	15	mean ± s.e.m.
Diazepam	5	15	mean ± s.e.m.
Ketamine/Xylazine	5	15	mean ± s.e.m.

fig. S5 H
Analysis: Example video frames immediately preceding, during and after an airpuff after infusion with different drugs.

fig. S6 B
Analysis: Average eye closure across trials (trials consist of 8 closely-spaced puffs), averaged across mice, comparing no injection (intact), saline injection, and VPM muscimol injection conditions. The conditions are paired across mice.

Condition	# mice	# trials per mouse	Plot
Intact	5	15	mean ± s.e.m.
Saline	5	15	mean ± s.e.m.
VPM Muscimol	5	15	mean ± s.e.m.

fig. S6 C
Analysis: Normalized affective eye closure (affective divided by reflexive) in mice for eighth puff in trial, comparing no injection, saline injection, and muscimol injection bilaterally in ventral posteromedial (VPM) thalamus.

Null hypothesis: No difference between each condition.

Condition	# mice	# trials per mouse	Mean ± s.e.m.	Statistical test	Comparison	Test statistic	Corrected p-Value
No injection	5	15	0.63 ± 0.03	Paired t-test with fdr-bh correction	No injection vs. saline	0.45	0.6727
Saline	5	15	0.60 ± 0.05		Saline vs. muscimol	3.85	0.0289
Muscimol	5	15	0.11 ± 0.12		No injection vs. muscimol	3.79	0.0289

fig. S6 D
Analysis: Reflexive eye closure in mice for eighth puff in trial, comparing no injection, saline injection, and muscimol injection bilaterally in ventral posteromedial (VPM) thalamus.

Null hypothesis: No difference between each condition.

Condition	# mice	# trials per mouse	Mean ± s.e.m.	Statistical test	Comparison	Test statistic	Corrected p-Value
No injection	5	15	0.45 ± 0.09	Paired t-test with fdr-bh correction	No injection vs. saline	0.37	0.7179
Saline	5	15	0.41 ± 0.07		Saline vs. muscimol	2.48	0.0614
Muscimol	5	15	0.20 ± 0.06		No injection vs. muscimol	2.43	0.0614

fig. S6 E
Analysis: Affective eye closure in mice for eighth puff in trial, comparing no injection, saline injection, and muscimol injection bilaterally in ventral posteromedial (VPM) thalamus.

Null hypothesis: No difference between each condition.

Condition	# mice	# trials per mouse	Mean ± s.e.m.	Statistical test	Comparison	Test statistic	Corrected p-Value
No injection	5	15	0.29 ± 0.06	Paired t-test with fdr-bh correction	No injection vs. saline	0.48	0.6423
Saline	5	15	0.25 ± 0.05		Saline vs. muscimol	3.96	0.0063
Muscimol	5	15	0.03 ± 0.03		No injection vs. muscimol	4.07	0.0063

fig. S6 F
Analysis: Early reflexive (within 40 ms of eyepuff onset) eye closure in mice for eighth puff in trial, comparing no injection, saline injection, and muscimol injection bilaterally in ventral posteromedial (VPM) thalamus.

Null hypothesis: No difference between each condition.

Condition	# mice	# trials per mouse	Mean ± s.e.m.	Statistical test	Comparison	Test statistic	Corrected p-Value
No injection	5	15	0.08 ± 0.01	Paired t-test with fdr-bh correction	No injection vs. saline	0.14	0.8898
Saline	5	15	0.08 ± 0.03		Saline vs. muscimol	0.37	0.8898
Muscimol	5	15	0.09 ± 0.02		No injection vs. muscimol	0.43	0.8898

fig. S6 G
Analysis: Average eye closure across trials around the first puff of a trial, averaged across mice, comparing no injection (intact), saline injection, and VPM muscimol injection conditions.

Condition	# mice	# trials per mouse	Plot
Intact	5	15	mean ± s.e.m.

Saline	5	15	mean ± s.e.m.
VPM Muscimol	5	15	mean ± s.e.m.

fig. S6 H
Analysis: Average eye closure across trials zoomed in after the first puff of a trial, averaged across mice, comparing no injection (intact), saline injection, and VPM muscimol injection conditions.

Condition	# mice	# trials per mouse	Plot
Intact	5	15	mean ± s.e.m.
Saline	5	15	mean ± s.e.m.
VPM Muscimol	5	15	mean ± s.e.m.

fig. S6 I
Analysis: Average eye closure across trials around the eighth puff of a trial, averaged across mice, comparing no injection (intact), saline injection, and VPM muscimol injection conditions.

Condition	# mice	# trials per mouse	Plot
Intact	5	15	mean ± s.e.m.
Saline	5	15	mean ± s.e.m.
VPM Muscimol	5	15	mean ± s.e.m.

fig. S6 J
Analysis: Average eye closure across trials zoomed in after the eighth puff of a trial, averaged across mice, comparing no injection (intact), saline injection, and VPM muscimol injection conditions.

Condition	# mice	# trials per mouse	Plot
Intact	5	15	mean ± s.e.m.
Saline	5	15	mean ± s.e.m.
VPM Muscimol	5	15	mean ± s.e.m.

fig. S8 A
Analysis: Comparison of normalized affective eye closure during preinfusion, ketamine infusion, and postinfusion for each human subject that met the behavioral inclusion criteria (significantly decreased eye closure during infusion relative to preinfusion and relative to postinfusion). Affective eye closure is normalized by the reflexive eye closure on each trial.

Null hypothesis: No difference between infusion and preinfusion or postinfusion.

Subject ID	Condition	# trials	Mean ± s.e.m.	Statistical test	Comparison	Test statistic	Corrected p-Value
SD168	Preinfusion	24	0.94 ± 0.01	Paired t-test with fdr-bh correction	Pre vs. Ketamine	5.8087	1.29e-05
	Ketamine		0.83 ± 0.02		Post vs. Ketamine	3.2763	0.0033
	Postinfusion		0.90 ± 0.01				
SD172	Preinfusion	39	0.53 ± 0.03	Paired t-test with fdr-bh correction	Pre vs. Ketamine	5.6281	1.84e-06
	Ketamine		0.35 ± 0.02		Post vs. Ketamine	11.7475	6.47e-14
	Postinfusion		0.75 ± 0.03				
SD181	Preinfusion	30	0.85 ± 0.04	Paired t-test with fdr-bh correction	Pre vs. Ketamine	10.5245	4.09e-11
	Ketamine		0.21 ± 0.04		Post vs. Ketamine	5.8804	2.21e-06
	Postinfusion		0.53 ± 0.04				
SD185	Preinfusion	31	0.60 ± 0.03	Paired t-test with fdr-bh correction	Pre vs. Ketamine	4.7462	4.77e-05
	Ketamine		0.45 ± 0.03		Post vs. Ketamine	6.8624	2.58e-07
	Postinfusion		0.67 ± 0.02				
SD186	Preinfusion	12	0.54 ± 0.05	Paired t-test with fdr-bh correction	Pre vs. Ketamine	2.7912	0.0236
	Ketamine		0.35 ± 0.04		Post vs. Ketamine	2.6265	0.0236
	Postinfusion		0.55 ± 0.06				

SD211	Preinfusion	39	0.31 ± 0.02	Paired t-test with fdr-bh correction	Pre vs. Ketamine	7.1858	1.38e-08
	Ketamine		0.15 ± 0.02		Post vs. Ketamine	10.4531	1.96e-12
	Postinfusion		0.36 ± 0.01				
SD245	Preinfusion	39	0.56 ± 0.02	Paired t-test with fdr-bh correction	Pre vs. Ketamine	5.2553	1.19e-05
	Ketamine		0.40 ± 0.02		Post vs. Ketamine	2.9258	0.0058
	Postinfusion		0.50 ± 0.02				

fig. S8 B
Analysis: Comparison of normalized affective eye closure during preinfusion, ketamine infusion, and postinfusion for each human subject that did not meet the behavioral inclusion criteria. Affective eye closure is normalized by the reflexive eye closure on each trial.

Null hypothesis: No difference between infusion and preinfusion or postinfusion.

Subject ID	Condition	# trials	Mean ± s.e.m.	Statistical test	Comparison	Test statistic	Corrected p-Value
SD163	Preinfusion	39	0.67 ± 0.03	Paired t-test with fdr-bh correction	Pre vs. Ketamine	0.3794	0.7065
	Ketamine		0.68 ± 0.03		Post vs. Ketamine	1.7575	0.1738
	Postinfusion		0.78 ± 0.05				
SD170	Preinfusion	39	0.13 ± 0.03	Paired t-test with fdr-bh correction	Pre vs. Ketamine	0.1429	0.8871
	Ketamine		0.12 ± 0.05		Post vs. Ketamine	1.1008	0.5558
	Postinfusion		0.05 ± 0.03				
SD178	Preinfusion	27	0.93 ± 0.02	Paired t-test with fdr-bh correction	Pre vs. Ketamine	1.7376	0.0941
	Ketamine		0.98 ± 0.02		Post vs. Ketamine	3.6936	0.0021
	Postinfusion		0.90 ± 0.01				
SD184	Preinfusion	30	0.40 ± 0.02	Paired t-test with fdr-bh correction	Pre vs. Ketamine	0.9389	0.3555
	Ketamine		0.43 ± 0.02		Post vs. Ketamine	2.4481	0.0413
	Postinfusion		0.50 ± 0.03				
SD207	Preinfusion	11	0.52 ± 0.03	Paired t-test with fdr-bh correction	Pre vs. Ketamine	4.8338	0.0014
	Ketamine		0.27 ± 0.04		Post vs. Ketamine	2.1669	0.0555
	Postinfusion		0.17 ± 0.03				
SD215	Preinfusion	39	0.33 ± 0.04	Paired t-test with fdr-bh correction	Pre vs. Ketamine	1.2251	0.2281
	Ketamine		0.21 ± 0.11		Post vs. Ketamine	2.3802	0.0448
	Postinfusion		0.45 ± 0.04				
SD242	Preinfusion	39	0.75 ± 0.02	Paired t-test with fdr-bh correction	Pre vs. Ketamine	4.8557	4.16e-05
	Ketamine		0.63 ± 0.01		Post vs. Ketamine	0.2170	0.8294
	Postinfusion		0.62 ± 0.02				

fig. S12 A
Analysis: Magnitude of event related potential (average of absolute value of local field potential for puff-triggered blinks) for each channel. Sorted by peak time on average across a train-set of trials. Plotted using average across held-out set of trials.

fig. S12 B
Analysis: Channels ordered according to (A), but with magnitude of event related potential triggered on non-puff blinks.

fig. S12 C

Analysis: Average event related potential across all channels. Event related potential magnitude is average absolute value of local field potential triggered on either onset of a puff-triggered blink or a natural (non-puff-triggered) blink. Mean traces are baseline-subtracted using the pre-blink interval.

Condition	# human participants	Total # blinks	Total # channels	Plot
Puff blink	7	52	773	mean ± s.e.m.
Non-puff blink	7	52	773	mean ± s.e.m.

fig. S12 D

Analysis: Average eye closure across blinks, triggered on either onset of a puff-triggered blink or a natural (non-puff-triggered) blink. Mean traces are baseline-subtracted using the pre-blink interval.

Condition	# human participants	Total # blinks	Plot
Puff blink	7	52	mean ± s.e.m.
Non-puff blink	7	52	mean ± s.e.m.

fig. S12 E

Analysis: Rise and decay times of average regional firing rates. Peri-puff local field potentials are transformed to d' measurements on a per-time-bin basis. The d' traces were smoothed with a 4-th order sos butterworth filter (cutoff=12.5 Hz). Time to rise is defined as the time after puff onset to reach a d' of either 0.75, 1.0, or 1.5. Regions with peak d' occurring 750ms following the puff were excluded from the analysis. Point estimates given are median values across replicates of puffs. Bars indicate 95% confidence intervals of the rise and decay values, obtained by bootstrap sampling of rise times across puff replicates for each region.

fig. S13

Analysis: Mean puff-triggered spectrogram by region. For each subject for each trial, a regional spectrogram was calculated as the mean across all channels in that region. Spectrograms were then averaged across all trials across all subjects that sampled a given region. Different regions were sampled in different subjects, so the number of trials differed.

fig. S14 G

Analysis: Spearman correlation between change in normalized affective eye closure (ketamine - preinfusion) versus Total CADSS score.

Null hypothesis: No correlation between change in normalized affective eye closure and CADSS.

Comparison	# human participants	Statistical test	Correlation coefficient	p-Value
Total CADSS vs. Change in normalized affective eye closure (ketamine - preinfusion)	10	Spearman rank-order correlation coefficient	0.744	0.014

fig. S16

Analysis: Mean puff-triggered spectrogram by region by subject.

fig. S21

Analysis: Comparison of permutation cluster test results using various parameters. Statistical test: paired t-test, independent t-test, and mixed-effect linear model with trials grouped by patient. Pixel threshold for cluster formation: p=0.05, p=0.01, p=0.001.

fig. S22

Analysis: Comparison of permutation cluster test results using various parameters. Statistical test: paired t-test,

independent t-test, independent t-test with hat variance adjustment, and mixed-effect linear model with trials grouped by patient. Pixel threshold for cluster formation: p=0.05, p=0.01, p=0.001, and threshold-free cluster enhancement (tfce) with start=0 and step=0.1.

fig. S23
Analysis: Comparison of permutation cluster test results using various parameters. Statistical test: paired t-test, independent t-test, and mixed-effect linear model with trials grouped by patient. Pixel threshold for cluster formation: p=0.05, p=0.01, p=0.001.

fig. S24
Analysis: Permutation cluster test for each channel individually, comparing infusion – preinfusion. Statistical test: paired t-test with p=0.01 pixel threshold for cluster formation. Each channel had from 12 to 39 trials per session depending on which subject the channel came from. Using the lowest cluster p-value from each channel, the p-values were fdr-bh corrected for multiple comparisons across channels. A channel was considered to have a significant change if the lowest corrected cluster p-value was < 0.05.

fig. S31 B
Analysis: fraction of recorded cells of a given cluster in each brain region, given for all regions with greater than 30 cells. P-value is calculated from a two-sided tail statistic given a per-region null distribution and corrected for multiple hypotheses using a fdr-bh correction. Regions above a corrected P-value threshold of 0.01 are labeled with the region acronym.

Null hypothesis: a given region has a uniform categorical distribution of clusters, computed by 20,000 bootstrapped cluster fractions from randomly assigned cluster labels within region.

fig. S31 F, G
Analysis: Distribution of changes (on ketamine, from pre-infusion) in rise times and decay times for all regions. Red line indicates the median of each distribution.

fig. S34 B
Analysis: Autocorrelation of puff response neural dimension of an example mouse subject for preinfusion and ketamine conditions, with exponential fit, f(t) = a*exp(-t/b)+c), where the intrinsic timescale is operationalized as the decay rate, b.

fig. S34 C
Analysis: eye closure across tone-series, for preinfusion and ketamine conditions. Mean ± 95% CI (1.96 x s.e.m.). Variation quantified across sessions, trials averaged within session.

Condition	# subjects	# sessions	# trials
Preinfusion	7	7	10
Ketamine	7	7	5
Postinfusion	7	7	3

fig. S34 D
Analysis: Left, peri-tone population activity along tone fast dimension. Mean ± 95% confidence interval (1.96 x s.e.m.). Right, average tone fast population activity during 0 to 70 ms after tone onset, compared between preinfusion, infusion, and postinfusion. Variation quantified across sessions, trials averaged per session per condition.

Null hypothesis: No difference between infusion and preinfusion or postinfusion.

Condition	# subjects	# sessions	# trials	Mean ± s.e.m.	Statistical test	Comparison	Test statistic	Corrected p-Value
Preinfusion	7	7	10	2.49 ± 0.245	Paired t-test with fdr-bh correction	Pre. vs. Ket.	1.088	0.318
Ketamine	7	7	5	1.83 ± 0.399				
Postinfusion	7	7	3	1.56 ± 0.275		Post vs. Ket.	1.438	0.318

fig. S34 E
Analysis: Left, peri-tone population activity along tone persistent dimension (difference between average activity in the window [150 ms, 350 ms] and baseline). Mean ± 95% confidence interval (1.96 x s.e.m.). Right, average tone persistent population activity during 350 ms to 500 ms after tone onset, comparing preinfusion, infusion, and postinfusion. Variation quantified across sessions, trials averaged per session per condition.

Null hypothesis: No difference between infusion and preinfusion or postinfusion.

Condition	# subjects	# sessions	# trials	Mean ± s.e.m.	Statistical test	Comparison	Test statistic	Corrected p-Value
Preinfusion	7	7	10	1.531 ± 0.081	Paired t-test with fdr-bh correction	Pre. vs. Ket.	6.942	0.0009
Ketamine	7	7	5	0.392 ± 0.177				
Postinfusion	7	7	3	1.385 ± 0.338		Post vs. Ket.	–3.753	0.009

fig. S34 F
Analysis: Comparison of timecourse of detectability of activity along the persistent dimension for eyepuff vs. tone. Detectability is calculated as d-prime. Mean ± 95% CI.

fig. S34 G
Analysis: Intrinsic timescale of tone persistent population activity during 30s preceding each tone series, during preinfusion and infusion conditions. Statistics across session replicates. Values averaged within session across trials for each condition.

Null hypothesis: No difference between preinfusion and infusion conditions.

Condition	# subjects	# sessions	# trials	Mean ± s.e.m.	Statistical test	Comparison	Test statistic	p-Value
Preinfusion	7	7	10	0.268 ± 0.037	Paired t-test	Preinfusion vs. ketamine	5.479	0.0015
Ketamine	7	7	5	0.089 ± 0.012				

fig. S34 H
Analysis: Pairwise correlation between tone persistent neurons (selected as neurons with greater than 90th percentile weight in tone persistent population dimension), during 30s preceding each tone series, for preinfusion and infusion conditions. Statistics across session replicates. Values averaged within session across trials for each condition.

Null hypothesis: No difference between preinfusion and infusion conditions.

Condition	# subjects	# sessions	# trials	Mean ± s.e.m.	Statistical test	Comparison	Test statistic	p-Value
Preinfusion	7	7	10	1.0 ± 0.0	Paired t-test	Preinfusion vs. ketamine	6.217	0.0008
Ketamine	7	7	5	0.460 ± 0.080				

fig. S35 A
Analysis: Participation (loading) of each channel in the Fast coding dimension (left) or Persistent coding dimension (right). For each plot, the loadings are normalized by the maximum value.

fig. S35 B
Analysis: Participation (loading) of each channel in the Fast coding dimension across all subjects. The loadings for each subject are normalized by the maximum value of that subject.

fig. S35 C
Analysis: Aggregate participation (loading) of each Yeo7 resting state network in the Fast coding dimension. Median across all channels in a network, with 95% confidence interval errorbars. To determine whether a network has over- or under-represented in the coding dimension, bootstrap shuffling is used, in which the network label assigned to each channel is shuffled, with 1000 shuffles. Dashed lines represent the upper and lower cutoffs for two-sided significance at p=0.05. P-values are computed based on the percentile of the true value among the shuffles (p=0.05 corresponds to 97.5 or 2.5 percentile), and are corrected across networks with fdr-bh.

Null hypothesis: A network does not have more or less loading on the Fast coding dimension than other networks.

Yeo7 network	# subjects	# channels	Mean ± s.e.m.	Statistical test	Corrected p-Value
Default	7	88	0.126 ± 0.014	Bootstrap shuffle (network labels) with fdr-bh correction across networks	0.1056
Dorsal Attention	5	18	0.080 ± 0.027		0.0440
Frontoparietal	5	23	0.070 ± 0.027		0.0180
Limbic	7	34	0.115 ± 0.018		0.1720
Somatomotor	7	50	0.446 ± 0.045		0.0000
Ventral Attention	7	63	0.209 ± 0.032		0.0960

fig. S35 D
Analysis: Same as previous panel but for Persistent coding dimension.

Null hypothesis: A network does not have more or less loading on the Persistent coding dimension than other networks.

Yeo7 network	# subjects	# channels	Mean ± s.e.m.	Statistical test	Corrected p-Value
Default	7	88	0.239 ± 0.021	Bootstrap shuffle (network labels) with fdr-bh correction across networks	0.9740
Dorsal Attention	5	18	0.292 ± 0.060		0.9740
Frontoparietal	5	23	0.350 ± 0.037		0.7920
Limbic	7	34	0.233 ± 0.035		0.9740
Somatomotor	7	50	0.253 ± 0.030		0.9740
Ventral Attention	7	63	0.246 ± 0.032		0.9740

fig. S37 A

Analysis: Pearson correlation coefficient between mean preinfusion persistent coding dimension projection (across subjects) for mouse firing and human bandpower, for each frequency band.

fig. S37 B

Analysis: Left, peri-puff population activity along persistent dimension. Mean ± s.e.m. Right, average persistent population activity during 0.05 to 0.10 s after puff onset, compared between preinfusion, infusion, and postinfusion, using different frequency bands.

Null hypothesis: No difference between infusion and preinfusion or postinfusion.

Frequency band	Condition	# human participants	Mean ± s.e.m.	Statistical test	Comparison	Test statistic	Corrected p-Value
Delta (1-4 Hz)	Preinfusion	7	3.20 ± 0.10	Paired t-test with fdr-bh correction	Pre. vs. Ket.	0.817	0.859
	Ketamine		3.15 ± 0.13				
	Postinfusion		3.14 ± 0.10		Post vs. Ket.	0.183	0.859
Theta (4-8 Hz)	Preinfusion	7	3.83 ± 0.05	Paired t-test with fdr-bh correction	Pre. vs. Ket.	0.274	0.793
	Ketamine		3.84 ± 0.06				
	Postinfusion		3.86 ± 0.04		Post vs. Ket.	0.428	0.793
Alpha (8-12 Hz)	Preinfusion	7	3.89 ± 0.04	Paired t-test with fdr-bh correction	Pre. vs. Ket.	0.848	0.508
	Ketamine		3.93 ± 0.06				
	Postinfusion		3.89 ± 0.07		Post vs. Ket.	0.703	0.508
Beta (12-30 Hz)	Preinfusion	7	4.12 ± 0.31	Paired t-test with fdr-bh correction	Pre. vs. Ket.	0.432	0.681
	Ketamine	7	4.00 ± 0.41				
	Postinfusion	7	3.86 ± 0.38		Post vs. Ket.	1.063	0.657
Gamma (30-50 Hz)	Preinfusion	7	3.19 ± 0.24	Paired t-test with fdr-bh correction	Pre. vs. Ket.	1.245	0.273
	Ketamine	7	2.89 ± 0.26				
	Postinfusion	7	3.18 ± 0.12		Post vs. Ket.	1.207	0.273
High Gamma (65-95 Hz)	Preinfusion	7	3.64 ± 0.37	Paired t-test with fdr-bh correction	Pre. vs. Ket.	0.053	0.960
	Ketamine	7	3.64 ± 0.35				
	Postinfusion	7	3.71 ± 0.46		Post vs. Ket.	0.371	0.960
Broadband LFP	Preinfusion	7	2.35 ± 0.59	Paired t-test with fdr-bh correction	Pre. vs. Ket.	0.816	0.761
	Ketamine	7	1.88 ± 0.88				
	Postinfusion	7	1.99 ± 0.81		Post vs. Ket.	0.319	0.761

fig. S37 C

Analysis: Left, peri-puff population activity along persistent dimension. Mean ± s.e.m. Right, average persistent population activity during 0.5 to 1.5 s after puff onset, compared between preinfusion, infusion, and postinfusion, using different frequency bands.

Null hypothesis: No difference between infusion and preinfusion or postinfusion.

Frequency band	Condition	# human participants	Mean ± s.e.m.	Statistical test	Comparison	Test statistic	Corrected p-Value
Delta (1-4 Hz)	Preinfusion	7	0.79 ± 0.16	Paired t-test with fdr-bh correction	Pre. vs. Ket.	3.930	0.015
	Ketamine		0.18 ± 0.07				
	Postinfusion		0.44 ± 0.12		Post vs. Ket.	2.502	0.046
Theta (4-8 Hz)	Preinfusion	7	0.82 ± 0.22	Paired t-test with fdr-bh correction	Pre. vs. Ket.	3.246	0.035
	Ketamine		0.11 ± 0.08				
	Postinfusion		0.35 ± 0.13		Post vs. Ket.	1.935	0.101

Alpha (8-12 Hz)	Preinfusion	7	0.97 ± 0.21	Paired t-test with fdr-bh correction	Pre. vs. Ket.	4.620	0.007
	Ketamine		0.25 ± 0.12				
	Postinfusion		0.47 ± 0.18		Post vs. Ket.	1.777	0.126
Beta (12-30 Hz)	Preinfusion	7	1.36 ± 0.16	Paired t-test with fdr-bh correction	Pre. vs. Ket.	5.887	0.002
	Ketamine	7	0.41 ± 0.07				
	Postinfusion	7	0.72 ± 0.11		Post vs. Ket.	3.171	0.019
Low Gamma (30-50 Hz)	Preinfusion	7	1.32 ± 0.08	Paired t-test with fdr-bh correction	Pre. vs. Ket.	6.252	0.002
	Ketamine	7	0.49 ± 0.17				
	Postinfusion	7	0.52 ± 0.14		Post vs. Ket.	0.383	0.715
High Gamma (65-95 Hz)	Preinfusion	7	0.90 ± 0.11	Paired t-test with fdr-bh correction	Pre. vs. Ket.	6.870	0.001
	Ketamine	7	0.27 ± 0.13				
	Postinfusion	7	0.39 ± 0.08		Post vs. Ket.	1.189	0.279
Broadband LFP	Preinfusion	7	1.66 ± 0.05	Paired t-test with fdr-bh correction	Pre. vs. Ket.	2.625	0.079
	Ketamine	7	1.37 ± 0.14				
	Postinfusion	7	1.49 ± 0.08		Post vs. Ket.	0.790	0.460

fig. S38 A
Analysis: Mixed effects linear model, grouped by subject, of preinfusion phase locking ~ high vs. low CD edge identity (categorical).

Null hypothesis: No correlation between preinfusion phase locking and an edge's identity as being between high vs. low CD channels.

Frequency band	# human participants	# inter-channel edges	Statistical test	Comparison	Coefficient	Confidence interval 95%	R^2	Corrected p-value
Delta (1-4 Hz)	7	716	Mixed-effects linear model with fdr-bh correction	Low vs. High	0.0017	[-0.005, 0.008]	1.3e-4	0.76
Theta (4-8 Hz)	7	716		Low vs. High	0.0026	[-0.004, 0.009]	-7.4e-4	0.76
Alpha (8-12 Hz)	7	716		Low vs. High	0.0017	[-0.006, 0.009]	-7.6e-4	0.76
Beta (12-30 Hz)	7	716		Low vs. High	0.0016	[-0.005, 0.008]	-3.7e-4	0.76
Gamma (30-50 Hz)	7	716		Low vs. High	0.0018	[-0.004, 0.007]	3.7e-4	0.76
High Gamma (65-95 Hz)	7	716		Low vs. High	-0.00060	[-0.006, 0.005]	4.6e-5	0.83

fig. S38 B
Analysis: Mixed effects linear model, grouped by subject, of phase locking change on ketamine ~ high vs. low CD edge identity (categorical).

Null hypothesis: No correlation between ketamine-induced change in an edge's phase locking and an edge's identity as being either high or low CD channels.

Frequency band	# human participants	# inter-channel edges	Statistical test	Comparison	Coefficient	Confidence interval 95%	R^2	Corrected p-value
Delta (1-4 Hz)	7	716	Mixed-effects linear model with fdr-bh correction	Low vs. High	-0.0038	[-0.024, 0.016]	-0.0093	0.70
Theta (4-8 Hz)	7	716		Low vs. High	-0.016	[-0.038, 0.005]	-0.00068	0.16

Alpha (8-12 Hz)	7	716		Low vs. High	-0.028	[-0.050, -0.007]	-0.0063	0.021
Beta (12-30 Hz)	7	716		Low vs. High	-0.038	[-0.057, -0.020]	0.020	0.00026
Gamma (30-50 Hz)	7	716		Low vs. High	-0.023	[-0.040, -0.006]	0.0080	0.019
High Gamma (65-95 Hz)	7	716		Low vs. High	-0.012	[-0.028, 0.003]	0.0031	0.16

fig. S38 D
Analysis: Mixed effects linear model, grouped by subject, of preinfusion power ~ high vs. low CD channel identity (categorical).

Null hypothesis: No correlation between a channel's pre-infusion power and a channel's identity as being high vs. low CD.

Frequency band	# human participants	# channels	Statistical test	Comparison	Coefficient	Confidence interval 95%	R^2	Corrected p-value
Delta (1-4 Hz)	7	104	Mixed-effects linear model with fdr-bh correction	Low vs. High	-2.36	[-4.6, -0.094]	0.0092	0.24
Theta (4-8 Hz)	7	104		Low vs. High	-1.28	[-3.49, 0.91]	0.0011	0.48
Alpha (8-12 Hz)	7	104		Low vs. High	-0.43	[-2.80, 1.93]	-0.0025	0.71
Beta (12-30 Hz)	7	104		Low vs. High	-0.74	[-2.82, 1.33]	-0.0011	0.57
Gamma (30-50 Hz)	7	104		Low vs. High	-0.99	[-2.97, 0.98]	-0.0014	0.48
High Gamma (65-95 Hz)	7	104		Low vs. High	-1.63	[-3.53, 0.25]	0.0015	0.26

fig. S38 E
Analysis: Mixed effects linear model, grouped by subject, of power change from preinfusion to ketamine ~ high vs. low CD channel identity (categorical).

Null hypothesis: No correlation between a channel's change in power on ketamine and a channel's identity as being high vs. low CD.

Frequency band	# human participants	# channels	Statistical test	Comparison	Coefficient	Confidence interval 95%	R^2	Corrected p-value
Delta (1-4 Hz)	7	104	Mixed-effects linear model with fdr-bh correction	Low vs. High	-0.19	[-0.47, 0.075]	0.0041	0.46
Theta (4-8 Hz)	7	104		Low vs. High	-0.11	[-0.39, 0.15]	0.0043	0.79
Alpha (8-12 Hz)	7	104		Low vs. High	-0.047	[-0.38, 0.28]	0.00063	0.80
Beta (12-30 Hz)	7	104		Low vs. High	0.079	[-0.40, 0.56]	0.00022	0.80
Gamma (30-50 Hz)	7	104		Low vs. High	0.87	[0.11, 1.63]	0.032	0.14
High Gamma (65-95 Hz)	7	104		Low vs. High	0.081	[-0.56, 0.73]	0.00044	0.80

fig. S38 H

Analysis: Mixed effects linear model, grouped by subject, of **single-trial** post-puff affective (late) window CD projection ~ pre-puff network-wide beta phase locking.

Null hypothesis: No correlation on single trials between the post-puff late projection and pre-puff beta phase locking.

Condition	# human participants	# total trials	Statistical test	Coefficient	Coeff 95% CI	R^2	p-value
Preinfusion	7	214	Mixed-effects linear model	12.12	[5.19, 19.04]	0.057	6.04e-4
Infusion	7	214		-2.15	[-6.78, 2.47]	0.00083	0.361

fig. S38 K

Analysis: Mixed effects linear model, grouped by subject, of **single-trial** post-puff affective eye closure ~ pre-puff network-wide beta phase locking.

Null hypothesis: No correlation on single trials between the post-puff eye closure behavior and pre-puff beta phase locking.

Condition	# human participants	# total trials	Statistical test	Coefficient	Confidence interval 95%	R^2	p-value
Preinfusion	7	214	Mixed-effects linear model	3.35	[1.26, 5.45]	0.034	0.0017
Infusion	7	214		-0.065	[-2.83, 2.70]	-0.0080	0.96

References

60. G. Lopes, N. Bonacchi, J. Frazão, J. P. Neto, B. V. Atallah, S. Soares, L. Moreira, S. Matias, P. M. Itskov, P. A. Correia, R. E. Medina, L. Calcaterra, E. Dreosti, J. J. Paton, A. R. Kampff, Bonsai: an event-based framework for processing and controlling data streams. *Front. Neuroinform.* **9**, 7 (2015).

61. A. Mathis, P. Mamidanna, K. M. Cury, T. Abe, V. N. Murthy, M. W. Mathis, M. Bethge, DeepLabCut: markerless pose estimation of user-defined body parts with deep learning. *Nat. Neurosci.* **21**, 1281–1289 (2018).

62. L. M. Hack, X. Zhang, B. D. Heifets, T. Suppes, P. J. van Roessel, J. A. Yesavage, N. J. Gray, R. Hilton, C. Bertrand, C. I. Rodriguez, K. Deisseroth, B. Knutson, L. M. Williams, Ketamine's acute effects on negative brain states are mediated through distinct altered states of consciousness in humans. *Nat. Commun.* **14**, 1–11 (2023).

63. A. Anand, D. S. Charney, D. A. Oren, R. M. Berman, X. S. Hu, A. Cappiello, J. H. Krystal, Attenuation of the neuropsychiatric effects of ketamine with lamotrigine: support for hyperglutamatergic effects of N-methyl-D-aspartate receptor antagonists. *Arch. Gen. Psychiatry* **57**, 270–276 (2000).

64. L. Yamada, T. Oskotsky, P. Nuyujukian, Stanford Comprehensive Epilepsy Center, Stanford Pediatric Epilepsy Center, A scalable platform for acquisition of high-fidelity human intracranial EEG with minimal clinical burden. *PLoS One* **19**, e0305009 (2024).

65. D. M. Groppe, S. Bickel, A. R. Dykstra, X. Wang, P. Mégevand, M. R. Mercier, F. A. Lado, A. D. Mehta, C. J. Honey, iELVis: An open source MATLAB toolbox for localizing and visualizing human intracranial electrode data. *J. Neurosci. Methods* **281**, 40–48 (2017).

66. B. Fischl, FreeSurfer. *Neuroimage* **62**, 774–781 (2012).

67. M. Jenkinson, P. Bannister, M. Brady, S. Smith, Improved optimization for the robust and accurate linear registration and motion correction of brain images. *Neuroimage* **17**, 825–841 (2002).

68. D. N. Greve, B. R. Fischl, A boundary-based cost function for within-subject, cross-modal registration. *Neuroimage* **47**, S100 (2009).

69. M. Jenkinson, S. Smith, A global optimisation method for robust affine registration of brain images. *Med. Image Anal.* **5**, 143–156 (2001).

70. X. Papademetris, M. P. Jackowski, N. Rajeevan, M. DiStasio, H. Okuda, R. T. Constable, L. H. Staib, BioImage Suite: An integrated medical image analysis suite: An update. *Insight J.* **2006**, 209 (2006).

71. A. R. Dykstra, A. M. Chan, B. T. Quinn, R. Zepeda, C. J. Keller, J. Cormier, J. R. Madsen, E. N. Eskandar, S. S. Cash, Individualized localization and cortical surface-based registration of intracranial electrodes. *Neuroimage* **59**, 3563–3570 (2012).

72. M. R. Mercier, S. Bickel, P. Megevand, D. M. Groppe, C. E. Schroeder, A. D. Mehta, F. A. Lado, Evaluation of cortical local field potential diffusion in stereotactic electro-encephalography recordings: A glimpse on white matter signal. *Neuroimage* **147**, 219–232 (2017).

73. E. C. Robinson, K. Garcia, M. F. Glasser, Z. Chen, T. S. Coalson, A. Makropoulos, J. Bozek, R. Wright, A. Schuh, M. Webster, J. Hutter, A. Price, L. Cordero Grande, E. Hughes, N. Tusor, P. V. Bayly, D. C.

Van Essen, S. M. Smith, A. D. Edwards, J. Hajnal, M. Jenkinson, B. Glocker, D. Rueckert, Multimodal surface matching with higher-order smoothness constraints. *Neuroimage* **167**, 453–465 (2018).

74. M. J. Prerau, R. E. Brown, M. T. Bianchi, J. M. Ellenbogen, P. L. Purdon, Sleep neurophysiological dynamics through the lens of multitaper spectral analysis. *Physiology (Bethesda)* **32**, 60–92 (2017).

75. A. Gramfort, M. Luessi, E. Larson, D. A. Engemann, D. Strohmeier, C. Brodbeck, R. Goj, M. Jas, T. Brooks, L. Parkkonen, M. Hämäläinen, MEG and EEG data analysis with MNE-Python. *Front. Neurosci.* **7**, 267 (2013).

76. P. Virtanen, R. Gommers, T. E. Oliphant, M. Haberland, T. Reddy, D. Cournapeau, E. Burovski, P. Peterson, W. Weckesser, J. Bright, S. J. van der Walt, M. Brett, J. Wilson, K. J. Millman, N. Mayorov, A. R. J. Nelson, E. Jones, R. Kern, E. Larson, C. J. Carey, İ. Polat, Y. Feng, E. W. Moore, J. VanderPlas, D. Laxalde, J. Perktold, R. Cimrman, I. Henriksen, E. A. Quintero, C. R. Harris, A. M. Archibald, A. H. Ribeiro, F. Pedregosa, P. van Mulbregt, SciPy 1.0 Contributors, SciPy 1.0: fundamental algorithms for scientific computing in Python. *Nat. Methods* **17**, 261–272 (2020).

77. J. P. Lachaux, E. Rodriguez, J. Martinerie, F. J. Varela, Measuring phase synchrony in brain signals. *Hum. Brain Mapp.* **8**, 194–208 (1999).

78. M. X. Cohen, *Analyzing Neural Time Series Data* (MIT Press, London, England, 2014)*Issues in Clinical and Cognitive Neuropsychology*.

79. C. R. Harris, K. J. Millman, S. J. van der Walt, R. Gommers, P. Virtanen, D. Cournapeau, E. Wieser, J. Taylor, S. Berg, N. J. Smith, R. Kern, M. Picus, S. Hoyer, M. H. van Kerkwijk, M. Brett, A. Haldane, J. F. Del Río, M. Wiebe, P. Peterson, P. Gérard-Marchant, K. Sheppard, T. Reddy, W. Weckesser, H. Abbasi, C. Gohlke, T. E. Oliphant, Array programming with NumPy. *Nature* **585**, 357–362 (2020).

80. R. Bruña, F. Maestú, E. Pereda, Phase locking value revisited: teaching new tricks to an old dog. *J. Neural Eng.* **15**, 056011 (2018).

81. T. Donoghue, M. Haller, E. J. Peterson, P. Varma, P. Sebastian, R. Gao, T. Noto, A. H. Lara, J. D. Wallis, R. T. Knight, A. Shestyuk, B. Voytek, Parameterizing neural power spectra into periodic and aperiodic components. *Nat. Neurosci.* **23**, 1655–1665 (2020).

82. J. R. Manning, J. Jacobs, I. Fried, M. J. Kahana, Broadband shifts in local field potential power spectra are correlated with single-neuron spiking in humans. *J. Neurosci.* **29**, 13613–13620 (2009).

83. D. C. Van Essen, S. M. Smith, D. M. Barch, T. E. J. Behrens, E. Yacoub, K. Ugurbil, WU-Minn HCP Consortium, The WU-Minn Human Connectome Project: an overview. *Neuroimage* **80**, 62–79 (2013).

84. M. F. Glasser, S. N. Sotiropoulos, J. A. Wilson, T. S. Coalson, B. Fischl, J. L. Andersson, J. Xu, S. Jbabdi, M. Webster, J. R. Polimeni, D. C. Van Essen, M. Jenkinson, The minimal preprocessing pipelines for the Human Connectome Project. *Neuroimage* **80**, 105–124 (2013).

85. S. N. Sotiropoulos, S. Jbabdi, J. Xu, J. L. Andersson, S. Moeller, E. J. Auerbach, M. F. Glasser, M. Hernandez, G. Sapiro, M. Jenkinson, D. A. Feinberg, E. Yacoub, C. Lenglet, D. C. Van Essen, K. Ugurbil, T. E. J. Behrens, Advances in diffusion MRI acquisition and processing in the Human Connectome Project. *Neuroimage* **80**, 125–143 (2013).

86. B. B. Avants, N. J. Tustison, M. Stauffer, G. Song, B. Wu, J. C. Gee, The Insight ToolKit image registration framework. *Front. Neuroinform.* **8**, 44 (2014).

87. K. Niemann, V. R. Mennicken, D. Jeanmonod, A. Morel, The Morel stereotactic atlas of the human thalamus: atlas-to-MR registration of internally consistent canonical model. *Neuroimage* **12**, 601–616 (2000).

88. M. Jenkinson, C. F. Beckmann, T. E. J. Behrens, M. W. Woolrich, S. M. Smith, FSL. *Neuroimage* **62**, 782–790 (2012).

89. J. D. Hunter, Matplotlib: A 2D Graphics Environment. *Comput. Sci. Eng.* **9**, 90–95 (2007).

90. W. McKinney, "Data Structures for Statistical Computing in Python" in *Proceedings of the Python in Science Conference* (SciPy, 2010), pp. 56–61.

91. M. Waskom, seaborn: statistical data visualization. *J. Open Source Softw.* **6**, 3021 (2021).

92. F. Pedregosa, G. Varoquaux, A. Gramfort, V. Michel, B. Thirion, O. Grisel, M. Blondel, A. Müller, J. Nothman, G. Louppe, P. Prettenhofer, R. Weiss, V. Dubourg, J. Vanderplas, A. Passos, D. Cournapeau, M. Brucher, M. Perrot, É. Duchesnay, Scikit-learn: Machine Learning in Python, *arXiv [cs.LG]* (2012). http://arxiv.org/abs/1201.0490.

93. M. Pachitariu, S. Sridhar, J. Pennington, C. Stringer, Spike sorting with Kilosort4. *Nat. Methods* **21**, 914–921 (2024).

94. erichamc, *Erichamc/Brainwide-Npix: Release v0.1* (Zenodo, 2025; http://dx.doi.org/10.5281/ZENODO.14768678).

95. C.-L. Chiu, N. Clack, the napari community, Napari: A python multi-dimensional image viewer platform for the research community. *Microsc. Microanal.* **28**, 1576–1577 (2022).

96. A. Syeda, L. Zhong, R. Tung, W. Long, M. Pachitariu, C. Stringer, Facemap: a framework for modeling neural activity based on orofacial tracking. *Nat. Neurosci.* **27**, 187–195 (2024).

97. F. A. Wolf, P. Angerer, F. J. Theis, SCANPY: large-scale single-cell gene expression data analysis. *Genome Biol.* **19**, 15 (2018).

98. L. McInnes, J. Healy, J. Melville, UMAP: Uniform Manifold Approximation and Projection for Dimension Reduction, *arXiv [stat.ML]* (2018). http://arxiv.org/abs/1802.03426.

99. V. A. Traag, L. Waltman, N. J. van Eck, From Louvain to Leiden: guaranteeing well-connected communities. *Sci. Rep.* **9**, 5233 (2019).

100. I. Kauvar, E. Richman, T. Liu, C. Li, C. Rodriguez, V. Buch, P. Nuyujukian, K. Deisseroth, Materials and contributors associated with Kauvar*, Richman*, Liu* et al., Science 2025 (Stanford Digital Repository, 2025); https://purl.stanford.edu/kw028qk2071

101. I. Kauvar, E. Richman, T. Liu, C. Li, C. Rodriguez, V. Buch, P. Nuyujukian, K. Deisseroth, Brain-wide human electrophysiology during aversive stimuli and ketamine (Zenodo, 2025); https://zenodo.org/records/14920570.

102. I. Kauvar, E. Richman, T. Liu, C. Li, C. Rodriguez, V. Buch, P. Nuyujukian, K. Deisseroth, Brain-wide human electrophysiology during aversive stimuli and ketamine (Dandi, 2025); https://dandiarchive.org/dandiset/001347.

103. E. Richman, Electrophysiological and behavioral analyses related to Kauvar*, Richman*, Liu* et al. 2025 (Zenodo, 2025); https://zenodo.org/records/14876694.

104. I. Kauvar, E. Richman, T. Liu, C. Li, C. Rodriguez, V. Buch, P. Nuyujukian, K. Deisseroth, Electrophysiological and behavioral analyses related to Kauvar*, Richman*, Liu* et al. 2025 (Dandi, 2025); https://dandiarchive.org/dandiset/001326.
